# Differences in Serum Cytokine Profiles Between Vegetarians and Omnivores: Exploratory Assessment of Dietary Factors

**DOI:** 10.3390/metabo16100793

**Published:** 2026-10-19

**Authors:** Ivana Šarac, Slavko Mojsilović, Marta Despotović, Milan Ivanov, Marija Paunović, Jelena Milešević, Marija Takić

**Affiliations:** 1 Group for Nutrition and Metabolism, Centre of Research Excellence in Nutrition and Metabolism, Institute for Medical Research, National Institute of the Republic of Serbia, University of Belgrade, 11000 Belgrade, Serbia; marta.despotovic@imi.bg.ac.rs (M.D.); jelena.milesevic@imi.bg.ac.rs (J.M.); marija.takic@imi.bg.ac.rs (M.T.); 2 Group for Hematology and Stem Cells, Institute for Medical Research, National Institute of the Republic of Serbia, University of Belgrade, 11000 Belgrade, Serbia; slavko@imi.bg.ac.rs; 3 Group for Cardiovascular Physiology, Institute for Medical Research, National Institute of the Republic of Serbia, University of Belgrade, 11000 Belgrade, Serbia; ivmilan@imi.bg.ac.rs; 4 Group for Nutritional Biochemistry and Dietology, Centre of Research Excellence in Nutrition and Metabolism, Institute for Medical Research, National Institute of the Republic of Serbia, University of Belgrade, 11000 Belgrade, Serbia; marija.paunovic@imi.bg.ac.rs

**Keywords:** cytokine, chemokine, immunity, inflammation, plant-based, vegetarian, vegan, diet, nutrition

## Abstract

**Background/Objectives:** Evidence suggests that plant-based diets are associated with reduced risk of chronic inflammatory diseases and lower circulating C-reactive protein (CRP) levels. However, associations with other inflammatory biomarkers, including interleukins (ILs), tumor necrosis factors (TNFs), interferons (IFNs), and chemokines, remain limited and inconclusive. This study compared multiple serum cytokine and chemokine levels between healthy vegetarians and omnivores. **Methods:** 160 apparently healthy, normal-weight participants aged 25–50 years, without acute and chronic illnesses or allergies, were included: 80 omnivores and 80 vegetarians (67 vegans and 13 lacto-ovo vegetarians). Serum concentrations of 13 cytokines/chemokines were measured using flow cytometry with a LEGENDplex™ bead-based multiplex immunoassay kit. Anthropometry, standard clinical hematological and biochemistry parameters, CRP, and dietary intakes were also analyzed. **Results:** The groups did not differ by sex (50% women), age, smoking, physical activity, or anthropometrics. Vegetarians had lower levels of TNF-α, IL-6, IL-8, IL-10, IL-12p70, IL-17A, IFN-γ, and IFN-α2, as well as lower leukocyte and lymphocyte counts, with no difference in IL-1β, IL-18, IL-33, IL-23, MCP-1, or CRP. The differences persisted after adjustment for potential confounders (although fewer remained significant after multiple-testing correction), and were principally present in both vegetarian subgroups, alongside substantial differences in multiple dietary factors. Regression analyses further confirmed the association of vegetarian dietary patterns with cytokine levels that differed. **Conclusions:** Vegetarian dietary patterns were associated with lower circulating concentrations of selected cytokines/chemokines in this cohort. However, the number of associations remaining after multiple-testing correction was limited; there was high multicollinearity between dietary factors, and causality could not be established. Further research is needed to elucidate causal relationships, specific dietary factors, underlying mechanisms, and clinical implications.

## 1. Introduction

For the immune response to be effective in eliminating pathogens during acute and chronic infections, as well as in the prevention and management of allergies, autoimmune, and chronic diseases, a balanced diet with an optimal intake of all essential nutrients is required [1,2,3,4,5,6,7]. Optimal nutritional status is, therefore, crucial for proper immune function and depends on adequate intake of macronutrients (proteins, fats, carbohydrates, and dietary fiber), micronutrients (vitamins and minerals), and protective bioactive compounds from both plant and animal sources [1,2,3,4,5,6,7].

Vegetarian and other plant-based diets have become increasingly popular in recent years due to their potential health and environmental benefits [8]; they are associated with several health benefits compared to omnivorous diets, including a lower risk of obesity, metabolic disorders (e.g., diabetes, dyslipidemia, and metabolic syndrome), cardiovascular diseases, chronic inflammatory and degenerative conditions, and certain cancer forms [3,8,9,10,11,12,13].

However, despite their many advantages, plant-based diets may also have certain drawbacks, particularly regarding the adequate intake of specific macro- and micronutrients and bioactive compounds, and not all plant-based diets are equally nutritious or healthy [13,14,15,16,17]. Numerous studies have shown that vegetarians, and particularly vegans, may exhibit subclinical nutritional deficiencies in minerals (including Fe, Zn, I, Se, Ca, and Cu), vitamins (especially B12 and D, but also A, B2, B3, and B6), and n-3 fatty acids (as well as an unfavorable n-3/n-6 fatty acids ratio [14,16,17,18,19,20]. They can also exhibit lower intakes of proteins [15,17], particularly certain amino acids, and animal-derived antioxidants (e.g., carnitine, carnosine, taurine, and coenzyme Q10) and bioactive peptides with antimicrobial and immunomodulatory effects [18,21,22,23]. Moreover, plant-based diets can contain anti-nutrients (like oxalates, phytates, and enzyme inhibitors), which may interfere with the absorption and metabolism of certain nutrients [13,14,18,24]. Importantly, supplementation and fortification do not necessarily ensure nutritional adequacy, with their effects varying across nutrients [20].

On the other hand, plant-based diets can provide an increased intake of many protective and anti-inflammatory compounds, including dietary fiber, vitamins C, E, and B9 (folates), polyphenols, carotenoids, glucosinolates, isothiocyanates, and other plant-derived antioxidants, as well as reduced intake of pro-inflammatory saturated fatty acids [13,17,23]. They can modulate gut microbiota and contribute to body weight reduction, and in that way indirectly modify immune response [3,13].

The differences in the intake of certain foods, and consequently in the intake of specific macro- and micronutrients and protective compounds, may also influence the immune system. In agreement with the differences in nutrient intakes, many differences have been observed between vegetarian and omnivorous dietary patterns in terms of immune status and immune response. Recent studies suggest that levels of various immune markers may be altered in individuals following vegetarian diets. For example, differences have been observed in inflammatory markers such as cytokines, including interleukin (IL)-1β and IL-6, as well as in the TNF (tumor necrosis factor)-α/IL-10 ratio, and lower levels of acute-phase proteins, including C-reactive protein (CRP), ferritin, and fibrinogen, and lower prostaglandin E2 (PGE2) [25,26,27,28,29,30,31,32,33,34]. Additionally, reductions in immune cell numbers were shown, considering leukocytes, neutrophils, lymphocytes, monocytes, eosinophils, basophils, platelets, and some of their subpopulations, particularly natural killer (NK) cells, CD4+ and CD8+ T lymphocytes, and B lymphocytes [25,32,35,36,37,38]. Furthermore, lower levels of antibodies (IgG and IgE) have been demonstrated [32,39]. Finally, changes in immune cell activity were shown, including reduced T-cell proliferation after mitogen stimulation, decreased phagocytic activity of neutrophils and monocytes, reduced production of reactive oxygen species (ROS), decreased cytotoxic activity of NK cells, and a shift from a pro-inflammatory (such as Th1 and Th17) toward an anti-inflammatory (T regulatory, Treg) immune response [1,32,38,40].

However, studies investigating vegetarian diets and immune response are relatively scarce and often yield conflicting results, preventing definitive conclusions [25,26,27,28,41,42,43]. Additionally, many of these studies have been conducted in individuals with pre-existing autoimmune conditions (e.g., ulcerative colitis, atopic dermatitis) and altered immune status, and have involved dietary transitions from omnivorous to vegetarian patterns. Other studies without dietary intervention have compared vegetarians and omnivores but often included specific populations (e.g., pregnant women, elderly individuals, patients with polycystic ovary syndrome or diabetes). Furthermore, many studies had a small sample size or a short intervention duration. Additionally, the predominant types and composition of vegetarian diets likely varied across the populations included in previous studies, potentially contributing to the observed inconsistencies in findings.

Given the increasing prevalence of vegetarianism and the substantial heterogeneity of vegetarian diets worldwide (e.g., vegan vs. lacto-ovo vegetarian) [14,44,45], it is important to investigate their potential associations with immune status also in the Serbian population, for which data remain scarce [15]. Moreover, due to inconsistent findings regarding the effects of vegetarian versus omnivorous diets on immune function, more comprehensive studies are needed that carefully control for potential confounding factors (such as sex, age, smoking status, physical activity, obesity and metabolic syndrome, presence of diseases, and use of medications or supplements that could affect immunity), in addition to considering the type and duration of vegetarian diet adherence [29,41]. Importantly, there is a lack of studies examining specific characteristics of vegetarian diets (e.g., intake of individual macro- and micronutrients, consumption of specific foods) and nutritional status indicators (e.g., body levels of n-3/n-6 fatty acids, vitamins, and minerals), and their direct association with immune status and response. In other words, very few studies have performed correlation or regression analyses between nutritional parameters (intake or status of nutrients) and immune parameters, particularly with proper adjustment for confounding factors. Addressing some of these scientific gaps represents an important objective and novel contribution of the present study.

Accordingly, in an effort to bridge some of these scientific gaps, the present study aimed to compare serum levels of multiple cytokines and chemokines between apparently healthy normal-weight vegetarians and omnivores living in Serbia, using flow cytometry with a commercially available LEGENDplex™ bead-based multiplex immunoassay kit, while adjusting for sex, age, anthropometrics, and lifestyle factors. Additionally, the study aimed to analyze the association between cytokine/chemokine levels and anthropometric parameters, standard clinical hematological and biochemistry markers, CRP, and dietary intake of specific macro- and micronutrients while adjusting for relevant lifestyle and demographic confounders.

## 2. Materials and Methods

### 2.1. Participants and Study Design

This cross-sectional study was conducted in accordance with the ethical principles of the Declaration of Helsinki and approved by the Institute for Medical Research University of Belgrade Ethics Board (Ethics Committee approval No. EO123/2017, dated 8 December 2017), within the framework of the EFSA (European Food Safety Authority)-supported project “Support to National Dietary Surveys in Compliance with the EU Menu Methodology (sixth support): The adults’ survey, including subjects from 10 to 74 years old” [46]. All participants in the study provided written informed consent prior to participation, after being thoroughly informed about the study objectives and methods.

The timeline of the study is given in the Supplementary Materials.

The study included 80 vegetarian and 80 omnivorous participants (40 males and 40 females in each group), aged 25–50 years, with normal weight (body mass index, BMI, ranging from 18.5–25 kg/m^2^), and recruited from Belgrade (the capital city).

The minimum required sample size was initially estimated based on data from a previous study directly comparing cytokine levels between vegetarians and omnivores [34]. Based on the reported effect size for IL-6, the calculation indicated that approximately 86 participants per group would be required. After recruitment had commenced, an additional relevant study by Wawrzyniak-Gramacka et al. [47], which compared inflammatory markers between individuals with lower and higher systemic inflammation, was identified. The inflammation status in that study was assessed based on serum CRP concentrations <3 and ≥3 mg/L. This threshold is also consistent with commonly used CRP classifications, in which concentrations >3 mg/L are considered associated with increased risk of chronic diseases [48,49,50]. Although this study did not directly compare dietary groups, we considered its findings useful for estimating the magnitude of a biologically meaningful difference in circulating cytokines, since our primary objective was to identify differences between vegetarians and omnivores that would be considered physiologically relevant. Using the reported TNF-α levels (pooled SD = 23.8 pg/mL; mean difference = 18.3 pg/mL between groups with higher and lower systemic inflammation), the minimum required sample size was calculated using the formula for sample size per group: N = 2σ^2^ (Z_1_ − α/2 + Z_1_ − β/2)^2^/δ^2^, with α = 0.05 and β = 0.2, corresponding to a two-sided significance level of 5% and 80% statistical power [47,51]. This calculation indicated that at least 42 participants per group were required. Considering the estimates from these two studies and the practical aspects of recruitment, we continued recruitment until 80 participants were included in each group (160 participants in total). This sample size was substantially larger than the estimate based on the inflammation-related study and was close to the more conservative estimate derived from the direct vegetarian–omnivore comparison, while also allowing for potential exclusions and the planned secondary analyses. Given the simultaneous assessment of 13 cytokines, an additional sample-size calculation was performed using a Bonferroni-adjusted significance level (α = 0.05/13 = 0.00385). This yielded an estimated requirement of 75 participants per group. The final sample of 80 participants per group therefore also met the sample-size requirement under this more conservative significance threshold.

Inclusion criteria were as follows: (1) for vegetarians, adherence to a vegetarian diet for at least two years prior to study enrollment (i.e., exclusion of meat and fish for lacto-ovo vegetarians, and exclusion of all animal-derived foods for vegans); and (2) for omnivores, regular consumption of meat (i.e., intake of meat and meat products at least 1–2 times per week) for at least two years prior to enrollment. Additional inclusion criteria were: (3) age between 25 and 50 years; (4) normal body weight (BMI between 18.5–25 kg/m^2^); and (5) residence in the Belgrade area.

Exclusion criteria were as follows: (1) presence of acute or chronic inflammatory diseases or autoimmune diseases, based on medical history, CRP levels, complete blood cell count, and erythrocyte sedimentation rate (ESR); (2) presence of allergies, based on medical history; (3) presence of other diseases or conditions affecting immune status (e.g., congenital or acquired immunodeficiency, malignancies, diabetes, hyperlipidemia, pregnancy or lactation, undernutrition or obesity, participation in weight loss programs or significant weight changes within the past 6 months); (4) use of medications affecting immune function (e.g., immunosuppressive or immunomodulatory therapy); (5) consumption of only fish (“pescatarian diet”) without consumption of meat; (6) reported total daily energy intake substantially differing from individual energy requirements calculated using the Mifflin–St Jeor equation and the Goldberg cut-off method, taking into account sex, age, height, weight, and physical activity level (PAL) [52,53] (the Black’s suggested coefficient of variation values for calculations of the cut-offs were used; the lower and upper cut-offs were, respectively, 0.616 and 1.625 for the ratio between individual energy intakes and estimated energy requirements; the respective PALs for energy requirements calculations were 1.4, 1.6, and 1.8 for sedentary, moderately active, and active participants, respectively [52,53]); (7) regular very high levels of physical activity, including athletes and heavy physical work; and (8) incomplete study assessments or data collection.

These criteria were applied to reduce group heterogeneity and exclude extreme values in dietary intake, physical activity, and nutritional status, thereby minimizing the influence of potential confounding factors. Additionally, participant recruitment was evenly distributed across all seasons, with equal numbers of men and women in both dietary groups (10 men and 10 women per group and season).

A flow chart of the study population selection is given in Supplementary Figure S1.

### 2.2. General and Lifestyle Data and Anthropometric Measurements

Face-to-face interviews were conducted utilizing a general questionnaire on sociodemographic, lifestyle (smoking, physical activity), and health-related information, including medical histories and medication usage. Self-reported physical activity levels were categorized as sedentary, moderately active, and active. Body height, weight, waist, and hip circumferences were measured according to standardized protocols [46]. Supplementary calculations included body mass index (BMI), waist-to-hip ratio (WHR), and waist-to-height ratio (WHtR) [54,55].

### 2.3. Laboratory Hematological and Biochemical Analyses of Blood Samples

Before blood sampling, participants were instructed to avoid intensive physical activity for three days and to consume their last meal prior to 20:00 on the preceding day, to allow for a 12 h fasting period [56,57]. Blood samples were then collected the following morning between 08:00 and 09:00 using BD Vacutainer blood collection tubes (Becton Dickinson, Franklin Lakes, NJ, USA). Blood cell count (including leukocytes, neutrophils, lymphocytes, monocytes, basophils, and eosinophils), hematocrit, hemoglobin, mean corpuscular volume (MCV), mean corpuscular hemoglobin (MCH), and mean corpuscular hemoglobin concentration (MCHC) analyses were performed using a DxH 500 hematology analyzer (Beckman Coulter, Brea, CA, USA). For measurement of serum glucose, total cholesterol, high-density lipoprotein (HDL)-cholesterol, low-density lipoprotein (LDL)-cholesterol, triglycerides, urea, creatinine, total bilirubin, uric acid, total protein, serum Fe, alkaline phosphatase (ALP), alanine aminotransferase (ALT), aspartate aminotransferase (AST), and γ-glutamyl transferase (GGT), serum samples were allowed to clot for at least 30 min in an upright position at room temperature, followed by centrifugation at 3000× *g* for 10 min. After centrifugation, biochemical parameters were measured using standard spectrophotometric methods on an ARCHITECT c8000 clinical chemistry analyzer (Abbott Laboratories, Abbott Park, IL, USA) using the original manufacturer’s reagents, calibrators, and control materials. Intra- and inter-assay coefficients of variation (CVs) for all measurements were below 5%.

### 2.4. Measurements of Serum Cytokines/Chemokines and CRP

Immediately after the analyses on the ARCHITECT c8000 clinical chemistry analyzer were completed, and within 2 h of blood collection, 0.5–1 mL serum aliquots were transferred from the serum coagulation tubes into 2-mL Eppendorf microtubes (Sarstedt, Nümbrecht, Germany) and stored at −80 °C until further analysis, without freeze–thaw cycles. Samples intended for cytokine/chemokine analysis were stored for 1–2 years, whereas those used for CRP determination were stored for 4–5 years. According to the available literature, storage at −80 °C for these periods does not adversely affect the majority of measured analytes [56,58,59]. Before analysis, the samples were thawed and vortex-mixed. Subsequently, 25 μL and 0.2 mL aliquots were transferred to prepared V-bottom plates and sample preparation vials for cytokine/chemokine and CRP analyses, respectively. Serum concentrations of cytokines and chemokines, including: IL-1β, IFN-α2, IFN-γ, TNF-α, monocyte chemoattractant protein-1 (MCP-1)/CCL2, IL-6, IL-8/CXCL8, IL-10, IL-12p70 (i.e., the biologically active heterodimeric IL-12), IL-17A, IL-18, IL-23, and IL-33 were determined by flow cytometry using a commercially available bead-based multiplex immunoassay kit, LEGENDplex™ Human Inflammation Panel 1 (13-plex) (Cat. No. 740809, BioLegend, San Diego, CA, USA) [60,61,62]. Briefly, the assay is based on a sandwich immunoassay principle employing distinct fluorescent bead populations coated with analyte-specific capture antibodies and phycoerythrin (PE)-conjugated detection antibodies. Cytokine concentrations were quantified according to PE fluorescence intensity using standard curves generated within the same analytical run. Data acquisition was performed on a BD FACSLyric™ flow cytometer (BD Biosciences, Franklin Lakes, NJ, USA), while data analysis was conducted using the proprietary QOGNIT online analysis software suite (version 2023-02-15; BioLegend, San Diego, CA, USA). All procedures, including preparation of standard curves and sample processing, were performed in accordance with the manufacturer’s instructions [61]. All cytokine samples were analyzed in a single analytical run on two 96-well plates. Laboratory personnel were not blinded to dietary group during sample processing and analysis. Samples from vegetarians and omnivores were analyzed in parallel and were not run in technical duplicates due to financial constraints. A quality-control sample was analyzed after every 20 study samples (four control samples per plate). Three calibration curves were included: two at the beginning of the run and one at the end of the second plate. No differences were observed between plates in control sample results or calibration curves. Information on the lower limit of detection (LOD) and lower and upper limits of quantification (LLOQ and ULOQ), and the percentage of measurements below LLOQ are given in the Supplementary Materials, Supplementary Table S1. No measured values exceeded the upper limit of the calibration range. CRP was measured by the immunoturbidimetric method on an automated BA 200 analyzer (BioSystems, Barcelona, Spain), using a commercial reagent kit from the same manufacturer and original calibrators and control materials. According to the manufacturers’ specifications, intra- and inter-assay CVs were, respectively, 2.1–4.3% and 6.6–21.9% for cytokine/chemokine measurements, and <5% and <7% for CRP determination. To reduce the inter-assay variation, all analyses of cytokines/chemokines and CRP were performed in a single batch run, within one day, with vegetarians and omnivores analyzed in parallel. During our measurements, CVs for cytokines/chemokines ranged 3.9–9.3% based on eight replicates of a control serum sample, while the CV for CRP was 3.7% based on three replicates of a control serum sample, evenly distributed throughout the batch run.

### 2.5. Assessment of Dietary Intakes

Dietary intake data were collected by a trained Ph.D. student using a twice-repeated 24 h dietary recall method (performed at a minimum of 7 days apart, covering all days of the week), in accordance with the EFSA EU Menu methodology [15,46]. The first dietary recall was conducted on the day of blood sampling, covering the food intake the previous day, while the second recall was within one month. During 24 h recalls, participants provided detailed information on consumed food, beverages, and dietary supplements. Portion sizes were estimated from food packaging or by the validated Food Atlas for Portion Size Estimation for the Balkan Region [46,63]. Additionally, to check the habitual diet over the year, the Food Propensity Questionnaire (FPQ) was applied [46]. The mean daily intakes of energy, macronutrients, and micronutrients were calculated for each participant based on their 24 h recall information on food, beverages, and supplement consumption using the DIET ASSESS & PLAN (DAP)—Advanced platform and software, version 04/2026. This software comprises the Serbian Food Composition Database (version 04/2026), developed in compliance with the European Food Information Resource Network (EuroFIR) standards, with yield and retention factors applied for recipe calculations [64,65,66]. Dietary supplement use was assessed using both FPQ data, which captured the frequency of intake of selected supplements, and 24 h dietary recalls, which additionally provided information on supplement doses. The dietary inflammatory index (DII) [67,68] was calculated using the algorithm developed by Shivappa et al. [69], employing the pro- or anti-inflammatory effects of available dietary components calculated by the DAP. Of the 45 originally proposed DII components [69], 28 were available in our dietary database for inclusion in the overall DII score. Those incorporated energy, proteins, carbohydrates, total fats, saturated fats, monounsaturated fats, polyunsaturated fats, n-3, n-6, trans-fats, cholesterol, dietary fiber, alcohol, β-carotene, vitamin A in retinol equivalents (RE), vitamins B1, B2, B3, B6, B9, B12, C, D, and E, and the minerals Fe, Mg, Se, and Zn. The remaining 17 components of the original DII—caffeine, eugenol, garlic, ginger, green/black tea, isoflavones, onion, flavan-3-ol, flavones, flavonols, flavonones, anthocyanidins, pepper, thyme/oregano, rosemary, saffron, and turmeric—were not available in the dietary database and were therefore not included. Similar adaptations of the original DII based on the availability of dietary components in food composition databases have been used in previous epidemiological studies [70,71,72,73,74]. The energy-adjusted dietary inflammatory index (E-DII) was also calculated, according to the algorithm by Petermann-Rocha et al., to account for inter-individual differences in energy intake [75,76,77], using the same 28-component approach.

### 2.6. Statistics

Data were analyzed using SPSS software (version 26.0, IBM, USA).

The distribution of continuous variables was estimated using the Kolmogorov–Smirnov test, along with skewness and kurtosis values, standard errors, and visual inspection of histograms, Q–Q plots, and box plots. As almost all continuous variables showed a non-normal distribution, they were presented as median and interquartile range (IQR), and non-parametric methods were applied. Differences between vegetarians and omnivores were analyzed using the Mann–Whitney U test and Quade’s non-parametric analysis of covariance (ANCOVA) when adjusting for age, sex, BMI, smoking, and physical activity levels [78,79,80], with homogeneity of variances assessed using a non-parametric Levene’s test. Categorical variables were expressed as counts (*n*) and percentages (%), and differences in their distribution were assessed using Pearson’s chi-square test or Fisher’s exact test, as appropriate.

To account for multiple comparisons, outcome variables were classified into primary, secondary, and exploratory analyses. The 13 cytokines/chemokines constituted the primary outcome family, and the Benjamini–Hochberg false discovery rate (FDR) correction was applied for multiple comparisons, with *q* < 0.05 considered statistically significant [81]. Nominal *p*-values without correction were presented in tables, but those that remained significant after the FDR correction were denoted. The hematological, biochemical, and dietary parameters constituted separate secondary outcome families and were likewise corrected for multiple comparisons by the Benjamini–Hochberg FDR correction, as separate outcome families. CRP was analyzed separately as a single inflammatory biomarker and was therefore not included in the cytokine/hematological outcome families. General and anthropometric variables were considered exploratory analyses and were reported using nominal *p*-values without multiplicity adjustment in comparison analyses. All primary and secondary outcomes’ comparisons between omnivore and vegetarian dietary patterns were corrected for multiple comparisons. The Mann–Whitney U and Quade’s ANCOVA analyses were considered complementary analyses of the same outcome variables, and multiple-testing corrections were applied separately to each analytical approach.

Supplementary exploratory stratified analyses were performed to compare vegans and lacto-ovo vegetarians with omnivores and with each other. The stratified analyses comparing vegans, lacto-ovo vegetarians, and omnivores were not included in the primary multiple-testing correction framework because their purpose was to assess the robustness and consistency of the primary vegetarian-versus-omnivore findings across vegetarian subgroups. Accordingly, these results are presented as supportive sensitivity analyses and are interpreted cautiously.

Additionally, sensitivity analyses were performed to assess the robustness of the findings to the potential influence of analytical limitations related to cytokine concentrations below the LLOQ. In the primary analysis, concentrations below the LLOQ were assigned a value of LLOQ/2, as a common analytical approach. All statistical analyses were then repeated using the original measured concentrations, including values below the LLOQ, without substitution by LLOQ/2. These analyses were presented as supplementary data, but included the primary multiple-testing correction framework.

Additional exploratory analyses were performed to examine associations between circulating cytokine/chemokine concentrations and general, anthropometric, biochemical, hematological, CRP, and dietary factors using unadjusted and adjusted (i.e., partial) Spearman’s rank correlations (adjusted for BMI, physical activity level, smoking status, sex, and age) [82]. Although these analyses covered several biologically distinct analytical domains and involved numerous individual associations, they were exploratory in nature and were not defined a priori as confirmatory hypotheses. They were included in the separate multiple-testing correction framework for each family of outcomes (e.g., separate associations with biochemical, hematological, anthropometric, dietary variables, etc.), and the corresponding FDR correction was applied. The results of these analyses are consequently interpreted as exploratory and hypothesis-generating rather than as confirmatory evidence.

We also performed sensitivity analyses, including Mann–Whitney test, Quade’s ANCOVA, and non-adjusted and adjusted (i.e., partial) Spearman’s rank correlations, in which participants with statistical upper-tail outliers/extreme cytokine values were excluded, and the primary analyses were repeated. Observations identified as upper-tail outliers (>1.5 × IQR) or extreme values (>3 × IQR) based on SPSS boxplots and Tukey’s fences were excluded from the analysis. Due to the limited number of subjects retained in the reduced dataset, the originally measured values below the LLOQ were retained to avoid an excessive proportion of values being represented by LLOQ/2 imputation, particularly in the correlation analyses. This analysis was conducted to assess the robustness of the observed findings to the potential influence of extreme observations. This analysis was intended as a supportive robustness check rather than an additional primary outcome analysis, and it was included in the primary multiple-testing correction framework. The results of this sensitivity analysis are reported as Supplementary Materials.

Multiple linear regression analyses were performed to examine associations between dietary pattern and serum cytokine/chemokine concentrations. Cytokine/chemokine concentrations were log-transformed before regression analyses. The primary predictor was dietary pattern (vegetarian vs. omnivorous), while sex, age, BMI, smoking status, and physical activity level were included a priori as potential confounders, based on available scientific data [47,57,83,84,85,86]. Both forced-entry and stepwise regression models were performed. The forced-entry models were used to assess the association between dietary pattern and cytokine/chemokine concentrations after adjustment for the prespecified covariates, whereas stepwise regression was performed as an exploratory analysis to identify predictors retained by the data-driven selection procedure. Because dietary pattern was the primary predictor of interest, FDR correction was applied across the 13 cytokine/chemokine outcomes only for this predictor significance. Due to space limitations, the full main model is presented in the Supplementary Materials. Additional inclusion of covariates was allowed in sensitivity analyses in case there was a significant association demonstrated with cytokine levels in our correlation analyses. Model assumptions and potential influential observations were assessed for all multiple linear regression models. Residual distributions were evaluated using histograms and normal P–P plots, which were visually inspected for departures from normality. Homoscedasticity was assessed by inspection of plots of standardized residuals against standardized predicted values. Potentially influential observations were evaluated using standardized and studentized residuals, leverage values, and Cook’s distance. Multicollinearity was assessed using tolerance and variance inflation factor (VIF) values. These diagnostic procedures were applied to all regression models; however, the complete diagnostic output for each individual model was not presented in the main manuscript because of space limitations.

All statistical tests were two-sided, and nominal *p*-values are reported in the tables, with *p* < 0.05 considered statistically significant. Asterisk (*) and dagger (†) symbols indicate *p*-values that remained statistically significant after correction for multiple testing using the Benjamini–Hochberg FDR procedure, where applicable. In all analyses, there were no missing data.

## 3. Results

### 3.1. General Data

The two groups were not different in terms of sex, age, body height and weight, BMI, waist, hip, WHR, WHtR, physical activity, and smoking (Table 1). In both groups, the majority were never smokers, and the percentage of current smokers was low. Although a doubled proportion of current smokers was observed among participants following the omnivorous diet, current smoking (current smokers vs. never and former smokers combined) was not different between the two groups (*p* = 0.127). Furthermore, no differences in general data were observed between vegans and lacto-ovo vegetarians (Supplementary Table S2).

### 3.2. Standard Serum Biochemistry Parameters

Vegetarians had lower levels of serum glucose, HDL-, LDL-, and total cholesterol, urea, creatinine, and GGT, while having higher ALP. Correction for sex, age, BMI, physical activity level, and smoking did not influence those differences, except for glucose (Table 2). After the Benjamini–Hochberg FDR correction for multiple comparisons within biochemical variables, the differences in ALP and GGT also disappeared.

Most of those differences were associated with the vegan dietary pattern, while lacto-ovo vegetarians, compared to omnivores, had only lower LDL- and total cholesterol, triglycerides, and urea (Supplementary Table S3). No differences in biochemical parameters were observed between vegans and lacto-ovo vegetarians, except for lower HDL-cholesterol and higher ALP among vegans. However, the disproportionately small number of lacto-ovo vegetarians limited the statistical power and scope of the subgroup analyses.

### 3.3. Standard Blood Cell Count Parameters and Serum CRP Levels

Vegetarians had lower total leucocyte and lymphocyte counts, higher MCV, lower MCHC, and a marginally lower eosinophil count (*p* = 0.0499). The counts of neutrophils and monocytes were also lower, but did not reach statistical significance. After adjusting for sex, age, BMI, physical activity level, and smoking, erythrocyte count and hemoglobin levels were also lower, but the difference in eosinophil count disappeared (*p* = 0.059) (Table 3). These differences remained statistically significant after FDR correction for multiple comparisons. Surprisingly, we did not find differences in serum CRP levels.

No significant differences in these parameters were observed between vegans and lacto-ovo vegetarians (Supplementary Table S4). Both vegetarian subgroups exhibited noticeably lower levels compared to omnivores; however, statistical significance was reached mainly among vegans, while among lacto-ovo vegetarians (due to a small number of participants, only 13), the statistical difference was reached only for lower MCHC.

### 3.4. Serum Cytokine/Chemokine Levels

Vegetarians had lower levels of IFN-α2, IFN-γ, TNF-α, IL-6, IL-8, IL-10, IL-12p70, and IL-17A, while no differences were observed for IL-1β, MCP-1, IL-18, IL-23, and IL-33 (although difference for IL23 was with the borderline significance, *p* = 0.077). After adjusting for sex, age, BMI, physical activity level, and smoking, the observed differences remained (Table 4). However, after the additional Benjamini–Hochberg FDR correction for multiple comparisons, the significance of IFN-α2 was lost in the unadjusted analyses, whereas in the adjusted analyses, only IL-17A, IL-8, IFN-γ, and IL-12p70 remained statistically significant.

No significant differences in cytokine/chemokine levels were observed between vegans and lacto-ovo vegetarians (Supplementary Table S5). Both subgroups exhibited observably lower levels compared to omnivores; however, statistical significance was not reached for IFN-α2, IL-6, and IL-10 among lacto-ovo vegetarians (due to a small number of participants), and for TNF-α among vegans. As noted above, the relatively small number of lacto-ovo vegetarians restricted the statistical power and limited the extent of meaningful subgroup analyses compared to vegans and omnivores.

Two additional sensitivity analyses were performed. To assess the robustness of the findings to the potential influence of analytical limitations related to cytokine concentrations below the LLOQ, the analyses were repeated using the original measured concentrations for values below the LLOQ, rather than substituting these values with LLOQ/2. The results were almost identical to those of the primary analyses (Supplementary Table S6).

Moreover, because cytokine concentrations showed considerable inter-individual variability, with several participants displaying outlying or extreme values across multiple cytokines, an additional sensitivity analysis was performed to assess whether the observed differences were driven predominantly by a limited number of extreme observations. Participants identified as having upper-tail outliers or extreme values according to Tukey’s fence for any cytokine were excluded from the analysis. Exclusions were performed at the participant level because the same individuals frequently exhibited extreme values for several cytokines. The resulting reduced dataset contained a similar number of vegetarians and omnivores (*n*N = 42 and *n*N = 40, respectively), and sex distribution was not altered (vegetarians: 22 men, 20 women; omnivores: 20 men, 20 women) compared to the primary dataset. The analyses were then repeated in this reduced dataset. Despite the substantially smaller sample size and, consequently, the expected reduction in statistical power, the statistical significance of several observed differences was maintained or even strengthened. All statistically significant differences had *p* < 0.007 and remained significant after FDR correction (Supplementary Table S7). In the reduced dataset, IL-23 and IL-33 also showed significant between-group differences, whereas only IL-1β, IL-18, and MCP-1 remained non-significant.

Interestingly, there was a high positive within-person intercorrelation between various cytokine/chemokine levels (*p* < 0.0001 for almost all comparisons, including IL-10), while MCP-1 (and, to a lesser extent, IL-1β and IL-18) showed the lowest correlations with other cytokines (Supplementary Table S8).

### 3.5. Dietary Intakes of Energy, Macronutrients, Micronutrients, and Different Food Groups

In the omnivorous group, the intake of meat was substantial (providing 16.2% of energy), while the intake of fish and sea products was negligible (Table 5). In the vegetarian group, the amount and proportion of energy coming from dairy products, eggs, and sweets were significantly lower than in the omnivorous group, while the amount and proportion of energy coming from vegetables, fruits, and nuts/seeds were higher (Table 5). The intake of edible oils/fats was lower only when expressed in absolute terms, while the amount and proportion of calories coming from cereals, alcoholic and non-alcoholic beverages were not different. All these differences in food group consumption remained significant even after correcting for sex, age, BMI, smoking status, and physical activity level. The same remained also after the FDR correction for multiple comparisons.

Differences were equally pronounced when compared to both vegans and lacto-ovo vegetarians, except for fats/oils and sweets, which were not different between lacto-ovo vegetarians and omnivores (Supplementary Table S9). When comparing vegans and lacto-ovo vegetarians, apart from differences in milk and egg product intakes, we only observed differences regarding higher intake of vegetables among vegans.

In the vegetarian group, the total daily energy intake and energy intake per kg body mass were much lower than in the omnivorous group, particularly due to a lower intake of proteins and fats, while the intake of carbohydrates and dietary fiber was higher, both in absolute terms and expressed as a percentage of energy (Table 6). In the vegetarian group, 17.5% of subjects had a protein intake lower than 0.66 g/kg/day, 26.3% had lower than 0.8 g/kg/day, while 56.3% had lower than 1.0 g/kg/day—corresponding, respectively, to the EFSA Average Requirements (ARs), Population Reference Intakes (PRIs) [87], and Italian Society of Human Nutrition (SINU) recommendations [16,88] for protein intake per kg of body mass. In contrast, in the omnivorous group, those percentages were 0.0%, 5.0%, and 7.5%, respectively (Pearson chi-square test or Fisher’s exact test, *p* < 0.001 for all). The intake and energy proportion of alcohol were no different. The intake of cholesterol, saturated, monounsaturated, polyunsaturated fats (particularly n-6 polyunsaturated fats), and trans-fats was also lower in the vegetarian group, while the intake of n-3 polyunsaturated fats was not different when expressed in absolute terms. However, when expressed in relative terms, the proportion of energy coming from n-3 fats became significantly higher in the omnivorous group, while the difference in the n-6 fats intake was lost. In addition, the n-6/n-3 fatty acid ratio was substantially higher in vegetarians. All these differences in macronutrient intakes remained significant even after the corrections for sex, age, BMI, smoking status, and physical activity. The differences remained even after the FDR correction for multiple comparisons.

Differences compared to omnivores were more observed among vegans, while among lacto-ovo vegetarians, only differences in protein, saturated fats, trans-fats, cholesterol, and dietary fiber intakes were observed (and in carbohydrate intakes expressed as a percentage of energy) (Supplementary Table S10). Vegans, compared to lacto-ovo vegetarians, had higher intakes of carbohydrates and dietary fiber, and lower intakes of fats (particularly saturated fats) and cholesterol, as well as a lower n-6/n-3 fatty acid ratio.

In the vegetarian group, the intake of Fe, Cu, K, and Mg was higher, while the intake of I, Se, Zn, Na, and P was lower, with no difference in the intake of added salt and Ca (Table 7). Regarding vitamins, the intake of β-carotene and vitamins B9 and C was higher, the intake of retinol and vitamins B2, B3, B12, and D was lower, and the intake of vitamins A (expressed as RE and RAE), B1, B5, B6, and E was no different. Additionally, the intake of polyphenols was higher, while the total DII and E-DII scores were significantly lower among vegetarians. All these significant differences in micronutrient intakes and dietary inflammatory scores remained significant even after corrections for sex, age, BMI, smoking status, and physical activity level. The FDR correction also did not reduce the significance of these differences. The results were similar in supplementary sensitivity analyses when intake was expressed per 1000 kcal, except for Zn, where significance in the difference was lost, and for vitamin B1 and vitamin A in RE, where higher intake per 1000 kcal was noted in vegetarians (Supplementary Table S11). The FDR correction did not influence also these results.

Differences in micronutrient intakes were more pronounced when compared to vegans, who also had a lower intake of Ca, whereas they were less evident when compared to lacto-ovo vegetarians, among whom differences in minerals were observed only for Cu, Mg, Na, and Se (Supplementary Table S12). Lacto-ovo vegetarians also had a lower intake of vitamin B5 and B6 compared to omnivores, apart from retinol, B3, B9, B12, C, D, and polyphenols, with the DII and E-DII scores being, respectively, borderline (*p* = 0.055) and significantly (*p* = 0.013) lower. Vegans, compared to lacto-ovo vegetarians, had higher intakes of B5 and, as expected, lower intakes of Ca, I, retinol, and B12, and lower E-DII.

The reported use of dietary supplements was generally low in both groups. Based on the FPQ data, only a small proportion of participants reported relatively frequent use of specific supplements (Supplementary Table S13). Among vegetarians, the most frequently reported supplements (≥1 time per week) were vitamin B12 (20% of participants reported), Mg, algae-based dietary supplements, and vitamins C and D (~10–14% of participants each), whereas among omnivores, the most frequently reported were vitamins C and D (~23–24% of participants), Zn, Se, Mg, and n-3 fatty acids (~10–14% of participants each). Statistically significant differences between dietary groups were observed for vitamins B12, D, C, Zn, Se, and n-3 fatty acids, being all notably higher in omnivores, except for B12, which was higher in vegetarians; however, after FDR correction for supplement family, only the difference in vitamin B12 remained significant. In contrast, the 24 h dietary recalls captured substantially fewer supplement users than the FPQ. The number of those who reported the use of supplements during 24 h recalls with estimated doses are presented in Supplementary Table S14. Overall, based on the small number of participants reporting supplement use in the 24 h recalls, supplements likely made only a minor contribution to the estimated micronutrient intakes in both groups.

### 3.6. Correlations of Cytokine Profiles with CRP Levels, Leukocyte Counts, and Dietary Intakes

We performed exploratory non-parametric correlation analyses between cytokine profiles, standard biochemistry parameters, CRP levels, leukocyte counts, anthropometrics, and dietary intakes (energy, macronutrients, micronutrients, food groups, and DII score), without and with adjustments for sex, age, BMI, smoking status, and physical activity level.

Cytokine levels were not correlated with specific leucocyte counts. Still, we found positive correlations of CRP with IL-18, IL-6, IL-8, and MCP-1 levels (in unadjusted analyses, Spearman’s correlation coefficients, respectively: *r_s_* = 0.271, *p* = 0.001, *r_s_* = 0.192, *p* = 0.020, *r_s_* = 0.172, *p* = 0.039, and *r_s_* = 0.137, *p* = 0.037). After adjustment for sex, age, BMI, physical activity level, and smoking, only correlations of CRP with IL-18 and IL-6 remained (adjusted Spearman’s partial correlation coefficients, respectively: *r_s_* = 0.232, *p* = 0.006 and *r*_s_ = 0.195, *p* = 0.021). However, after FDR correction for 13 cytokines, only unadjusted associations with IL-18 remained.

In unadjusted analyses, no significant correlations were observed between cytokine profiles and CRP with anthropometric or sociodemographic factors, except for sex, with higher IFN-γ levels in women (Mann–Whitney, *p* = 0.040), and age being positively correlated with MCP-1 (Spearman’s *r_s_* = 0.232, *p* = 0.004), IL-10 (*r_s_* = 0.171, *p* = 0.036), and CRP (*r_s_* = 0.178, *p* = 0.032). However, when we adjusted for sex, age, physical activity, and smoking, IL-6 positively correlated with body weight, BMI, and hip circumference (*r_s_* = 0.178, *p* = 0.031, *r_s_* = 0.163, *p* = 0.049, and *r_s_* = 0.248, *p* = 0.003, respectively), TNF-α with WHtR (*r_s_* = 0.163, *p* = 0.048), and IL-8 with waist circumference and WHtR (*r_s_* = 0.178, *p* = 0.032 and *r_s_* = 0.228, *p* = 0.006, respectively). Additionally, hip circumference also correlated with IL-12p70, IL-17A, IL-23, and IL-33 (*r_s_* = 0.239, *p* = 0.004, *r_s_* = 0.184, *p* = 0.026, *r_s_* = 0.183, *p* = 0.027, and *r_s_* = 0.166, *p* = 0.044, respectively). Nevertheless, after the FDR correction for 13 cytokines and these six anthropometric variables (78 tests) was applied, no associations remained significant.

Similarly, few associations were found with standard biochemical parameters. Positive correlations were observed with total cholesterol (IFN-α2, IL-12p70, IL-17A, IL-33), LDL-cholesterol (IL-1β, IFN-α2, IL-12p70, IL-17A, IL-33), urea (IL-10, IL-17A), creatinine (IL-23), and total proteins (IL-1β, IL-6, IL-8), while negative correlations were found with total bilirubin (IFN-γ, TNF-α, IL-6, IL-17A, IL-18) and uric acid (IFN-α2). However, those correlations were weak (unadjusted analyses: Spearman’s *r_s_*, ranging from 0.165 to 0.253 for positive correlations and from −0.162 to −0.246 for negative correlations, *p* = 0.002–0.047; adjusted analyses: *r_s_* from 0.166 to 0.246 for positive correlations and from −0.177 to −0.221 for negative correlations, *p* = 0.003–0.046). Additionally, after FDR correction for 13 cytokines and 15 biochemical variables (195 tests), no associations remained.

Similarly, we observed no significant or meaningful correlations of cytokine/chemokine levels with dietary intakes and DII/E-DII scores, apart from a few isolated associations (e.g., negative correlations of various cytokines with fruit, nuts/seeds, dietary fiber, and n-3 intakes; positive correlations with meat, eggs, added fats and oils, saturated fat, the n-6/n-3 ratio, cholesterol, protein, and total energy intakes). However, those correlations were also weak (unadjusted analyses: Spearman’s *r_s_*, ranging from 0.167 to 0.236 for positive and from −0.167 to −0.242 for negative correlations, *p* = 0.003–0.043; adjusted analyses: Spearman’s *r_s_* from 0.164 to 0.229 for positive and from −0.166 to −0.229 for negative correlations, *p* = 0.004–0.049). The greatest number of correlations was noted with cholesterol and saturated fat intakes. A similar pattern was observed for DII score: it was only weakly associated with IL-8 and IL-17A (respectively, unadjusted analyses: *r_s_* = 0.187, *p* = 0.023 and *r_s_* = 0.182, *p* = 0.027; adjusted analyses: *r_s_* = 0.171, *p* = 0.042 and *r_s_* = 0.209, *p* = 0.012). Those associations were slightly more pronounced when adjusted for energy in E-DII scores (respectively, unadjusted analyses: *r_s_* = 0.214, *p* = 0.009 and *r_s_* = 0.216, *p* = 0.008; adjusted analyses: *r_s_* = 0.193, *p* = 0.021 and *r_s_* = 0.273, *p* < 0.001). However, all these correlations lost significance after the FDR correction for multiple testing (e.g., 13 cytokines x 26 dietary variables = 338 multiple correlations). Nevertheless, when we performed the same analyses using our reduced dataset after exclusion of participants with statistical upper-tail outlier/extreme cytokine values, as sensitivity analyses, the strength and number of significant associations increased, with many correlations remaining significant even after FDR correction for the 13 cytokines and 26 dietary variables included in the analyses. In the unadjusted analyses (Supplementary Table S15), 61 of 338 correlations remained significant after FDR correction. The highest number of significant correlations was observed with meat, saturated fats, cholesterol (all positive associations), and with fiber intake (negative associations). No significant correlations were observed for grain products, sweets, beverages, polyunsaturated, n-3, and trans-fatty acids, or DII. In the adjusted analyses (Supplementary Table S16), 102 of 338 correlations remained significant after FDR correction. The highest number of significant correlations was observed again with meat, saturated fats, cholesterol, and fiber intakes. No significant correlations were observed only for grain products, beverages, n-3 and trans-fatty acids. Thus, after adjustment for BMI, physical activity level, smoking, sex, and age, a larger number of dietary–cytokine associations remained significant following FDR correction in the outlier-excluded dataset.

Importantly, many dietary factors were highly intercorrelated (e.g., *p* < 0.0001 for correlations between various macronutrients, micronutrients, and dietary groups), indicating substantial multicollinearity, making it difficult to distinguish the individual nutrient associations with cytokine/chemokine levels and increasing the potential for confounding. For example, protein intake was positively correlated with total energy, fat (including all fat subsets), dietary cholesterol, Ca, Fe, I, Na, P, Se, Zn, retinol, vitamin B1, B2, B3, B5, B6, B12, D, and E intakes, and negatively with fiber, vitamin C, β-carotene, and polyphenol intakes. Similarly, meat intake correlated negatively with dietary fiber, polyphenols, Mg, K, Fe, Cu, β-carotene, folate, and vitamin C intakes, and positively with energy intake and all other analyzed macro- and micronutrients, except n-3 fats, alcohol, Ca, vitamins A, B1, B6, and E, with which no correlation was shown. This complicates interpretation and limits the ability to isolate independent effects. Correlations of food groups with specific nutrients are presented in Supplementary Table S17.

### 3.7. Linear Regression for Vegetarian Dietary Pattern as a Predictor of Serum Cytokine Levels

We performed both forced-entry and stepwise models in linear regression analyses with logarithmically transformed cytokine levels as dependent variables, and dietary patterns as predictors, classified as vegetarian vs. omnivorous. Other covariates in the regression model included sex, age, BMI, smoking status, and physical activity level.

The forced-entry models, provided in Table 8 (abbreviated, due to space limitations) and Supplementary Table S18 (full model description), were used to assess the association between dietary pattern and cytokine/chemokine concentrations after adjustment for the prespecified covariates, and were considered as main models. The stepwise models (Supplementary Table S19) were considered exploratory analyses to identify predictors retained by the data-driven selection procedure.

In the forced-entry regression models, a vegetarian dietary pattern was significantly inversely associated with several cytokine levels, including TNF-α, IL-6, IL-8, IL-10, IFN-γ, IFN-α2, IL-12p70, and IL-17A (Table 8 and Supplementary Table S18). Dietary pattern was not significantly associated with IL-1β, MCP-1, IL-18, IL-23, or IL-33. Other covariates were not significantly associated in the models, except for TNF-α and MCP-1, where female sex and age were associated with higher cytokine values, respectively. Because dietary pattern was the primary predictor of interest, while the other variables were included as adjustment covariates rather than as primary hypotheses, FDR correction was applied across the 13 cytokine/chemokine outcomes, rather than across all 78 predictor–outcome combinations. After this correction, in the forced-entry model, the associations of dietary pattern with IFN-γ, IL-8, IL-12p70, and IL-17A remained statistically significant.

In the stepwise regression models (Supplementary Table S19), a vegetarian dietary pattern was significantly inversely associated with the same cytokines as in the forced-entry models (TNF-α, IL-6, IL-8, IL-10, IFN-γ, IFN-α2, IL-12p70, and IL-17A). Other predictors were not significant in the models, except for IL-1β and IFN-γ, where female sex was associated with higher values, and for MCP-1 and IL-10, where age was a positive predictor. For other cytokines (IL-18, IL-23, and IL-33), none of the predictors was significant. The FDR correction did not eliminate the significance of dietary patterns in the stepwise models, due to the lower *p*-values obtained compared to the forced-entry models.

The adjusted *R*^2^ values for the diet-associated models were quite low, ranging from 0.018 to 0.092 in the forced-entry model, and 0.027 to 0.092 in the stepwise model; thus, the models explained only a modest proportion of between-person variation (1.8–9.2% and 2.7–9.2%, respectively). Since the entered covariates were not, in general, significantly associated with cytokine concentrations in our study population, the forced-entry models showed lower adjusted *R*^2^ values and overall model significance, reflecting the inclusion of non-associated predictors in most of the cases.

Visual inspection of the normal P–P plots indicated no substantial departures from normality of the residuals, and the residual-versus-predicted-value plots did not indicate substantial heteroscedasticity. The maximum standardized residuals and studentized deleted residuals were below commonly used thresholds for potentially extreme observations (|3|). Across the forced-entry regression models, maximum leverage values were approximately 0.1 (0.096–0.097), while Cook’s distances remained up to 0.117, indicating no evidence of substantial influence of individual observations on the regression estimates. Across the stepwise regression models, maximum leverage values were approximately 0.010 (0.007 for all, except 0.014 for IFN-γ and 0.034 for IL-10), while Cook’s distances remained low (maximal up to 0.061). Overall, the diagnostic assessment did not identify major violations of the assumptions underlying the linear regression models, particularly for those that identified vegetarian dietary pattern as a significant predictor.

Only for MCP-1, the stepwise model was characterized by two observations with large negative standardized residuals (−3.248 and −3.642), and maximal Cook’s distance was 0.239, suggesting some influence of individual observations on model fit. However, stepwise regression selected age as the only significant predictor for MCP-1, whereas dietary group was not retained in the model. The same was true for IL-18 in the forced-entry regression model, where two observations had large standardized residuals, above |3|, and maximal Cook’s distance was 0.080. However, this model did not find any significant predictors.

Therefore, the models that identified dietary pattern as a significant predictor of certain cytokines (both forced-entry and stepwise) were not significantly influenced by individual observations and satisfied assumptions for residual distribution, heteroscedasticity, and influential observations in case all subjects were retained in the dataset. In all forced-entry models, VIFs were up to 1.560.

In additional sensitivity analyses, based on the observed associations of bilirubin and uric acid with cytokine levels, including bilirubin as an additional covariate did not change the effect of the vegetarian diet in both models, despite recognizing bilirubin as an additional significant negative predictor for several cytokines. These included IFN-γ, TNF-α, IL-6, IL-12p70, IL-17A, and IL-18 in the forced-entry models, with IL-1β additionally identified in the stepwise models. The addition of bilirubin slightly increased the significance of the models, confirming the independent effects of bilirubin and diet. Adding uric acid as a covariate also did not change the effect of the diet. The independent effect of uric acid was found only in the stepwise models for IL-1β, IFN-α2, and IFN-γ, but not in the forced-entry models.

## 4. Discussion

The present study addressed the issue of differences in serum cytokine status between apparently healthy normal-weight subjects on an omnivorous diet or a vegetarian (lacto-ovo vegetarian and vegan) diet, while matching for potential confounders, including sex, age, BMI, smoking, and physical activity level. We observed in vegetarian subjects significantly lower levels of several cytokines, including TNF-α, IL-6, IL-10, IL-12p70, IFN-γ, IFN-α2, IL-17A, and IL-8, while we failed to demonstrate significant differences in IL-1β, IL-18, IL-33, IL-23, or MCP-1 (although we found marginally lower IL-23). The differences found remained significant even after adjustment for potential confounding factors (including BMI), and regression analyses further confirmed that a vegetarian dietary pattern was significantly associated with lower cytokine/chemokine levels. However, further correction for multiple testing kept only differences and associations with IFN-γ, IL-8, IL-12p70, and IL-17A significant. Additionally, individuals following a vegetarian diet had lower leukocyte and lymphocyte counts, whereas serum CRP levels did not differ between groups. Overall, these findings suggest that a vegetarian diet may be associated with immune status, beyond its effects on body weight.

Vegetarian diet differed significantly in terms of intakes of certain food groups, which translated into differences in various macro- and micronutrient intakes and dietary inflammation scores. These differences were highly significant and remained after correction for multiple testing. In addition, several isolated dietary factors have shown significant associations with cytokine levels. Nevertheless, these exploratory associations were weak and were lost after the multiple testing corrections. Moreover, there was a substantial multicollinearity between individual dietary factors, limiting the ability to identify their independent contributions, as the observed associations may partly reflect the influence of other correlated dietary factors.

Compared with previous studies comparing vegetarians/vegans and omnivores, which in general failed to detect differences in baseline circulating cytokine concentrations [25,26,27,28,29,30,31,32,33,34,43,89], our study identified a greater number of significant differences. This discrepancy may be attributable not only to differences in study populations, sample sizes, and analytical methods, but also to our rigorous control of potential confounding factors during participant selection. Furthermore, we performed additional statistical adjustments for residual confounding, thereby strengthening the robustness of our findings and increasing confidence that the observed differences were attributable to dietary pattern rather than other participant characteristics.

In parallel with observed differences in cytokine levels, the vegetarian diet differed significantly from the omnivorous diet in the intake of various food groups, which was reflected in marked differences in the consumption of nearly all macro- and micronutrients. These included marked differences in intakes of proteins, carbohydrates, fats (particularly saturated, monounsaturated, n-6 polyunsaturated, and trans- fats, as well as n-6/n-3 fatty acid ratio), dietary cholesterol and fiber, polyphenols, vitamins (higher: β-carotene, B9, and C; lower: retinol, B2, B3, B12, and D), and minerals (higher Fe, Cu, K, and Mg; lower Na, P, I, Se, and Zn), resulting in marked differences in total daily energy intakes and inflammatory DII and E-DII scores. When we adjusted our analyses for possible confounding factors, including BMI and physical activity, the differences persisted. The correction for total energy intake also did not generally influence these findings. Differences were most pronounced in comparison to the vegan diet, but were also found in comparison to the lacto-ovo vegetarian diet, although to a lesser extent. In agreement with the literature [16,90,91,92,93,94,95,96,97], the intake of eggs and milk compensated for the intake of several minerals (Ca, Se, Zn, and I) and vitamins (B2 and, only partly, B12) in lacto-ovo vegetarians, compared to vegans. Importantly, the estimated intake of certain minerals should also be evaluated in the context of their lower bioavailability from plant-based dietary sources (e.g., Fe, Zn, and Ca) [13,16]. Our findings on dietary intakes are in accordance with the existing literature [16,20,90,91,92,93,94,95,96,97,98,99,100,101], except perhaps for n-6 fat intakes and the n-6/n-3 ratio, which some researchers found to be higher in vegetarians [97,102,103,104]. The n-3 intake was low in both vegetarians and omnivores in our study, due to low intake of fish generally in the Serbian population [15,105,106]. The main source of n-3 in both diets is α-linolenic acid (ALA), which has only a limited internal conversion to more biologically potent eicosapentaenoic acid (EPA) and docosahexaenoic acid (DHA) [104].

Interestingly, the marked difference in total energy intake between the groups was not accompanied by notable differences in body weight or BMI, and persisted when expressed per kg body mass and after adjustment for sex, age, smoking, and physical activity level. As the groups were comparable with respect to these major factors that could influence energy requirements and body weight, the reason for the higher reported energy intake among omnivores remains unclear. Although the higher protein intake in omnivores could potentially contribute through its greater thermic effect of food and effects on fat-free mass [107,108,109,110,111,112,113,114], evidence for differences in resting energy expenditure and diet-induced thermogenesis between vegetarians and omnivores remains inconclusive, with some studies reporting opposite findings [33,115,116]. Therefore, this potential explanation should be considered cautiously.

According to the existing literature, multiple interconnected molecular and metabolic pathways may explain how dietary factors associated with vegetarian and omnivorous dietary patterns influence cytokine production, secretion, and circulating concentrations. These may involve the activation or modulation of several interconnected immunological signaling pathways, including (1) the nuclear factor kappa B (NF-κB) pathway; (2) the NOD-, LRR-, and pyrin domain-containing protein 3 (NLRP3) inflammasome pathway; (3) the mitogen-activated protein kinase (MAPK)/activator protein 1 (AP-1) pathway; (4) the mammalian target of rapamycin (mTOR) pathway; (5) the phosphatidylinositol-3 kinase (PI3K)–protein kinase B (PKB/Akt) pathway; (6) the nuclear factor erythroid 2-related factor 2 (Nrf2) pathway; (7) the eicosanoid and endocannabinoid signaling pathways; and (8) the gut dysbiosis and LPS pathways. These and other interacting pathways regulate the production, secretion, and activity of numerous cytokines and may therefore contribute to the lower circulating cytokine levels observed in plant-based dietary patterns. A brief overview of these mechanistic pathways and the known dietary factors that influence them is provided in Supplementary Material, Section S4.1, to facilitate interpretation of our findings.

As noted above, our study identified some significant associations between cytokines and several dietary factors. In particular, we observed negative associations with intakes of fruit, nuts/seeds, dietary fiber, and n-3 fatty acids, and positive associations with intakes of meat, eggs, added fats and oils, the n-6/n-3 fatty acid ratio, saturated fatty acids, cholesterol, protein, total energy intakes, and dietary inflammation indexes. Overall, these patterns are broadly consistent with associations reported in the existing literature and with the potential biological mechanisms discussed in the Supplementary Materials, Section S4.1. Nevertheless, all of these associations were weak and did not remain significant after correction for multiple testing. The generally weak correlations observed in this study may reflect not only genuinely weak associations but also a combination of other factors, including day-to-day variation in dietary intake, the lack of temporal alignment between the dietary recalls and biological sampling, dietary reporting and intake-calculation errors, pre-analytical and analytical limitations, biological variability in cytokine levels, and the relatively small sample size. Importantly, after exclusion of participants with upper-tail cytokine outlier/extreme values, substantially more associations with these dietary factors became significant and remained after correction for multiple testing, despite the considerable reduction in sample size and statistical power. For example, in the adjusted analyses, even after the FDR correction, multiple cytokines remained associated with cholesterol, saturated fat, meat, dietary fiber, n-6 fatty acids, nuts/seeds, added fats and oils, protein, and fruit and vegetables, as well as other dietary factors. No significant associations were observed only for grain products, non-alcoholic and alcoholic beverages, n-3, and trans-fatty acids. This sensitivity analysis therefore indicates that the observed associations were not driven solely by the extreme cytokine values, although the findings should be interpreted cautiously given the reduced sample size and high multicollinearity.

The main potential mechanistic aspects and possible pathways underlying the associations of cytokine levels with these dietary factors are discussed in the Supplementary Materials, Section S4.2, to keep the main manuscript concise and focused. In this Supplementary Section, we discuss our findings in relation to dietary fats, cholesterol, protein, fiber, polyphenols, and micronutrients that differed significantly between the two groups, as well as their potential relationships with food groups that differed in intake between the two dietary patterns and were associated with cytokine levels in the correlation analyses. For some of these factors, such as fats, cholesterol, fiber, and polyphenols, mechanistic studies provide substantial supporting evidence, whereas for others, such as proteins, the available evidence is more limited or conflicting. Importantly, this section considers these mechanisms as potential explanations for the observed differences in and associations with cytokine levels rather than as mechanisms demonstrated by the present study. Their testing and confirmation would require targeted mechanistic and longitudinal or intervention studies, which are beyond the scope of the present cross-sectional study. Moreover, given the substantial multicollinearity among dietary parameters, it is not possible to determine the independent contribution of any specific dietary factor or to identify a primary dietary factor underlying the observed associations. For example, protein intake was highly correlated with the intake of meat, milk, eggs, cholesterol, all types of fat (but particularly saturated fat), and numerous micronutrients (e.g., vitamin D, B12, retinol, B2, B3, B6, Se, I, Zn, Ca, Na, and P) primarily associated with animal-derived foods. Therefore, the independent associations of proteins could not be distinguished in this cross-sectional study, and the observed associations may be partly attributable to confounding by other correlated dietary factors.

The biomarkers analyzed in this study represent different components of innate and adaptive immune-inflammatory signaling pathways and include cytokines and chemokines traditionally classified as predominantly pro-inflammatory (TNF-α, IL-1β, IL-18, IL-12, IFN-γ, INF-α, IL-23, IL-17A, IL-8, and MCP-1), predominantly anti-inflammatory ([IL-10), or context-dependent (IL-6 and IL-33) [117,118,119,120,121,122]. However, such classification is increasingly considered an oversimplification, as the biological effects of most cytokines depend on multiple factors, including cellular source, tissue microenvironment, receptor distribution, signaling pathway, concentration, timing, and physiological or pathological context [121,123,124,125]. Many cytokines may therefore exert both pro- and anti-inflammatory effects under different conditions [119,120,123,124]. Detailed information regarding the biological role of each analyzed cytokine/chemokine, including their cellular sources, activation pathways, signaling mechanisms, and secretion kinetics, is provided in the Supplementary Materials, Section S4.3.

The observed reductions across both pro-inflammatory (e.g., TNF-α, IL-6, IL-17A, IFN-γ) and regulatory cytokines (e.g., IL-10) suggest that vegetarian dietary patterns may modulate multiple interconnected signaling pathways and more broadly influence the immune tone. Notably, numerous serum cytokine and chemokine concentrations, both pro-inflammatory and regulatory, were highly positively intercorrelated in our study, which likely reflects the coordinated nature of immune and inflammatory signaling networks. Cytokines rarely act independently; rather, they are produced through interconnected regulatory cascades in which activation of upstream pro-inflammatory pathways, such as NF-κB, MAPK/AP-1, mTOR, and NLRP3 inflammasome signaling, could simultaneously induce, while upstream anti-inflammatory pathways, such as Nrf2, aryl hydrocarbon receptor (AhR), adenosine monophosphate-activated protein kinase (AMPK), and G protein-coupled receptors (GPRs) signaling, could simultaneously block, expression of multiple cytokines and chemokines [126]. In addition, many cytokines regulate each other through autocrine and paracrine feedback mechanisms [122] (see Supplementary Materials, Section S4.2). For example, IL-1β and TNF-α stimulate each other’s production and the production of IL-6 and chemokines IL-8 and MCP-1 [118,127], whereas chemokines IL-8 and MCP-1 may further amplify leukocyte recruitment and cytokine secretion [128]. Likewise, IL-23, IL-6, and IL-1β augment production of IL-17A [69,129], IL-6 and IL-1β stimulate production of IL-10 [130], while IL-6 (and less IL-1β and TNF-α) stimulates production of CRP [130]. Similarly, IL-12, IL-18, and IL-1β augment production of IFN-γ [131,132], while IFN-γ, in turn, augments production of multiple cytokines (e.g., TNF-α, IL-1β, IL-6, IL-12, and IL-18), chemokines (MCP-1, CXCL9, CXCL10, and CXCL11), and inducible nitric oxide synthase (iNOS), thereby amplifying immune responses [117,131,133,134]. The observed correlations may therefore reflect shared upstream regulatory mechanisms, coordinated immune activation, cytokine networking, and positive and negative feedback loops [135]. Furthermore, circulating cytokine levels in healthy individuals are generally low and influenced by several biological determinants, including genetic background, sex, age, hormonal status, level and type of adiposity, gut microbiota, and circadian regulation [83,135,136,137,138]. Numerous cytokine-related genes contain functional polymorphisms that may influence cytokine production and responsiveness, while X chromosome-linked genes and sex hormones additionally modulate innate and adaptive immune responses [86,135,139]. Such multiple intrinsic determinants may contribute both to the substantial inter-individual variability and to the coordinated covariance observed among cytokines and chemokines. In addition, external factors, including dietary factors, smoking, and physical activity, can rapidly affect oxidative stress, metabolic status, intestinal permeability, and adipose tissue activity, thereby temporarily modulating multiple cytokine productions [135,136,137,138]. Nevertheless, substantial multicollinearity among inflammatory markers and dietary variables may complicate the identification of independent associations and causal pathways.

Interestingly, in our study, we did not observe a significant difference between the two dietary groups for several circulating cytokines, including IL-1β, IL-18, IL-33, IL-23, and MCP-1, despite observed differences in dietary factors known to modulate their inflammatory signaling pathways (Supplementary Materials, Section S4.1). In sensitivity analyses, including the values below LLOQ, the results were the same. All of them, except MCP-1, had lower levels in vegetarians, and for IL-23, that was on the borderline level of significance. Additionally, MCP-1 in particular, and IL-1β and IL-18 to a lesser extent, also had weaker correlations with other measured cytokines.

The absence of significant differences in circulating levels of the mentioned cytokines may be explained by several factors. One of the reasons could be related to increased inter-individual variability for those cytokines in our study, which may have reduced the statistical power to detect significant differences. The high relative and absolute variability was observed for all of them (apart from the high variability also observed for IL-8, and, to a lesser extent, TNF-α and IL-6). There could be expected physiological fluctuations leading to low repeatability of the measurements. Intra-individual variations among circulating cytokine concentrations could exist due to seasonal, hormonal, or circadian physiological variability, apart from the influence of lifestyle factors, including smoking, physical activity, or recent diet [86,135,137,138,140,141,142,143,144,145]. Repeated plasma measurements in some studies have demonstrated good within-person reliability for TNF-α, IL-6, IL-8, IL-10, IL-17A, and IFN-γ [144,146], but poor reliability for IL-1β, IL-12, and MCP-1 [137,144]. Therefore, serum measurements at a single time point may not adequately capture transient, short-term releases of cytokines with rapid responses and/or short half-lives (e.g., IL-1β, IL-18, and IL-33) [139,147,148,149,150,151]. However, their effect on the stimulation of downstream mediators (i.e., other cytokines, including IL-6, TNF-α, IL-10, IFN-γ, and IL-17) can be prolonged, resulting in an increased tone of their downstream effectors. Probably for such cytokines that are more “upstream” and with more acute, pulsatile secretion, multiple blood samples or the stimulation tests ex vivo (e.g., with LPS) or in vivo with a meal challenge would be more suitable to detect the meaningful differences [143].

The absence of differences in IL-1β, IL-18, IL-33, IL-23, and MCP-1 should also be interpreted in the context of their distinct biological regulation and predominantly local modes of action. Many of these cytokines (e.g., IL-1β, IL-18, IL-33, and IL-23) function predominantly in a local paracrine or autocrine manner, resulting in low systemic concentrations under homeostatic fasting conditions [152]. For example, inflammasome-derived cytokines such as IL-1β and IL-18 are largely compartmentalized within tissue-resident immune and other cells, with only limited spillover into the circulation in the absence of overt inflammation [118,132,143]. Their secretion is tightly regulated and typically occurs in a transient, pulsatile manner following inflammasome activation, resulting in marked temporal fluctuations after acute stimuli, including acute dietary exposures, fasting, and refeeding [118,132,143,145,153]. For example, IL-1β is transiently present in the circulation following in vivo stimulation, with reported half-lives of approximately 1–3 h [150,154,155,156]. Consequently, circulating concentrations may vary considerably depending on the timing of blood collection relative to stimulation [157]. However, the absence of differences cannot be attributed to the relatively short circulation half-lives of those cytokines, as several cytokines for which significant between-group differences were observed (e.g., TNF-α, IFN-γ, IL-6, IL-8, and IL-10) also exhibit similarly short, or even shorter, half-lives [154,156,157,158,159,160]. Of note, human studies have reported variable changes in circulating IL-18 levels following in vivo stimulation; thus, its circulating kinetics are less characterized [139,161,162]. Moreover, circulating IL-18 activity is strongly regulated by IL-18-binding protein (IL-18BP), which neutralizes free IL-18 and prolongs its persistence in circulation [118,139,163,164,165]. Unfortunately, we did not measure IL-18BP. Similarly, IL-1β activity is negatively regulated in circulation by its soluble IL-1 receptor (sIL-1R) and IL-1 receptor antagonist (IL-1RA) [150]. Finally, previous studies comparing vegetarians and omnivores have also reported no differences in IL-1β or IL-18, while some have reported higher IL-1β in vegetarians or positive associations of IL-18 with a vegan diet duration [26,27,33,166,167]. Meal stimulation studies in humans also reported inconsistent results regarding postprandial responses of these cytokines, with the effect being influenced largely by the meal type [143,148,168,169,170,171,172,173,174,175,176,177].

Regarding MCP-1, its levels are influenced by tissue-specific chemotactic gradients, and circulating concentrations may not adequately reflect local monocyte recruitment [152]. After LPS stimulation, MCP-1 increases after 1.5–2 h and remains elevated for 6–12 h or even longer [154,160,178,179]. Therefore, MCP-1 has more prolonged kinetics after stimulation. However, meal stimulation studies generally did not find increases in serum levels, with only rare studies reporting modest elevations that peaked at 6 h [143,173,180,181]. Of note, other authors have also not found differences in MCP-1 between vegetarians and omnivores [26,33]. Furthermore, obesity can primarily influence MCP-1 levels, and in this study, we had a narrow BMI range and normal-weight subjects.

IL-33, another member of the IL-1 family, functions as an “alarmin” released upon cell damage (i.e., trauma or high levels of physical activity) or the presence of allergens [182,183,184]. In our study, subjects with allergic reactions were excluded, and all participants were asked to refrain from physical activity before the study; therefore, the IL-33 circulating levels should not be increased. IL-33 is released immediately upon cell damage, acts rapidly locally, and has a very short half-life of ~1.5 h due to oxidation (forming a biologically inactive oxidized form) and proteolytic inactivation [149,182,185,186,187,188]. Additionally, binding to its soluble receptor sST2 limits its activity [184]. Unfortunately, we did not measure the sST2 levels, which show a delayed and much more prolonged increase after IL-33 secretion, even for several days [149,184,186]. In cell culture models, after LPS stimulation, IL-33 secretion by viable cells was not detected [189]. Similarly, after LPS stimulation in vivo in humans, serum IL-33 levels were not increased, but its sST2 receptor showed delayed and prolonged elevations, even for 24 h [184]. In addition, the single available study investigating postprandial responses did not demonstrate changes in circulating IL-33 levels after a fatty meal [190]. Therefore, dietary modulation of IL-33 is less likely to be reflected in circulating concentrations.

The heterodimeric cytokine IL-23, a member of the IL-12 family composed of a unique p19 subunit and the p40 subunit shared with IL-12, also primarily functions within local tissue microenvironments, with a predominantly local and paracrine mode of action to maintain and expand Th17 cells [118,129]. Consequently, fasting serum IL-23 concentrations in healthy individuals are typically low and may provide limited insight into overall IL-23/Th17 axis activity [191]. Nevertheless, downstream effects, particularly sustained IL-17 production, may persist due to Th17 cell expansion, with IL-17 secretion further being enhanced by IL-1β and IL-6 [129]. In cell culture models, LPS stimulation induced detectable IL-23 expression approximately 2–4 h after stimulation, remaining elevated for at least 16 h [192]; however, data on circulating IL-23 kinetics following physiological challenges in humans remain scarce. In rare human meal-challenge studies that measured IL-23, circulating IL-23 started to increase 2 h after meal ingestion and remained elevated after 4 h, but the subsequent time course and duration of elevation were not examined [193].

Curiously, we observed substantially higher circulating concentrations of some analyzed cytokines compared with values commonly reported in the literature [144], particularly for members of the IL-1 family (e.g., IL-1β, IL-18, and IL-33) and the chemokine family (IL-8, less MCP-1). Some of these cytokines, especially IL-1β and IL-33, are frequently reported to be present at very low or even undetectable concentrations in healthy individuals [136,137,144]. Nevertheless, both in our study and studies using the same assay platform [194], as well as in the LEGENDplex™ manufacturer’s reference data [61], substantially higher concentration ranges were shown, without a clear methodological explanation provided by the manufacturer. Compared with conventional ELISA and other methods, multiplex bead-based immuno-fluorescence assays may yield systematically higher concentration estimates due to methodological differences in calibration procedures, curve-fitting and data-processing algorithms, fluorescence signal amplification, background fluorescence, matrix effects, antibody affinity and specificity, possible cross-reactivity between structurally related analytes, and differential detection of free, bound, precursor, or partially degraded (e.g., biologically inactive oxidized forms) cytokine forms [56,139,149,152,185,195,196]. For example, the LEGENDplex™ assay quantifies immunoreactive IL-1β and IL-18. Because the manufacturer does not report antibody reactivity toward the precursor forms (pro-IL-1β and pro-IL-18) or bound cytokines [61], the measured concentrations cannot be assumed to represent exclusively the mature or unbound, biologically active cytokines. However, the serum precursor levels of IL-1β and IL-18 are generally low. Nevertheless, their active mature forms are often suppressed by binding to circulating antagonizing factors (IL-1RA, sIL-1R, IL-18BP) [118,132,139,150,163]. IL-33 is also suppressed by binding to its sST2 soluble receptor in circulation, which has more prolonged circulating half-lives [186,187]. In addition, serum measurements may be further influenced by ex vivo cytokine release during coagulation and sample processing, particularly for IL-1β, IL-8, and MCP-1 [56,140,152,197,198,199,200,201]. Importantly, potential cross-reactivity among structurally related cytokines (e.g., the IL-1 family, including IL-1β, IL-1α, IL-18, and IL-33, or the IL-12 family, including IL-23 and IL-12) and interference from soluble receptors or binding proteins may additionally contribute to overestimation in multiplex platforms such as LEGENDplex™ [152,202,203]. However, the LEGENDplex™ manufacturer demonstrated negligible cross-reactivity among cytokines in the panel (except for IFN-α6/IFN-α2 cross-reactivity) but did not report antibody specificity for precursor, bound, or other molecular forms of the measured cytokines [61]. For all those reasons, absolute cytokine concentrations reported across studies should be interpreted cautiously, as considerable variability exists between analytical platforms, particularly for low-abundance cytokines or those with potential cross-reactivity [202,203,204]. Because all samples in the present study were analyzed using the same assay platform, within the same sample preparation, storage, analytical conditions, processing procedures, and analytical run and calibration, the relative differences between dietary groups may be more convincing, particularly for those with low potential cross-reactivity and serum sample processing influences.

Therefore, the lack of significant differences in MCP-1, IL-1β, IL-18, IL-33, and IL-23 between dietary groups may also reflect pre-analytical and analytical limitations associated with their measurement, and not only a true absence of biological differences. This is particularly relevant for these cytokines because their circulating concentrations are low, tightly regulated, or highly sensitive to sample handling and assay characteristics. Potential sources of variability include cytokine release during serum clotting from platelets, monocytes, neutrophils, and erythrocyte surfaces, as well as analytical interferences related to matrix effects, cytokine-binding proteins, detection of precursor or degraded molecular forms, and antibody specificity or cross-reactivity [56,140,152,197,198,199,200,201,202,203,204,205]. These factors may substantially influence measured concentrations, especially for low-abundance and predominantly tissue-localized cytokines (such as IL-1β, IL-18, and IL-33), or those that could be released during coagulation (IL-1β, IL-18, MCP-1, and IL-8), thereby potentially masking relatively small differences associated with dietary patterns. The unexpectedly high absolute concentrations and high variability observed in our study for these cytokines further advocate that methodological factors may have reduced the sensitivity to detect subtle biological differences.

Interestingly, excluding participants with upper-tail outliers/extreme cytokine values in our sensitivity analyses made significant differences for all cytokines (including also IL-23 and IL-33), even after the FDR correction, except for MCP-1, IL-18, and IL-1β. Additionally, removal of outliers made the associations between cytokines and various dietary factors more significant, with the direction and pattern of these associations being broadly consistent with previously reported or biologically plausible relationships described in the literature. Therefore, despite the substantially smaller sample size in the reduced dataset and the consequent expected reduction in statistical power, the statistical significance of several observed differences and associations was maintained or even strengthened. Nevertheless, these findings should be interpreted cautiously, as post hoc exclusion of observations has several limitations, including the potential for selection bias, loss of genuine biological variability and analytical flexibility, and reduced representativeness of the study sample. Consequently, all primary analyses and conclusions presented in the manuscript are based on the full dataset, whereas the reduced dataset was used solely as a sensitivity analysis. Importantly, the exclusions were distributed almost equally between the two dietary groups and two genders. Consequently, although the overall sample size was substantially reduced, the reduced dataset remained well balanced with respect to the number of participants in the vegetarian and omnivorous groups and sex. This is important because the reduced dataset was not characterized by a disproportionate exclusion of participants from either dietary group. Nevertheless, we cannot determine whether the extreme cytokine values were related to pre-analytical factors, such as sample handling or processing, analytical factors (e.g., matrix effects, background fluorescence, cross-reactivity), or to genuine biological variability. However, given the marked variability observed for some cytokines, we considered it appropriate to apply a stringent criterion as an additional robustness check. The reduced dataset was therefore not intended to represent a "corrected" dataset or to imply that the excluded samples were technically invalid. Rather, this analysis was designed to determine whether the main findings persisted after removal of the most extreme observations. The overall consistency of the findings between the full and reduced datasets, together with the comparable exclusion of participants from the two dietary groups and the persistence of the findings after covariate adjustment, supports the robustness of the observed associations and indicates that they were not solely driven by extreme observations.

Various serum cytokines (except MCP-1) also exhibited weak associations with several biochemical markers known to be highly influenced by dietary factors, including total cholesterol, LDL-cholesterol, urea, creatinine, total proteins, total bilirubin, and uric acid, further supporting the effects of diet on cytokine production. The correlations of serum cytokines with total cholesterol and LDL-cholesterol, which are often associated with saturated fat and cholesterol intakes [206,207], are not unexpected. Likewise, serum urea, total protein, and creatinine may partly reflect recent dietary protein and meat intake in healthy individuals [208,209].

Negative associations observed between several cytokines and circulating levels of bilirubin and uric acid may be explained by their recognized antioxidative and immunomodulatory properties. Both bilirubin and uric acid have been described as endogenous antioxidants capable of modulating inflammatory processes [210,211]. Bilirubin suppresses pro-inflammatory cytokine production and immune cell activation, at least in part through inhibition of the NF-κB signaling and inflammasome activation, and through activation of the Nrf2/ heme oxygenase-1 (HO-1) pathway [212,213,214,215]. Conversely, the antioxidative and anti-inflammatory Nrf2/HO-1 pathway is involved in bilirubin synthesis [215,216]. In addition, bilirubin has been shown to attenuate inflammatory cell infiltration and modulate T-cell responses, supporting its role as a negative regulator of inflammation [212,217]. Uric acid exhibits a dual biological role in inflammation. At physiological concentrations, it functions as an extracellular antioxidant by scavenging ROS and contributing to redox homeostasis [211,218,219]. In contrast, at elevated levels or under specific conditions, uric acid may exert pro-inflammatory effects, particularly through activation of the Toll-like receptors (TLRs)-2 and TLR-4, NF-kB, and NLRP3 inflammasome [211,218,219,220]. Thus, within the physiological range observed in this study, uric acid is more likely to contribute to attenuation of oxidative stress-related inflammatory pathways rather than act as a primary driver of inflammation. However, since bilirubin and uric acid concentrations did not differ between dietary groups, it is unlikely that they mediate the association between vegetarian dietary patterns and circulating cytokine levels. This is further supported by regression analyses, in which adjustment for bilirubin or uric acid did not attenuate the effect of diet, but rather identified them as independent contributors. In addition, we did not observe associations between dietary factors and serum bilirubin and uric acid levels.

In our study, we also found that subjects on a vegetarian diet had lower total leucocyte and lymphocyte counts, and borderline lower eosinophil counts, with no significant difference in neutrophil and monocyte counts, although they were also lower. However, local tissue concentrations of neutrophils and monocytes could be changed due to chemotactic withdrawal from circulation under local chemokine production. Other studies have also found the same effect of a vegetarian diet on lower total leukocyte count and, to a lesser extent, lymphocyte and neutrophil counts, but the effect varied across studies [25,35,36,37,40]. However, we failed to demonstrate associations between cytokine levels and specific leucocyte counts.

Unexpectedly, our study failed to confirm the differences in CRP between the two dietary patterns, which is in contrast with other studies that generally have found lower CRP levels among vegetarians, particularly among vegans [25,26,28,31], although not all studies confirmed that finding [25,26,27,41,166,167]. The lack of observed differences in CRP should, however, be interpreted with caution, given the characteristics of the study population and analytical approach. Critically, no high-sensitivity (*hs*)-CRP was available for our study. In healthy populations, *hs*-CRP provides a more sensitive measure of low-grade systemic inflammation and cardiometabolic risk than standard CRP [49,221], which is primarily suited for detecting acute-phase inflammatory responses. Additionally, we included healthy, normal-weight adults, with no differences between the two groups in terms of anthropometrics, while the CRP levels are strongly influenced by body weight and adipose tissue mass [29,31,144,222,223]. Obesity is associated with increased production of CRP and IL-6, which is the main trigger for CRP production in the liver [130,223,224], by both adipocytes and macrophages in adipose tissue [225]. Mature adipocytes are also able to secrete CRP, and with obesity, this local CRP secretion increases [29,222,225]. In numerous studies, both IL-6 and CRP correlate with BMI [144,222,223]. However, as we did not have a wide range of BMI, we did not observe a significant correlation between BMI and CRP. In agreement, one study showed that differences between vegetarians and omnivores in CRP were mostly mediated by differences in BMI [29], while, in our study, BMI was comparable between the dietary groups. CRP levels were significantly positively associated with IL-18, IL-6, IL-8, and MCP-1; however, only the association with IL-18 remained significant after correction for multiple testing. This finding is consistent with the established involvement of IL-6 and IL-18 in the regulation of acute-phase protein production [130,224,226,227,228,229]. However, the lack of *hs*-CRP measurements could have contributed to the limited number of significant associations observed with cytokine levels.

In general, no correlation was found between cytokine levels and anthropometric indices, except for body weight and BMI (with IL-6), hip circumference (with IL-6, IL-12, IL-17A, IL-23, and IL-33), and centripetal obesity (with TNF-α and IL-8) after correcting for possible confounders, only partly confirming positive associations found in other studies [144,223,230]. However, correction for multiple testing eliminated these associations. Similarly to CRP, the main reason for not observing the expected associations with anthropometric indices is most probably the narrow selection of subjects regarding the overall nutritional status and inclusion only of normal-weight subjects, with a narrow range of BMI.

In our study, we did not find associations between cytokine levels and age, sex, smoking, and physical activity, except for female sex being associated with higher IL-1β, IFN-γ, or TNF-α (depending on the model), and age being associated with higher MCP-1 and IL-10. Other studies have shown the possible influence of those factors on cytokine production and baseline levels, but often with conflicting results, and quite contradicting our results [86,135,136,144,231,232,233]. In general, studies in humans suggest that sex- and age-associated changes in cytokine production and serum levels are not consistent [84,85,135,136,137,231,234,235,236].

### 4.1. Strengths and Limitations

This study has several strengths. The use of a flow cytometry–based multiplex cytokine assay (LEGENDplex™ Human Inflammation Panel 1) enabled simultaneous quantification of multiple cytokines and chemokines from small-volume serum samples, improving efficiency, reducing sample consumption, and allowing high-throughput analysis compared with conventional single-plex methods [56,60]. The bead-based flow cytometry format provides a broad dynamic range and automated and standardized measurements, supporting reproducibility [56,60]. To minimize inter-assay variability, all cytokine and chemokine measurements were performed within a single analytical run using a single calibration. Additionally, vegetarians and omnivores were analyzed in parallel to minimize potential limitations associated with processing all samples within a single analytical batch (including reagent instability, within-batch matrix effects, bead aggregation, and possible flow cytometer performance fluctuations), any of which could otherwise introduce systematic bias across the dataset. Quality control samples were evenly distributed throughout the batch to ensure analytical stability and monitor assay precision. The study also benefits from a well-characterized population of healthy adults, including both sexes, with careful control for major confounders such as age, body mass index, smoking status, and physical activity. Strict inclusion criteria and exclusion of acute and chronic conditions and other states that could influence immune status or data relevance, supported by clinical and laboratory parameters, reduced potential bias related to underlying health conditions. In addition, the study included a comprehensive set of validated measurements, including anthropometric, biochemical, hematological, and dietary data, enabling more robust adjustment for confounding and exploration of potential mechanisms, with no missing data present.

However, several limitations should be considered when interpreting our findings. First, cytokine concentrations were assessed from a single blood sample, assuming that one time-point measurement reflects medium-term inflammatory status. This approach does not capture existing temporal variability (dynamic cytokine fluctuations) or functional immune activity, and previous studies have shown variable temporal repeatability of cytokine measurements, with particularly low reliability for some markers [137,142,144]. Repeated measurements would provide more accurate estimates, especially for correlation analyses, as the sample size was primarily defined to detect differences between groups. Additionally, in our study sample, we found a higher inter-individual variability in cytokine concentrations, which reduced statistical power and increased the likelihood of type II error (i.e., inability to detect significant differences), particularly in the context of multiple comparisons. Therefore, a larger sample size or repeated measurements may be required to more reliably detect group differences and correlations with dietary factors. Our sample size calculation was based on a single cytokine, while data for the majority of other cytokines were not available. In particular, cytokines with greater biological and/or analytical variability may require larger sample sizes to reliably detect between-group differences. In addition, adjustment for multiple testing may further increase the required sample size. However, repeated sampling or increased sample size is associated with increased costs, logistical complexity, and additional burden for study participants, which may limit its feasibility. In addition, we did not measure specific cytokine-binding proteins (e.g., IL-1RA, sIL-1R, IL-18BP, sST2), which would provide better insights into the free (i.e., bioavailable)cytokine concentrations, which are very important for some cytokine functions. Although multiplexing represents a key strength, bead-based immunoassays are subject to technical limitations, including antibody cross-reactivity, uncertainty regarding the detection of free, bound, precursor, or partially degraded cytokine forms, signal overlap between bead populations, background fluorescence, and matrix effects inherent to complex biological samples such as serum, all of which may affect analytical accuracy or interpretation [56,195,205,237]. In addition, the sensitivity of this method is generally sufficient for moderate-to-high-abundance cytokines but may be limited for low-concentration cytokines, which are typical in healthy populations, thereby increasing measurement error [195,238]. More advanced proteomic approaches, such as electrochemiluminescence or proximity extension assays, offer higher sensitivity, improved reproducibility, and broader protein coverage from small sample volumes, but are costly and require specialized platforms [239,240,241,242]. Flow cytometry–based analyses also require well-calibrated instruments, and reliance on proprietary reagents and software may reduce comparability across studies and different platforms. As previously described, cytokine concentrations measured by multiplex bead-based assays may differ from those obtained using ELISA or other methods [56,152,241,243]. Therefore, absolute cytokine concentrations should be interpreted with caution and may not be directly comparable across different analytical platforms. In addition, the cytokine samples were not analyzed in analytical duplicates due to financial constraints. Moreover, serum measurements may be further influenced by ex vivo cytokine release during coagulation and sample processing [56,198,244]. It is conceivable that these methodological limitations reduced sensitivity for detecting subtle differences in all analyzed cytokines. EDTA plasma may be more suitable than serum for circulating cytokine measurements, provided that strict preanalytical handling procedures are followed (e.g., immediate cooling of blood samples, centrifugation within 1–2 h of collection, storage at −80 °C up to one year, and avoidance of freeze–thaw cycles) [198,199,244]. Importantly, our study was not adjusted for the duration of sample storage, which may affect the stability of some cytokines [59]. However, participants from both dietary groups were recruited and sampled in parallel during the same study period, rather than sequentially, which may have limited systematic differences in sample storage duration between the groups. Although a standard 12 h overnight fast is the conventional and preferred preparation for measuring baseline serum cytokine levels [56,141], several cytokines (e.g., MCP-1) may remain altered for up to 12 h or longer following bolus LPS or certain meals (e.g., high fat). However, prolonged fasting can induce significant metabolic changes and hormonal shifts, which can suppress or alter basal circulating cytokine concentrations [245]; additionally, they can induce greater discomfort for participants. Furthermore, our cytokine panel did not include other relevant markers (e.g., TGF-β1, IL-2, IL-4, IL-13), and no functional immune assays (e.g., stimulation tests such as LPS-induced cytokine production) were performed, which would provide a more comprehensive assessment of immune responsiveness [246]. In addition, standard CRP was measured rather than *hs*-CRP, a more sensitive measure of low-grade systemic inflammation and cardiometabolic risk [49]. The vegetarian group was heterogeneous, with an unequal distribution of vegans and lacto-ovo vegetarians. Although both groups showed lower cytokine levels, the relatively small number of lacto-ovo vegetarians limited statistical power to detect subgroup-specific differences, and findings should be interpreted with caution. Additionally, participants were healthy adults from a single geographic area (Belgrade), which may limit generalizability. However, given the lack of prior data on vegetarian dietary patterns in the Serbian population [15], this study provides important initial insights. Several limitations related to dietary assessment should also be acknowledged. Dietary intake was assessed using two 24 h dietary recalls. This method is subject to recall bias, misreporting, and dependence on the quality of food composition databases, potentially affecting nutrient estimates [247]. Two 24 h dietary recalls may be too few to fully represent habitual intake, particularly for certain nutrients and food components with episodic consumption [247,248]. For such dietary factors, two recall days may not adequately represent an individual’s usual intake, introducing within-person measurement error and potentially attenuating associations with circulating biomarkers. Moreover, only one of the 24 h recalls was closely aligned with blood sampling, whereas the other was conducted within one month after blood sampling. Thus, the weaker associations observed in correlation analyses compared with between-group comparisons may partly reflect limitations in estimating habitual individual intake and the temporal distance between dietary assessment and biomarker measurement. In contrast, for between-group comparisons, day-to-day variation is expected to be distributed across participants and is therefore less likely to systematically affect the comparison between groups, particularly given the substantial differences in their overall dietary patterns. Another limitation is that the DII and E-DII were calculated using 28 of the 45 components included in the original algorithms [69,75], as the remaining components were not available in our dietary database. Although reduced-component DII/E-DII approaches have been used in previous epidemiological studies [70,71,72,73,74], this specific 28-component implementation has not been separately validated in the Serbian population, and obtained scores cannot be used for comparison with differently calculated DII/E-DII scores. Nevertheless, the same calculations were applied to all participants in the present study, allowing consistent within-study comparison of the dietary inflammatory potential. Finally, and crucially, the cross-sectional design precludes causal inference, and the high degree of multicollinearity among dietary variables introduces potential confounding, limiting the ability to distinguish independent associations between specific dietary factors and cytokine levels. In addition, data on the status of specific micro- and macronutrients are currently lacking, which limits the ability to confirm associations with individual dietary factors.

### 4.2. Clinical Implications and Further Directions

The clinical significance of the observed lower cytokine concentrations with a vegetarian diet remains uncertain, as reduced circulating levels do not necessarily translate into improved health outcomes without clear evidence that they improve disease risk, outcomes, or immune responses [5,6]. Indeed, it is still not well established whether a generally attenuated cytokine response is beneficial across different contexts, including acute infections (e.g., viral, bacterial, or parasitic), chronic conditions (e.g., cardiovascular or neurodegenerative diseases), or autoimmune disorders [5,6]. For example, during COVID-19, both exaggerated inflammatory responses (“cytokine storm”) and insufficient antiviral immune activation were associated with adverse outcomes, highlighting that the optimal immune response is highly context-dependent and balanced, rather than uniformly “lower” or “higher” [4,124,249,250,251,252]. Nevertheless, there is still some evidence suggesting that plant-based diets may be associated with a lower risk of COVID-19 infection or more favorable clinical outcomes [250,253,254]. However, only three studies have investigated these associations, and they either did not specifically include strict vegetarians or vegans (i.e., they included flexitarians) or included only a small number of participants following vegetarian diets (e.g., 22 patients). Thus, the underlying mechanisms and causality remain to be fully established.

Future research should therefore extend beyond the measurement of static circulating immune markers and examine functional immune responses to relevant immunological challenges, such as vaccination and controlled immune stimulation in vivo or in vitro. Such approaches would provide greater insight into whether the observed differences in cytokine profiles translate into meaningful differences in immune competence and disease susceptibility.

In addition, longitudinal and mechanistic studies are needed to confirm whether vegetarian dietary patterns influence immune regulation and to clarify the biological pathways involved. Attention should be given to examining nutrient status, including nutrients that differ substantially between vegetarian and omnivorous diets, as these may partly mediate the observed immunological differences. Future studies would also benefit from larger sample sizes, optimized and standardized pre-analytical procedures (including appropriate selection of sample matrix, rapid sample processing, and standardized storage conditions), the use of more sensitive analytical platforms, and repeated biological sampling to improve statistical power, analytical accuracy, and account for within-person temporal variability.

## 5. Conclusions

In conclusion, this study demonstrates that adherence to a vegetarian dietary pattern—including both vegan and lacto-ovo vegetarian diets—is associated with lower circulating levels of selected cytokines and chemokines in a cohort of healthy adults, independent of sex, age, body weight, or other lifestyle-related confounders. These findings extend existing evidence beyond CRP, a major systemic inflammation marker, or leucocyte counts.

However, the number of associations remaining after multiple-testing correction was limited, while the cross-sectional design and the high degree of intercorrelation among dietary variables complicate the identification of specific dietary components underlying these associations, making it difficult to distinguish causal effects from potential confounding. In addition, the use of the twice-repeated 24 h recall method has inherent limitations and may not fully capture habitual or long-term dietary intake, while the lack of biochemical measures of specific nutrient status, which could provide objective confirmation of long-term intake, further limits our ability to disentangle the specific dietary factors that may be involved. Moreover, given the complex and context-dependent role of cytokines in immune function, it remains unclear whether lower circulating levels necessarily reflect a beneficial immunological state across different health conditions.

Future studies are therefore warranted to test causality, clarify the contribution of individual dietary factors, elucidate underlying biological mechanisms, investigate functional immune responses to different stimuli, and determine the clinical relevance of these immunological differences.

## Figures and Tables

**Table 1:** Comparison of socioeconomic, lifestyle, and anthropometric data between subjects following vegetarian vs. omnivorous diets.

	Vegetarian Diet (*n* = 80)		Omnivorous Diet (*n* = 80)		Pearson’s Chi-Square/ Mann–Whitney
	*n*/Median	(%)/(IQR)	*n*/Median	(%)/(IQR)	*p*
Sex:					1.000
Men, *n* (%)	40	(50.0%)	40	(50.0%)	
Women, *n* (%)	40	(50.0%)	40	(50.0%)	
Age (years)	34.0	(28.0–39.0)	33.0	(28.0–40.0)	0.965
Smoking status:					0.168
Never smoker, *n* (%)	47	(58.8%)	48	(60.0%)	
Former smoker, *n* (%)	24	(30.0%)	16	(20.0%)	
Current smoker, *n* (%)	9	(11.3%)	16	(20.0%)	
Physical activity:					
Sedentary, *n* (%)	26	(32.5%)	20	(25.0%)	0.577
Moderately active, *n* (%)	34	(42.5%)	38	(47.5%)	
Active, *n* (%)	20	(25.0%)	22	(27.5%)	
Height (cm)	175.0	(168.0–182.0)	174.5	(169.0–184.8)	0.310
Weight (kg)	67.0	(58.0–78.0)	69.5	(58.3–80.0)	0.390
BMI (kg/m^2^)	22.3	(20.1–24.5)	22.6	(20.6–24.3)	0.672
Waist circumference (cm)	80.0	(73.0–87.8)	81.5	(72.0–90.0)	0.458
Hip circumference (cm)	94.5	(90.0–100.0)	95.0	(88.3–99.0)	0.975
WHR	0.86	(0.79–0.92)	0.86	(0.80–0.92)	0.688
WHtR	0.46	(0.43–0.49)	0.46	(0.43–0.49)	0.783

BMI = body mass index, WHR = waist-to-hip ratio, WHtR = waist-to-height ratio, IQR = interquartile range, *p* = statistical significance of the difference for Mann–Whitney test or Pearson’s chi-square test, as appropriate. Exploratory analyses. Nominal *p*-values are reported.

**Table 2:** Comparison of standard biochemistry serum metabolite levels between subjects following vegetarian vs. omnivorous diets.

	Vegetarian Diet (*n* = 80)		Omnivorous Diet (*n* = 80)		Mann–Whitney	Quade’s ANCOVA
Biochemistry Metabolite	Median	(IQR)	Median	(IQR)	*p*	*p*′
Glucose (mmol/L)	4.8	(4.5–5.2)	4.9	(4.7–5.3)	**0.043**	0.057
Total cholesterol (mmol/L)	4.0	(3.4–4.5)	4.8	(4.3–5.5)	<0.001 *	<0.001 *
HDL-cholesterol (mmol/L)	1.4	(1.2–1.7)	1.6	(1.4–1.9)	0.006 *	0.002 *
LDL-cholesterol (mmol/L)	2.1	(1.7–2.6)	2.7	(2.2–3.3)	<0.001 *	<0.001 *
Triglycerides (mmol/L)	0.82	(0.59–0.99)	0.85	(0.62–1.37)	0.303	0.203
Urea (mmol/L)	3.5	(2.8–4.2)	4.4	(3.4–5.2)	<0.001 *	<0.001 *
Creatinine (µmol/L)	72.5	(63.2–79.0)	78.0	(65.3–88.0)	**0.025**	<0.001 *
Total bilirubin (µmol/L)	11.8	(8.4–15.9)	11.4	(7.8–15.7)	0.707	0.707
Uric acid (µmol/L)	278.0	(226.3–334.8)	257.0	(223.0–318.8)	0.230	0.144
Total protein (g/L)	72.0	(69.5–74.8)	71.9	(69.6–75.0)	0.967	0.851
Serum Fe (µmol/L)	20.1	(15.0–26.5)	18.8	(14.2–26.7)	0.651	0.666
ALP (U/L)	63.0	(52.3–77.8)	56.5	(48.0–66.0)	**0.034**	**0.036**
ALT (U/L)	15.6	(11.7–21.3)	17.1	(12.2–21.8)	0.401	0.288
AST (U/L)	17.9	(15.3–22.3)	18.8	(15.6–22.9)	0.526	0.447
GGT (U/L)	11.8	(9.0–15.0)	13.3	(11.0–17.0)	**0.033**	**0.009**

HDL = high-density lipoprotein, LDL = low-density lipoprotein, ALP = alkaline phosphatase, ALT = alanine aminotransferase, AST = aspartate aminotransferase, GGT = γ-glutamyl transferase, IQR = interquartile range, *p* = statistical significance of the difference for Mann–Whitney test, *p*′ = statistical significance of the difference for Quade’s non-parametric analysis of covariance (ANCOVA), with adjustment for sex, age, BMI, smoking, and physical activity level. Secondary outcomes. Nominal *p*-values are reported. Bold values indicate statistical significance at *p* < 0.05. Values marked with an asterisk (*) remained statistically significant after Benjamini–Hochberg false discovery rate (FDR) correction for 15 biochemistry-related multiple comparisons.

**Table 3:** Comparison of standard hematological parameters and serum CRP levels between subjects following vegetarian vs. omnivorous diets.

	Vegetarian Diet (*n* = 80)		Omnivorous Diet (*n* = 80)		Mann–Whitney	Quade’s ANCOVA
Hematological Parameter/CRP	Median	(IQR)	Median	(IQR)	*p*	*p*′
Erythrocyte count (×10^12^/L)	4.7	(4.4–5.0)	4.8	(4.5–5.1)	0.135	0.019 *
Hemoglobin (g/L)	142.1	(135.0–153.1)	145.5	(136.8–157.4)	0.166	0.021 *
Hematocrit (L/L)	0.43	(0.41–0.46)	0.44	(0.41–0.46)	0.599	0.272
MCV (fL)	92.3	(89.7–95.0)	90.4	(88.5–93.1)	0.008 *	0.007 *
MCH (pg)	30.6	(29.8–32.1)	30.4	(29.7–31.4)	0.502	0.400
MCHC (g/L)	331.8	(327.2–336.7)	336.0	(332.0–341.8)	<0.001 *	<0.001 *
Platelet count (×10^9^/L)	252.8	(197.7–286.8)	229.5	(190.0–279.5)	0.404	0.370
Leukocyte count (×10^9^/L)	5.3	(4.8–6.4)	6.1	(5.3–7.3)	0.004 *	0.003 *
Neutrophil count (×10^9^/L)	3.04	(2.58–3.81)	3.29	(2.73–4.13)	0.111	0.090
Lymphocyte count (×10^9^/L)	1.83	(1.47–2.09)	2.04	(1.74–2.34)	0.004 *	0.004 *
Monocyte count (×10^9^/L)	0.44	(0.37–0.53)	0.47	(0.37–0.57)	0.217	0.211
Eosinophil count (×10^9^/L)	0.12	(0.06–0.22)	0.16	(0.09–0.24)	**0.050**	0.059
Basophil count (×10^12^/L)	0.03	(0.02–0.04)	0.03	(0.02–0.04)	0.481	0.530
CRP (mg/L)	1.45	(1.20–1.80)	1.50	(1.20–1.90)	0.984	1.000

MCV = mean corpuscular volume, MCH = mean corpuscular hemoglobin, MCHC = mean corpuscular hemoglobin concentration, CRP = C-reactive protein, IQR = interquartile range, *p* = statistical significance of the difference for Mann–Whitney test, *p*′ = statistical significance of the difference for Quade’s non-parametric analysis of covariance (ANCOVA), with adjustment for sex, age, BMI, smoking, and physical activity level. Secondary outcomes. Nominal *p*-values are reported. Bold values indicate statistical significance at *p* < 0.05. Values marked with an asterisk (*) remained statistically significant after Benjamini–Hochberg false discovery rate (FDR) correction for 13 hematological multiple comparisons; CRP was analyzed separately.

**Table 4:** Comparison of serum cytokine levels between subjects following vegetarian vs. omnivorous diets.

	Vegetarian Diet (*n* = 80)		Omnivorous Diet (*n* = 80)		Mann–Whitney	Quade’s ANCOVA
Cytokine/Chemokine	Median	(IQR)	Median	(IQR)	*p*	*p*′
IL-1β (pg/mL)	41.6	(2.9–141.6)	45.6	(14.9–106.9)	0.511	0.473
IFN-α2 (pg/mL)	5.5	(2.5–11.7)	8.1	(3.5–15.6)	**0.037**	**0.043**
IFN-γ (pg/mL)	8.5	(2.6–23.3)	15.5	(8.2–25.3)	0.006 *	0.006 *
TNF-α (pg/mL)	13.8	(4.0–44.3)	26.4	(12.3–53.6)	0.019 *	**0.028**
MCP-1 (pg/mL)	308.5	(221.7–399.9)	273.8	(187.6–375.0)	0.316	0.378
IL-6 (pg/mL)	7.7	(3.3–20.9)	14.0	(5.6–27.9)	0.018 *	**0.026**
IL-8 (pg/mL)	24.7	(1.9–120.8)	75.8	(34.2–169.6)	0.002 *	0.001 *
IL-10 (pg/mL)	7.1	(2.4–15.5)	10.3	(4.4–26.0)	0.024 *	**0.024**
IL-12p70 (pg/mL)	5.3	(1.9–13.0)	8.8	(3.9–17.0)	0.010 *	0.008 *
IL-17A (pg/mL)	1.0	(0.2–2.6)	2.1	(0.9–4.2)	<0.001 *	<0.001 *
IL-18 (pg/mL)	261.4	(168.9–342.6)	268.7	(180.1–391.2)	0.213	0.182
IL-23 (pg/mL)	11.6	(0.9–21.4)	13.1	(4.3–36.5)	0.077	0.077
IL-33 (pg/mL)	54.6	(17.4–208.1)	81.3	(32.7–217.0)	0.130	0.128

IL = interleukin, IFN = interferon, TNF = tumor necrosis factor, MCP = monocyte chemoattractant protein, IQR = interquartile range, *p* = statistical significance of the difference for Mann–Whitney test, *p*′ = statistical significance of the difference for Quade’s non-parametric analysis of covariance (ANCOVA), with adjustment for sex, age, BMI, smoking, and physical activity level. Primary outcomes. Nominal *p*-values are reported. Bold values indicate statistical significance at *p* < 0.05. Values marked with an asterisk (*) remained statistically significant after Benjamini–Hochberg false discovery rate (FDR) correction for 13 cytokine/chemokine multiple comparisons.

**Table 5:** Comparison of energy intake coming from different food groups between subjects following vegetarian vs. omnivorous diets.

	Vegetarian Diet (*n* = 80)		Omnivorous Diet (*n* = 80)		Mann–Whitney	Quade’s ANCOVA
Food Groups’ Energy Contribution	Median	(IQR)	Median	(IQR)	*p*	*p*′
Energy from milk products (kcal/day)	0.0	(0.0–7.2)	236.2	(153.4–339.0)	<0.001 *	<0.001 *
Energy from egg products (kcal/day)	0.0	(0.0–4.8)	93.9	(14.6–184.5)	<0.001 *	<0.001 *
Energy from meat products (kcal/day)	0.0	(0.0–0.0)	385.0	(235.7–586.7)	<0.001 *	<0.001 *
Energy from fish products (kcal/day)	0.0	(0.0–0.0)	0.0	(0.0–161.6)	<0.001 *	<0.001 *
Energy from edible fats/oils (kcal/day)	352.4	(198.4–523.3)	416.9	(285.6–576.6)	0.026 *	0.012 *
Energy from grain products (kcal/day)	575.1	(344.6–755.7)	509.9	(402.6–731.5)	0.907	0.888
Energy from nuts/seeds (kcal/day)	265.9	(137.0–520.5)	90.9	(13.2–199.8)	<0.001 *	<0.001 *
Energy from vegetable products (kcal/day)	401.5	(271.9–570.7)	159.9	(106.5–268.7)	<0.001 *	<0.001 *
Energy from fruit products (kcal/day)	278.2	(153.0–435.5)	89.4	(1.7–163.1)	<0.001 *	<0.001 *
Energy from sugar products (kcal/day)	41.1	(0.0–141.6)	157.8	(24.3–306.7)	<0.001 *	<0.001 *
Energy from beverages (kcal/day)	2.6	(0.0–82.3)	5.5	(0.0–109.3)	0.609	0.572
% Energy from milk products (%kcal/day)	0.0	(0.0–0.3)	9.0	(6.1–12.9)	<0.001 *	<0.001 *
% Energy from egg products (%kcal/day)	0.0	(0.0–0.2)	3.5	(0.5–5.9)	<0.001 *	<0.001 *
% Energy from meat products (%kcal/day)	0.0	(0.0–0.0)	16.2	(10.4–23.1)	<0.001 *	<0.001 *
% Energy from fish products (%kcal/day)	0.0	(0.0–0.0)	0.0	(0.0–6.9)	<0.001 *	<0.001 *
% Energy from edible fats/oils (%kcal/day)	15.4	(8.6–21.4)	16.3	(12.1–21.7)	0.290	0.224
% Energy from grain products (%kcal/day)	24.0	(15.6–32.5)	20.9	(15.8–26.0)	0.177	0.154
% Energy from nuts/seeds (%kcal/day)	12.6	(4.8–22.1)	3.3	(0.6–8.2)	<0.001 *	<0.001 *
% Energy from vegetable prod. (%kcal/day)	18.4	(11.4–24.9)	6.2	(3.9–10.7)	<0.001 *	<0.001 *
% Energy from fruit products (%kcal/day)	11.9	(6.4–20.1)	3.7	(0.1–5.9)	<0.001 *	<0.001 *
% Energy from sugar products (%kcal/day)	1.6	(0.0–6.9)	6.2	(1.0–11.5)	0.002 *	0.002 *
% Energy from beverages (%kcal/day)	0.1	(0.0–3.7)	0.2	(0.0–4.5)	0.903	0.870

IQR = interquartile range, *p* = statistical significance of the difference for Mann–Whitney test, *p*′ = statistical significance of the difference for Quade’s non-parametric analysis of covariance (ANCOVA), with adjustment for sex, age, BMI, smoking, and physical activity level. Secondary outcomes. Nominal *p*-values are reported. Bold values indicate statistical significance at *p* < 0.05. Values marked with an asterisk (*) remained statistically significant after Benjamini–Hochberg false discovery rate (FDR) correction for 76 dietary multiple comparisons.

**Table 6:** Comparison of dietary macronutrient intakes between subjects following vegetarian vs. omnivorous diets.

	Vegetarian Diet (*n* = 80)		Omnivorous Diet (*n* = 80)		Mann–Whitney	Quade’s ANCOVA
Macronutrient and Its Energy Contribution	Median	(IQR)	Median	(IQR)	*p*	*p*′
Energy intake (kcal/day)	2234.3	(1932.6–2667.6)	2614.0	(2093.5–3040.4)	0.006 *	<0.001 *
Energy intake per kg body mass (kcal/kg/day)	32.8	(29.4–39.8)	36.0	(33.1–42.8)	0.009 *	**0.010**
Protein intake (g/day)	67.1	(48.4–83.0)	109.2	(85.0–128.1)	<0.001 *	<0.001 *
Carbohydrate intake (g/day)	244.3	(207.7–318.9)	206.7	(163.3–254.4)	<0.001 *	<0.001 *
Fat intake (g/day)	90.3	(74.2–116.3)	137.2	(113.5–168.2)	<0.001 *	<0.001 *
Saturated fat intake (g/day)	18.3	(13.2–25.3)	39.4	(30.9–53.0)	<0.001 *	<0.001 *
Monounsaturated fat intake (g/day)	36.2	(28.1–47.4)	48.2	(40.0–56.4)	<0.001 *	<0.001 *
Polyunsaturated fat intake (g/day)	27.9	(21.5–38.6)	35.1	(28.1–47.4)	<0.001 *	<0.001 *
n-6 polyunsaturated fat intake (g/day)	19.9	(14.4–30.3)	28.4	(20.6–36.8)	<0.001 *	<0.001 *
n-3 polyunsaturated fat intake (g/day)	1.5	(0.8–2.8)	1.5	(1.1–2.2)	0.741	0.759
n-6/n-3 polyunsaturated fat intake ratio	12.7	(7.4–24.9)	18.5	(12.5–26.7)	0.012 *	0.008 *
Trans-fat intake (g/day)	0.3	(0.0–0.8)	1.2	(0.4–2.9)	<0.001 *	<0.001 *
Cholesterol intake (mg/day)	5.0	(0.0–35.1)	428.0	(281.3–588.2)	<0.001 *	<0.001 *
Fiber intake (g/day)	43.5	(34.2–56.4)	22.2	(16.6–27.8)	<0.001 *	<0.001 *
Alcohol intake (g/day)	0.0	(0.0–0.1)	0.0	(0.0–5.0)	0.630	0.586
% Energy from protein (%kcal/day)	11.5	(9.7–13.7)	16.3	(14.2–18.3)	<0.001 *	<0.001 *
% Energy from carbohydrate (%kcal/day)	45.0	(39.2–51.1)	32.9	(27.7–38.0)	<0.001 *	<0.001 *
% Energy from fat (%kcal/day)	36.6	(32.2–43.2)	48.1	(41.6–52.6)	<0.001 *	<0.001 *
% Energy from saturated fat (%kcal/day)	7.4	(5.5–9.6)	14.3	(12.5–16.5)	<0.001 *	<0.001 *
% Energy from monounsaturated fat (%kcal/day)	14.5	(11.3–17.4)	16.8	(14.6–18.9)	<0.001 *	<0.001 *
% Energy from polyunsaturated fat (%kcal/day)	11.8	(9.4–13.9)	12.2	(10.0–15.6)	0.054	0.061
% Energy from n-6 polyunsaturated fat (%kcal/day)	0.5	(0.4–0.9)	0.5	(0.4–0.8)	0.298	0.265
% Energy from n-3 polyunsaturated fat (%kcal/day)	8.5	(6.2–11.1)	10.2	(8.1–12.2)	0.002 *	0.001 *
% Energy from trans-fat (%kcal/day)	0.1	(0.0–0.3)	0.4	(0.2–1.0)	<0.001 *	<0.001 *
% Energy from fiber (%kcal/day)	3.9	(3.2–4.7)	1.7	(1.2–2.3)	<0.001 *	<0.001 *
% Energy from alcohol (%kcal/day)	0.0	(0.0–0.0)	0.0	(0.0–1.2)	0.712	0.664

IQR = interquartile range, *p* = statistical significance of the difference for Mann–Whitney test, *p*′ = statistical significance of the difference for Quade’s non-parametric analysis of covariance (ANCOVA), with adjustment for sex, age, BMI, smoking, and physical activity level, except for energy intake per kg body mass, where adjusted for sex, age, smoking, and physical activity level. Secondary outcomes. Nominal *p*-values are reported. Bold values indicate statistical significance at *p* < 0.05. Values marked with an asterisk (*) remained statistically significant after Benjamini–Hochberg false discovery rate (FDR) correction for 76 dietary multiple comparisons.

**Table 7:** Comparison of dietary micronutrient intakes between subjects following vegetarian vs. omnivorous diets.

	Vegetarian Diet (*n* = 80)		Omnivorous Diet (*n* = 80)		Mann–Whitney	Quade’s ANCOVA
Micronutrient/Index	Median	(IQR)	Median	(IQR)	*p*	*p*′
Fe (mg/day)	19.3	(13.7–23.4)	14.3	(11.9–16.8)	<0.001 *	<0.001 *
Zn (mg/day)	11.2	(8.3–14.2)	13.0	(9.9–15.2)	0.025 *	0.012 *
Cu (mg/day)	2.9	(2.2–3.6)	1.7	(1.4–2.1)	<0.001 *	<0.001 *
I (µg/day)	126.7	(97.8–157.8)	207.4	(174.4–247.3)	<0.001 *	<0.001 *
Se (µg/day)	25.3	(17.2–32.0)	59.1	(43.4–74.5)	<0.001 *	<0.001 *
Ca (mg/day)	729.4	(553.9–888.7)	794.2	(593.5–1057.7)	0.136	0.094
P (mg/day)	1334.4	(1005.8–1670.0)	1644.9	(1384.0–1939.5)	<0.001 *	<0.001 *
Mg (mg/day)	629.9	(478.2–764.5)	380.2	(319.8–474.5)	<0.001 *	<0.001 *
K (mg/day)	4156.4	(3371.2–5210.6)	3293.0	(2801.4–3902.8)	<0.001 *	<0.001 *
Na (mg/day)	2649.2	(1999.2–3487.1)	3854.1	(3023.1–4633.0)	<0.001 *	<0.001 *
NaCl (mg/day)	4333.4	(2602.4–5650.6)	4615.8	(3535.6–6043.7)	0.112	0.082
β-carotene (µg/day)	7316.4	(3076.0–11,119.2)	2944.8	(1759.2–5350.3)	<0.001 *	<0.001 *
Retinol (µg/day)	4.5	(0.0–36.7)	336.3	(225.1–515.4)	<0.001 *	<0.001 *
Vitamin A RE (µg/day)	1327.9	(592.9–1853.2)	903.8	(667.2–1444.5)	0.087	0.072
Vitamin A RAE (µg/day)	677.5	(297.1–987.8)	650.2	(497.0–889.7)	0.598	0.629
Vitamin B1 (mg/day)	2.1	(1.5–3.9)	1.9	(1.5–3.0)	0.340	0.413
Vitamin B2 (mg/day)	1.5	(1.1–1.9)	1.8	(1.4–2.5)	<0.001 *	<0.001 *
Vitamin B3 NE (mg/day)	27.1	(22.0–36.5)	42.3	(32.4–54.0)	<0.001 *	<0.001 *
Vitamin B5 (mg/day)	6.5	(4.8–10.4)	7.7	(6.2–10.0)	0.182	0.156
Vitamin B6 (mg/day)	2.2	(1.7–3.0)	2.5	(2.0–3.2)	0.191	0.152
Vitamin B9 (µg/day)	555.0	(429.3–716.9)	327.2	(269.7–407.1)	<0.001 *	<0.001 *
Vitamin B12 (µg/day)	0.9	(0.3–1.4)	5.7	(4.2–8.1)	<0.001 *	<0.001 *
Vitamin C (mg/day)	206.2	(152.4–320.6)	103.3	(59.6–167.0)	<0.001 *	<0.001 *
Vitamin D (mg/day)	0.3	(0.0–1.9)	5.6	(2.5–9.0)	<0.001 *	<0.001 *
Vitamin E (mg/day)	27.3	(21.7–35.2)	28.1	(22.4–35.8)	0.430	0.334
Polyphenols (mg/day)	1543.6	(1055.1–2211.1)	786.9	(478.9–1149.4)	<0.001 *	<0.001 *
DII score	−2.77	(−3.59–−1.83)	−0.83	(−2.33–0.09)	<0.001 *	<0.001 *
E-DII score	−2.44	(−3.27–−1.53)	0.23	(−1.34–1.22)	<0.001 *	<0.001 *

RE = retinol equivalents, RAE = retinol activity equivalents, NE = niacin equivalents, DII = dietary inflammatory index, E-DII = energy-adjusted dietary inflammatory index, IQR = interquartile range, *p* = statistical significance of the difference for Mann–Whitney test, *p*′ = statistical significance of the difference for Quade’s non-parametric analysis of covariance (ANCOVA), with adjustment for sex, age, BMI, smoking, and physical activity level. Secondary outcomes. Nominal *p*-values are reported. Bold values indicate statistical significance at *p* < 0.05. Values marked with an asterisk (*) remained statistically significant after Benjamini–Hochberg false discovery rate (FDR) correction for 76 dietary multiple comparisons.

**Table 8:** Linear regression—forced-entry multivariate models for the association of vegetarian dietary pattern with serum cytokine levels (only vegetarian status coefficients presented).

Cytokine	*B*	95% *CI* for *B*	Predictor Significance *p*	Adjusted *R*^2^	Model Significance *p*
IL-1β	−0.211	(−0.478–0.057)	0.122	0.026	0.131
IFN-α2	−0.168	(−0.312–−0.025)	**0.022**	0.018	0.195
IFN-γ	−0.259	(−0.452–−0.067)	0.009 *	0.040	0.064
TNF-α	−0.235	(−0.446–−0.025)	**0.028**	0.045	0.050
MCP-1	0.023	(−0.053–0.099)	0.547	0.037	0.076
IL-6	−0.181	(−0.351–−0.012)	**0.036**	0.026	0.133
IL-8	−0.540	(−0.827–−0.254)	<0.001 *	0.068	**0.013**
IL-10	−0.206	(−0.383–−0.028)	**0.024**	0.037	0.074
IL-12p70	−0.216	(−0.374–−0.058)	0.008 *	0.052	**0.033**
IL-17A	−0.318	(−0.482–−0.154)	<0.001 *	0.092	**0.003**
IL-18	−0.049	(−0.131–0.033)	0.237	0.014	0.682
IL-23	−0.180	(−0.391–0.031)	0.093	0.020	0.177
IL-33	−0.169	(−0.362–0.024)	0.085	0.010	0.284

Cytokine/chemokine levels were log-transformed for regression analyses. Model predictors: type of diet (vegetarian vs. omnivorous), sex, age, BMI, smoking status, and physical activity level. *B* = unstandardized regression coefficient, *CI* = confidence interval, *R*^2^ = coefficient of determination, *p* = statistical significance. Exploratory analyses. Nominal *p*-values are reported. Bold values indicate statistical significance at *p* < 0.05. Predictor *p*-values marked with an asterisk (*) remained significant after Benjamini–Hochberg false discovery rate (FDR) correction for 13 cytokine/chemokine multiple testing.

## Data Availability

The datasets generated and/or analyzed during the current study are not publicly available because they are being used for ongoing analyses that have not yet been published. Further inquiries may be directed to the corresponding author.

## Supplementary Materials

The following supporting information can be downloaded at: https://www.mdpi.com/article/10.3390/metabo16100793/s1, Supplementary Section S2.1.1. The study timeline; Supplementary Figure S1. Flow diagram of the study participants’ selection; Supplementary Table S1. Analytical characteristics and quantification limits of the cytokine/chemokine assay; Supplementary Table S2. Subgroup comparison of socioeconomic, lifestyle, and anthropometric data between participants following vegan vs. lacto-ovo vegetarian vs. omnivorous diets; Supplementary Table S3. Subgroup comparison of standard biochemistry serum metabolite levels between participants following vegan vs. lacto-ovo vegetarian vs. omnivorous diets; Supplementary Table S4. Subgroup comparison of standard hematological parameters and serum CRP levels between participants following vegan vs. lacto-ovo vegetarian vs. omnivorous diets; Supplementary Table S5. Subgroup comparison of serum cytokine/chemokine levels between participants following vegan vs. lacto-ovo vegetarian vs. omnivorous diets; Supplementary Table S6. Comparison of serum cytokine levels between participants following vegetarian vs. omnivorous diets when crude analytical values below LLOQ were included in analysis; Supplementary Table S7. Comparison of serum cytokine levels between participants following vegetarian vs. omnivorous diets in a subset of participants after removal of those with statistical upper-tail outlier/extreme values; Supplementary Table S8. Within-person intercorrelation between different serum cytokines/chemokines (all dietary groups merged); Supplementary Table S9. Subgroup comparison of energy intakes coming from different food groups between participants following vegan vs. lacto-ovo vegetarian vs. omnivorous diets; Supplementary Table S10. Subgroup comparison of dietary macronutrient intakes between participants following vegan vs. lacto-ovo vegetarian vs. omnivorous diets; Supplementary Table S11. Comparison of dietary micronutrient intakes per 1000 kcal between participants following vegetarian vs. omnivorous diets; Supplementary Table S12. Subgroup comparison of dietary micronutrient intakes between participants following vegan vs. lacto-ovo vegetarian vs. omnivorous diets; Supplementary Table S13. Comparison of frequency of supplement use between participants following vegetarian and omnivorous diets; Supplementary Table S14. Reported supplement use according to 24 h recalls among participants following vegetarian and omnivorous diets; Supplementary Table S15. Correlation of serum cytokines/chemokines with energy intake from different food groups and macronutrient intakes in a subset of 82 participants (comprising 42 vegetarians and 40 omnivores) after removal of those with statistical upper-tail outlier/extreme values; Supplementary Table S16. Correlation of serum cytokines/chemokines with energy intake from different food groups and macronutrient intakes in a subset of 82 participants (comprising 42 vegetarians and 40 omnivores) after removal of those with statistical upper-tail outlier/extreme values, with adjustment for potential confounders; Supplementary Table S17. Correlations among various food groups and dietary factors (heatmap); Supplementary Table S18. Linear regression—forced-entry multivariate models for predicting serum cytokine levels (full description of model); Supplementary Table S19. Linear regression—stepwise multivariate models for predicting serum cytokine levels; Supplementary Section S4.1. Overview of the Main Biological Pathways Involved in Cytokine and Chemokine Production and Their Association with Dietary Influences; Supplementary Section S4.2. Overview of the Potential Mechanistic Aspects Underlying Associations of Specific Dietary Factors with Cytokine/Chemokine Levels; Supplementary Section S4.3. Overview of the Biological Roles of the Analyzed Cytokines and Chemokines [255,256,257,258,259,260,261,262,263,264,265,266,267,268,269,270,271,272,273,274,275,276,277,278,279,280,281,282,283,284,285,286,287,288,289,290,291,292,293,294,295,296,297,298,299,300,301,302,303,304,305,306,307,308,309,310,311,312,313,314,315,316,317,318,319,320,321,322,323,324,325,326,327,328,329,330,331,332,333,334,335,336,337,338,339,340,341,342,343,344,345,346,347,348,349,350,351,352,353,354,355,356,357,358,359,360,361,362,363,364,365,366,367,368,369,370,371,372,373,374,375,376,377,378,379,380,381,382,383,384,385,386,387,388,389,390,391,392,393,394,395,396,397,398,399,400,401,402,403,404,405,406,407,408,409,410,411,412,413,414,415,416,417,418,419,420,421,422,423,424,425,426,427,428,429,430,431,432,433,434,435,436,437,438,439,440].

## Abbreviations

The following abbreviations are used in this manuscript:

2-AG	2-arachidonoylglycerol
4E-BP1	Eukaryotic translation initiation factor 4E-binding protein 1
AEA	Anandamide (N-arachidonoylethanolamine)
AGE	Advanced glycation end product
AhR	Aryl hydrocarbon receptor
ALA	α-linolenic acid
ALP	Alkaline phosphatase
ALT	Alanine aminotransferase
AMPK	Adenosine monophosphate-activated protein kinase
ANCOVA	Analysis of covariance
AP-1	Activator protein 1
AST	Aspartate aminotransferase
BCR	B cell receptor
BMI	Body mass index
C/EBP	CCAAT/enhancer-binding protein
cAMP	Cyclic AMP
CB1/CB2	Cannabinoid receptor type 1 or type 2
CCL	C-C motif chemokine ligand
CCR2	C-C chemokine receptor type 2
CD36	Cluster of differentiation 36
c-Jun/c-Fos	Jun proto-oncogene/Fos proto-oncogene
COX	Cyclooxygenase
cPLA2	Cytosolic phospholipase A2
CRP	C-reactive protein
CV	Coefficient of variation
CXCL	C-X-C motif chemokine ligand
DAG	Diacylglycerol
DAMPs	Danger-associated signals
DAP	Diet Assess & Plan
DCs	Dendritic cells
DHA	Docosahexaenoic acid
DHEA	Docosahexaenoyl ethanolamide
DII	Dietary Inflammatory Index
DIT	Diet-induced thermogenesis
E-DII	Energy-adjusted Dietary Inflammatory Index
EFSA	European Food Safety Authority
EPA	Eicosapentaenoic acid
EPEA	Eicosapentaenoyl ethanolamide
ER	Endoplasmic reticulum
ERK	Extracellular signal–regulated kinase
ESR	Erythrocyte sedimentation rate
EuroFIR	European Food Information Resource Network
FFAR	Free fatty acid receptor
FOXO	Forkhead box O
FOXP3	Forkhead box P3
FPQ	Food Propensity Questionnaire
GCL	Glutamate cysteine ligase
GGT	Gamma-glutamyl transferase
GM-CS	Granulocyte–macrophage colony-stimulating factor
GPR	G protein-coupled receptor
GPX	Glutathione peroxidase
GR	Glutathione reductase
Grx	Glutaredoxin
GSH	Glutathione
GSK3β	Glycogen synthase kinase 3 beta
GST	Glutathione S-transferase
HDACs	Histone deacetylases
HDL	High-density lipoprotein
HETEs	Hydroxyeicosatetraenoic acids
HIF-1α	Hypoxia-inducible factor 1 alpha
HO-1	Heme oxygenase-1
HOTrE	Hydroxy-octadecatrienoic acids
HpOTrE	Hydroperoxy-octadecatrienoic acids
IFN	Interferon
IFNAR	Interferon-α/β receptor
IFNGR	IFN-γ receptor
IGF-1	Insulin-like growth factor 1
IKKβ	IκB kinase β
IL	Interleukin
IL-18BP	IL-18 binding protein
IL-1R	IL-1 receptor
IL-1RA	IL-1 receptor antagonist
IL-1RL1	IL-1 receptor-like 1
ILC2s	Innate lymphoid cells group 2
iNOS	Inducible nitric oxide synthase
IQR	Interquartile range
IRF	Interferon regulatory factor
IκB	Inhibitor of nuclear factor kappa B
JAK	Janus kinase
JNK	c-Jun N-terminal kinase
LDL	Low-density lipoprotein
LOX	Lipooxigenase
LPS	Lipopolysaccharide
LTB_4_	Leukotriene B4
MAPK	Mitogen-activated protein kinase
MCH	Mean corpuscular hemoglobin
MCHC	Mean corpuscular hemoglobin concentration
MCP-1	Monocyte chemoattractant protein-1
MCV	Mean corpuscular volume
mTOR	Mammalian target of rapamycin
MyD88	Myeloid differentiation primary response 88
NE	Niacin equivalents
NF-κB	Nuclear factor kappa B
NK	Natural killer
NLRP3	NOD-, LRR-, and pyrin domain-containing protein 3
NQO1	NAD(P)H quinone oxidoreductase 1
oxLDL	Oxidized LDL
pDCs	plasmacytoid dendritic cells
PE	Phycoerythrin
PGE2	Prostaglandin E2
PI3K–Akt	Phosphatidylinositol-3 kinase–protein kinase B (PKB/Akt)
PKB	Protein kinase B (Akt)
PKC	Protein kinase C
PLC	Phospholipase C
PUFA	Polyunsaturated fatty acids
RAE	Retinol activity equivalents
RE	Retinol equivalents
REE	Resting energy expenditure
ROS	Reactive oxygen species
S6K1	S6 kinase 1
SCFAs	Short-chain fatty acids
SFA	Saturated fatty acids
sIL-1R	Soluble IL-1 receptor
SIRT1	Sirtuin 1
SOD	Superoxide dismutase
SPMs	Specialized pro-resolving mediators
SPSS	Statistical Package for the Social Sciences
STAT	Signal transducer and activator of transcription
TAK1	Transforming growth factor-β (TGF-β)-activated kinase 1
TCR	T-cell receptor
TEF	Thermic effect of food
TGF	Transforming growth factor
Th	T helper
TLR	Toll-like receptor
TNF	Tumor necrosis factor
TNFR	Tumor necrosis factor receptor
TRAF	TNF receptor-associated factor
TRAF3-IP2	TRAF3-interacting protein 2
TRE	TPA (12-O-tetradecanoylphorbol-13-acetate) response elements
Treg	T regulatory
TXA_2_	Thromboxane A2
TXN	Thioredoxin
TXNIP	Thioredoxin-interacting protein
TXNRD	Thioredoxin reductase
TYK2	Tyrosine kinase 2
WHR	Waist-to-hip ratio
WHtR	Waist-to-height ratio
