# Supplementary material for: Differences in Serum Cytokine Profiles Between Vegetarians and Omnivores: Exploratory Assessment of Dietary Factors
Source: Metabolites. 2026 Oct 19;16(10):793. doi: 10.3390/metabo16100793 (PMC13707265; doi:10.3390/metabo16100793)
Supplement: Supplementary file 1 [file metabolites-16-00793-s001.zip › metabolites-4515719-supplementary.pdf]

metabolites

MDPI

Article

Differences in Serum Cytokine Profiles Between Vegetarians and Omnivores: Exploratory Assessment of Dietary Factors

Supplementary material

Supplementary Section S2.1.1. The study timeline

Timeline of the study:

- **2017:** Ethical approval obtained for the project "Support to National Dietary Surveys in Compliance with the EU Menu methodology (sixth support): The adults' survey, including subjects from 10 to 74 years old", including blood sampling and laboratory analyses.
- **Late 2019:** Participant recruitment initiated (screening; no sample collection).
- **2020–2021:** Recruitment and sample collection temporarily interrupted due to the COVID-19 pandemic.
- **2021–2022:** Recruitment and biological sample collection resumed and were completed.
- **2019–2022:** Routine biochemical and hematological analyses were performed on the day of blood collection. The first 24-hour dietary recall, general data questionnaire, and anthropometric assessment were conducted on the same day. The second 24-hour recall was performed within one month after blood collection and at least 7 days after the first recall.
- **Early 2023:** Cytokine laboratory analyses were performed using samples stored at −80°C; no multiple freeze-thaw cycles in the meantime.
- **Early 2026:** CRP analyses were performed using samples stored at −80°C; no multiple freeze-thaw cycles in the meantime.
- **2023–2026:** Completion and updating of the Serbian Food Composition Database, assignment of macro- and micronutrient values to newly reported foods, dietary intake calculations,
- **2026:** Integration of dietary and laboratory data, and final statistical analyses.

Participant recruitment and eligibility flow

VEGETARIAN CANDIDATES [n = 102]	OMNIVORE CANDIDATES [n = 103]	TOTAL CANDIDATES SCREENED [n = 205]
↓	↓	↓
DIETARY ELIGIBILITY<br>≥2 years vegetarian diet<br>• Lacto-ovo: no meat/fish<br>• Vegan: no animal-derived foods<br><br>Eligible: [n = 99]<br>Excluded: [n = 3]<br>Reason(s): <2 years vegetarian diet (3)	DIETARY ELIGIBILITY<br>≥2 years regular meat intake<br>• Meat/meat products ≥1–2×/week<br><br>Eligible: [n = 102]<br>Excluded: [n = 1]<br>Reason(s): <1–2×/week meat intake (1)	DIETARY GROUP ELIGIBILITY<br><br>Eligible: [n = 201]<br>Excluded: [n = 4]
↓	↓	↓
ADDITIONAL INCLUSION CRITERIA<br>• Age 25–50 years<br>• BMI 18.5–25 kg/m^2<br>• Residence in Belgrade area<br><br>Eligible: [n = 91]<br>Excluded: [n = 8]<br>Reason(s): age (3); BMI (4); residence (1);	ADDITIONAL INCLUSION CRITERIA<br>• Age 25–50 years<br>• BMI 18.5–25 kg/m^2<br>• Residence in Belgrade area<br><br>Eligible: [n = 94]<br>Excluded: [n = 8]<br>Reason(s): age (4); BMI (4);	ADDITIONAL ELIGIBILITY<br><br>Eligible: [n = 185]<br>Excluded: [n = 16]
↓	↓	↓
EXCLUSION CRITERIA<br>1. Inflammatory/autoimmune disease<br>Excluded: [n = 0]<br>2. Allergies<br>Excluded: [n = 1]<br>3. Other conditions affecting immune status<br>Excluded: [n = 2]<br>4. Immune-modulating medication<br>Excluded: [n = 0]<br>5. Pescatarian diet<br>Excluded: [n = 1]<br>6. Implausible energy intake<br>Excluded: [n = 3]<br>7. Very high physical activity<br>Excluded: [n = 1]<br>8. Did not complete all analyses<br>Excluded: [n = 3]<br><br>Excluded total: [n = 11]	EXCLUSION CRITERIA<br>1. Inflammatory/autoimmune disease<br>Excluded: [n = 1]<br>2. Allergies<br>Excluded: [n = 2]<br>3. Other conditions affecting immune status<br>Excluded: [n = 2]<br>4. Immune-modulating medication<br>Excluded: [n = 1]<br>5. Pescatarian diet<br>Excluded: [n = 0]<br>6. Implausible energy intake<br>Excluded: [n = 3]<br>7. Very high physical activity<br>Excluded: [n = 2]<br>8. Did not complete all analyses<br>Excluded: [n = 3]<br><br>Excluded total: [n = 14]	EXCLUSION SCREENING<br><br>Excluded: [n = 25]
↓	↓	↓
**FINAL VEGETARIAN SAMPLE**<br>**[n = 80]**<br>**• Vegans: n = 67**<br>**• Lacto-ovo: n = 13**	**FINAL OMNIVORE SAMPLE**<br>**[n = 80]**	**FINAL ANALYTIC SAMPLE**<br>**[n = 160]**<br>**Vegetarians + omnivores**

Implausible energy-intake criterion: EI/EER < 0.616 or > 1.625; EER calculated using the Mifflin–St Jeor equation and PAL = 1.4, 1.6, or 1.8 for sedentary, moderately active, or active participants, respectively.

Supplementary Figure S1. Flow diagram of the study participants' selection. BMI = body mass index, EI = energy intake, EER = estimated energy requirement, PAL= physical activity level.

Supplementary Table S1. Analytical characteristics and quantification limits of the cytokine/chemokine assay.

Cytokine/chemokine	LOD (pg/mL)	LLOQ (pg/mL)	Percentage of samples below LLOQ			ULOQ
			Total	Vegetarian	Omnivorous	
IL-1β (pg/mL)	0.80	1.45	11.3%	17.5%	5.0%	11000
IFN-α2 (pg/ml)	1.29	1.78	3.1%	6.3%	0.0%	13000
IFN-γ (pg/mL)	0.57	1.04	8.1%	12.5%	3.8%	8000
TNF-α (pg/mL)	0.64	1.31	4.4%	6.3%	2.5%	10500
MCP-1 (pg/mL)	0.83	2.11	0.0%	0.0%	0.0%	10000
IL-6 (pg/mL)	1.00	2.12	6.9%	10.0%	3.8%	16600
IL-8 (pg/mL)	0.48	1.11	12.5%	21.3%	3.8%	9000
IL-10 (pg/mL)	0.82	1.54	10.0%	13.8%	6.3%	11500
IL-12p70 (pg/mL)	0.84	1.67	8.8%	13.8%	3.8%	13000
IL-17A (pg/mL)	0.22	0.45	17.5%	28.8%	6.3%	3500
IL-18 (pg/mL)	1.75	3.43	0.0%	0.0%	0.0%	19000
IL-23 (pg/mL)	1.48	1.92	18.1%	25.0%	11.3%	15000
IL-33 (pg/mL)	8.16	12.40	7.5%	12.5%	2.5%	100000

IL = interleukin, IFN = interferon, TNF = tumor necrosis factor, MCP = monocyte chemoattractant protein, LOD = lower limit of detection, predicted concentration of blank + 3 x SD, LLOQ = lower limit of quantification, predicted concentration of blank + 10 x SD, ULOQ = upper limit of quantification (concentration of the highest standard).

Supplementary Table S2. Subgroup comparison of socioeconomic, lifestyle, and anthropometric data between participants following vegan vs. lacto-ovo vegetarian vs. omnivorous diets.

	**Vegan diet (*n*=67)**		**Lacto-ovo vegetarian diet (*n*=13)**		**Vegan vs. Omnivorous**	**Lacto-Ovo vs. Omnivorous**	**Vegan vs. Lacto-Ovo**
	***n*/Median**	**(%)/(IQR)**	***n*/Median**	**(%)/(IQR)**	***p***	***p***	***p***
Sex:							
• Men, *n* (%)	33	(49.3%)	8	(61.5%)	0.951	0.392	0.417
• Women, *n* (%)	34	(50.7%)	5	(38.5%)			
Age (years)	34.0	(28.0-40.0)	32.0	(28.5-36.5)	0.885	0.790	0.592
Smoking status:							
• Never smoker, *n* (%)	39	(58.2%)	8	(61.5%)	0.233	0.464	0.906
• Former smoker, *n* (%)	20	(29.2%)	4	(30.8%)			
• Current smoker, *n* (%)	8	(11.9%)	1	(7.7%)			
Physical activity level:							
• Low, *n* (%)	23	(34.3%)	3	(23.1%)	0.464	0.969	0.712
• Moderate, *n* (%)	28	(41.8%)	6	(46.2%)			
• High, *n* (%)	16	(23.9%)	4	(30.8%)			
Height (cm)	174.0	(167.0-181.0)	178.0	(171.5-183.5)	0.196	0.698	0.194
Weight (kg)	66.0	(58.0-78.0)	72.0	(62.5-80.5)	0.291	0.824	0.354
BMI (kg/m^2)	80.0	(73.0-87.0)	81.0	(74.0-88.5)	0.398	0.996	0.638
Waist circumference (cm)	94.0	(89.0-100.0)	95.0	(90.0-103.0)	0.161	0.621	0.875
Hip circumference (cm)	22.2	(20.1-24.4)	22.5	(20.4-24.6)	0.608	0.929	0.686
WHR	0.9	(0.8-0.9)	0.8	(0.8-0.9)	0.700	0.837	0.901
WHtR	0.5	(0.4-0.5)	0.5	(0.4-0.5)	0.824	0.794	0.901

BMI = body mass index, WHR = waist-to-hip ratio, WHtR = waist-to-height ratio, IQR = interquartile range, *p* = statistical significance of the difference for the Mann-Whitney test. Exploratory sensitivity analyses. Nominal *p*-values are reported. Bold values indicate statistical significance at $p < 0.05$. No correction for multiple testing.

Supplementary Table S3. Subgroup comparison of standard biochemistry serum metabolite levels between participants following vegan vs. lacto-ovo vegetarian vs. omnivorous diets.

	Vegan diet (n=67)		Lacto-ovo vegetarian diet (n=13)		Vegan vs. Omnivorous	Lacto-Ovo vs. Omnivorous	Vegan vs. Lacto-Ovo
**Biochemistry metabolite**	**Median**	**(IQR)**	**Median**	**(IQR)**	***p***	***p***	***p***
Glucose (mmol/L)	4.8	(4.5–5.1)	4.8	(4.2–5.5)	**0.044**	0.466	0.900
Total cholesterol (mmol/L)	3.9	(3.4–4.5)	4.2	(3.8–4.5)	**<0.001**	**0.003**	0.186
HDL-cholesterol (mmol/L)	1.4	(1.2–1.6)	1.7	(1.5–1.8)	**<0.001**	0.510	**0.006**
LDL-cholesterol (mmol/L)	2.0	(1.6–2.6)	2.2	(2.0–2.4)	**<0.001**	**0.003**	0.449
Triglycerides (mmol/L)	0.8	(0.6–1.0)	0.6	(0.5–0.8)	0.634	**0.047**	**0.035**
Urea (mmol/L)	3.5	(2.8–4.2)	3.6	(2.8–4.7)	**<0.001**	**0.036**	0.845
Creatinine (μmol/L)	73.0	(64.0–79.0)	70.0	(60.5–79.0)	**0.041**	0.151	0.657
Total bilirubin (μmol/L)	11.4	(8.3–15.5)	14.1	(10.5–19.3)	0.978	0.195	0.171
Uric acid (μmol/L)	278.0	(222.0–331.0)	278.0	(231.0–350.0)	0.328	0.265	0.566
Total protein (g/L)	71.6	(69.3–74.9)	72.6	(71.0–74.7)	0.791	0.535	0.461
Serum Fe (μmol/L)	19.4	(13.9–24.5)	21.1	(19.6–30.5)	0.893	0.064	0.070
ALP (U/L)	66.0	(54.0–78.0)	52.0	(40.0–71.5)	**0.004**	0.169	**0.019**
ALT (U/L)	15.5	(11.7–21.4)	15.6	(12.5–22.3)	0.416	0.682	0.907
AST (U/L)	17.8	(15.1–22.4)	19.9	(16.0–22.2)	0.425	0.833	0.544
GGT (U/L)	12.0	(9.0–15.0)	11.0	(8.5–15.0)	0.066	0.088	0.464

HDL = high density lipoprotein, LDL = low density lipoprotein, ALP = alkaline phosphatase, ALT = alanine aminotransferase, AST = aspartate aminotransferase, GGT = γ-glutamyl transferase, IQR = interquartile range, p = statistical significance of the difference for the Mann-Whitney test. Exploratory sensitivity analyses. Nominal p-values are reported. Bold values indicate statistical significance at $p < 0.05$. No correction for multiple testing.

Supplementary Table S4. Subgroup comparison of standard hematological parameters and serum CRP levels between participants following vegan vs. lacto-ovo vegetarian vs. omnivorous diets.

	**Vegan diet (*n*=67)**		**Lacto-ovo vegetarian diet (*n*=13)**		**Vegan vs. Omnivorous**	**Lacto-Ovo vs. Omnivorous**	**Vegan vs. Lacto-Ovo**
**Hematological parameter**	**Median**	**(IQR)**	**Median**	**(IQR)**	***p***	***p***	***p***
Erythrocyte count (x10¹²/L)	4.7	(4.4–5.0)	4.7	(4.5–5.0)	0.124	0.634	0.566
Hemoglobin (g/L)	142.0	(135.0–153.1)	143.7	(135.0–153.0)	0.188	0.455	0.964
Hematocrit (L/L)	0.4	(0.4–0.5)	0.4	(0.4–0.5)	0.620	0.769	0.938
MCV (fL)	92.4	(90.0–95.0)	90.5	(88.7–95.9)	**0.005**	0.583	0.502
MCH (pg)	30.9	(29.8–32.1)	30.5	(29.5–31.6)	0.362	0.674	0.485
MCHC (g/L)	332.0	(327.6–337.0)	331.0	(326.1–334.0)	**<0.001**	**0.004**	0.457
Platelet count (x10¹²/L)	255.0	(201.8–292.8)	198.0	(178.0–259.5)	0.218	0.422	0.103
Leukocyte count (x10⁹/L)	5.33	(4.86-6.45)	5.15	(4.71-6.80)	**0.007**	0.088	0.611
Neutrophil count (x10⁹/L)	3.08	(2.57-3.81)	2.71	(2.53-3.57)	0.176	0.185	0.523
Lymphocyte count (x10⁹/L)	1.78	(1.47-2.01)	1.97	(1.40-2.49)	**0.002**	0.682	0.291
Monocyte count (x10⁹/L)	0.44	(0.39-0.53)	0.40	(0.30-0.50)	0.454	0.061	0.179
Eosinophil count (x10⁹/L)	0.12	(0.06-0.22)	0.10	(0.08-0.20)	0.082	0.159	0.845
Basophil count (x10¹²/L)	0.03	(0.02-0.04)	0.03	(0.02-0.04)	0.493	0.738	0.930
CRP	1.4	(1.2–1.8)	1.5	(1.1–2.7)	0.997	0.955	0.949

MCV = mean corpuscular volume, MCH = mean corpuscular hemoglobin, MCHC = mean corpuscular hemoglobin concentration, CRP = C-reactive protein, IQR = interquartile range, p = statistical significance of the difference for the Mann-Whitney test. Exploratory sensitivity analyses. Nominal p-values are reported. Bold values indicate statistical significance at $p < 0.05$. No correction for multiple testing.

Supplementary Table S5. Subgroup comparison of serum cytokine/chemokine levels between participants following vegan vs. lacto-ovo vegetarian vs. omnivorous diets.

	**Vegan diet (*n*=67)**		**Lacto-ovo vegetarian diet (*n*=13)**		**Vegan vs. Omnivorous**	**Lacto-Ovo vs. Omnivorous**	**Vegan vs. Lacto-Ovo**
**Cytokine / Chemokine**	**Median**	**(IQR)**	**Median**	**(IQR)**	***p***	***p***	***p***
IL-1β (pg/mL)	43.4	(3.3–145.4)	21.7	(0.8–132.2)	0.638	0.407	0.560
IFN-α2(pg/ml)	5.5	(2.5–11.1)	5.4	(2.4–16.9)	**0.042**	0.325	0.908
IFN-γ (pg/mL)	10.4	(2.6–24.0)	5.9	(1.4–9.6)	**0.027**	**0.006**	0.172
TNF-α (pg/mL)	14.3	(3.6–46.6)	12.7	(5.7–21.9)	0.064	**0.015**	0.436
MCP-1 (pg/mL)	319.3	(225.9–409.6)	275.9	(204.8–309.7)	0.199	0.650	0.199
IL-6 (pg/mL)	8.1	(3.3–21.0)	6.8	(3.3–15.3)	**0.038**	0.063	0.590
IL-8 (pg/mL)	27.2	(1.9–137.9)	13.9	(0.6–55.8)	**0.008**	**0.004**	0.330
IL-10 (pg/mL)	7.1	(2.4–15.5)	7.9	(2.2–17.1)	**0.029**	0.263	0.902
IL-12p70 (pg/mL)	5.3	(2.1–13.0)	3.3	(0.8–12.4)	**0.025**	**0.036**	0.280
IL-17A (pg/mL)	1.1	(0.2–2.7)	0.2	(0.2–1.7)	**0.002**	**0.004**	0.145
IL-18 (pg/mL)	266.1	(168.9–342.6)	229.6	(166.9–353.5)	0.251	0.430	0.816
IL-23 (pg/mL)	12.3	(0.9–23.1)	5.4	(1.3–13.3)	0.143	0.102	0.382
IL-33 (pg/mL)	54.6	(17.4–159.6)	83.9	(15.7–252.6)	0.108	0.746	0.603

IL = interleukin, IFN = interferon, TNF = tumor necrosis factor, MCP = monocyte chemoattractant protein, IQR = interquartile range, p = statistical significance of the difference for the Mann- Whitney test. Exploratory sensitivity analyses. Nominal p-values are reported. Bold values indicate statistical significance at $p < 0.05$. No correction for multiple testing.

Supplementary Table S6. Comparison of serum cytokine levels between participants following vegetarian vs. omnivorous diets when originally measured analytical values below LLOQ were included in analysis.

	**Vegetarian diet (n=80)**		**Omnivorous diet (n=80)**		**Mann-Whitney**	**Quade's ANCOVA**
**Cytokine/chemokine**	**Median**	**(IQR)**	**Median**	**(IQR)**	***p***	***p′***
IL-1β (pg/mL)	41.6	(2.9–141.6)	45.6	(14.9–106.9)	0.520	0.469
IFN-α2 (pg/ml)	5.5	(2.5–11.7)	8.1	(3.5–15.6)	**0.036**	**0.041**
IFN-γ (pg/mL)	8.5	(2.6–23.3)	15.5	(8.2–25.3)	**0.006***	**0.006***
TNF-α (pg/mL)	13.8	(4.0–44.3)	26.4	(12.3–53.6)	**0.020***	**0.030**
MCP-1 (pg/mL)	308.5	(221.7–399.9)	273.8	(187.6–375.0)	0.316	0.359
IL-6 (pg/mL)	7.7	(3.3–20.9)	14.0	(5.6–27.9)	**0.020***	**0.028**
IL-8 (pg/mL)	24.7	(1.9–120.8)	75.8	(34.2–169.6)	**0.004***	**0.005***
IL-10 (pg/mL)	7.1	(2.4–15.5)	10.3	(4.4–26.0)	**0.026***	**0.026**
IL-12p70 (pg/mL)	5.3	(1.9–13.0)	8.8	(3.9–17.0)	**0.010***	**0.009***
IL-17A (pg/mL)	1.0	(0.3–2.6)	2.1	(0.9–4.2)	**<0.001***	**<0.001***
IL-18 (pg/mL)	261.4	(168.9–342.6)	268.7	(180.1–391.2)	0.197	0.175
IL-23 (pg/mL)	11.6	(1.1–21.4)	13.1	(4.3–36.5)	0.074	0.077
IL-33 (pg/mL)	54.6	(17.4–208.1)	81.3	(32.7–217.0)	0.128	0.128

LLOQ = lower limit of quantification, IL = interleukin, IFN = interferon, TNF = tumor necrosis factor, MCP = monocyte chemoattractant protein, IQR = interquartile range, p = statistical significance of the difference for the Mann-Whitney test, p' = statistical significance of the difference for Quade's non-parametric analysis of covariance (ANCOVA), with adjustment for sex, age, BMI, smoking, and physical activity level. Exploratory sensitivity analyses. Nominal p-values are reported. Bold values indicate statistical significance at $p < 0.05$. Values marked with an asterisk (*) remained statistically significant after Benjamini–Hochberg false discovery rate (FDR) correction for 13 cytokine/chemokine multiple comparisons.

Supplementary Table S7. Comparison of serum cytokine levels between participants following vegetarian vs. omnivorous diets in a subset of participants after removal of those with statistical upper-tail outlier/extreme values.

	Vegetarian diet (*n*=42)		Omnivorous diet (*n*=40)		Mann-Whitney	Quade's ANCOVA
**Cytokine/chemokine**	**Median**	**(IQR)**	**Median**	**(IQR)**	***p***	***p′***
IL-1β (pg/mL)	14.8	(0.5-58.5)	23.9	(6.5-56.4)	0.186	0.143
IFN-α2 (pg/ml)	2.2	(1.3-4.9)	6.1	(2.3-8.9)	**<0.001***	**<0.001***
IFN-γ (pg/mL)	4.0	(1.1-9.5)	12.3	(6.3-17.1)	**0.001***	**<0.001***
TNF-α (pg/mL)	7.6	(2.7-13.7)	19.0	(6.8-29.3)	**0.001***	**0.002***
MCP-1 (pg/mL)	265.9	(165.5-334.9)	252.7	(170.5-339.9)	0.817	0.848
IL-6 (pg/mL)	4.2	(1.0-8.3)	13.2	(3.2-20.1)	**0.002***	**0.004***
IL-8 (pg/mL)	8.1	(0.9-27.6)	42.0	(11.7-81.0)	**<0.001***	**0.001***
IL-10 (pg/mL)	3.6	(0.9-7.9)	6.9	(3.1-16.3)	**0.007***	**0.004***
IL-12p70 (pg/mL)	2.5	(0.9-4.8)	5.8	(2.6-11.2)	**0.002***	**<0.001***
IL-17A (pg/mL)	0.4	(0.2-1.2)	1.3	(0.6-2.3)	**<0.001***	**<0.001***
IL-18 (pg/mL)	199.3	(152.2-297.8)	250.4	(165.8-354.6)	0.164	0.223
IL-23 (pg/mL)	3.1	(0.0-10.6)	11.3	(2.7-29.0)	**<0.001***	**0.001***
IL-33 (pg/mL)	18.0	(6.1-47.9)	49.5	(28.6-97.0)	**0.001***	**<0.001***

IL = interleukin, IFN = interferon, TNF = tumor necrosis factor, MCP = monocyte chemoattractant protein, IQR = interquartile range, p = statistical significance of the difference for the Mann-Whitney test, p' = statistical significance of the difference for Quade's non-parametric analysis of covariance (ANCOVA), with adjustment for sex, age, BMI, smoking, and physical activity level. Exploratory sensitivity analyses. Nominal p-values are reported. Bold values indicate statistical significance at $p < 0.05$. Values marked with an asterisk (*) remained statistically significant after Benjamini–Hochberg false discovery rate (FDR) correction for 13 cytokine/chemokine multiple comparisons.

Supplementary Table S8. Within-person intercorrelation between different serum cytokines/chemokines (all dietary groups merged).

		IL-1β	IFN-α2	IFN-γ	TNF-α	MCP-1	IL-6	IL-8	IL-10	IL-12p70	IL-17A	IL-18	IL-23	IL-33
**IL-1β**	r_s	-	**0.405**	**0.356**	**0.361**	0.121	**0.330**	**0.248**	**0.425**	**0.414**	**0.456**	**0.272**	**0.352**	**0.423**
	p		**<0.0001*** **	**<0.0001*** **	**<0.0001*** **	0.135	**<0.0001*** **	**0.002***	**<0.0001*** **	**<0.0001*** **	**<0.0001*** **	**<0.001** **	**<0.0001*** **	**<0.0001*** **
**IFN-α2**	r_s	**0.405**	-	**0.636**	**0.631**	**0.237**	**0.619**	**0.471**	**0.594**	**0.655**	**0.527**	**0.402**	**0.608**	**0.900**
	p	**<0.0001*** **		**<0.0001*** **	**<0.0001*** **	**0.003***	**<0.0001*** **	**<0.0001*** **	**<0.0001*** **	**<0.0001*** **	**<0.0001*** **	**<0.0001*** **	**<0.0001*** **	**<0.0001*** **
**IFN-γ**	r_s	**0.356**	**0.636**	-	**0.582**	0.049	**0.531**	**0.448**	**0.452**	**0.576**	**0.568**	**0.278**	**0.614**	**0.528**
	p	**<0.0001*** **	**<0.0001*** **		**<0.0001*** **	0.543	**<0.0001*** **	**<0.0001*** **	**<0.0001*** **	**<0.0001*** **	**<0.0001*** **	**<0.001** **	**<0.0001*** **	**<0.0001*** **
**TNF-α**	r_s	**0.361**	**0.631**	**0.582**	-	**0.183**	**0.584**	**0.565**	**0.491**	**0.533**	**0.563**	**0.362**	**0.469**	**0.522**
	p	**<0.0001*** **	**<0.0001*** **	**<0.0001*** **		**0.023***	**<0.0001*** **	**<0.0001*** **	**<0.0001*** **	**<0.0001*** **	**<0.0001*** **	**<0.0001*** **	**<0.0001*** **	**<0.0001*** **
**MCP-1**	r_s	0.121	**0.237**	0.049	**0.183**	-	**0.265**	**0.434**	**0.255**	**0.303**	**0.206**	**0.327**	0.107	**0.188**
	p	0.135	**0.003***	0.543	**0.023***		**<0.001** **	**<0.0001*** **	**0.001***	**<0.001** **	**0.010***	**<0.0001*** **	0.185	**0.020***
**IL-6**	r_s	**0.330**	**0.619**	**0.531**	**0.584**	**0.265**	-	**0.644**	**0.686**	**0.685**	**0.538**	**0.371**	**0.508**	**0.555**
	p	**<0.0001*** **	**<0.0001*** **	**<0.0001*** **	**<0.0001*** **	**<0.001** **		**<0.0001*** **	**<0.0001*** **	**<0.0001*** **	**<0.0001*** **	**<0.0001*** **	**<0.0001*** **	**<0.0001*** **
**IL-8**	r_s	**0.248**	**0.471**	**0.448**	**0.565**	**0.434**	**0.644**	-	**0.419**	**0.559**	**0.615**	**0.398**	**0.421**	**0.399**
	p	**0.002***	**<0.0001*** **	**<0.0001*** **	**<0.0001*** **	**<0.0001*** **	**<0.0001*** **		**<0.0001*** **	**<0.0001*** **	**<0.0001*** **	**<0.0001*** **	**<0.0001*** **	**<0.0001*** **
**IL-10**	r_s	**0.425**	**0.594**	**0.452**	**0.491**	**0.255**	**0.686**	**0.419**	-	**0.712**	**0.482**	**0.271**	**0.494**	**0.523**
	p	**<0.0001*** **	**<0.0001*** **	**<0.0001*** **	**<0.0001*** **	**0.001***	**<0.0001*** **	**<0.0001*** **		**<0.0001*** **	**<0.0001*** **	**0.001**	**<0.0001*** **	**<0.0001*** **
**IL-12p70**	r_s	**0.414**	**0.655**	**0.576**	**0.533**	**0.303**	**0.685**	**0.559**	**0.712**	-	**0.619**	**0.442**	**0.608**	**0.627**
	p	**<0.0001*** **	**<0.0001*** **	**<0.0001*** **	**<0.0001*** **	**<0.0001*** **	**<0.0001*** **	**<0.0001*** **	**<0.0001*** **		**<0.0001*** **	**<0.0001*** **	**<0.0001*** **	**<0.0001*** **
**IL-17A**	r_s	**0.456**	**0.527**	**0.568**	**0.563**	**0.206**	**0.538**	**0.615**	**0.482**	**0.619**	-	**0.403**	**0.528**	**0.456**
	p	**<0.0001*** **	**<0.0001*** **	**<0.0001*** **	**<0.0001*** **	**0.010***	**<0.0001*** **	**<0.0001*** **	**<0.0001*** **	**<0.0001*** **		**<0.0001*** **	**<0.0001*** **	**<0.0001*** **
**IL-18**	r_s	**0.272**	**0.402**	**0.278**	**0.362**	**0.327**	**0.371**	**0.398**	**0.271**	**0.442**	**0.403**	-	**0.267**	**0.393**
	p	**<0.001** **	**<0.0001*** **	**<0.001** **	**<0.0001*** **	**<0.0001*** **	**<0.0001*** **	**<0.0001*** **	**<0.001** **	**<0.0001*** **	**<0.0001*** **		**<0.001** **	**<0.0001*** **
**IL-23**	r_s	**0.352**	**0.608**	**0.614**	**0.469**	0.107	**0.508**	**0.421**	**0.494**	**0.608**	**0.528**	**0.267**	-	**0.586**
	p	**<0.0001*** **	**<0.0001*** **	**<0.0001*** **	**<0.0001*** **	0.185	**<0.0001*** **	**<0.0001*** **	**<0.0001*** **	**<0.0001*** **	**<0.0001*** **	**<0.001** **		**<0.0001*** **
**IL-33**	r_s	**0.423**	**0.900**	**0.528**	**0.522**	**0.188**	**0.555**	**0.399**	**0.523**	**0.627**	**0.456**	**0.393**	**0.586**	-
	p	**<0.0001*** **	**<0.0001*** **	**<0.0001*** **	**<0.0001*** **	**0.020***	**<0.0001*** **	**<0.0001*** **	**<0.0001*** **	**<0.0001*** **	**<0.0001*** **	**<0.0001*** **	**<0.0001*** **	

IL = interleukin, IFN = interferon, TNF = tumor necrosis factor, MCP = monocyte chemoattractant protein, r_s = Spearman's rank correlation coefficients, p = statistical significance of correlations. Exploratory analyses. Nominal p-values are reported. Bold values indicate statistical significance at * $p < 0.05$, ** $p < 0.001$, *** $p < 0.0001$. All bolded p-values labelled with * remained significant after Benjamini–Hochberg false discovery rate (FDR) correction for 78 cytokine/chemokine intercorrelations presented.

Supplementary Table S9. Subgroup comparison of energy intakes coming from different food groups between participants following vegan vs. lacto-ovo vegetarian vs. omnivorous diets.

	**Vegan diet (*n*=67)**		**Lacto-ovo vegetarian diet (*n*=13)**		**Vegan vs. Omnivorous**	**Lacto-Ovo vs. Omnivorous**	**Vegan vs. Lacto-Ovo**
**Food groups' energy contribution**	**Median**	**(IQR)**	**Median**	**(IQR)**	***p***	***p***	***p***
Energy from milk products (kcal/day)	0.0	(0.0–0.0)	133.2	(16.9–245.7)	**<0.001**	**0.037**	**<0.001**
Energy from egg products (kcal/day)	0.0	(0.0–0.0)	42.5	(0.0–66.5)	**<0.001**	**0.026**	**0.002**
Energy from meat products (kcal/day)	0.0	(0.0–0.0)	0.0	(0.0–0.0)	**<0.001**	**<0.001**	0.660
Energy from fish products (kcal/day)	0.0	(0.0–0.0)	0.0	(0.0–0.0)	**<0.001**	**0.005**	0.531
Energy from edible fats/oils (kcal/day)	344.2	(196.3–516.7)	473.8	(193.6–565.2)	**0.017**	0.666	0.372
Energy from grain products (kcal/day)	581.5	(327.8–773.5)	532.2	(370.4–747.0)	0.964	0.801	0.901
Energy from nuts/seeds (kcal/day)	252.5	(135.2–549.8)	369.7	(114.5–507.5)	**<0.001**	**0.001**	0.477
Energy from vegetable products (kcal/day)	434.1	(292.0–590.2)	293.8	(207.9–369.2)	**<0.001**	**0.004**	**0.007**
Energy from fruit products (kcal/day)	288.3	(150.6–462.7)	219.3	(141.2–367.5)	**<0.001**	**0.001**	0.318
Energy from sugar products (kcal/day)	24.1	(0.0–141.8)	69.9	(22.6–232.0)	**<0.001**	0.388	0.162
Energy from beverages (kcal/day)	2.5	(0.0–82.6)	18.8	(0.0–84.7)	0.605	0.849	0.863
% Energy from milk products (%kcal/day)	0.0	(0.0–0.0)	7.0	(0.7–9.5)	**<0.001**	**0.037**	**<0.001**
% Energy from egg products (%kcal/day)	0.0	(0.0–0.0)	1.4	(0.0–2.9)	**<0.001**	**0.040**	**<0.001**
% Energy from meat products (%kcal/day)	0.0	(0.0–0.0)	0.0	(0.0–0.0)	**<0.001**	**<0.001**	0.660
% Energy from fish products (%kcal/day)	0.0	(0.0–0.0)	0.0	(0.0–0.0)	**<0.001**	**<0.001**	0.531
% Energy from edible fats/oils (%kcal/day)	15.2	(8.5–20.1)	17.6	(9.6–27.6)	0.230	0.987	0.720
% Energy from grain products (%kcal/day)	26.4	(15.4–32.5)	23.2	(17.7–30.5)	0.189	0.519	0.779
% Energy from nuts/seeds (%kcal/day)	12.5	(4.8–21.4)	13.6	(4.6–22.8)	**<0.001**	**0.001**	0.819
% Energy from vegetable products (%kcal/day)	19.5	(12.1–27.5)	12.2	(8.0–17.9)	**<0.001**	**0.004**	**0.007**
% Energy from fruit products (%kcal/day)	13.1	(6.3–22.5)	8.7	(6.5–15.1)	**<0.001**	**<0.001**	0.129
% Energy from sugar products (%kcal/day)	1.2	(0.0–7.0)	3.2	(0.8–11.6)	**<0.001**	0.560	0.143
% Energy from beverages (%kcal/day)	0.1	(0.0–3.7)	0.9	(0.0–3.9)	0.867	0.936	0.826

IQR = interquartile range, p = statistical significance of the difference for the Mann-Whitney test. Exploratory sensitivity analyses. Nominal p-values are reported. Bold values indicate statistical significance at $p < 0.05$. No correction for multiple testing.

Supplementary Table 10. Subgroup **c**omparison of dietary macronutrient intakes between participants following vegan vs. lacto-ovo vegetarian vs. omnivorous diets.

	**Vegan diet (n=67)**		**Lacto-ovo vegetarian diet (n=13)**		**Vegan vs. Omnivorous**	**Lacto-Ovo vs. Omnivorous**	**Vegan vs. Lacto-Ovo**
**Macronutrient and its energy contribution**	**Median**	**(IQR)**	**Median**	**(IQR)**	***p***	***p***	***p***
Energy intake (kcal/day)	2227.3	(1940.5–2632.0)	2466.3	(1904.6–2736.3)	**0.005**	0.344	0.562
Energy intake per kg body mass (kcal/kg/day)	33.0	(29.4–39.9)	32.1	(29.2–41.0)	**0.014**	0.140	0.789
Protein intake (g/day)	66.1	(51.3–82.3)	71.0	(46.8–90.0)	**<0.001**	**0.001**	0.536
Carbohydrate intake (g/day)	254.2	(212.9–321.0)	211.4	(178.7–250.1)	**<0.001**	0.610	**0.044**
Fat intake (g/day)	87.6	(72.0–111.6)	136.6	(81.7–167.4)	**<0.001**	0.413	**0.006**
Saturated fat intake (g/day)	17.6	(11.6–22.6)	24.2	(17.0–41.4)	**<0.001**	**0.009**	**0.012**
Monounsaturated fat intake (g/day)	35.4	(27.2–46.8)	37.5	(32.7–54.1)	**<0.001**	0.109	0.137
Polyunsaturated fat intake (g/day)	26.5	(22.1–36.7)	28.3	(21.1–49.2)	**<0.001**	0.443	0.238
n-6 polyunsaturated fat intake (g/day)	19.2	(14.0–29.6)	22.6	(17.8–41.2)	**<0.001**	0.443	0.144
n-3 polyunsaturated fat intake (g/day)	1.7	(0.9–2.9)	1.1	(0.6–1.6)	0.783	0.062	0.123
n-6/n-3 polyunsaturated fat intake ratio	11.9	(6.3–22.2)	19.1	(13.4–46.4)	**0.001**	0.251	**0.017**
Trans-fat intake (g/day)	0.2	(0.0–0.8)	0.5	(0.2–1.1)	**<0.001**	**0.037**	0.213
Cholesterol intake (mg/day)	1.8	(0.0–15.4)	158.1	(13.0–346.4)	**<0.001**	**0.001**	**<0.001**
Fiber intake (g/day)	45.6	(35.5–62.9)	33.9	(29.3–43.3)	**<0.001**	**<0.001**	**0.006**
Alcohol intake (g/day)	0.0	(0.0–0.1)	0.0	(0.0–5.5)	0.475	0.652	0.374
% Energy from protein (%kcal/day)	11.6	(9.3–14.5)	10.8	(10.1–13.3)	**<0.001**	**<0.001**	0.964
% Energy from carbohydrate (%kcal/day)	46.3	(41.4–52.1)	38.0	(33.4–41.3)	**<0.001**	**0.038**	**<0.001**
% Energy from fat (%kcal/day)	35.7	(31.8–41.1)	45.4	(39.5–50.0)	**<0.001**	0.255	**<0.001**
% Energy from saturated fat (%kcal/day)	7.0	(5.2–9.1)	10.0	(7.9–13.5)	**<0.001**	**0.003**	**0.004**
% Energy from monounsaturated fat (%kcal/day)	14.1	(11.1–17.1)	15.6	(13.6–19.8)	**<0.001**	0.602	0.186
% Energy from polyunsaturated fat (%kcal/day)	11.6	(9.2–13.8)	13.2	(9.8–16.7)	**0.027**	0.978	0.181
% Energy from n-6 polyunsaturated fat (%kcal/day)	8.5	(5.9–10.9)	9.0	(7.6–14.2)	**<0.001**	0.642	0.228
% Energy from n-3 polyunsaturated fat (%kcal/day)	0.6	(0.4–1.0)	0.4	(0.2–0.6)	0.105	0.215	0.091
% Energy from trans-fat (%kcal/day)	0.1	(0.0–0.3)	0.2	(0.0–0.5)	**<0.001**	0.057	0.267
% Energy from fiber (%kcal/day)	4.0	(3.6–4.8)	2.9	(2.4–3.4)	**<0.001**	**<0.001**	**<0.001**
% Energy from alcohol (%kcal/day)	0.0	(0.0–0.0)	0.0	(0.0–1.7)	0.542	0.606	0.374

IQR = interquartile range, p = statistical significance of the difference for the Mann-Whitney test. Exploratory sensitivity analyses. Nominal p-values are reported. Bold values indicate statistical significance at $p < 0.05$. No correction for multiple testing.

Supplementary Table S11. Comparison of dietary micronutrient intakes per 1000 kcal between participants following vegetarian vs. omnivorous diets.

	**Vegetarian diet (*n*=80)**		**Omnivorous diet (*n*=80)**		**Mann-Whitney**	**Quade's ANCOVA**
**Micronutrient**	**Median**	**(IQR)**	**Median**	**(IQR)**	***p***	***p′***
Fe (mg/1000 kcal/day)	8.3	(6.8–10.0)	4.7	(5.6–6.4)	**<0.001***	**<0.001***
Zn (mg/1000 kcal/day)	4.7	(4.1–5.9)	4.0	(4.8–5.6)	0.834	0.827
Cu (mg/1000 kcal/day)	1.2	(1.0–1.4)	0.5	(0.6–0.8)	**<0.001***	**<0.001***
I (μg/1000 kcal/day)	57.9	(39.2–68.0)	69.6	(79.4–93.4)	**<0.001***	**<0.001***
Se (μg/1000 kcal/day)	10.1	(8.4–13.3)	16.5	(21.6–29.1)	**<0.001***	**<0.001***
Ca (mg/1000 kcal/day)	326.4	(250.1–409.7)	248.2	(303.1–379.4)	0.385	0.363
P (mg/1000 kcal/day)	578.2	(474.0–700.1)	549.8	(632.7–720.7)	**0.014***	**0.013***
Mg (mg/1000 kcal/day)	269.1	(220.9–324.3)	116.5	(154.7–193.4)	**<0.001***	**<0.001***
K (mg/1000 kcal/day)	1888.3	(1579.3–2202.7)	1085.7	(1284.7–1571.2)	**<0.001***	**<0.001***
Na (mg/1000 kcal/day)	1204.4	(914.4–1522.4)	1265.8	(1478.2–1736.2)	**<0.001***	**<0.001***
NaCl (mg/1000 kcal/day)	1809.8	(1151.1–2474.4)	1352.4	(1848.5–2255.6)	0.992	0.982
β-carotene (μg/1000 kcal/day)	3079.3	(1422.8–5066.5)	636.1	(1012.3–2398.7)	**<0.001***	**<0.001***
Retinol (μg/1000 kcal/day)	2.0	(0.0–15.4)	93.0	(124.9–181.2)	**<0.001***	**<0.001***
Vitamin A RE (μg/1000 kcal/day)	548.9	(275.7–855.9)	225.7	(342.3–581.0)	**0.015***	**0.008***
Vitamin A RAE (μg/1000 kcal/day)	274.5	(147.1–442.4)	172.9	(247.4–360.1)	0.659	0.559
Vitamin B1 (mg/1000 kcal/day)	0.9	(0.7–1.6)	0.6	(0.7–1.1)	**0.019***	**0.020***
Vitamin B2 (mg/1000 kcal/day)	0.6	(0.5–0.8)	0.6	(0.7–0.9)	**0.019***	**0.022***
Vitamin B3 NE (mg/1000 kcal/day)	12.2	(9.4–15.0)	13.5	(16.6–19.9)	**<0.001***	**<0.001***
Vitamin B5 (mg/1000 kcal/day)	3.0	(2.3–4.2)	2.3	(3.0–3.8)	0.617	0.554
Vitamin B6 (mg/1000 kcal/day)	1.0	(0.8–1.3)	0.8	(1.0–1.2)	0.395	0.352
Vitamin B9 (μg/1000 kcal/day)	240.8	(199.7–283.4)	100.0	(126.2–161.6)	**<0.001***	**<0.001***
Vitamin B12 (μg/1000 kcal/day)	0.4	(0.1–0.7)	1.5	(2.1–3.1)	**<0.001***	**<0.001***
Vitamin C (mg/1000 kcal/day)	97.2	(60.5–149.1)	24.7	(43.9–67.6)	**<0.001***	**<0.001***
Vitamin D (mg/1000 kcal/day)	0.1	(0.0–0.8)	1.1	(1.8–3.5)	**<0.001***	**<0.001***
Vitamin E (mg/1000 kcal/day)	13.2	(9.2–15.2)	9.0	(11.1–14.6)	0.137	0.147
Polyphenols (mg/1000 kcal/day)	673.8	(470.1–986.5)	183.8	(306.3–487.5)	**<0.001***	**<0.001***

RE = retinol equivalents, RAE = retinol activity equivalents, NE = niacin equivalents, IQR = interquartile range, p = statistical significance of the difference for Mann- Whitney test, p' = statistical significance of the difference for Quade's non-parametric analysis of covariance (ANCOVA), with adjustment for sex, age, BMI, smoking, and physical activity level. Exploratory sensitivity analyses. Nominal p-values are reported. Bold values indicate statistical significance at $p < 0.05$. Values marked with an asterisk (*) remained statistically significant after Benjamini–Hochberg false discovery rate (FDR) correction for 76 dietary multiple comparisons.

Supplementary Table S12. Subgroup comparison of dietary micronutrient intakes between participants following vegan vs. lacto-ovo vegetarian vs. omnivorous diets.

	Vegan diet (n=67)		Lacto-ovo vegetarian diet (n=13)		Vegan vs. Omnivorous	Lacto-Ovo vs. Omnivorous	Vegan vs. Lacto-Ovo
**Micronutrient / index**	**Median**	**(IQR)**	**Median**	**(IQR)**	p	p	p
Fe (mg/day)	19.8	(14.6–23.8)	15.2	(12.6–19.9)	**<0.001**	0.324	0.100
Zn (mg/day)	11.2	(8.7–14.2)	11.2	(8.0–13.3)	**0.040**	0.156	0.764
Cu (mg/day)	2.9	(2.3–3.7)	2.6	(1.6–3.3)	**<0.001**	**0.004**	0.245
I (μg/day)	124.2	(97.3–145.8)	173.5	(111.2–229.1)	**<0.001**	0.070	**0.023**
Se (μg/day)	23.5	(17.1–29.6)	35.1	(19.6–41.4)	**<0.001**	**<0.001**	0.054
Ca (mg/day)	696.7	(545.2–841.6)	877.5	(666.2–1344.2)	**0.034**	0.233	**0.018**
P (mg/day)	1318.0	(995.9–1624.8)	1433.9	(1032.2–1751.9)	**<0.001**	0.067	0.502
Mg (mg/day)	641.1	(500.8–783.8)	538.5	(383.5–713.3)	**<0.001**	**0.002**	0.150
K (mg/day)	4286.2	(3336.2–5461.0)	3679.1	(3447.2–4621.7)	**<0.001**	0.074	0.248
Na (mg/day)	2638.6	(2035.5–3288.8)	3487.5	(1984.4–4069.9)	**<0.001**	**0.043**	0.325
NaCl (mg/day)	4397.8	(2449.2–5659.9)	4114.1	(3084.8–5343.2)	0.113	0.519	0.799
β-carotene (μg/day)	7834.8	(3529.5–11216.0)	5639.9	(2266.8–9116.8)	**<0.001**	0.069	0.223
Retinol (μg/day)	0.0	(0.0–20.3)	136.5	(80.6–320.2)	**<0.001**	**0.003**	**<0.001**
Vitamin A RE (μg/day)	1338.1	(588.2–1934.7)	1156.3	(538.8–1798.8)	0.078	0.587	0.700
Vitamin A RAE (μg/day)	672.0	(294.1–985.4)	713.1	(333.5–1077.1)	0.557	0.969	0.769
Vitamin B1 (mg/day)	2.1	(1.6–4.1)	1.9	(1.3–4.1)	0.247	0.840	0.477
Vitamin B2 (mg/day)	1.4	(1.1–1.8)	1.7	(1.2–2.1)	**<0.001**	0.259	0.269
Vitamin B3 NE (mg/day)	27.1	(22.0–39.6)	24.7	(21.4–33.3)	**<0.001**	**<0.001**	0.494
Vitamin B5 (mg/day)	6.6	(5.2–11.3)	5.1	(4.3–6.8)	0.696	**0.001**	**0.025**
Vitamin B6 (mg/day)	2.3	(1.7–3.0)	2.1	(1.7–2.3)	0.428	**0.046**	0.217
Vitamin B9 (μg/day)	563.4	(439.6–750.1)	457.2	(416.3–611.0)	**<0.001**	**<0.001**	0.213
Vitamin B12 (μg/day)	0.7	(0.3–1.3)	2.0	(0.7–3.3)	**<0.001**	**<0.001**	**0.010**
Vitamin C (mg/day)	205.2	(151.5–328.9)	214.0	(133.7–278.7)	**<0.001**	**0.003**	0.681
Vitamin D (mg/day)	0.3	(0.0–1.6)	2.1	(0.3–5.3)	**<0.001**	**0.003**	**0.004**
Vitamin E (mg/day)	26.4	(20.1–33.5)	31.6	(24.4–43.3)	0.181	0.211	0.055
Polyphenols (mg/day)	1589.6	(1133.4–2241.7)	1161.1	(1010.1–1781.4)	**<0.001**	**0.003**	0.125

Supplementary Table S12. - continued

Fe (mg/1000 kcal/day)	8.5	(7.1–10.3)	7.1	(5.8–7.9)	**<0.001**	**0.005**	**0.007**
Zn (mg/1000 kcal/day)	4.8	(4.1–6.0)	4.3	(3.9–5.2)	0.568	0.344	0.233
Cu (mg/1000 kcal/day)	1.3	(1.0–1.5)	1.0	(0.8–1.1)	**<0.001**	**<0.001**	**0.009**
I (µg/1000 kcal/day)	56.5	(38.3–64.9)	68.1	(53.9–87.3)	**<0.001**	0.053	**0.021**
Se (µg/1000 kcal/day)	9.8	(8.2–11.8)	11.5	(10.6–16.0)	**<0.001**	**<0.001**	0.053
Ca (mg/1000 kcal/day)	310.8	(243.7–382.4)	410.8	(336.6–470.6)	0.926	**0.010**	**0.023**
P (mg/1000 kcal/day)	576.7	(472.5–712.0)	583.0	(488.4–681.5)	**0.021**	0.166	0.901
Mg (mg/1000 kcal/day)	277.6	(229.0–331.9)	218.3	(187.7–288.2)	**<0.001**	**<0.001**	**0.020**
K (mg/1000 kcal/day)	1951.3	(1594.3–2264.4)	1530.5	(1284.7–1947.6)	**<0.001**	**0.019**	**0.028**
Na (mg/1000 kcal/day)	1191.2	(866.6–1505.8)	1251.7	(915.1–1623.5)	**<0.001**	0.060	0.662
NaCl (mg/1000 kcal/day)	1817.4	(1076.2–2545.5)	1711.8	(1180.2–2360.2)	0.942	0.862	0.974
β-carotene (µg/1000 kcal/day)	3317.2	(1635.5–5151.5)	2151.2	(1071.2–4269.9)	**<0.001**	**0.039**	0.177
Retinol (µg/1000 kcal/day)	0.0	(0.0–8.7)	74.9	(33.3–119.4)	**<0.001**	**0.005**	**<0.001**
Vitamin A RE (µg/1000 kcal/day)	552.9	(273.4–858.6)	401.8	(277.0–813.4)	**0.012**	0.463	0.519
Vitamin A RAE (µg/1000 kcal/day)	276.4	(144.1–446.8)	212.9	(177.1–462.3)	0.659	0.862	0.984
Vitamin B1 (mg/1000 kcal/day)	1.0	(0.7–1.4)	0.7	(0.5–2.0)	**0.006**	0.801	0.190
Vitamin B2 (mg/1000 kcal/day)	0.6	(0.5–0.8)	0.6	(0.5–0.8)	**0.023**	0.251	0.871
Vitamin B3 NE (mg/1000 kcal/day)	12.3	(10.0–15.0)	10.9	(8.7–15.4)	**<0.001**	**0.002**	0.173
Vitamin B5 (mg/1000 kcal/day)	3.2	(2.4–4.5)	2.3	(2.0–2.6)	0.120	**0.005**	**0.001**
Vitamin B6 (mg/1000 kcal/day)	1.0	(0.8–1.3)	0.8	(0.7–1.0)	0.141	0.153	**0.048**
Vitamin B9 (µg/1000 kcal/day)	248.7	(202.6–288.5)	221.5	(153.8–265.3)	**<0.001**	**<0.001**	0.094
Vitamin B12 (µg/1000 kcal/day)	0.3	(0.1–0.5)	0.9	(0.3–1.4)	**<0.001**	**<0.001**	**0.009**
Vitamin C (mg/1000 kcal/day)	98.6	(60.6–158.9)	86.8	(43.6–112.2)	**<0.001**	**0.002**	0.358
Vitamin D (mg/1000 kcal/day)	0.1	(0.0–0.7)	1.1	(0.1–2.1)	**<0.001**	**0.009**	**0.004**
Vitamin E (mg/1000 kcal/day)	12.4	(9.1–14.7)	16.3	(10.5–17.5)	0.321	**0.044**	0.071
Polyphenols (mg/1000 kcal/day)	703.6	(483.7–1096.9)	475.3	(424.3–680.6)	**<0.001**	**0.002**	**0.028**
DII score	-2.8	(-3.7– -1.9)	-2.1	(-3.1– -1.4)	**<0.001**	0.055	0.116
E-DII score	-2.6	(-3.4– -1.7)	-1.8	(-2.5– -0.2)	**<0.001**	**0.013**	**0.021**

RE = retinol equivalents, RAE = retinol activity equivalents, NE = niacin equivalents, DII = Dietary inflammatory index, E-DII = Energy-adjusted dietary inflammatory index, IQR = interquartile range, p = statistical significance of the difference for the Mann-Whitney test. Exploratory sensitivity analyses. Nominal p-values are reported. Bold values indicate statistical significance at $p < 0.05$. No correction for multiple testing.

Supplementary Table S13. Comparison of frequency of supplement use between participants following vegetarian and omnivorous diets.

**Supplement**	**Vegetarian diet (*n*=80)** ≥1 time per week		**Omnivorous diet (*n*=80)** ≥1 time per week		**Pearson's chi-square/ Fisher's exact**
	n	***(%)***	***n***	***(%)***	***p***
Vitamin A	0	(0.0%)	1	(1.3%)	1.000
Beta-carotene	0	(0.0%)	1	(1.3%)	1.000
Folic acid	4	(5.0%)	3	(3.8%)	1.000
Vitamin B12	16	(20.0%)	0	(0.0%)	**<0.001***
B-complex	1	(1.3%)	0	(0.0%)	1.000
Vitamin D	8	(10.0%)	18	(22.5%)	**0.032**
Vitamin E	1	(1.3%)	4	(5.0%)	0.367
Vitamin C	8	(10.0%)	19	(23.8%)	**0.020**
Calcium	1	(1.3%)	1	(1.3%)	1.000
Magnesium	11	(13.8%)	8	(10.0%)	0.463
Iron	1	(1.3%)	2	(2.5%)	1.000
Zinc	2	(2.5%)	11	(13.8%)	**0.017**
Selenium	1	(1.3%)	10	(12.5%)	**0.009**
Multivitamin without minerals	0	(0.0%)	2	(2.5%)	0.497
Multivitamin with minerals	1	(1.3%)	5	(6.3%)	0.210
Fish oil, n-3 fatty acids	1	(1.3%)	8	(10.0%)	**0.034**
Herbal dietary supplements	3	(3.8%)	3	(3.8%)	1.000
Algae-based dietary supplements	9	(11.3%)	2	(2.5%)	0.056
Other supplements	2	(2.5%)	2	(2.5%)	1.000

p = statistical significance of the difference for Fisher's exact test or Pearson's chi-square test, as appropriate. Exploratory analyses; nominal p-values are reported. Bold values indicate statistical significance at $p < 0.05$. Values marked with an asterisk (*) remained statistically significant after Benjamini–Hochberg false discovery rate (FDR) correction for 19 supplement-related multiple comparisons.

Supplementary Table S14. Reported supplement use according to 24-h recalls among participants following vegetarian and omnivorous diets.

Supplement	Vegetarian diet (*n*=80)		Omnivorous diet (*n*=80)	
	n (%)	Reported dose	*n* (%)	Reported dose
Folic acid	—	—	1 (1.3%)	5 mg
Vitamin B12	3 (3.8%)	1000 µg	—	—
B-complex	1 (1.3%)	B1 10 mg, B2 10 mg, B3 48 mg, B5 30 mg, B6 10 mg	—	—
Vitamin D	2 (2.5%)	10–25 µg	1 (1.3%)	12.5 µg
Vitamin E	—	—	0 (0%)	—
Vitamin C	4 (5.0%)	500–1000 mg	1 (1.3%)	300 mg
Calcium	1 (1.3%)	800 mg	0 (0%)	—
Magnesium	3 (3.8%)	100–400 mg	2 (2.5%)	300–375 mg
Zinc	1 (1.3%)	5 mg	2 (2.5%)	7–10 mg
Selenium	—	—	1 (1.3%)	30 µg
Multivitamin with minerals	1 (1.3%)	Centrum "From A to Z"®	—	—
Herbal dietary supplements	1 (1.3%)	Maka 5 g	—	—
Algae-based dietary supplements	2 (2.5%)	500 mg	1 (1.3%)	500 mg
Brewer's yeast	1 (1.3%)	10 g	0 (0%)	—

Supplement use was assessed from the two 24-h dietary recalls, and reported doses represent estimated daily doses."—" indicates no reported use.

Supplementary Table S15. Correlation of serum cytokines/chemokines with energy intake from different food groups and macronutrient intakes in a subset of 82 participants (comprising 42 vegetarians and 40 omnivores) after removal of those with statistical upper-tail outlier/extreme values.

		IL-1β	IFN-α2	IFN-γ	TNF-α	MCP-1	IL-6	IL-8	IL-10	IL-12p70	IL-17A	IL-18	IL-23	IL-33
Milk products	r_s	0.210	**0.254**	**0.270**	**0.254**	-0.046	0.141	**0.291**	0.164	0.114	**0.297**	0.177	**0.318**	**0.228**
(kcal/day)	p	0.058	**0.021***	**0.014***	**0.021***	0.679	0.208	**0.008*†**	0.140	0.310	**0.007*†**	0.112	**0.004*†**	**0.040***
Egg products	r_s	0.020	**0.224**	**0.297**	0.163	0.000	**0.228**	**0.241**	0.151	0.075	0.111	0.089	**0.282**	**0.258**
(kcal/day)	p	0.855	**0.043***	**0.007*†**	0.144	0.998	**0.040***	**0.030***	0.174	0.501	0.321	0.425	**0.010***	**0.019***
Meat products	r_s	0.088	**0.309**	**0.284**	**0.296**	-0.025	**0.323**	**0.444**	0.216	**0.263**	**0.329**	0.193	**0.374**	**0.251**
(kcal/day)	p	0.434	**0.005*†**	**0.010***	**0.007*†**	0.827	**0.003*†**	**<0.001**†**	0.051	**0.017***	**0.003*†**	0.083	**<0.001**†**	**0.023***
Fish products	r_s	0.005	**0.359**	**0.243**	**0.340**	0.004	**0.251**	**0.366**	0.210	0.202	**0.260**	-0.031	0.144	**0.275**
(kcal/day)	p	0.963	**<0.001**†**	**0.028***	**0.002*†**	0.973	**0.024***	**<0.001**†**	0.058	0.069	**0.018***	0.784	0.196	**0.012***
Edible fats/oils	r_s	0.205	**0.265**	0.215	0.133	**0.259**	**0.261**	**0.224**	0.155	**0.268**	**0.252**	**0.345**	**0.273**	0.169
(kcal/day)	p	0.065	**0.016***	0.052	0.233	**0.019***	**0.019***	**0.043***	0.165	**0.015***	**0.022***	**0.002*†**	**0.013***	0.129
Grain products	r_s	0.070	-0.011	0.121	-0.040	0.047	0.047	0.089	-0.091	0.099	0.081	0.164	**0.232**	-0.117
(kcal/day)	p	0.535	0.924	0.279	0.722	0.674	0.678	0.425	0.414	0.376	0.469	0.140	**0.036***	0.294
Nuts/seeds	r_s	**-0.323**	**-0.393**	**-0.282**	**-0.272**	0.139	-0.218	**-0.240**	-0.206	-0.198	**-0.241**	**-0.233**	**-0.339**	**-0.375**
(kcal/day)	p	**0.003*†**	**<0.001**†**	**0.010***	**0.013***	0.212	0.051	**0.030***	0.064	0.075	**0.029***	**0.035***	**0.002*†**	**<0.001**†**
Vegetable products	r_s	**-0.263**	**-0.350**	-0.185	**-0.277**	0.098	-0.172	**-0.289**	**-0.240**	-0.126	**-0.268**	-0.104	-0.211	**-0.348**
(kcal/day)	p	**0.017***	**0.001*†**	0.096	**0.012***	0.380	0.125	**0.008*†**	**0.030***	0.260	**0.015***	0.355	0.057	**0.001*†**
Fruit products	r_s	-0.150	**-0.282**	**-0.254**	**-0.227**	0.037	-0.216	**-0.298**	-0.149	-0.167	**-0.301**	**-0.274**	**-0.323**	-0.154
(kcal/day)	p	0.179	**0.010***	**0.021***	**0.040***	0.738	0.053	**0.006*†**	0.181	0.133	**0.006*†**	**0.013***	**0.003*†**	0.167
Sugar products	r_s	0.017	0.084	0.205	0.208	-0.117	**0.263**	0.184	**0.218**	0.162	**0.274**	-0.004	0.169	0.043
(kcal/day)	p	0.880	0.452	0.065	0.061	0.296	**0.018***	0.097	**0.049***	0.145	**0.013***	0.972	0.128	0.698
Beverages	r_s	0.193	0.034	0.175	0.113	0.198	0.191	-0.012	0.044	0.127	**0.239**	0.202	0.092	-0.049
(kcal/day)	p	0.082	0.760	0.115	0.311	0.074	0.088	0.912	0.697	0.255	**0.030***	0.069	0.410	0.664
Energy intake	r_s	-0.171	-0.056	0.160	0.061	0.199	**0.311**	**0.226**	0.100	0.195	0.142	0.120	**0.229**	-0.177
(kcal/day)	p	0.124	0.616	0.151	0.583	0.074	**0.005*†**	**0.041***	0.373	0.078	0.204	0.284	**0.038***	0.111
Protein intake	r_s	-0.101	0.176	**0.321**	0.213	-0.039	**0.312**	**0.344**	0.098	0.185	**0.244**	0.151	**0.390**	0.063
(g/day)	p	0.369	0.113	**0.003*†**	0.054	0.727	**0.005*†**	**0.002*†**	0.382	0.096	**0.027***	0.176	**<0.001**†**	0.571
Carbohydrate	r_s	-0.116	**-0.249**	-0.032	-0.111	0.050	0.093	-0.015	-0.085	-0.013	-0.045	-0.027	-0.016	**-0.307**
intake (g/day)	p	0.298	**0.024***	0.773	0.323	0.656	0.409	0.894	0.449	0.905	0.687	0.812	0.884	**0.005*†**
Fat intake	r_s	-0.111	0.130	0.199	0.206	0.204	**0.328**	**0.303**	0.177	0.211	**0.234**	0.167	**0.277**	0.020
(g/day)	p	0.322	0.245	0.073	0.063	0.067	**0.003*†**	**0.006*†**	0.112	0.057	**0.034***	0.134	**0.012***	0.856
Saturated fat intake	r_s	0.100	**0.286**	**0.335**	**0.288**	0.057	**0.328**	**0.361**	0.185	**0.314**	**0.373**	0.190	**0.398**	**0.222**
(g/day)	p	0.372	**0.009***	**0.002*†**	**0.009*†**	0.611	**0.003*†**	**<0.001**†**	0.097	**0.004*†**	**<0.001**†**	0.088	**<0.001**†**	**0.045***

Supplementary Table S15. ***(continued)***

		IL-1β	IFN-α2	IFN-γ	TNF-α	MCP-1	IL-6	IL-8	IL-10	IL-12p70	IL-17A	IL-18	IL-23	IL-33
Monounsaturated fat intake (g/day)	r_s	-0.111	0.094	0.172	0.079	**0.282**	**0.343**	**0.272**	0.096	0.183	0.189	0.190	0.205	0.032
	p	0.321	0.400	0.122	0.482	**0.010***	**0.002*†**	**0.014***	0.391	0.101	0.090	0.088	0.065	0.773
Polyunsaturated fat intake (g/day)	r_s	-0.093	**0.233**	0.184	**0.230**	0.150	**0.283**	**0.219**	**0.219**	**0.227**	**0.258**	0.156	**0.231**	0.040
	p	0.405	**0.035***	0.098	**0.038***	0.179	**0.011***	**0.048***	**0.049***	**0.040***	**0.019***	0.162	**0.037***	0.721
n-6 fat intake (g/day)	r_s	0.035	**0.232**	**0.231**	0.213	0.217	**0.338**	**0.298**	0.155	0.210	**0.269**	**0.264**	**0.271**	0.073
	p	0.758	**0.036***	**0.036***	0.055	0.050	**0.002*†**	**0.007*†**	0.165	0.058	**0.014***	**0.016***	**0.014***	0.516
n-3 fat intake (g/day)	r_s	-0.211	-0.146	-0.081	-0.163	-0.134	-0.125	-0.186	-0.076	-0.147	-0.134	-0.111	**-0.278**	-0.207
	p	0.058	0.190	0.472	0.144	0.230	0.267	0.095	0.498	0.186	0.231	0.323	**0.011***	0.062
n-6/n-3 fat intake ratio	r_s	0.163	0.186	0.155	0.203	0.205	**0.245**	**0.303**	0.108	0.174	0.209	**0.239**	**0.328**	0.128
	p	0.144	0.094	0.165	0.068	0.064	**0.028***	**0.006*†**	0.333	0.118	0.060	**0.030***	**0.003*†**	0.253
Trans-fat intake (g/day)	r_s	-0.048	0.093	0.113	0.145	-0.044	0.113	0.129	0.147	0.180	0.039	0.126	**0.245**	0.016
	p	0.667	0.407	0.314	0.194	0.697	0.314	0.249	0.187	0.106	0.730	0.260	**0.026**	0.884
Cholesterol intake (mg/day)	r_s	0.097	**0.360**	**0.338**	**0.343**	0.003	**0.379**	**0.447**	**0.272**	**0.242**	**0.290**	0.175	**0.410**	**0.296**
	p	0.388	**<0.001**†**	**0.002*†**	**0.002*†**	0.976	**<0.001**†**	**<0.001**†**	**0.013***	**0.028***	**0.008*†**	0.116	**<0.001**†**	**0.007*†**
Fiber intake (g/day)	r_s	**-0.335**	**-0.407**	**-0.246**	**-0.381**	0.050	**-0.251**	**-0.358**	**-0.282**	-0.163	**-0.360**	-0.213	**-0.385**	**-0.372**
	p	**0.002*†**	**<0.001**†**	**0.026***	**<0.001**†**	0.657	**0.024***	**<0.001**†**	**0.010***	0.144	**<0.001**†**	0.055	**<0.001**†**	**<0.001**†**
DII score	r_s	0.205	0.167	0.024	0.191	-0.112	0.053	0.191	0.117	-0.024	0.215	0.100	0.214	0.109
	p	0.064	0.134	0.834	0.085	0.318	0.641	0.085	0.296	0.832	0.053	0.371	0.054	0.329
E-DII score	r_s	0.073	0.146	0.109	**0.226**	-0.030	0.193	**0.298**	0.111	0.029	**0.261**	0.145	**0.343**	0.064
	p	0.513	0.192	0.331	**0.041***	0.789	0.084	**0.006*†**	0.319	0.796	**0.018***	0.193	**0.002*†**	0.567

IL = interleukin, IFN = interferon, TNF = tumor necrosis factor, MCP = monocyte chemoattractant protein, DII = dietary inflammatory index, E-DII = energy-adjusted dietary inflammatory index, r_s = Spearman's correlation coefficients, p = statistical significance of correlations. Exploratory sensitivity analyses. Nominal p-values are reported. Bold values indicate statistical significance at * $p < 0.05$, ** $p < 0.001$. Values marked with a dagger (†) remained significant after Benjamini–Hochberg false discovery rate (FDR) correction for 13 cytokine/chemokine and 26 dietary variables multiple correlations.

Supplementary Table S16. Correlation of serum cytokines/chemokines with energy intake from different food groups and macronutrient intakes in a subset of 82 participants (comprising 42 vegetarians and 40 omnivores) after removal of those with statistical upper-tail outlier/extreme values, with adjustment for potential confounders.

		**IL-1β**	**IFN-α2**	**IFN-γ**	**TNF-α**	**MCP-1**	**IL-6**	**IL-8**	**IL-10**	**IL-12p70**	**IL-17A**	**IL-18**	**IL-23**	**IL-33**
Milk products	r_s	**0.234**	**0.274**	**0.297**	**0.262**	-0.059	0.118	**0.259**	0.200	0.150	**0.354**	0.138	**0.291**	**0.261**
(kcal/day)	p	**0.041***	**0.016***	**0.009*†**	**0.021***	0.610	0.310	**0.023***	0.082	0.192	**0.002*†**	0.233	**0.010*†**	**0.022***
Egg products	r_s	-0.009	**0.227**	**0.313**	0.150	0.008	0.209	0.211	0.173	0.094	0.165	0.060	**0.254**	**0.251**
(kcal/day)	p	0.938	**0.047***	**0.006*†**	0.193	0.945	0.070	0.066	0.133	0.415	0.151	0.606	**0.026***	**0.027***
Meat products	r_s	0.130	**0.347**	**0.333**	**0.307**	-0.049	**0.306**	**0.417**	**0.260**	**0.326**	**0.401**	0.148	**0.351**	**0.309**
(kcal/day)	p	0.260	**0.002*†**	**0.003*†**	**0.007*†**	0.675	**0.007*†**	**<0.001**†**	**0.022***	**0.004*†**	**<0.001**†**	0.200	**0.002*†**	**0.006*†**
Fish products	r_s	0.045	**0.374**	**0.260**	**0.337**	0.032	**0.258**	**0.396**	0.219	0.211	**0.266**	-0.004	0.154	**0.310**
(kcal/day)	p	0.700	**<0.001**†**	**0.022***	**0.003*†**	0.785	**0.024***	**<0.001**†**	0.056	0.066	**0.019***	0.972	0.180	**0.006*†**
Edible fats/oils	r_s	0.217	**0.306**	**0.248**	0.215	**0.245**	**0.309**	**0.235**	**0.240**	**0.362**	**0.327**	**0.277**	**0.288**	**0.243**
(kcal/day)	p	0.058	**0.007*†**	**0.030***	0.060	**0.032***	**0.007*†**	**0.040***	**0.036***	**0.001*†**	**0.004*†**	**0.015*†**	**0.011*†**	**0.034***
Grain products	r_s	0.158	0.025	0.190	-0.074	0.016	-0.013	0.005	-0.068	0.178	0.179	0.107	0.200	-0.085
(kcal/day)	p	0.169	0.832	0.098	0.525	0.887	0.912	0.966	0.557	0.122	0.119	0.355	0.081	0.461
Nuts/seeds	r_s	**-0.304**	**-0.398**	**-0.280**	**-0.279**	0.137	-0.212	-0.223	**-0.230**	-0.211	**-0.276**	-0.222	**-0.316**	**-0.377**
(kcal/day)	p	**0.007*†**	**<0.001**†**	**0.014*†**	**0.014*†**	0.234	0.066	0.052	**0.044***	0.066	**0.015*†**	0.053	**0.005*†**	**<0.001**†**
Vegetable products	r_s	**-0.296**	**-0.373**	-0.198	**-0.297**	0.131	-0.166	**-0.278**	**-0.251**	-0.130	**-0.261**	-0.089	-0.195	**-0.392**
(kcal/day)	p	**0.009*†**	**<0.001**†**	0.085	**0.009*†**	0.257	0.153	**0.014*†**	**0.027***	0.259	**0.022***	0.441	0.090	**<0.001**†**
Fruit products	r_s	-0.157	**-0.313**	**-0.284**	-0.224	-0.022	-0.204	**-0.283**	-0.215	**-0.225**	**-0.394**	**-0.279**	**-0.298**	-0.190
(kcal/day)	p	0.172	**0.006*†**	**0.012*†**	0.050	0.850	0.077	**0.013*†**	0.061	**0.049***	**<0.001**†**	**0.014*†**	**0.008*†**	0.098
Sugar products	r_s	0.021	0.084	**0.227**	0.158	-0.016	**0.252**	0.171	**0.269**	0.195	**0.360**	0.030	0.137	0.038
(kcal/day)	p	0.857	0.466	**0.047***	0.170	0.888	**0.028***	0.138	**0.018***	0.090	**0.001*†**	0.795	0.236	0.744
Beverages	r_s	0.075	-0.007	0.126	0.170	**0.243**	**0.249**	0.013	0.064	0.115	**0.243**	0.193	0.092	-0.109
(kcal/day)	p	0.519	0.950	0.274	0.140	**0.033***	**0.030***	0.910	0.579	0.317	**0.033***	0.093	0.425	0.346
Energy intake	r_s	-0.041	0.014	**0.310**	0.070	0.130	**0.309**	0.167	0.171	**0.357**	**0.294**	0.023	**0.239**	-0.102
(kcal/day)	p	0.723	0.907	**0.006*†**	0.546	0.260	**0.007*†**	0.147	0.137	**0.001*†**	**0.009*†**	0.845	**0.036***	0.375
Protein intake	r_s	0.015	**0.256**	**0.444**	**0.225**	-0.064	**0.309**	**0.332**	0.178	**0.314**	**0.386**	0.102	**0.411**	0.178
(g/day)	p	0.894	**0.025***	**<0.001**†**	**0.049***	0.582	**0.007*†**	**0.003*†**	0.122	**0.005*†**	**<0.001**†**	0.376	**<0.001**†**	0.122
Carbohydrate in-	r_s	-0.037	**-0.229**	0.030	-0.145	0.012	0.052	-0.086	-0.064	0.055	0.043	-0.091	-0.045	**-0.293**
take (g/day)	p	0.751	**0.045***	0.795	0.210	0.919	0.652	0.458	0.579	0.635	0.711	0.431	0.696	**0.010*†**
Fat intake	r_s	-0.006	0.197	**0.296**	0.222	0.160	**0.314**	**0.262**	0.224	**0.306**	**0.322**	0.113	**0.274**	0.108
(g/day)	p	0.961	0.086	**0.009*†**	0.053	0.164	**0.006*†**	**0.021***	0.050	**0.007*†**	**0.004*†**	0.328	**0.016***	0.350
Saturated fat intake	r_s	0.125	**0.314**	**0.377**	**0.290**	0.053	**0.308**	**0.324**	0.218	**0.368**	**0.443**	0.153	**0.371**	**0.257**
(g/day)	p	0.280	**0.005*†**	**<0.001**†**	**0.011*†**	0.648	**0.007*†**	**0.004*†**	0.057	**<0.001**†**	**<0.001**†**	0.184	**<0.001**†**	**0.024***

Supplementary Table S16. ***(continued)***

		IL-1β	IFN-α2	IFN-γ	TNF-α	MCP-1	IL-6	IL-8	IL-10	IL-12p70	IL-17A	IL-18	IL-23	IL-33
Monounsaturated fat intake (g/day)	r_s	-0.020	0.154	**0.258**	0.101	**0.247**	**0.353**	**0.253**	0.143	**0.277**	**0.272**	0.134	0.213	0.121
	p	0.865	0.181	**0.023***	0.383	**0.031***	**0.002*†**	**0.026***	0.214	**0.015*†**	**0.017***	0.244	0.063	0.293
Polyunsaturated fat intake (g/day)	r_s	0.027	**0.312**	**0.273**	**0.263**	0.152	**0.303**	**0.228**	**0.300**	**0.337**	**0.351**	0.133	**0.262**	0.158
	p	0.816	**0.006*†**	**0.016***	**0.021***	0.186	**0.008*†**	**0.046***	**0.008*†**	**0.003*†**	**0.002*†**	0.247	**0.021***	0.170
n-6 fat intake (g/day)	r_s	0.132	**0.306**	**0.320**	**0.261**	0.217	**0.362**	**0.294**	**0.251**	**0.337**	**0.396**	0.206	**0.283**	0.183
	p	0.254	**0.007*†**	**0.004*†**	**0.022***	0.059	**0.001*†**	**0.009*†**	**0.028***	**0.003*†**	**<0.001**†**	0.072	**0.012*†**	0.111
n-3 fat intake (g/day)	r_s	-0.143	-0.125	-0.050	-0.155	-0.163	-0.104	-0.154	-0.058	-0.126	-0.140	-0.105	**-0.247**	-0.155
	p	0.215	0.277	0.667	0.177	0.156	0.373	0.183	0.616	0.273	0.226	0.364	**0.030***	0.178
n-6/n-3 fat intake ratio	r_s	0.164	0.208	0.178	**0.224**	**0.224**	**0.236**	**0.269**	0.162	**0.238**	**0.289**	0.181	**0.300**	0.157
	p	0.154	0.069	0.121	**0.050***	**0.050***	**0.040***	**0.018***	0.159	**0.037***	**0.011*†**	0.116	**0.008*†**	0.173
Trans-fat intake (g/day)	r_s	-0.093	0.081	0.103	0.117	-0.011	0.088	0.098	0.148	0.177	0.043	0.141	0.216	-0.016
	p	0.424	0.481	0.371	0.312	0.927	0.451	0.398	0.198	0.124	0.710	0.221	0.060	0.893
Cholesterol intake (mg/day)	r_s	0.102	**0.395**	**0.381**	**0.360**	0.007	**0.374**	**0.429**	**0.337**	**0.308**	**0.381**	0.124	**0.386**	**0.341**
	p	0.380	**<0.001**†**	**<0.001**†**	**0.001*†**	0.953	**<0.001**†**	**<0.001**†**	**0.003*†**	**0.006*†**	**<0.001**†**	0.281	**<0.001**†**	**0.002*†**
Fiber intake (g/day)	r_s	**-0.347**	**-0.436**	**-0.259**	**-0.410**	0.050	**-0.244**	**-0.341**	**-0.327**	-0.187	**-0.411**	-0.191	**-0.363**	**-0.409**
	p	**0.002*†**	**<0.001**†**	**0.023***	**<0.001**†**	0.663	**0.034***	**0.002*†**	**0.004*†**	0.103	**<0.001**†**	0.096	**0.001*†**	**<0.001**†**
DII score	r_s	**0.246**	0.199	0.043	0.187	-0.152	-0.013	0.105	0.154	0.006	**0.295**	0.035	0.153	0.136
	p	**0.031***	0.083	0.707	0.104	0.188	0.909	0.363	0.181	0.961	**0.009*†**	0.763	0.183	0.237
E-DII score	r_s	0.186	0.222	0.204	**0.230**	-0.101	0.129	0.204	0.173	0.121	**0.417**	0.055	**0.310**	0.144
	p	0.105	0.053	0.075	**0.044***	0.384	0.268	0.074	0.132	0.294	**<0.001**†**	0.633	**0.006*†**	0.212

IL = interleukin, IFN = interferon, TNF = tumor necrosis factor, MCP = monocyte chemoattractant protein, DII = dietary inflammatory index, E-DII = energy-adjusted dietary inflammatory index, r_s = Partial Spearman's correlation coefficients, adjusted for sex, age, BMI, smoking, and physical activity level, p = statistical significance of correlations. Exploratory sensitivity analyses. Nominal p-values are reported. Bold values indicate statistical significance at * $p < 0.05$, ** $p < 0.001$. Values marked with a dagger (†) remained significant after Benjamini–Hochberg false discovery rate (FDR) correction for 13 cytokine/chemokine and 26 dietary variables multiple correlations.

Supplementary Table S17. Correlations among various food groups and dietary factors (heatmap).

Food group/nutrient	Milk products	Egg products	Meat products	Fish products	Edible fats/oils	Grain products	Nuts/ seeds	Vegetable products	Fruit products	Sugar products	Beverages
						r_s					
Milk products (kcal/day)	-	0.645	0.704	0.314	0.208	0.115	-0.333	-0.537	-0.507	0.315	0.153
Egg products (kcal/day)	0.645	-	0.579	0.337	0.228	-0.020	-0.369	-0.361	-0.403	0.280	0.104
Meat products (kcal/day)	0.704	0.579	-	0.265	0.215	0.128	-0.398	-0.613	-0.597	0.308	0.049
Fish products (kcal/day)	0.314	0.337	0.265	-	0.086	-0.203	-0.205	-0.228	-0.134	0.069	-0.077
Edible fats/oils (kcal/day)	0.208	0.228	0.215	0.086	-	0.128	-0.236	0.007	-0.303	0.098	0.145
Grain products (kcal/day)	0.115	-0.020	0.128	-0.203	0.128	-	-0.046	-0.004	-0.232	0.174	-0.007
Nuts/seeds (kcal/day)	-0.333	-0.369	-0.398	-0.205	-0.236	-0.046	-	0.264	0.293	-0.262	0.033
Vegetable products (kcal/day)	-0.537	-0.361	-0.613	-0.228	0.007	-0.004	0.264	-	0.365	-0.144	-0.046
Fruit products (kcal/day)	-0.507	-0.403	-0.597	-0.134	-0.303	-0.232	0.293	0.365	-	-0.319	-0.145
Sugar products (kcal/day)	0.315	0.280	0.308	0.069	0.098	0.174	-0.262	-0.144	-0.319	-	0.126
Beverages (kcal/day)	0.153	0.104	0.049	-0.077	0.145	-0.007	0.033	-0.046	-0.145	0.126	-
Energy (kcal/day)	0.320	0.226	0.339	0.014	0.400	0.516	0.085	0.057	-0.109	0.260	0.142
Protein (g/day)	0.572	0.499	0.628	0.305	0.182	0.325	-0.111	-0.190	-0.378	0.240	0.046
Carbohydrate (g/day)	-0.172	-0.188	-0.206	-0.279	0.050	0.567	0.103	0.352	0.314	0.225	0.082
Fat (g/day)	0.546	0.459	0.569	0.211	0.590	0.248	0.026	-0.230	-0.437	0.284	0.115
Saturated fat (g/day)	0.715	0.594	0.729	0.262	0.452	0.231	-0.282	-0.377	-0.557	0.483	0.150
Monounsaturated fat (g/day)	0.420	0.378	0.451	0.183	0.544	0.214	0.137	-0.122	-0.352	0.153	0.147
Polyunsaturated fat (g/day)	0.268	0.218	0.300	0.262	0.567	0.167	0.094	-0.011	-0.253	0.156	0.028
n-6 polyunsaturated fat (g/day)	0.284	0.235	0.354	0.175	0.647	0.214	0.048	-0.042	-0.328	0.156	0.074
n-3 polyunsaturated fat (g/day)	0.010	0.110	0.025	0.240	0.139	0.103	0.126	0.138	0.107	-0.036	-0.152
n-6/n-3 polyunsaturated fat ratio	0.217	0.071	0.253	-0.097	0.321	0.073	-0.077	-0.192	-0.325	0.126	0.144
Trans-fat (g/day)	0.381	0.343	0.386	0.163	0.227	0.211	-0.232	-0.156	-0.431	0.247	0.122
Cholesterol (mg/day)	0.753	0.829	0.782	0.373	0.327	0.080	-0.425	-0.508	-0.564	0.358	0.123
Fiber (g/day)	-0.612	-0.528	-0.717	-0.242	-0.149	0.064	0.492	0.699	0.645	-0.245	-0.157
Alcohol (g/day)	0.131	-0.016	0.024	0.008	0.117	-0.054	0.013	-0.088	-0.052	-0.033	0.618
Fe (mg/day)	-0.274	-0.140	-0.313	-0.102	0.006	0.236	0.368	0.590	0.325	-0.041	-0.121
Zn (mg/day)	0.260	0.196	0.308	-0.015	0.090	0.425	0.231	0.097	-0.091	0.089	0.047
Cu (mg/day)	-0.439	-0.371	-0.541	-0.264	-0.142	0.115	0.591	0.551	0.538	-0.183	-0.050
I (μg/day)	0.680	0.599	0.649	0.280	0.437	0.190	-0.318	-0.293	-0.451	0.305	0.186
Se (μg/day)	0.627	0.681	0.686	0.548	0.212	0.060	-0.216	-0.343	-0.360	0.252	0.068
Ca (mg/day)	0.426	0.214	0.151	0.081	0.048	0.236	0.101	-0.001	0.024	0.122	0.052
P (mg/day)	0.433	0.359	0.384	0.230	0.084	0.359	0.105	-0.007	-0.150	0.191	0.071
Mg (mg/day)	-0.438	-0.407	-0.542	-0.193	-0.201	0.159	0.671	0.604	0.516	-0.144	-0.008
K (mg/day)	-0.285	-0.196	-0.330	-0.069	0.017	-0.044	0.356	0.555	0.584	-0.140	-0.017
Na (mg/day)	0.472	0.394	0.555	0.136	0.478	0.293	-0.256	-0.153	-0.408	0.297	0.131
NaCl (mg/day)	0.133	0.092	0.246	-0.064	0.539	0.078	-0.132	0.003	-0.244	0.066	0.159
β-carotene (μg/day)	-0.293	-0.200	-0.412	-0.014	0.036	-0.146	0.049	0.375	0.381	-0.180	-0.059
Retinol (μg/day)	0.832	0.752	0.775	0.385	0.235	0.069	-0.433	-0.545	-0.547	0.366	0.098
Vitamin A RE (μg/day)	-0.039	0.049	-0.144	0.067	0.132	-0.101	-0.138	0.196	0.223	-0.067	-0.077
Vitamin A RAE (μg/day)	0.133	0.209	0.042	0.142	0.183	-0.085	-0.227	0.060	0.109	0.006	-0.068
Vitamin B1 (mg/day)	-0.067	0.008	-0.034	-0.043	0.067	0.258	0.209	0.262	-0.057	0.245	0.226
Vitamin B2 (mg/day)	0.403	0.364	0.346	0.080	0.161	0.096	-0.035	-0.144	-0.132	0.142	0.229
Vitamin B3 NE (mg/day)	0.357	0.310	0.493	0.279	0.227	0.223	-0.041	-0.202	-0.303	0.250	0.116
Vitamin B5 (mg/day)	0.113	0.125	0.158	0.096	0.120	0.036	-0.030	0.117	0.105	0.059	0.027
Vitamin B6 (mg/day)	0.034	0.085	0.148	0.173	0.030	-0.032	0.125	0.143	0.322	-0.001	-0.047
Vitamin B9 (μg/day)	-0.479	-0.274	-0.586	-0.137	0.029	-0.031	0.321	0.702	0.521	-0.106	-0.063
Vitamin B12 (μg/day)	0.659	0.581	0.674	0.465	0.151	0.037	-0.325	-0.486	-0.435	0.319	0.128
Vitamin C (mg/day)	-0.380	-0.257	-0.501	-0.086	-0.013	-0.211	0.264	0.453	0.483	-0.180	0.081
Vitamin D (mg/day)	0.610	0.750	0.569	0.536	0.121	-0.012	-0.386	-0.333	-0.359	0.333	0.084
Vitamin E (mg/day)	0.091	0.104	0.066	0.094	0.545	0.088	0.146	0.158	-0.077	0.085	0.072
Polyphenols (mg/day)	-0.462	-0.323	-0.533	-0.174	-0.115	-0.106	0.322	0.441	0.627	-0.150	0.057
DII score	0.343	0.194	0.481	-0.062	0.084	0.080	-0.302	-0.543	-0.508	0.184	0.009
E-DII score	0.540	0.378	0.647	-0.021	0.256	0.317	-0.288	-0.518	-0.549	0.317	0.074

DII = dietary inflammatory index, E-DII = energy-adjusted dietary inflammatory index, r_s = Spearman's correlation coefficients. Exploratory analyses.

Heatmap color scale legend (r_s correlation coefficients values):

1.0	0.9	0.8	0.7	0.6	0.5	0.4	0.3	0.2	0.1	0.0	-0.1	-0.2	-0.3	-0.4	-0.5	-0.6	-0.7	-0.8	-0.9	-1.0

Supplementary Table S18. Linear regression – forced-entry multivariate models for predicting serum cytokine levels (full description of the model).

Cytokine	Predictor	*B*	95% *CI* for *B*	Predictor significance *p*	Adjusted R^2	Model significance *p*
IL-1β	(Constant)	2.322	(0.430 – 4.214)	**0.017**	0.026	0.131
	Vegetarian diet	-0.211	(-0.478 – 0.057)	0.122		
	Sex (female)	0.111	(-0.220 – 0.441)	0.508		
	Age	0.007	(-0.011 – 0.024)	0.458		
	BMI	-0.030	(-0.091 – 0.031)	0.332		
	Physical activity level	-0.157	(-0.344 – 0.030)	0.100		
	Smoking status	-0.008	(-0.377 – 0.362)	0.967		
IFN-α2	(Constant)	0.606	(-0.408 – 1.619)	0.240	0.018	0.195
	Vegetarian diet	-0.168	(-0.312 – -0.025)	**0.022**		
	Sex (female)	0.121	(-0.056 – 0.298)	0.178		
	Age	0.003	(-0.006 – 0.012)	0.522		
	BMI	0.009	(-0.023 – 0.042)	0.568		
	Physical activity level	-0.018	(-0.119 – 0.082)	0.719		
	Smoking status	0.002	(-0.196 – 0.200)	0.988		
IFN-γ	(Constant)	1.027	(-0.333 – 2.387)	0.138	0.040	0.064
	Vegetarian diet	-0.259	(-0.452 – -0.067)	**0.009***		
	Sex (female)	0.179	(-0.059 – 0.416)	0.140		
	Age	0.004	(-0.009 – 0.0179)	0.540		
	BMI	-0.001	(-0.045 – 0.043)	0.963		
	Physical activity level	-0.033	(-0.167 – 0.102)	0.632		
	Smoking status	0.019	(-0.246 – 0.285)	0.886		
TNF-α	(Constant)	0.248	(-1.238 – 1.733)	0.742	0.045	0.050
	Vegetarian diet	-0.235	(-0.446 – -0.025)	**0.028**		
	Sex (female)	0.304	(0.044 – 0.563)	**0.022**		
	Age	-0.008	(-0.021 – 0.006)	0.276		
	BMI	0.047	(-0.001 – 0.095)	0.053		
	Physical activity level	0.041	(-0.106 – 0.188)	0.585		
	Smoking status	0.047	(-0.244 – 0.337)	0.751		
MCP-1	(Constant)	2.287	(1.750 – 2.824)	**<0.001**	0.037	0.076
	Vegetarian diet	0.023	(-0.053 – 0.099)	0.547		
	Sex (female)	-0.064	(-0.158 – 0.030)	0.179		
	Age	0.006	(0.001 – 0.011)	**0.019**		
	BMI	0.003	(-0.014 – 0.020)	0.730		
	Physical activity level	-0.027	(-0.080 – 0.026)	0.321		
	Smoking status	-0.061	(-0.166 – 0.044)	0.255		
IL-6	(Constant)	0.058	(-1.141 – 1.256)	0.924	0.026	0.133
	Vegetarian diet	-0.181	(-0.351 – -0.012)	**0.036**		
	Sex (female)	0.201	(-0.008 – 0.411)	0.059		
	Age	0.001	(-0.010 – 0.012)	0.857		

	BMI	0.037	(-0.001 – 0.076)	0.059		
	Physical activity level	0.017	(-0.101 – 0.136)	0.772		
	Smoking status	-0.033	(-0.267 – 0.201)	0.779		
IL-8	(Constant)	1.312	(-0.710 – 3.334)	0.202	0.068	**0.013**
	Vegetarian diet	-0.540	(-0.827 – -0.254)	**<0.001***		
	Sex (female)	0.065	(-0.288 – 0.418)	0.715		
	Age	0.008	(-0.011 – 0.027)	0.387		
	BMI	0.033	(-0.032 – 0.098)	0.321		
	Physical activity level	-0.079	(-0.279 – 0.122)	0.439		
	Smoking status	-0.146	(-0.541 – 0.249)	0.466		
IL-10	(Constant)	0.473	(-0.782 – 1.727)	0.458	0.037	0.074
	Vegetarian diet	-0.206	(-0.383 – -0.028)	**0.024**		
	Sex (female)	0.135	(-0.085 – 0.354)	0.227		
	Age	0.011	(-0.001 – 0.022)	0.070		
	BMI	0.008	(-0.032 – 0.049)	0.692		
	Physical activity level	-0.015	(-0.140 – 0.109)	0.806		
	Smoking status	-0.097	(-0.343 – 0.148)	0.433		
IL-12p70	(Constant)	0.591	(-0.526 – 1.708)	0.297	0.052	**0.033**
	Vegetarian diet	-0.216	(-0.374 – -0.058)	**0.008***		
	Sex (female)	0.125	(-0.070 – 0.320)	0.208		
	Age	0.009	(-0.001 – 0.020)	0.081		
	BMI	0.003	(-0.033 – 0.040)	0.851		
	Physical activity level	-0.031	(-0.141 – 0.080)	0.584		
	Smoking status	-0.063	(-0.281 – 0.155)	0.567		
IL-17A	(Constant)	-0.083	(-1.242 – 1.077)	0.888	0.092	**0.003**
	Vegetarian diet	-0.318	(-0.482 – -0.154)	**<0.001***		
	Sex (female)	0.166	(-0.036 – 0.369)	0.107		
	Age	0.007	(-0.004 – 0.017)	0.232		
	BMI	0.004	(-0.034 – 0.041)	0.850		
	Physical activity level	0.042	(-0.073 – 0.157)	0.469		
	Smoking status	0.021	(-0.205 – 0.248)	0.852		
IL-18	(Constant)	2.474	(1.895 – 3.053)	**<0.001**	0.014	0.682
	Vegetarian diet	-0.049	(-0.131 – 0.033)	0.237		
	Sex (female)	-0.048	(-0.150 – 0.053)	0.345		
	Age	0.003	(-0.002 – 0.009)	0.246		
	BMI	0.0001	(-0.019 – 0.019)	0.994		
	Physical activity level	-0.020	(-0.077 – 0.037)	0.491		
	Smoking status	0.001	(-0.112 – 0.114)	0.992		
IL-23	(Constant)	0.469	(-1.021 – 1.959)	0.535	0.020	0.177
	Vegetarian diet	-0.180	(-0.391– 0.031)	0.093		
	Sex (female)	0.116	(-0.144 – 0.376)	0.380		
	Age	0.001	(-0.013 – 0.015)	0.918		
	BMI	0.032	(-0.016 – 0.080)	0.194		
	Physical activity level	-0.091	(-0.239 – 0.056)	0.223		

	Smoking status	0.199	(-0.092 – 0.490)	0.178		
IL-33	(Constant)	1.409	(0.047 – 2.772)	**0.043**	0.010	0.284
	Vegetarian diet	-0.169	(-0.362 – 0.024)	0.085		
	Sex (female)	0.145	(-0.093 – 0.383)	0.230		
	Age	0.006	(-0.006 – 0.019)	0.332		
	BMI	0.015	(-0.029 – 0.059)	0.514		
	Physical activity level	-0.040	(-0.175 – 0.095)	0.560		
	Smoking status	-0.133	(-0.399 – 0.133)	0.324		

Cytokine/chemokine levels were log-transformed for regression analyses. Model predictors: type of diet (vegetarian vs. omnivorous), sex, age, BMI, smoking status, and physical activity level. BMI = body mass index, *B* = unstandardized regression coefficient, *CI* = confidence interval, R^2 = coefficient of determination, *p* = statistical significance. Exploratory analyses. Nominal *p*-values are reported. Bold values indicate statistical significance at $p < 0.05$. Predictor *p*-values marked with an asterisk (*) remained significant after Benjamini–Hochberg false discovery rate (FDR) correction for 13 cytokine/chemokine multiple testing.

Supplementary Table S19. Linear regression – stepwise multivariate models for predicting serum cytokine levels

Cytokine	Model	*B*	95% *CI* for *B*	Predictor significance *p*	Adjusted R^2	Model significance *p*
IL-1β	Constant	1.006	(0.586 – 1.425)	**<0.001**	0.021	**0.043**
	Sex (female)	0.274	(0.008 – 0.539)	**0.043**		
IFN-α2	Constant	1.075	(0.853 – 1.298)	**<0.001**	0.031	**0.017**
	Vegetarian diet	-0.172	(-0.312 – -0.031)	**0.017***		
IFN-γ	Constant	1.047	(0.633 – 1.462)	**<0.001**	0.062	**0.003**
	Vegetarian diet	-0.258	(-0.446 – -0.069)	**0.008***		
	Sex (female)	0.198	(0.010 – 0.386)	**0.039**		
TNF-α	Constant	1.621	1.290 – 1.951)	**<0.001**	0.032	**0.016**
	Vegetarian diet	-0.258	(-0.467 – -0.049)	**0.016***		
MCP-1	Constant	2.227	(2.052 – 2.403)	**<0.001**	0.032	**0.016**
	Age	0.006	(0.001 – 0.011)	**0.016**		
IL-6	Constant	1.273	(1.009 – 1.538)	**<0.001**	0.027	**0.025**
	Vegetarian diet	-0.192	(-0.359 – -0.0249	**0.025***		
IL-8	Constant	2.236	(1.792 – 2.680)	**<0.001**	0.079	**<0.001**
	Vegetarian diet	-0.528	(-0.809 – -0.247)	**<0.001***		
IL-10	Constant	0.769	(0.283 – 1.255)	**0.002**	0.046	**0.011**
	Vegetarian diet	-0.200	(-0.375 – -0.026)	**0.025***		
	Age	0.012	(0.000 – 0.023)	**0.045**		
IL-12p70	Constant	1.099	(0.851 – 1.348)	**<0.001**	0.039	**0.009**
	Vegetarian diet	-0.212	(-0.369 – -0.055)	**0.009***		
IL-17A	Constant	0.578	(0.322 – 0.8359	**<0.001**	0.092	**<0.001**
	Vegetarian diet	-0.330	(-0.492 – -0.1679	**<0.001***		
IL-18	No significant predictors					
IL-23	No significant predictors					
Il-33	No significant predictors					

Cytokine/chemokine levels were log-transformed for regression analyses. Model predictors: type of diet (vegetarian vs. omnivorous), sex, age, BMI, smoking status, and physical activity level. BMI = body mass index, *B* = unstandardized regression coefficient, *CI* = confidence interval, R^2 = coefficient of determination, *p* = statistical significance. Exploratory sensitivity analyses. Nominal *p*-values are reported. Bold values indicate statistical significance at $p < 0.05$. Predictor *p*-values for dietary pattern marked with an asterisk (*) remained statistically significant after Benjamini–Hochberg false discovery rate (FDR) correction for 13 cytokine/chemokine multiple comparisons.

Supplementary Section S4.1. Overview of the Main Biological Pathways Involved in Cytokine and Chemokine Production and Their Association with Dietary Influences.

The following supplementary section describes the main biological pathways involved in cytokine and chemokine production and associated with dietary influences, providing the necessary background for interpreting our dietary findings. These included: (1) the nuclear factor kappa B (NF-κB) pathway; (2) the NOD-, LRR- and pyrin domain-containing protein 3 (NLRP3) inflammasome pathway; (3) the mitogen-activated protein kinase (MAPK)/activator protein 1 (AP-1) pathway; (4) the mammalian target of rapamycin (mTOR) pathway; (5) the phosphatidylinositol-3 kinase (PI3K)–protein kinase B (PKB/Akt) pathway; (6) the nuclear factor erythroid 2-related factor 2 (Nrf2) pathway; (7) eicosanoid and endocannabinoid signaling pathways; and (8) the gut dysbiosis - LPS pathway.

1. NF-κB	The NF-κB signaling pathway is a central transcriptional regulator of inflammatory responses and is activated by multiple nutrient- and metabolite-sensitive receptors [255]. In unstimulated cells, NF-κB is bound to an inhibitory protein, the inhibitor of nuclear factor kappa B (IκB). The canonical NF-κB pathway is activated by microbial products such as LPS and nucleic acids acting through Toll-like receptors (TLRs), pro-inflammatory cytokines (e.g., TNF-α and IL-1β) acting through their receptors, tumor necrosis factor receptor (TNFR) and interleukin 1 receptor (IL-1R), oxidative stress, and other cellular stress signals, leading to activation of the myeloid differentiation primary response 88 (MyD88)-dependent signaling cascade [256]. This results in the IκB kinase β (IKKβ) activation, consecutive phosphorylation and degradation of IκB, and nuclear translocation of NF-κB (p65/p50), promoting transcription of pro-inflammatory cytokines, including IL-1β, IL-6, IL-8, IL-12, IL-23, TNF-α, and MCP-1 [255,257]. There is also the MyD88-independent pathway that activates late-phase NF-κB and MAPK signaling and induces type I interferons (IFN-α/β) and interferon-inducible genes through the activation of the transcription factor interferon regulatory factor 3 (IRF3) [256,258]. Additionally, TLR activation, through NF-κB and MAPK signaling, also mediates activation of cytosolic phospholipase A2 (cPLA2), leading to arachidonic acid release and the induction of cyclooxygenase (COX) and lipoxygenase (LOX) for eicosanoid production [257,259,260], and proinflammatory inducible nitric oxide synthase (iNOS) [257]. TLRs, which respond to bacterial LPS and other microbial ligands, can also be modulated by dietary fatty acids. Saturated fatty acids, particularly palmitic acid, less stearic, myristic, and lauric, may enhance TLR4 (and TLR2) NF-κB activation indirectly via increased membrane rigidity, increased TLR4 requirement in membrane lipid rafts, and increased TLR4/TLR2 heterodimerization, although direct binding of saturated fatty acids to TLR4 remains controversial [261]. In contrast, monounsaturated fatty acids (e.g., oleic acid), and n-3 polyunsaturated fatty acids, particularly eicosapentaenoic acid (EPA) and docosahexaenoic acid (DHA), attenuate NF-κB signaling through multiple mechanisms, including suppression of TLR4 activation via inhibition of lipid raft formation, alteration of membrane lipid composition, and activation of G protein-coupled receptor 120 (GPR120), also known as free fatty acid receptor 4 (FFAR4) [261–263]. Activation of GPR120 subsequently inhibits transforming growth factor-β (TGF-β)-activated kinase 1 (TAK1) and IKKβ signaling, thereby reducing downstream inflammatory responses [261–263]. The n-6 fatty acid, arachidonic acid, can also lead to inhibition of LPS or saturated fatty acids binding to TLR4, but in some tissues, it can also activate TLR4 [262]. Dietary polyphenols (e.g., resveratrol, quercetin) further suppress NF-κB activation

	through inhibition of IKKβ activity and activation of adenosine monophosphate-activated protein kinase (AMPK) and sirtuin 1 (SIRT1), which regulate p65 acetylation and transcriptional activity [264–266]. In addition, dietary fiber-derived short-chain fatty acids (SCFAs), particularly butyrate, activate GPR41 (FFAR3), GPR43 (FFAR2), and GPR109a, which reduce cyclic AMP (cAMP) and, through β-arrestin pathways, inhibit MAPK and NF-κB signaling, involved in cytokine production [267–273]. Additionally, they inhibit histone deacetylases (HDACs), thereby reducing NF-κB DNA binding and inflammatory gene transcription [267–271,273]. These anti-inflammatory mechanisms are typically more prominent in plant-based diets, which are richer in fiber, polyphenols, and mostly based on unsaturated fats, while lower in saturated fat and endotoxin-promoting dietary factors.
2. NLRP3 inflammasome	The NLRP3 inflammasome is a cytosolic multiprotein complex that regulates innate immune activation and maturation of pro-inflammatory cytokines. Its activation requires two signals: a priming signal and an activation signal [274,275]. The priming step is commonly mediated by NF-κB and induces transcription of inactive precursors, including pro-IL-1β and pro-IL-18. TLR signaling and NF-κB also induce transcription of the NLRP3 inflammasome and can also induce post-translational modifications of NLRP3 (deubiquitination and phosphorylation), making NLRP3 more responsive to activation signals [275,276]. The second signal is triggered by cellular stressors such as mitochondrial reactive oxygen species (ROS), K^+ efflux, lysosomal damage, ATP, or cholesterol crystals, leading to caspase-1 activation and cleavage of pro-IL-1β and pro-IL-18 into their biologically active forms [274,277]. Dietary saturated fatty acids (e.g., palmitic acid, less stearic, myristic, and lauric) promote NLRP3 inflammasome activation through TLR4/TLR2 (via NF-κB pathway) and cluster of differentiation 36 (CD36)-mediated lipid uptake, inducing endoplasmic reticulum (ER) stress, mitochondrial dysfunction and ROS production, ceramide synthesis, Ca^{2+} dysregulation, and thioredoxin-interacting protein (TXNIP) activation, all of which promote inflammasome assembly and subsequent maturation of IL-1β and IL-18 [274,278,279]. Cholesterol crystals, often associated with diets rich in animal fats, directly activate NLRP3 via lysosomal destabilization and cathepsin release [274,278]. Trans fatty acids may further enhance inflammasome activation through oxidative stress and membrane remodeling [278]. High sugar and refined carbohydrate intake also activate NLRP3 through oxidative stress [274]. In contrast, n-3 polyunsaturated fatty acids (EPA and DHA) and monounsaturated oleic acid (and, to a lesser extent, n-6 polyunsaturated linoleic and arachidonic acid) inhibit inflammasome expression and activation by reducing TLR4 priming, limiting ROS production, activating AMPK, and generating specialized pro-resolving mediators (SPMs) [274,278]. Short-chain fatty acids such as butyrate (less acetate and propionate) suppress NLRP3 transcription and activation by HDACs, by improving mitochondrial function, and by reducing oxidative stress [267,268,272,273,280,281]. Dietary polyphenols (e.g., curcumin, resveratrol, epigallocatechin gallate) directly inhibit NLRP3 assembly and caspase-1 activation, while enhancing Nrf2-mediated antioxidant defenses [266,277,282–284]. Excess animal protein and heme Fe from red meat may promote oxidative stress, gut dysbiosis, and endotoxin translocation, further facilitating inflammasome priming [285–287]. Diets rich in plant-based foods, therefore, tend to reduce inflammasome activation and downstream production of IL-1β, IL-18, and, indirectly, other cytokines, because IL-1β is

	a strong inducer of NF-κB and MAPK signaling [118]. In general, vegetarian dietary patterns, through higher intake of fiber, polyphenols, and unsaturated fatty acids, combined with lower intake of saturated fat, cholesterol, and heme Fe, collectively shift signaling away from inflammasome activation [274], resulting in reduced IL-1β and IL-18 maturation and consequently lower downstream cytokine amplification.
3. MAPK/AP-1	Beyond the classical NF-κB–dependent pathway, cytokine transcription is also considerably regulated by activation of the AP-1 complex, which is triggered downstream of metabolically active phospholipase C (PLC)/diacylglycerol (DAG)/protein kinase C (PKC)/MAPK pathways, including extracellular signal–regulated kinase (ERK), and stress kinases, c-Jun N-terminal kinase (JNK) and p38 MAPK [288–290]. These pathways are also activated following stimulation of specific receptor families, including TLR4, TLR3, TLR7, and TLR9, recognizing also LPS and fatty acids [289]. Additionally, they are activated by cytokine receptors such as TNFR1 and IL-1R1, which respond to inflammatory cytokines from the TNF and IL-1 family, respectively, amplifying systemic inflammation by a positive feedback loop, and by antigen receptors, including the T cell receptor (TCR) and B cell receptor (BCR), which mediate adaptive immune activation [289,291,292]. AP-1, primarily composed of c-Jun (Jun proto-oncogene) and c-Fos (Fos proto-oncogene), binds to TPA (12-O-tetradecanoylphorbol-13-acetate) response elements (TRE) within promoter and enhancer regions and cooperates with NF-κB and other transcription factors to amplify transcriptional output [288,290,293]. This pathway drives the expression of several pro-inflammatory and immunoregulatory cytokines, including IL-1β, TNF-α, IL-6, IL-12, IL-23, and IL-10, and, in cooperation with IL-12, IL-23, IL-18, and IL-33, contributes to IFN-γ transcription and stabilization [134,151,294–299]. AP-1 also regulates chemokine genes such as IL-8 and MCP-1 [300–302], and can modulate both pro- and anti-inflammatory outputs depending on context, including effects on IL-10 [299]. Collectively, these observations highlight that AP-1 functions as a complementary and context-dependent transcriptional regulator that amplifies or fine-tunes cytokine expression beyond NF-κB–dominant signaling [293]. Dietary factors can modulate activation of AP-1 predominantly through regulation of upstream MAPKs, including JNK, ERK, and p38, as well as through redox-sensitive signaling, PKC, and TLR activation [303]. Pro-inflammatory dietary components such as saturated fatty acids (e.g., palmitic, stearic, and myristic acids) can enhance TLR4 signaling [304,305] and increase ER stress, ceramide, DAG, and ROS production and PKC activation, thereby activating MAPK cascades and enhancing AP-1–dependent transcription of cytokines [306]. Similarly, high glycemic load and hyperglycemia promote AP-1 activation via mitochondrial ROS generation, advanced glycation end product (AGE)–mediated signaling, and PKC activation [303,307]. In contrast, n-3 polyunsaturated fatty acids (e.g., EPA and DHA), oleic acid, and SCFAs attenuate AP-1 activity by inhibiting JNK, ERK1/2, and p38 signaling and reducing oxidative stress [271,308,309]. Amino acids also exert immunomodulatory effects. Leucine and glutamine support immune cell activation and proliferation through metabolic pathways, including activation of the mTOR and MAPK signaling, thereby indirectly facilitating AP-1-dependent gene transcription [310,311]. In contrast, cysteine serves as a precursor for glutathione synthesis, reducing intracellular oxidative stress and suppressing AP-1 activation through redox-dependent mechanisms [312]. Folate may also indirectly support glutathione homeostasis through

	its role in one-carbon metabolism and homocysteine remethylation, thereby maintaining sulfur amino acid balance, preserving cysteine availability for glutathione synthesis, improving cellular redox status, and indirectly attenuating redox-sensitive AP-1 activation [313]. Dietary fiber–derived SCFAs further contribute to AP-1 suppression through epigenetic mechanisms, including histone deacetylase inhibition [267–269,271]. Micronutrients and phytochemicals, including vitamins C, E, D, folates, vitamin A metabolites (all-trans-retinoic acid and 9-cis-retinoic acid), Zn, Se, and polyphenolic compounds such as resveratrol and curcumin, inhibit AP-1 by reducing ROS levels, suppressing MAPK activation, and enhancing Nrf2 transcription factor–mediated antioxidant responses [314–318]. Collectively, these findings indicate that diet influences AP-1–dependent cytokine transcription primarily by modulating oxidative stress, MAPK/stress kinases, NF-κB signaling, and epigenetic histone modifications, with pro-inflammatory dietary patterns enhancing, while fiber- and antioxidant-rich dietary patterns attenuating AP-1–mediated inflammatory responses.
4. mTOR	The mTOR pathway functions as a central immune–metabolic integrator that couples nutrient availability and receptor-mediated signaling to cytokine production by immune cells [319,320]. This pathway operates mainly through two complexes, mTOR complex 1 (mTORC1) and mTOR complex 2 (mTORC2) [321]. It is activated by converging upstream signals, including signaling downstream of insulin and insulin-like growth factor 1 (IGF-1), PI3K–Akt, MAPK, amino acid sensing pathways (particularly leucine and arginine, via Rag GTPases, less glutamine), and immune receptor signaling downstream of the TLRs, TCRs, and cytokine receptors such as the IL-2 receptor (IL-2R) [303,310,322]. These inputs converge to activate mTORC1, which then phosphorylates downstream effectors such as ribosomal protein S6 kinase (S6K1) and eukaryotic translation initiation factor 4E-binding protein 1 (4E-BP1), thereby enhancing translation of cytokine mRNAs [322]. This results in increased production of cytokines (including IL-2, TNF-α, IL-6, and IL-1β), while in T helper cells ($CD4^+$) it stimulates Th1/Th17 cell programming and promotes transcription of IFN-γ and IL-17 production through cooperation with hypoxia-inducible factor 1 alpha (HIF-1α) [323–325]. In $CD8^+$ cytotoxic T cells and natural killer (NK) cells, mTORC1 supports production of IFN-γ [323–326]. In plasmacytoid dendritic cells (pDCs), mTORC1 activation downstream of TLR7/9 signaling promotes IRF7-dependent production of type I interferons (IFN-α and IFN-β) [323]. In macrophages and monocytes, mTORC1 activation in some circumstances stimulates M1 polarization and synergizes with NF-κB and AP-1 to enhance transcription and translation of IL-1β, TNF-α, IL-6, and MCP-1, thereby amplifying monocyte recruitment and inflammatory amplification loops [323–325]. In conventional dendritic cells (cDCs), depending on type and activation conditions, mTORC1 can promote IL-12 production for Th1 priming [323,324]. Conversely, inhibition of mTOR signaling via energy deficit or AMPK activation can promote a regulatory T cell (Treg) phenotype and production of regulatory IL-10 via Forkhead box P3 (FOXP3), a master transcription factor of Tregs [269,318,324]. However, in myeloid immune cells (including monocytes, macrophages, and DCs), mTORC1 activation can also promote IL-10 production while suppressing NF-κB pathway and IL-12-driven inflammatory responses, thereby contributing to the regulation of immune activation [323]. Thus, mTOR functions as a nutrient- and growth factor–dependent metabolic checkpoint that determines

	whether immune cells adopt a pro-inflammatory cytokine-producing phenotype or a more metabolically restrained regulatory phenotype, depending on the immune cell type and activation context [323,326,327]. Diets rich in animal protein, particularly branched-chain amino acids such as leucine, together with increased circulating insulin and IGF-1 concentrations, may enhance mTORC1 activity, whereas plant-based diets, characterized by lower leucine intake, higher polyphenol and dietary fiber consumption, and increased production of SCFAs, may suppress mTOR signaling, by activating AMPK [328–330].
5. PI3K–Akt	In addition, the PI3K–Akt pathway is another key intracellular signaling cascade involved in the regulation of metabolism, cell survival, and immune responses, exerting context-dependent effects on inflammatory signaling and cytokine production [303]. The signaling pathway may play both pro- and anti-inflammatory roles depending on the cellular context and duration of activation. The pathway is activated by diverse extracellular signals, including insulin, growth factors, TLR ligands, antigen receptors (TCRs and BCRs), cytokines, chemokines, endocannabinoids, SCFAs, and SPMs, acting through their distinct receptor systems [303]. Following activation of PI3K, downstream phosphorylation of Akt regulates multiple metabolic and inflammatory pathways, including mTOR, NF-κB, AP-1, signal transducer and activator of transcription-3 (STAT3), and Forkhead box O (FOXO) nuclear factors signaling [331,332]. By inhibiting glycogen synthase kinase 3 beta (GSK3β), it negatively influences NF-κB and AP-1 pathways [333]. In several innate immune cell types, transient PI3K–Akt activation acts as a negative feedback regulator of TLR-mediated inflammatory responses, thereby limiting excessive NF-κB activation and production of proinflammatory cytokines, while it can stimulate M2 polarization and IL-10 production [332,334]. However, chronic or dysregulated activation of the pathway, particularly in obesity and insulin resistance, may contribute to persistent low-grade inflammation, altered macrophage polarization, and increased production of inflammatory mediators, including various pro-inflammatory cytokines [274,303,335–337]. Several dietary factors may modulate PI3K–Akt signaling [303,335,336]. Diets rich in saturated and trans fatty acids may promote chronic activation of inflammatory PI3K–Akt/mTOR pathways through metabolic stress and TLR signaling, whereas n-3 polyunsaturated fatty acids, SCFAs, polyphenols, carotenoids, and other antioxidant compounds abundant in plant-based diets may attenuate inflammatory signaling and oxidative stress through modulation of Akt, NF-κB, MAPK, AMPK, and Nrf2-related pathways [271,303,318,329,330,337,338]. In addition, insulin and amino acid availability strongly influence PI3K–Akt activation, linking nutrient sensing with immune and metabolic regulation [303].
6. Nrf2	The Nrf2 pathway is a major cellular defense system involved in protection against oxidative stress, electrophilic damage, and chronic inflammation [257,339,340]. It induces transcription of numerous (over 500) cytoprotective and antioxidant enzymes, including heme oxygenase-1 (HO-1) – responsible for degradation of proinflammatory free heme and synthesis of antioxidants bilirubin and carbon-monoxide [216,341], superoxide dismutase (SOD), catalase, glutathione (GSH)/glutathione peroxidase (GPX), glutathione reductase (GR), glutaredoxin (Grx), glutathione S-transferase (GST), thioredoxin (TXN), thioredoxin reductase (TXNRD), glutamate cysteine ligase (GCL), and NAD(P)H quinone oxidoreductase 1 (NQO1) [257,339,340]. Through reduction of ROS and modulation of redox-sensitive

	signaling pathways, Nrf2 suppresses activation of NF-κB, MAPK, mTOR, PI3K–Akt, and NLRP3 inflammasome pathways, thereby reducing production of pro-inflammatory cytokines such as IL-6, TNF-α, IL-1β, IL-17, and MCP-1, as well as COX-2 and iNOS [257,339,342]. Several dietary factors abundant in plant-based diets may activate Nrf2 signaling, including polyphenols (e.g., curcumin, resveratrol, quercetin, epigallocatechin gallate), isothiocyanates (particularly sulforaphane from cruciferous vegetables), carotenoids, vitamins C, E, Zn, Mg, n-6 and n-3 polyunsaturated fatty acids, and dietary fiber-derived SCFAs [267,318,329,337,339,342–345]. In contrast, chronic overnutrition, oxidative stress, hyperglycemia, trans fatty acids, and excessive intake of saturated fats, via NF-κB and other pathways, may impair or dysregulate Nrf2 signaling, thereby contributing to persistent oxidative stress and low-grade inflammation [257,337,346,347]. Consequently, activation of Nrf2 is considered one of the important mechanisms through which plant-rich dietary patterns may exert antioxidant and anti-inflammatory effects.
7. Eicosanoid and endocannabinoid systems	Furthermore, the role of the eicosanoid (oxylipin) and endocannabinoid systems in cytokine production should be discussed. The n-6 fatty acids, via arachidonic acid and COX and LOX pathways, generate classical eicosanoids such as prostaglandin E2 (PGE_2), thromboxane A2 (TXA_2), leukotriene B4 (LTB_4) and oxidized derivatives hydroxyeicosatetraenoic acids (HETEs; 5-HETE, 12-HETE, 15-HETE) that activate G protein–coupled receptor (GPR) signaling and TLR/NF-κB, PKC /MAPK (JNK, ERK, p38)/AP-1 and inflammasome pathways, promoting pro-inflammatory cytokine and chemokine expression [263,348–350]. Vice versa, NF-κB and MAPK signaling pathways stimulate cPLA2, COX, and LOX induction and activation, making a positive feedback loop [351,352]. In contrast, n-3 fatty acids, specifically EPA and DHA, and their plant precursor – α-linolenic acid (ALA), generate structurally distinct eicosanoids including prostaglandin E3 (PGE_3), leukotriene B5 (LTB_5), oxilipins hydroxy-octadecatrienoic and hydroperoxy-octadecatrienoic acids (9-HOTrE, 13-HOTrE, 13-HpOTrE), as well as SPMs such as resolvins, protectins, and maresins, which are generally much weaker stimulators pro-inflammatory pathways, or directly suppress inflammatory signaling (e.g., NF-kB and NLRP3 inflammasome), actively resolve inflammation, and have antioxidative properties [263,348,349,353]. SPMs reduce cytokine production (e.g., TNF-α, IL-1β, IL-6, IL-8, MCP-1, IFN-γ, IL-17A, IL-22) by suppressing NF-κB, NLRP3 inflammasome, and MAPK signaling, Th1 and Th17 activation, neutrophil recruitment, and generation of pro-inflammatory eicosanoids (particularly LTB4), while promoting macrophage polarization toward a pro-resolving (M2-like) phenotype, IL-10 secretion, and stimulating tissue repair [348]. Furthermore, n-6–derived endocannabinoids such as anandamide (N-arachidonoylethanolamine, AEA) and 2-arachidonoylglycerol (2-AG), as well as n-3–derived ethanolamides including eicosapentaenoyl ethanolamide (EPEA) and docosahexaenoyl ethanolamide (DHEA), modulate cannabinoid receptor type 1 (CB1) and cannabinoid receptor type 2 (CB2) signaling, which is involved in the control of immunity and cytokine production (particularly CB2) [354–359]. CB1 is known to activate MAPK/PI3K–Akt, COX/LOX, and iNOS cascades, while CB2 generally has anti-inflammatory effects, but conflicting findings exist [274,355,356,359]. n-3 derived endocannabinoids generally reduce CB1 overactivation, stimulate CB2-mediated immunoregulation, and reduce NF-κB/MAPK/AP-1/PI3K–Akt

	/mTOR/NLRP3 and COX-2 signaling, thereby inhibiting the proinflammatory cytokine and eicosanoid production and promoting resolution of inflammation [274,358,360,361]. Therefore, since ALA-derived pathways indirectly reduce inflammatory signaling by competing for enzymatic processing by desaturases, elongases, COX, LOX, and endocannabinoid-producing enzymes, and shifting membrane phospholipid composition [349], the balance between n-6 and n-3 polyunsaturated fatty acids, rather than their absolute intake alone, appears to play an important role in regulating inflammatory and immune responses.
8. Gut microbiota - LPS	Finally, although bacterial endotoxin (LPS) is not a metabolic factor per se, circulating LPS concentrations are considered an important marker of intestinal permeability and metabolic endotoxemia, both of which are strongly influenced by dietary composition and gut microbiota [362–364]. Increased translocation of LPS from the intestinal lumen into the circulation may activate innate immune signaling pathways, particularly through TLR4, leading to activation of NF-κB and subsequent production of pro-inflammatory cytokines, including IL-6, TNF-α, IL-1β, MCP-1, and other inflammatory mediators [362,364]. Chronic low-grade endotoxemia has been associated with obesity, insulin resistance, metabolic syndrome, and cardiovascular disease [362–364]. Dietary factors may substantially modulate these processes. Diets rich in saturated and trans fatty acids may promote gut dysbiosis, impair intestinal barrier integrity, and facilitate LPS absorption, while saturated fatty acids themselves may additionally amplify the LPS/TLR4-mediated inflammatory signaling and inflammasome activation [1,274,365,366]. In contrast, dietary fiber, polyphenols, and n-3 polyunsaturated fatty acids (also n-6 and monounsaturated) generally exert protective effects by improving gut barrier function, increasing SCFAs production, promoting beneficial microbial composition, suppressing NF-κB/NLRP3 signaling, reducing oxidative stress, and stimulating the formation of SPMs and anti-inflammatory endocannabinoids and activating anti-inflammatory aryl hydrocarbon receptors (AhR), which promote intestinal integrity [1,274,330,359,367]. SCFAs also promote regulatory T cell (Treg) differentiation (via FOXP3 upregulation), shift macrophages from M1 (pro-inflammatory) to M2 (anti-inflammatory) phenotype, and inhibit production of M1-associated cytokines (IL-6, TNF-α, IFN-γ, IL-12) while increasing production of M2-associated anti-inflammatory cytokine (IL-10) [267,268,270,330,368].

All mechanistic pathways mentioned above may contribute to lower circulating levels of cytokines observed in plant-based dietary patterns.

Supplementary Section S4.2. Overview of the Potential Mechanistic Aspects Underlying Associations of Specific Dietary Factors with Cytokine/Chemokine Levels.

The following supplementary section provides the main potential mechanistic pathways underlying associations of specific dietary factors with cytokine levels. We discussed our findings in relation to dietary fats, cholesterol, proteins, fiber, polyphenols, and micronutrients that we have found statistically different between the two groups, as well as their relations with different food groups whose intakes we have found different between the two dietary patterns and associated with cytokine levels in correlation analyses.

Dietary fats and cholesterol	Dietary fats, in particular, saturated fatty acids (palmitic, stearic, and lauric) can induce NF-κB/MAPK activation or the NLRP3 inflammasome induction via amplification of TLR activation (particularly TLR4, less TLR2), mitochondrial ROS overproduction, ER stress, DAG and ceramide formation, and lysosomal damage [274,365,369]. A high-fat meal increases postprandial circulating LPS (a canonical activator of TLR4) and cytokine levels [180,370–372]. High-fat and high-saturated-fat (especially palmitic acid) diets impair the gut barrier and shift the microbiota toward bile-tolerant Gram-negative bacteria producing LPS [1,366,367]. In contrast, monounsaturated fatty acids (e.g., oleic acid) act more neutrally or protectively, since they do not activate TLR and NLRP3 and can even inhibit SFA-induced activation [274,365]. However, they may promote LPS absorption [373]. n-3 polyunsaturated fatty acids (ALA, EPA, DHA) may directly suppress NF-κB, MAPK, and NLRP3 inflammasome signaling through multiple mechanisms, including suppression of TLR4 activation via inhibition of lipid raft formation, alteration of membrane lipid composition, activation of GPRs, AMPK, and Nrf2 signaling, leading to decreased oxidative stress [271,308,309]. They also competitively inhibit proinflammatory eicosanoid and endocannabinoid production, and can serve as precursors of less inflammatory eicosanoids and anti-inflammatory pro-resolving mediators (resolvins, protectins, and maresins) and endocannabinoids [263,348,349,353,374–376]. n-6 polyunsaturated fatty acids (linoleic and arachidonic acid) act in a more context-dependent manner and can be pro-inflammatory (via eicosanoids and endocannabinoids), neutral, or even beneficial depending on the balance [350,377]. Since n-3 and n-6 fatty acids compete for enzymatic processing by desaturases, elongases, and eicosanoid- and endocannabinoid-producing enzymes [106,349], their balance, rather than their absolute intake alone, may play a more significant role in regulating cytokine secretion. Industrial trans fatty acids (e.g., elaidic acid) exert pro-inflammatory effects and have been shown to activate TLR4/NF-κB signaling pathways, leading to increased cytokine production, while their role in inflammasome and MAPK activation appears to be indirect and mediated through ER stress, oxidative stress, and mitochondrial dysfunction [378]. Cholesterol crystals are potent danger-associated signals (DAMPs) and can also activate the NLRP3 inflammasome [379]. Macrophages phagocytose cholesterol crystals, which can cause lysosomal damage, rupture, and release of cathepsins into cytosol, triggering NLRP3 activation [274,379–381]. Saturated and trans fatty acids and sugars increase LDL-cholesterol levels and ROS production, promoting LDL oxidation [3,369]. Oxidized LDL (oxLDL) is subsequently taken up by macrophages via scavenger receptors, activating inflammatory signaling through cooperation with TLRs [3,369]. Consequently, oxLDL can prime the inflammasome by activating TLRs [379].
Dietary proteins	Unlike saturated fats or LPS, dietary proteins and amino acids do not directly affect TLR signaling or the NLRP3 inflammasome, and can have even anti-inflammatory effects, depending on the source [382–385]. For example, an isocaloric high-protein diet can diminish the LPS-induced inflammation and cytokine production in rats, and a decrease in circulating MCP-1 was noted immediately after different high-protein meals in humans [383,384]. Active metabolites of tryptophan (from dairy products,

	fish, poultry, cereals, peanuts, chocolate, and prunes), made by intestinal bacteria or tissue enzymes, stimulate anti-inflammatory Nfr2 [386] and AhR pathways, strengthening intestinal integrity [330,387,388]. Nevertheless, excess protein intake may contribute indirectly when associated with caloric excess, leading to obesity, adipose expansion, macrophage infiltration, and cytokine production [385]. Some amino acids in excess (e.g., leucine-rich diets) can activate nutrient-sensing pathways, most notably mTORC1, but also PI3K–Akt and MAPK signaling, thus contributing to metabolic stress and reduced autophagy [389–392]. High-protein diets (especially animal protein) can modify gut microbiota, increase protein fermentation in the colon, and produce metabolites like ammonia, hydrogen sulfide, and p-cresol [368,389,393]. These may impair the gut barrier, increase endotoxin translocation, and TLR4 activation [368]. Particularly, animal proteins have higher inflammatory potential associated with possible endotoxin exposure, higher insulin-like growth factor 1 (IGF-1) secretion, and co-occurring higher saturated fat, cholesterol, and heme Fe intake, while plant proteins often co-occur with fiber, polyphenols, other plant antioxidants, and generally have an anti-inflammatory context [385,391,393–395]. Processed meats contain nitrites/nitrates, emulsifiers, high salt, which can disrupt epithelial tight junction proteins (occludins and claudins), and high saturated fat content (palmitic and stearic acid), shifting microbiota composition toward bile-tolerant and LPS-producing Gram-negative bacteria [366,396–398]. Heme Fe (particularly abundant in red meat) promotes oxidative stress in gut epithelium, increases lipid peroxidation, and damages the mucosal barrier [399–401]. Therefore, protein intakes are often confounded by other important pro- and anti-inflammatory nutrients [385,391,401], which we also confirmed in our study. For this reason, we cannot distinguish possible confounding effects associated with higher protein intakes in our study.
Dietary fiber, polyphenols, and micronutrients abundant in plant foods	In contrast, diets characterized by higher intakes of dietary fiber and polyphenols—typical of plant-based dietary patterns—may attenuate these inflammatory pathways [318,329,337,393,402–404]. Gut microbiota composition and activity, influenced by dietary fiber and polyphenols, affect intestinal epithelial integrity and immune cell function, thereby modifying intestinal permeability [330,362,397,398]. Low-fiber diets increase LPS translocation, leading to TLR4 activation, while high-fiber diets increase SCFAs production, which exert anti-inflammatory effects [271,362,398,405]. Fiber-derived SCFAs (particularly butyrate) enhance mucus layer production, improve gut barrier integrity, and reduce endotoxemia. In addition, they directly modulate immune signaling by activating GPRs and inhibiting HDACs, thereby suppressing NF-κB, MAPK/AP-1, mTORC1, and NLRP3 inflammasome signaling, while promoting activation of the Nrf2 and AhR pathways [268–271,273,330,337,345,373]. Furthermore, several micronutrients abundant in plant foods, including Mg, folate, carotenoids, vitamin C, vitamin E, and various polyphenols, may additionally support antioxidant defense systems and directly modulate inflammatory pathways such as Nrf2, NF-κB, MAPK, mTORC, and inflammasome signaling [318,329,337,345,402,403].
Different food groups that were different between the two patterns and/or associated with cytokine levels	Meat intakes are associated with fat intakes (particularly saturated fat and, to a lesser extent, monounsaturated and *n*-6 polyunsaturated, i.e., arachidonic acid), cholesterol, heme Fe, and protein intakes. Egg and milk intakes are associated with saturated fat, cholesterol, and protein intakes. Added fat intakes are associated with total energy intake and increased n-6 fat (in the case of vegetable sunflower oil), monounsaturated fat (in the case of olive oil), n-3 fat (in the case of flaxseed oil), saturated fat (in the case of lard, margarine, palm oil), or trans fat intakes (in the case of margarines or fried food). High fruit and vegetable intake is associated with high content of dietary fiber, many antioxidant and anti-inflammatory vitamins (β-carotene, vitamin C), minerals, polyphenols, and low caloric and fat content. Nuts and seeds are associated with high fiber, antioxidants (Se and vitamin E), n-3 (ALA), monounsaturated fats, and non-heme proteins. Sweets, usually, contain not only high sugar content, but also high fat

	content (particularly saturated and *trans*-fats), have high energy density, but are usually low in fiber, polyphenols and some vitamins (e.g., vitamins C and D, retinol). Notably, in our study, we also found all those associations between different dietary factors and specific nutrients. We also found some inverse associations of, e.g., meat intake with dietary fiber, polyphenols, Mg, K, Fe, Cu, β-carotene, folate, and vitamin C intakes (apart from positive associations of meat intake with energy intake and almost all other analyzed macro- and micronutrients). In addition, fish intake was very low in our study and, by definition, was reported only among participants following an omnivorous dietary pattern. Consequently, fish intake was positively correlated with the intake of eggs, milk, and meat, as well as with cholesterol and saturated fat. For these reasons, the independent associations of isolated dietary factors could not be distinguished in this cross-sectional study, and the observed associations may be partly attributable to confounding by other correlated dietary factors.

Supplementary Section S4.3. Overview of the Biological Roles of the Analyzed Cytokines and Chemokines.

The following supplementary section provides concise information regarding the biological role of the analyzed cytokines/chemokines, including their cellular sources, activation pathways, signaling mechanisms, role in immunity, secretion kinetics, and circulating half-life.

1. IL-1β [143,147,406,407,148,154–156,170–172,370]	IL-1β is a prototypical pro-inflammatory cytokine of the IL-1 family produced mainly by innate immune cells, including monocytes, macrophages, dendritic cells, and neutrophils. Unlike most cytokines, IL-1β is synthesized as an inactive precursor (pro-IL-1β) that requires proteolytic cleavage by caspase-1 following inflammasome activation to generate the biologically active mature form. Its production is induced by microbial components, particularly LPS and other TLR ligands, as well as by endogenous danger signals such as ATP, oxidative stress, mitochondrial dysfunction, and tissue injury. IL-1β expression is primarily regulated through NF-κB-dependent transcription, whereas its maturation and secretion depend mainly on NLRP3 and other inflammasomes. IL-1β acts predominantly locally through IL-1 receptor 1 (IL-1R1) signaling, amplifying inflammatory responses by inducing the production of IL-6, TNF-α, IFN-γ, chemokines, and promoting Th17 differentiation and IL-17A secretion in cooperation with IL-6 and IL-23. Circulating IL-1β is tightly regulated by IL-1 receptor antagonist (IL-1RA) and soluble IL-1 receptors (sIL-1R), and although IL-1β may remain detectable in circulation for 6–12 h or longer following experimental LPS administration in humans, it is affected by rapid soluble receptor binding and inactivation. After LPS stimulation, its levels are increased between 1–6 h, with a peak after 2–4 h. However, human meal challenge studies have reported inconsistent results, ranging from a decrease, no change, to an increase. Some studies have shown a prolonged increase for up to 8 h or longer, depending on the meal.
2. IL-18 [132,139,175–177,408,409,156,161–163,168,169,173,174]	IL-18 is another IL-1 family cytokine produced mainly by monocytes, macrophages, dendritic cells, and epithelial cells. Similar to IL-1β, IL-18 is synthesized as an inactive precursor (pro-IL-18) that requires cleavage by inflammasome-associated caspase-1 for activation and secretion. IL-18 production is induced by microbial signals, including LPS and other pattern recognition receptor ligands, as well as metabolic and cellular stress signals such as oxidative stress, mitochondrial dysfunction, and altered lipid metabolism. Its expression requires NF-κB-dependent transcriptional priming, while maturation is controlled primarily by NLRP3 and other inflammasomes. IL-18 is a potent inducer of IFN-γ production by NK cells and T cells, particularly in combination with IL-12, and contributes to Th1 immune responses and inflammatory amplification. In circulation, IL-18 activity is strongly regulated by IL-18 binding protein (IL-18BP), which binds free IL-18 and limits receptor activation. Although the reported IL-18 circulating persistence varied after stimulation (e.g., surgical trauma) or infusion of human recombinant IL-18, its biological activity depends largely on the balance between free IL-18 and IL-18BP-bound forms. While in mice, LPS stimulation managed to increase circulating IL-18, studies in humans, however, failed to show circulating increases after LPS stimulation. Meal challenge in humans produced inconsistent results, with some studies showing modestly increased circulating levels 1–6 h after stimulation.
3. IL-33 [120,149,182,184,186–190,410,411]	IL-33 is another IL-1 family cytokine that functions primarily as an alarmin released following cellular stress, tissue injury, or necrosis. It is constitutively expressed in the nuclei of structural cells, particularly epithelial cells, endothelial cells, fibroblasts, and stromal cells, with lower expression in immune cells. IL-33 expression is induced by inflammatory stimuli, including LPS,

	primarily through TLR4-mediated activation of NF-κB and MAPK/AP-1 signaling pathways. In contrast to classical pro-inflammatory cytokines, however, IL-33 is predominantly retained intracellularly as a nuclear alarmin and is generally released extracellularly only following cellular stress or tissue damage rather than through conventional secretion. IL-33 release is triggered by mechanical damage, allergens, infections, oxidative stress, and tissue injury rather than classical immune cell activation. Once released, IL-33 binds the IL-1 receptor-like 1 (IL1RL1, also known as ST2), which has two major isoforms: a transmembrane form (ST2 or ST2L) and a soluble form (sST2). ST2L forms a heterodimer with the IL-1 receptor accessory protein (IL-1RAcP), and activates MyD88-dependent signaling, leading to NF-κB, MAPK, and PI3K–Akt /mTOR activation. IL-33 promotes type 2 immune responses by stimulating group 2 innate lymphoid cells (ILC2s), Th2 cells, mast cells, basophils, eosinophils, Treg cells, NK cells, granulocytes, and alternatively activated macrophages, inducing production of IL-5, IL-4, IL-13, IL-10, IL-6, IL-17, IL-8, MCP-1, while downregulating secretion of IL-1β and IL-6, under LPS-stimulated conditions. Therefore, IL-33 possesses both proinflammatory and antiinflammatory properties. sST2 circulates in the blood as a decoy receptor, trapping free IL-33 and blocking it from reaching its receptors. sST2 secretion follows after IL-33 secretion but remains more prolonged. Circulating IL-33 is highly unstable and rapidly (within 2 h) inactivated by oxidation, proteolytic cleavage, and trapping by sST2, with a half-life ~1.5 h. In cell culture of monocytes, after LPS stimulation, its expression begins after 3 h, staying prolonged (up to 48 h and more), with a peak about 6–9 h after stimulation. However, in humans, after LPS stimulation, serum IL-33 did not change, but sST2 response showed a marked delayed rise, staying elevated even 24 hours after LPS exposure. The only available meal-challenge study did not detect differences in circulating IL-33 concentrations.
4. TNF-α [127,143,412–416,150,154,156,158,170,180,370,407]	TNF-α is a major pro-inflammatory cytokine produced predominantly by activated macrophages and monocytes, but also by dendritic cells, T cells, NK cells, neutrophils, and other immune and stromal cells. Its production is induced by microbial components, particularly LPS through TLR4 activation, as well as by TNF receptor signaling, oxidative stress, tissue injury, and metabolic stress. TNF-α expression is regulated mainly through NF-κB and MAPK/AP-1 pathways. It acts through its receptors, TNFR1 and TNFR2, while inhibitory proteins and soluble TNF receptors (sTNF-Rs) in plasma can block its effects. TNF-α promotes the production of IL-6, IL-8, MCP-1, and other inflammatory mediators, while also contributing to Th1 immune responses. Its circulating half-life is short after LPS stimulation, about 1 h. It starts to increase after 30 min, peaks after 1.5–2 h, and falls down after 3–4 h. Following meal stimulation, TNF-α also rapidly increases, peaking within 2–3 h, although, depending on the type of meal, the effect can last for more than 8 h. Nevertheless, some studies showed even a decrease after meal stimulation.
5. IL-6 [130,150,407,414–419,154,158,170–172,180,193,370]	IL-6 is a pleiotropic cytokine produced by monocytes, macrophages, dendritic cells, endothelial cells, fibroblasts, skeletal muscle cells, and adipocytes. Its production is stimulated by microbial components, particularly LPS, as well as TNF-α, IL-1β, tissue injury, exercise, and metabolic stress. IL-6 expression is regulated mainly through NF-κB, MAPK/AP-1, and signal transducer and activator of transcription 3 (STAT3)-associated pathways. IL-6 signals through membrane-bound or soluble IL-6 receptor complexes, activating Janus kinase(JAK)/STAT3 signaling and regulating acute-phase responses, immune cell differentiation, metabolism, and tissue repair. In adaptive immunity, IL-6 cooperates with IL-1β to promote human Th17 differentiation, with IL-23 supporting subsequent expansion and stabilization of Th17 cells. A soluble IL-6 receptor (sIL-6R) also binds the circulating IL-6, but

	unlike other soluble cytokine receptors, it does not inhibit and can even increase IL-6 biological activity. After LPS stimulation, IL-6 circulating levels are increased after 1–6 h, with a peak around 2–4 h. After meal stimulation, serum IL-6 increases after 2–3 h, typically reaching peak concentrations at 2–4 h, and falling down after 6 h, but, depending on the type of meal, the effect can last for even more than 8 h.
6. IL-12 [143,156,407,420–425]	IL-12 (or IL-12p70, the biologically active heterodimeric form of IL-12, composed of p35 and p40 subunits), belongs to the IL-12 cytokine family. It is produced predominantly by activated DCs, monocytes, and macrophages in response to microbial stimulation, particularly through TLR activation by LPS, bacterial DNA, and other pathogen-associated molecular patterns. IL-12 p35 and p40 production is regulated mainly through NF-κB, IRF, and MAPK/AP-1 signaling pathways and is enhanced by IFN-γ, creating a positive feedback loop during innate immune activation. IL-12 binds to the IL-12 receptor complex (IL-12Rβ1/IL-12Rβ2) and activates Janus kinase 2/tyrosine kinase 2–signal transducer and activator of transcription 4 (JAK2/TYK2–STAT4) signaling, promoting differentiation and activation of Th1 cells and NK cells and stimulating production of IFN-γ. Consequently, IL-12 plays a central role in cellular immunity against intracellular pathogens and tumor surveillance. After LPS stimulation, its circulating concentrations modestly increase within 2–6 h. After meal stimulation, circulating levels are increased for 6 h, but the effect can last for even more than 8 h, depending on the meal.
7. IL-23 [192,193,420,421,426–428]	IL-23 is another heterodimeric cytokine of the IL-12 family, composed of a unique p19 subunit and a p40 subunit shared with IL-12. It is produced predominantly by innate immune cells, including activated DCs, monocytes, macrophages, and, to a lesser extent, neutrophils and epithelial cells, in response to microbial products and inflammatory stimuli. p19 IL-23 production by monocytes, macrophages, and DCs is induced primarily through TLR-mediated activation of NF-κB and MAPK/AP-1 signaling pathways, with CCAAT/enhancer-binding protein β (C/EBPβ) further enhancing transcription. Its expression is enhanced also by cytokines, including IL-1β and TNF-α, and supported by the PI3K–Akt/mTORC1 pathway. Unlike IL-12, IL-23 is not a major inducer of naïve T-cell differentiation but primarily functions to maintain, expand, and stabilize Th17 cells. Through binding to the IL-23 receptor complex (IL-23R/IL-12Rβ1), it activates JAK2/TYK2–STAT3 signaling, promoting production of IL-17A, IL-17F, IL-22, and granulocyte–macrophage colony-stimulating factor (GM-CSF). Thus, IL-23 contributes to host defense against extracellular bacteria and fungi, whereas excessive IL-23 signaling promotes chronic inflammatory diseases, including psoriasis, inflammatory bowel disease, ankylosing spondylitis, and multiple sclerosis. Following LPS stimulation, IL-23 expression becomes detectable approximately 2–4 h after stimulation, peaks around 8 h, and remains elevated for at least 16 h. In human meal-challenge studies, circulating IL-23 has been reported to increase from ~2 h after meal ingestion, remain elevated after 4 h, but the subsequent time course and duration of the response remain unknown. However, even inconsistent findings were reported, with decreased levels after meal stimulation.
8. IL-17A [173,193,416,426–433]	IL-17A is the prototypical cytokine of the IL-17 family and is produced predominantly by Th17 CD4⁺ T cells, but also by γδ T cells, invariant NK T cells, innate lymphoid cells, and neutrophils under specific inflammatory conditions. IL-17A production is induced mainly by cytokine-driven differentiation and activation of Th17 cells, requiring coordinated signaling by IL-6 and IL-1β for differentiation, and IL-23 for expansion and stabilization. Its expression is regulated through the transcription factor RORγt, with additional regulation by STAT3 and NF-κB signaling. IL-17A binds to IL-17 receptor complexes (IL-17RA/IL-17RC), activating TNF receptor-associated factor 3

	(TRAF3)-interacting protein 2 (TRAF3IP2)-dependent signaling and downstream NF-κB, MAPK, and CCAAT/enhancer-binding protein (C/EBP) pathways. This induces production of chemokines (including IL-8), G-CSF, antimicrobial peptides, and inflammatory mediators that promote neutrophil recruitment and mucosal defense. IL-17A plays an important role in protection against extracellular bacteria and fungi but contributes to chronic inflammatory diseases when dysregulated. After LPS stimulation in humans, serum IL-17 levels can be increased for more than 24 h, with a peak at ~12 h. Following meal ingestion, serum IL-17A has been reported to increase after ~1 h and persist for more than 6–8 h. However, also decreases have also been reported, depending on the type of meal.
9. IFN-γ [133,143,156,159,407,434]	IFN-γ is the major type II interferon and is produced primarily by activated NK cells and T lymphocytes, particularly Th1 CD4+ T cells and cytotoxic CD8+ T cells, with additional production by innate lymphoid cells. Its secretion is induced by innate immune activation, particularly by IL-12 and IL-18 produced by DCs and macrophages, as well as by antigen stimulation through T-cell receptors. IFN-γ production is regulated mainly through STAT4- and T-bet-dependent transcriptional programs, with additional modulation by NF-κB and AP-1 pathways. Through binding to the IFN-γ receptor (IFNGR), it activates JAK1/JAK2–STAT1 signaling, promoting macrophage activation, antigen presentation, major histocompatibility complex (MHC) expression, and Th1 immune responses. IFN-γ is essential for defense against intracellular pathogens and tumor immune surveillance but may contribute to chronic inflammatory conditions when persistently activated. After LPS stimulation, IFN-γ levels are increased within 1–6 h, with a peak after 1.5–4 h. After meal stimulation, the effect can persist even after 8 h.
10. IFN-α2 [143,435–438]	IFN-α2 is a type I interferon produced predominantly by pDCs, but also by monocytes, macrophages, cDCs, and other nucleated cells in response to viral and microbial stimuli. Its production is primarily induced by activation of intracellular nucleic acid-sensing pattern recognition receptors, including TLR7 and TLR9. IFN-α2 expression is regulated mainly through IRF7 and IRF3 activation, with additional involvement of NF-κB signaling. Following secretion, IFN-α2 binds to the type I interferon receptor (IFNAR), activating JAK1/TYK2–STAT1/STAT2 signaling and inducing interferon-stimulated genes that promote antiviral defense, immune cell activation, and modulation of innate and adaptive immunity. Type I interferons also influence antigen presentation, NK-cell activity, and T-cell differentiation. After LPS stimulation, serum IFN-α2 levels can be increased ~24 h, with a peak at ~5 h. After meal stimulation, the effect can also persist after 8 h.
11. IL-10 [123,143,154,299,407,424,433,439,440]	IL-10 is a major anti-inflammatory cytokine produced by both innate and adaptive immune cells, including regulatory macrophages, dendritic cells, monocytes, regulatory T cells, Th1 cells, B cells, and NK cells. Its production is induced by microbial stimulation, particularly LPS and TLR activation, as well as by IL-2, IL-12, IFN-γ, immune complexes, and resolution-phase signals. IL-10 expression is regulated through multiple transcription factors, including STAT3, c-Maf, Sp1, and AP-1, with additional modulation by NF-κB-dependent pathways. IL-10 signals through the IL-10 receptor complex, activating JAK1/TYK2 and STAT3 signaling, resulting in suppression of excessive inflammatory responses by inhibiting NF-κB activity, antigen presentation, and production of pro-inflammatory cytokines such as TNF-α, IL-6, IL-1β, and IL-12. It promotes immune tolerance and resolution of inflammation while maintaining host defense. IL-10 production generally follows inflammatory activation as a regulatory feedback mechanism, often detectable several hours after stimulation. After LPS stimulation, its concentrations are increased from 1–6 h, with a peak at ~3 h. Although it has kinetics similar to IL-6 and IL-8, increasing within 1–6 h, its peak is slightly delayed. After meal

	stimulation, its circulating levels are usually increased 2–4 h , although the effect can persist even after 8 h.

References

(Including references from the main manuscript)

1. Bacaloni, S.; Agrawal, D.K. Nutrition, Gut Microbiota, and Epigenetics in the Modulation of Immune Response and Metabolic Health. *Cardiol. Cardiovasc. Med.* **2025**, *9*, 111–124.
2. Yu, X.; Pu, H.; Voss, M. Overview of Anti-Inflammatory Diets and Their Promising Effects on Non-Communicable Diseases. *Br. J. Nutr.* **2024**, *132*, 898–918, doi:10.1017/S0007114524001405.
3. Christ, A.; Lauterbach, M.; Latz, E. Western Diet and the Immune System: An Inflammatory Connection. *Immunity* **2019**, *51*, 794–811, doi:10.1016/j.immuni.2019.09.020.
4. Chaari, A.; Bendriss, G.; Zakaria, D.; McVeigh, C. Importance of Dietary Changes During the Coronavirus Pandemic: How to Upgrade Your Immune Response. *Front. Public Heal.* **2020**, *8*, 476, doi:10.3389/fpubh.2020.00476.
5. Childs, C.E.; Calder, P.C.; Miles, E.A. Diet and Immune Function. *Nutrients* 2019, *11*.
6. Calder, P.C.; Carr, A.C.; Gombart, A.F.; Eggersdorfer, M. Optimal Nutritional Status for a Well-Functioning Immune System Is an Important Factor to Protect against Viral Infections. *Nutrients* 2020, *12*, 1181.
7. Elmadfa, I.; Meyer, A.L. The Role of the Status of Selected Micronutrients in Shaping the Immune Function. *Endocr. Metab. Immune Disord. Drug Targets* **2019**, *19*, 1100–1115, doi:10.2174/1871530319666190529101816.
8. Springmann, M.; Wiebe, K.; Mason-D'Croz, D.; Sulser, T.B.; Rayner, M.; Scarborough, P. Health and Nutritional Aspects of Sustainable Diet Strategies and Their Association with Environmental Impacts: A Global Modelling Analysis with Country-Level Detail. *Lancet Planet. Heal.* **2018**, *2*, e451–e461, doi:10.1016/S2542-5196(18)30206-7.
9. Selinger, E.; Neuenschwander, M.; Koller, A.; Gojda, J.; Kühn, T.; Schwingshackl, L.; Barbaresko, J.; Schlesinger, S. Evidence of a Vegan Diet for Health Benefits and Risks - an Umbrella Review of Meta-Analyses of Observational and Clinical Studies. *Crit. Rev. Food Sci. Nutr.* **2023**, *63*, 9926–9936, doi:10.1080/10408398.2022.2075311.
10. Oussalah, A.; Levy, J.; Berthezène, C.; Alpers, D.H.; Guéant, J.-L. Health Outcomes Associated with Vegetarian Diets: An Umbrella Review of Systematic Reviews and Meta-Analyses. *Clin. Nutr.* **2020**, *39*, 3283–3307, doi:10.1016/j.clnu.2020.02.037.
11. Derbyshire, E.J. Flexitarian Diets and Health: A Review of the Evidence-Based Literature. *Front. Nutr.* **2017**, *3*, doi:10.3389/fnut.2016.00055.
12. Herpich, C.; Müller-Werdan, U.; Norman, K. Role of Plant-Based Diets in Promoting Health and Longevity. *Maturitas* **2022**, *165*, 47–51, doi:10.1016/j.maturitas.2022.07.003.
13. Wang, T.; Masedunskas, A.; Willett, W.C.; Fontana, L. Vegetarian and Vegan Diets: Benefits and Drawbacks. *Eur. Heart J.* **2023**, *44*, 3423–3439, doi:10.1093/eurheartj/ehad436.
14. Kent, G.; Kehoe, L.; Flynn, A.; Walton, J. Plant-Based Diets: A Review of the Definitions and Nutritional Role in the Adult Diet. *Proc. Nutr. Soc.* **2022**, *81*, 62–74, doi:10.1017/S0029665121003839.
15. Šarac, I.; Milešević, J.; Knez, M.; Despotović, M.; Takić, M.; Debeljak-Martačić, J.; Zeković, M.; Kadvan, A.; Gurinović, M. Energy and Macronutrient Dietary Intakes of Vegetarian and Semi-Vegetarian Serbian Adults: Data from the EFSA EU Menu Food Consumption Survey (2017-2022). *Foods (Basel, Switzerland)* **2025**, *14*, doi:10.3390/foods14081285.
16. Agnoli, C.; Baroni, L.; Bertini, I.; Ciappellano, S.; Fabbri, A.; Papa, M.; Pellegrini, N.; Sbarbati, R.; Scarino, M.L.; Siani, V.; et al. Position Paper on Vegetarian Diets from the Working Group of the Italian Society of Human Nutrition. *Nutr. Metab. Cardiovasc. Dis.* **2017**, *27*, 1037–1052, doi:10.1016/j.numecd.2017.10.020.
17. Bakaloudi, D.R.; Halloran, A.; Rippin, H.L.; Oikonomidou, A.C.; Dardavesis, T.I.; Williams, J.; Wickramasinghe, K.; Breda, J.; Chourdakis, M. Intake and Adequacy of the Vegan Diet. A Systematic Review of the Evidence. *Clin. Nutr.* **2021**, *40*, 3503–3521, doi:10.1016/j.clnu.2020.11.035.

18. Rogerson, D. Vegan Diets: Practical Advice for Athletes and Exercisers. *J. Int. Soc. Sports Nutr.* **2017**, *14*, 36, doi:10.1186/s12970-017-0192-9.
19. Gajski, G.; Gerić, M.; Vučić Lovrenčić, M.; Božičević, S.; Rubelj, I.; Nanić, L.; Škrobot Vidaček, N.; Bendix, L.; Peraica, M.; Rašić, D.; et al. Analysis of Health-Related Biomarkers between Vegetarians and Non-Vegetarians: A Multi-Biomarker Approach. *J. Funct. Foods* **2018**, *48*, 643–653, doi:10.1016/j.jff.2018.07.054.
20. Neufingerl, N.; Eilander, A. Nutrient Intake and Status in Adults Consuming Plant-Based Diets Compared to Meat-Eaters: A Systematic Review. *Nutrients* **2021**, *14*, doi:10.3390/nu14010029.
21. Lynch, H.; Johnston, C.; Wharton, C. Plant-Based Diets: Considerations for Environmental Impact, Protein Quality, and Exercise Performance. *Nutrients* **2018**, *10*, doi:10.3390/nu10121841.
22. Soh, B.X.P.; Smith, N.W.; von Hurst, P.R.; McNabb, W.C. Achieving High Protein Quality Is a Challenge in Vegan Diets: A Narrative Review. *Nutr. Rev.* **2024**, nuae176, doi:10.1093/nutrit/nuae176.
23. Abeyrathne, E.D.N.S.; Nam, K.; Huang, X.; Ahn, D.U. Plant- and Animal-Based Antioxidants' Structure, Efficacy, Mechanisms, and Applications: A Review. *Antioxidants (Basel, Switzerland)* **2022**, *11*, doi:10.3390/antiox11051025.
24. Salim, R.; Nehvi, I.B.; Mir, R.A.; Tyagi, A.; Ali, S.; Bhat, O.M. A Review on Anti-Nutritional Factors: Unraveling the Natural Gateways to Human Health. *Front. Nutr.* **2023**, *10*, 1215873, doi:10.3389/fnut.2023.1215873.
25. Craddock, J.C.; Neale, E.P.; Peoples, G.E.; Probst, Y.C. Vegetarian-Based Dietary Patterns and Their Relation with Inflammatory and Immune Biomarkers: A Systematic Review and Meta-Analysis. *Adv. Nutr.* **2019**, *10*, 433–451, doi:10.1093/advances/nmy103.
26. Menzel, J.; Jabakhanji, A.; Biemann, R.; Mai, K.; Abraham, K.; Weikert, C. Systematic Review and Meta-Analysis of the Associations of Vegan and Vegetarian Diets with Inflammatory Biomarkers. *Sci. Rep.* **2020**, *10*, 21736, doi:10.1038/s41598-020-78426-8.
27. Menzel, J.; Biemann, R.; Longree, A.; Isermann, B.; Mai, K.; Schulze, M.B.; Abraham, K.; Weikert, C. Associations of a Vegan Diet with Inflammatory Biomarkers. *Sci. Rep.* **2020**, *10*, 1933, doi:10.1038/s41598-020-58875-x.
28. Eichelmann, F.; Schwingshackl, L.; Fedirko, V.; Aleksandrova, K. Effect of Plant-Based Diets on Obesity-Related Inflammatory Profiles: A Systematic Review and Meta-Analysis of Intervention Trials. *Obes. Rev.* **2016**, *17*, 1067–1079, doi:10.1111/obr.12439.
29. Jaceldo-Siegl, K.; Haddad, E.; Knutsen, S.; Fan, J.; Lloren, J.; Bellinger, D.; Fraser, G.E. Lower C-Reactive Protein and IL-6 Associated with Vegetarian Diets Are Mediated by BMI. *Nutr. Metab. Cardiovasc. Dis.* **2018**, *28*, 787–794, doi:10.1016/j.numecd.2018.03.003.
30. Carolina, A.; Moraes, F. De; Pititto, B.D.A.; Fernandes, R.; Gomes, E.P.; Pereira, C.; Ferreira, S.R.G. Worse Inflammatory Profile in Omnivores than in Vegetarians Associates with the Gut Microbiota Composition. *Diabetol. Metab. Syndr.* **2017**, 1–8, doi:10.1186/s13098-017-0261-x.
31. Smidowicz, A.; Regula, J. Effect of Nutritional Status and Dietary Patterns on Human Serum C-Reactive Protein and Interleukin-6 Concentrations. *Adv. Nutr.* **2015**, *6*, 738–747, doi:10.3945/an.115.009415.
32. Tanaka, T.; Kouda, K.; Kotani, M.; Takeuchi, A.; Tabei, T.; Masamoto, Y.; Nakamura, H.; Takigawa, M.; Suemura, M.; Takeuchi, H.; et al. Vegetarian Diet Ameliorates Symptoms of Atopic Dermatitis through Reduction of the Number of Peripheral Eosinophils and of PGE2 Synthesis by Monocytes. *J. Physiol. Anthropol. Appl. Human Sci.* **2001**, *20*, 353–361, doi:10.2114/jpa.20.353.
33. Montalcini, T.; De Bonis, D.; Ferro, Y.; Carè, I.; Mazza, E.; Accattato, F.; Greco, M.; Foti, D.; Romeo, S.; Gulletta, E.; et al. High Vegetable Fats Intake Is Associated with High Resting Energy Expenditure in Vegetarians. *Nutrients* **2015**, *7*, 5933–5947, doi:10.3390/nu7075259.
34. Lee, Y.-J.; Wang, M.-Y.; Lin, M.-C.; Lin, P.-T. Associations between Vitamin B-12 Status and Oxidative Stress and Inflammation in Diabetic Vegetarians and Omnivores. *Nutrients* **2016**, *8*, 118, doi:10.3390/nu8030118.
35. Haddad, E.H.; Berk, L.S.; Kettering, J.D.; Hubbard, R.W.; Peters, W.R. Dietary Intake and Biochemical, Hematologic, and

Immune Status of Vegans Compared with Nonvegetarians. *Am. J. Clin. Nutr.* **1999**, *70*, 586S-593S, doi:10.1093/ajcn/70.3.586s.
36. Link, L.B.; Hussaini, N.S.; Jacobson, J.S. Change in Quality of Life and Immune Markers after a Stay at a Raw Vegan Institute: A Pilot Study. *Complement. Ther. Med.* **2008**, *16*, 124–130, doi:10.1016/j.ctim.2008.02.004.
37. Lederer, A.-K.; Maul-Pavicic, A.; Hannibal, L.; Hettich, M.; Steinborn, C.; Gründemann, C.; Zimmermann-Klemd, A.M.; Müller, A.; Sehnert, B.; Salzer, U.; et al. Vegan Diet Reduces Neutrophils, Monocytes and Platelets Related to Branched-Chain Amino Acids - A Randomized, Controlled Trial. *Clin. Nutr.* **2020**, *39*, 3241–3250, doi:10.1016/j.clnu.2020.02.011.
38. Neubauerova, E.; Tulinska, J.; Lehotska Mikusova, M.; Liskova, A.; Volkovova, K.; Kudlackova, M. The Effect of Vegetarian Diet on Immune Response. In Proceedings of ISEE 2007 Conference, Mexico City, Mexico. In Proceedings of the Epidemiology. ISEE 2007 Conference Abstract Supplement.; September 1 2007; Vol. 18, p. S196.
39. Zhang, C.; Björkman, A.; Cai, K.; Liu, G.; Wang, C.; Li, Y.; Xia, H.; Sun, L.; Kristiansen, K.; Wang, J.; et al. Impact of a 3-Months Vegetarian Diet on the Gut Microbiota and Immune Repertoire. *Front. Immunol.* **2018**, *Volume 9*, 908.
40. Malter, M.; Schriever, G.; Eilber, U. Natural Killer Cells, Vitamins, and Other Blood Components of Vegetarian and Omnivorous Men. *Nutr. Cancer* **1989**, *12*, 271–278, doi:10.1080/01635588909514026.
41. Haghighatdoost, F.; Bellissimo, N.; Totosy de Zepetnek, J.O.; Rouhani, M.H. Association of Vegetarian Diet with Inflammatory Biomarkers: A Systematic Review and Meta-Analysis of Observational Studies. *Public Health Nutr.* **2017**, *20*, 2713–2721, doi:10.1017/S1368980017001768.
42. Ganie, M.A.; Sahar, T.; Rashid, A.; Wani, I.A.; Nisar, S.; Sathyapalan, T.; Vishnubhatla, S.; Ramakrishnan, L.; Parvez, T.; Geer, I. Comparative Evaluation of Biomarkers of Inflammation Among Indian Women With Polycystic Ovary Syndrome (PCOS) Consuming Vegetarian vs. Non-Vegetarian Diet. *Front. Endocrinol. (Lausanne).* **2019**, *Volume 10*.
43. Klein, L.; Lenz, C.; Krüger, K.; Lorkowski, S.; Kipp, K.; Dawczynski, C. Comparative Analysis of Fatty Acid Profiles across Omnivorous, Flexitarians, Vegetarians, and Vegans: Insights from the NuEva Study. *Lipids Health Dis.* **2025**, *24*, 133, doi:10.1186/s12944-025-02517-6.
44. Hargreaves, S.M.; Rosenfeld, D.L.; Moreira, A.V.B.; Zandonadi, R.P. Plant-Based and Vegetarian Diets: An Overview and Definition of These Dietary Patterns. *Eur. J. Nutr.* **2023**, *62*, 1109–1121, doi:10.1007/s00394-023-03086-z.
45. Storz, M.A. What Makes a Plant-Based Diet? A Review of Current Concepts and Proposal for a Standardized Plant-Based Dietary Intervention Checklist. *Eur. J. Clin. Nutr.* **2022**, *76*, 789–800, doi:10.1038/s41430-021-01023-z.
46. Zeković, M.; Gurinović, M.; Milešević, J.; Kadvan, A.; Glibetić, M.; Institute for Medical Research (IMR) University of Belgrade National Food Consumption Survey among 10 – 74 Years Old Individuals in Serbia. *EFSA Support. Publ.* **2022**, *19*, 7401E, doi:10.2903/sp.efsa.2022.EN-7401.
47. Wawrzyniak-Gramacka, E.; Hertmanowska, N.; Tylutka, A.; Morawin, B.; Wacka, E.; Gutowicz, M.; Zembron-Lacny, A. The Association of Anti-Inflammatory Diet Ingredients and Lifestyle Exercise with Inflammaging. *Nutrients* **2021**, *13*, doi:10.3390/nu13113696.
48. Pearson, T.A.; Mensah, G.A.; Hong, Y.; Smith, S.C.J. CDC/AHA Workshop on Markers of Inflammation and Cardiovascular Disease: Application to Clinical and Public Health Practice: Overview. *Circulation* 2004, *110*, e543-4.
49. Pearson, T.A.; Mensah, G.A.; Alexander, R.W.; Anderson, J.L.; Cannon, R.O.; Criqui, M.; Fadl, Y.Y.; Fortmann, S.P.; Hong, Y.; Myers, G.L.; et al. Markers of Inflammation and Cardiovascular Disease: Application to Clinical and Public Health Practice: A Statement for Healthcare Professionals From the Centers for Disease Control and Prevention and the American Heart Association. *Circulation* **2003**, *107*, 499–511, doi:10.1161/01.CIR.0000052939.59093.45.
50. Ali, S.; Zehra, A.; Khalid, M.U.; Hassan, M.; Shah, S.I.A. Role of C-Reactive Protein in Disease Progression, Diagnosis and Management. *Discov. (Craiova, Rom.* **2023**, *11*, e179, doi:10.15190/d.2023.18.
51. Clifton, L.; Birks, J.; Clifton, D.A. Comparing Different Ways of Calculating Sample Size for Two Independent Means: A Worked Example. *Contemp. Clin. trials Commun.* **2019**, *13*, 100309, doi:10.1016/j.conctc.2018.100309.
52. Livingstone, M.B.E.; Black, A.E. Markers of the Validity of Reported Energy Intake. *J. Nutr.* **2003**, *133 Suppl*, 895S-920S, doi:10.1093/jn/133.3.895S.

53. Black, A.E. Critical Evaluation of Energy Intake Using the Goldberg Cut-off for Energy Intake:Basal Metabolic Rate. A Practical Guide to Its Calculation, Use and Limitations. *Int. J. Obes. Relat. Metab. Disord. J. Int. Assoc. Study Obes.* **2000**, *24*, 1119–1130, doi:10.1038/sj.ijo.0801376.

54. World Health Organization *Waist Circumference and Waist-Hip Ratio : Report of a WHO Expert Consultation, Geneva, 8-11 December 2008*; World Health Organization, 2011; ISBN 9789241501491.

55. Ashwell, M.; Gunn, P.; Gibson, S. Waist-to-Height Ratio Is a Better Screening Tool than Waist Circumference and BMI for Adult Cardiometabolic Risk Factors: Systematic Review and Meta-Analysis. *Obes. Rev.* **2012**, *13*, 275–286, doi:10.1111/j.1467-789X.2011.00952.x.

56. Zhou, X.; Fragala, M.S.; McElhaney, J.E.; Kuchel, G.A. Conceptual and Methodological Issues Relevant to Cytokine and Inflammatory Marker Measurements in Clinical Research. *Curr. Opin. Clin. Nutr. Metab. Care* **2010**, *13*, 541–547, doi:10.1097/MCO.0b013e32833cf3bc.

57. Suzuki, K.; Nakaji, S.; Yamada, M.; Totsuka, M.; Sato, K.; Sugawara, K. Systemic Inflammatory Response to Exhaustive Exercise. Cytokine Kinetics. *Exerc. Immunol. Rev.* **2002**, *8*, 6–48.

58. Doumatey, A.P.; Zhou, J.; Adeyemo, A.; Rotimi, C. High Sensitivity C-Reactive Protein (Hs-CRP) Remains Highly Stable in Long-Term Archived Human Serum. *Clin. Biochem.* **2014**, *47*, 315–318, doi:10.1016/j.clinbiochem.2013.12.014.

59. de Jager, W.; Bourcier, K.; Rijkers, G.T.; Prakken, B.J.; Seyfert-Margolis, V. Prerequisites for Cytokine Measurements in Clinical Trials with Multiplex Immunoassays. *BMC Immunol.* **2009**, *10*, 52, doi:10.1186/1471-2172-10-52.

60. Lehmann, J.S.; Rughwani, P.; Kolenovic, M.; Ji, S.; Sun, B. Chapter Nine - LEGENDplex™: Bead-Assisted Multiplex Cytokine Profiling by Flow Cytometry. In *Tumor Immunology and Immunotherapy – Molecular Methods*; Galluzzi, L., Rudqvist, N.-P.B.T.-M. in E., Eds.; Academic Press, 2019; Vol. 629, pp. 151–176 ISBN 0076-6879.

61. BioLegend LEGENDplex™ Human Inflammation Panel 1 (13-Plex) Instruction Manual; Cat. No. 740809 Available online: www.biolegend.com/legendplex (accessed on 9 May 2026).

62. Jachimowicz, L.; Guenther, G.; Technologies, A. Extensive Assessment of Cytokine Production Following Immune Cell Stimulation on the NovoCyte Penteon Using the LEGENDplex Bead-Based Multiplex Assay.

63. Nikolić, M.; Milešević, J.; Zeković, M.; Gurinović, M.; Glibetić, M. The Development and Validation of Food Atlas for Portion Size Estimation in the Balkan Region. *Front. Nutr.* **2018**, *5*, 78, doi:10.3389/fnut.2018.00078.

64. Gurinović, M.; Milešević, J.; Kadvan, A.; Djekić-Ivanković, M.; Debeljak-Martačić, J.; Takić, M.; Nikolić, M.; Ranković, S.; Finglas, P.; Glibetić, M. Establishment and Advances in the Online Serbian Food and Recipe Data Base Harmonized with EuroFIR™ Standards. *Food Chem.* **2016**, *193*, 30–38, doi:10.1016/j.foodchem.2015.01.107.

65. Glibetic, M.; Kadvan, A.; Tepsic, J.; Martacic, J.D.; Djekic-Ivankovic, M.; Gurinovic, M. Management of Food Composition Database Harmonized with EuroFIR Criteria Using a Web Application. *J. Food Compos. Anal.* **2011**, *24*, 741–743, doi:10.1016/j.jfca.2010.09.002.

66. Gurinović, M.; Nikolić, M.; Zeković, M.; Milešević, J.; Kadvan, A.; Ranić, M.; Glibetić, M. Implementation of Harmonized Food Consumption Data Collection in the Balkan Region According to the EFSA EU Menu Methodology Standards. *Front. Nutr.* **2022**, *8*, doi:10.3389/fnut.2021.809328.

67. Hébert, J.R.; Shivappa, N.; Wirth, M.D.; Hussey, J.R.; Hurley, T.G. Perspective: The Dietary Inflammatory Index (DII)—Lessons Learned, Improvements Made, and Future Directions. *Adv. Nutr.* **2019**, *10*, 185–195, doi:10.1093/advances/nmy071.

68. Zeb, F.; Osaili, T.; Naqeeb, H.; Faris, M.E.; Cheikh Ismail, L.; Obaid, R.S.; Naja, F.; Radwan, H.; Hasan, H.; Hashim, M.; et al. Scientific Basis of Dietary Inflammatory Index (DII): A Dietary Tool to Metabolic Syndrome Risk. *Clin. Nutr. Open Sci.* **2025**, *61*, 138–161, doi:10.1016/j.nutos.2025.04.002.

69. Shivappa, N.; Steck, S.E.; Hurley, T.G.; Hussey, J.R.; Hébert, J.R. Designing and Developing a Literature-Derived, Population-Based Dietary Inflammatory Index. *Public Health Nutr.* **2014**, *17*, 1689–1696, doi:DOI: 10.1017/S1368980013002115.

70. Tabung, F.K.; Steck, S.E.; Zhang, J.; Ma, Y.; Liese, A.D.; Agalliu, I.; Hingle, M.; Hou, L.; Hurley, T.G.; Jiao, L.; et al. Construct Validation of the Dietary Inflammatory Index among Postmenopausal Women. *Ann. Epidemiol.* **2015**, *25*, 398–405,

doi:10.1016/j.annepidem.2015.03.009.
71. Tabung, F.K.; Steck, S.E.; Liese, A.D.; Zhang, J.; Ma, Y.; Caan, B.; Chlebowski, R.T.; Freudenheim, J.L.; Hou, L.; Mossavar-Rahmani, Y.; et al. Association between Dietary Inflammatory Potential and Breast Cancer Incidence and Death: Results from the Women's Health Initiative. *Br. J. Cancer* **2016**, *114*, 1277–1285, doi:10.1038/bjc.2016.98.
72. Pajunen, L.; Korkalo, L.; Koivuniemi, E.; Houttu, N.; Pellonperä, O.; Mokkala, K.; Shivappa, N.; Hébert, J.R.; Vahlberg, T.; Tertti, K.; et al. A Healthy Dietary Pattern with a Low Inflammatory Potential Reduces the Risk of Gestational Diabetes Mellitus. *Eur. J. Nutr.* **2022**, *61*, 1477–1490, doi:10.1007/s00394-021-02749-z.
73. Li, Q.; Chen, Y.; Yang, M.; Li, P. The Dietary Inflammatory Index (DII(®)) and Human Papillomavirus Infection: A Cross-Sectional Study of US Women. *BMC Public Health* **2024**, *24*, 3031, doi:10.1186/s12889-024-20490-0.
74. Shivappa, N.; Schoenaker, D.A.J.M.; Hebert, J.R.; Mishra, G.D. Association between Inflammatory Potential of Diet and Risk of Depression in Middle-Aged Women: The Australian Longitudinal Study on Women's Health. *Br. J. Nutr.* **2016**, *116*, 1077–1086, doi:DOI: 10.1017/S0007114516002853.
75. Petermann-Rocha, F.; Wirth, M.D.; Boonpor, J.; Parra-Soto, S.; Zhou, Z.; Mathers, J.C.; Livingstone, K.; Forrest, E.; Pell, J.P.; Ho, F.K.; et al. Associations between an Inflammatory Diet Index and Severe Non-Alcoholic Fatty Liver Disease: A Prospective Study of 171,544 UK Biobank Participants. *BMC Med.* **2023**, *21*, 123, doi:10.1186/s12916-023-02793-y.
76. Wang, C.; Wu, R.; Yao, D.; Yu, Z.; Shen, X. Comparison of Dietary Inflammatory Index and Inflammatory Biomarkers between Vegetarians and Omnivores in Chinese Population. *Sci. Rep.* **2024**, *14*, 19593, doi:10.1038/s41598-024-69168-y.
77. Zuercher, M.D.; Harvey, D.J.; Au, L.E.; Shadyab, A.H.; Santiago-Torres, M.; Liu, S.; Shivappa, N.; Hébert, J.R.; Robbins, J.A.; Garcia, L. Energy-Adjusted Dietary Inflammatory Index and Diabetes Risk in Postmenopausal Hispanic Women. *J. Acad. Nutr. Diet.* **2024**, *124*, 1431–1439, doi:10.1016/j.jand.2023.08.002.
78. Quade, D. Rank Analysis of Covariance. *J. Am. Stat. Assoc.* **1967**, *62*, 1187–1200, doi:10.1080/01621459.1967.10500925.
79. Siegel, S.; Castellan, N.J. *Nonparametric Statistics for the Behavioral Sciences*; McGraw-Hill international editions statistics series; McGraw-Hill: New York, NY, 1988; ISBN 9780070573574.
80. Sihombing, P.R. Alternative Evaluation of Nonparametric Analysis of Covariance (Ancova) Methods: Simulation Study for Violation of Parametric Assumptions. *Theta J. Stat. Vol 1, No 2 Available Online Sept. 2025* **2025**.
81. Benjamini, Y.; Hochberg, Y. Controlling the False Discovery Rate: A Practical and Powerful Approach to Multiple Testing. *J. R. Stat. Soc. Ser. B* **1995**, *57*, 289–300, doi:10.1111/j.2517-6161.1995.tb02031.x.
82. Kendall, M.G.; Gibbons, J.D. *Rank Correlation Methods*; A Charles Griffin title; Edward Arnold: London, 1990; ISBN 9780852643051.
83. Patin, E.; Hasan, M.; Bergstedt, J.; Rouilly, V.; Libri, V.; Urrutia, A.; Alanio, C.; Scepanovic, P.; Hammer, C.; Jönsson, F.; et al. Natural Variation in the Parameters of Innate Immune Cells Is Preferentially Driven by Genetic Factors. *Nat. Immunol.* **2018**, *19*, 302–314, doi:10.1038/s41590-018-0049-7.
84. Oertelt-Prigione, S. The Influence of Sex and Gender on the Immune Response. *Autoimmun. Rev.* **2012**, *11*, A479–A485, doi:10.1016/j.autrev.2011.11.022.
85. Gardner, E.M.; Murasko, D.M. Age-Related Changes in Type 1 and Type 2 Cytokine Production in Humans. *Biogerontology* **2002**, *3*, 271–290, doi:10.1023/a:1020151401826.
86. D'Esposito, V.; Di Tolla, M.F.; Lecce, M.; Cavalli, F.; Libutti, M.; Misso, S.; Cabaro, S.; Ambrosio, M.R.; Parascandolo, A.; Covelli, B.; et al. Lifestyle and Dietary Habits Affect Plasma Levels of Specific Cytokines in Healthy Subjects. *Front. Nutr.* **2022**, *9*, 913176, doi:10.3389/fnut.2022.913176.
87. European Food Safety Authority (EFSA) Dietary Reference Values for Nutrients Summary Report. *EFSA Support. Publ.* **2017**, *14*, e15121E, doi:doi.org/10.2903/sp.efsa.2017.e15121.
88. Kniskern, M.A.; Johnston, C.S. Protein Dietary Reference Intakes May Be Inadequate for Vegetarians If Low Amounts of Animal Protein Are Consumed. *Nutrition* **2011**, *27*, 727–730, doi:10.1016/j.nut.2010.08.024.
89. Paalani, M.; Lee, J.W.; Haddad, E.; Tonstad, S. Determinants of Inflammatory Markers in a Bi-Ethnic Population. *Ethn. Dis.*

2011, *21*, 142–149.

90. Fallon, N.; Dillon, S.A. Low Intakes of Iodine and Selenium Amongst Vegan and Vegetarian Women Highlight a Potential Nutritional Vulnerability. *Front. Nutr.* **2020**, *7*, 72, doi:10.3389/fnut.2020.00072.
91. Kramer, L.B.; Osis, D.; Coffey, J.; Spencer, H. Mineral and Trace Element Content of Vegetarian Diets. *J. Am. Coll. Nutr.* **1984**, *3*, 3–11, doi:10.1080/07315724.1984.10720032.
92. Klein, L.; Dawczynski, C.; Schwarz, M.; Maares, M.; Kipp, K.; Haase, H.; Kipp, A.P. Selenium, Zinc, and Copper Status of Vegetarians and Vegans in Comparison to Omnivores in the Nutritional Evaluation (NuEva) Study. *Nutrients* **2023**, *15*, doi:10.3390/nu15163538.
93. Groufh-Jacobsen, S.; Larsson, C.; Margerison, C.; Mulkerrins, I.; Aune, D.; Medin, A.C. Micronutrient Intake and Status in Young Vegans, Lacto-Ovo-Vegetarians, Pescatarians, Flexitarians, and Omnivores. *Eur. J. Nutr.* **2024**, *63*, 2725–2741, doi:10.1007/s00394-024-03453-4.
94. Dinu, M.; Pagliai, G.; Cesari, F.; Giusti, B.; Gori, A.M.; Marcucci, R.; Casini, A.; Sofi, F. Effects of a 3-Month Dietary Intervention with a Lacto-Ovo-Vegetarian Diet on Vitamin B(12) Levels in a Group of Omnivores: Results from the CARDIVEG (Cardiovascular Prevention with Vegetarian Diet) Study. *Br. J. Nutr.* **2019**, *121*, 756–762, doi:10.1017/S0007114518003835.
95. Kristensen, N.B.; Madsen, M.L.; Hansen, T.H.; Allin, K.H.; Hoppe, C.; Fagt, S.; Lausten, M.S.; Gøbel, R.J.; Vestergaard, H.; Hansen, T.; et al. Intake of Macro- and Micronutrients in Danish Vegans. *Nutr. J.* **2015**, *14*, 115, doi:10.1186/s12937-015-0103-3.
96. Waldmann, A.; Koschizke, J.W.; Leitzmann, C.; Hahn, A. Dietary Intakes and Lifestyle Factors of a Vegan Population in Germany: Results from the German Vegan Study. *Eur. J. Clin. Nutr.* **2003**, *57*, 947–955, doi:10.1038/sj.ejcn.1601629.
97. Clarys, P.; Deliens, T.; Huybrechts, I.; Deriemaeker, P.; Vanaelst, B.; De Keyzer, W.; Hebbelinck, M.; Mullie, P. Comparison of Nutritional Quality of the Vegan, Vegetarian, Semi-Vegetarian, Pesco-Vegetarian and Omnivorous Diet. *Nutrients* **2014**, *6*, 1318–1332, doi:10.3390/nu6031318.
98. American Dietetic Association; Dietitians of Canada Position of the American Dietetic Association and Dietitians of Canada: Vegetarian Diets. *J. Am. Diet. Assoc.* **2003**, *103*, 748–765, doi:10.1053/jada.2003.50142.
99. Schüpbach, R.; Wegmüller, R.; Berguerand, C.; Bui, M.; Herter-Aeberli, I. Micronutrient Status and Intake in Omnivores, Vegetarians and Vegans in Switzerland. *Eur. J. Nutr.* **2017**, *56*, 283–293, doi:10.1007/s00394-015-1079-7.
100. Farmer, B.; Larson, B.T.; Fulgoni, V.L. 3rd; Rainville, A.J.; Liepa, G.U. A Vegetarian Dietary Pattern as a Nutrient-Dense Approach to Weight Management: An Analysis of the National Health and Nutrition Examination Survey 1999-2004. *J. Am. Diet. Assoc.* **2011**, *111*, 819–827, doi:10.1016/j.jada.2011.03.012.
101. Rizzo, N.S.; Jaceldo-Siegl, K.; Sabate, J.; Fraser, G.E. Nutrient Profiles of Vegetarian and Nonvegetarian Dietary Patterns. *J. Acad. Nutr. Diet.* **2013**, *113*, 1610–1619, doi:10.1016/j.jand.2013.06.349.
102. Santos, H.O.; Price, J.C.; Bueno, A.A. Beyond Fish Oil Supplementation: The Effects of Alternative Plant Sources of Omega-3 Polyunsaturated Fatty Acids upon Lipid Indexes and Cardiometabolic Biomarkers-An Overview. *Nutrients* **2020**, *12*, doi:10.3390/nu12103159.
103. Menzel, J.; Longree, A.; Abraham, K.; Schulze, M.B.; Weikert, C. Dietary and Plasma Phospholipid Profiles in Vegans and Omnivores-Results from the RBVD Study. *Nutrients* **2022**, *14*, doi:10.3390/nu14142900.
104. Burns-Whitmore, B.; Froyen, E.; Heskey, C.; Parker, T.; San Pablo, G. Alpha-Linolenic and Linoleic Fatty Acids in the Vegan Diet: Do They Require Dietary Reference Intake/Adequate Intake Special Consideration? *Nutrients* **2019**, *11*, doi:10.3390/nu11102365.
105. Milešević, J.; Zeković, M.; Šarac, I.; Knez, M.; Krga, I.; Takić, M.; Debeljak-Martačić, J.; Stevanović, V.; Vidović, N.; Kadvan, A.; et al. EFSA EU Menu Food Consumption Survey in Serbia 2017-2022: Energy and Macronutrient Dietary Intakes and Anthropometric Status of Serbian Adults 18–64 Years Old. *Foods* **2025**, *(submiited*.
106. Šarac, I.; Debeljak-Martačić, J.; Takić, M.; Stevanović, V.; Milešević, J.; Zeković, M.; Popović, T.; Jovanović, J.; Vidović, N.K.

Associations of Fatty Acids Composition and Estimated Desaturase Activities in Erythrocyte Phospholipids with Biochemical and Clinical Indicators of Cardiometabolic Risk in Non-Diabetic Serbian Women: The Role of Level of Adiposity. *Front. Nutr.* **2023**, *10*, 1065578, doi:10.3389/fnut.2023.1065578.
107. Moon, J.; Koh, G. Clinical Evidence and Mechanisms of High-Protein Diet-Induced Weight Loss. *J. Obes. Metab. Syndr.* **2020**, *29*, 166–173, doi:10.7570/jomes20028.
108. Simonson, M.; Boirie, Y.; Guillet, C. Protein, Amino Acids and Obesity Treatment. *Rev. Endocr. Metab. Disord.* **2020**, *21*, 341–353, doi:10.1007/s11154-020-09574-5.
109. Wycherley, T.P.; Moran, L.J.; Clifton, P.M.; Noakes, M.; Brinkworth, G.D. Effects of Energy-Restricted High-Protein, Low-Fat Compared with Standard-Protein, Low-Fat Diets: A Meta-Analysis of Randomized Controlled Trials. *Am. J. Clin. Nutr.* **2012**, *96*, 1281–1298, doi:10.3945/ajcn.112.044321.
110. Westerterp, K.R. Diet Induced Thermogenesis. *Nutr. Metab. (Lond).* **2004**, *1*, 5, doi:10.1186/1743-7075-1-5.
111. Calcagno, M.; Kahleova, H.; Alwarith, J.; Burgess, N.N.; Flores, R.A.; Busta, M.L.; Barnard, N.D. The Thermic Effect of Food: A Review. *J. Am. Coll. Nutr.* **2019**, *38*, 547–551, doi:10.1080/07315724.2018.1552544.
112. Fernández-Verdejo, R.; Sanchez-Delgado, G.; Ravussin, E. Energy Expenditure in Humans: Principles, Methods, and Changes Throughout the Life Course. *Annu. Rev. Nutr.* **2024**, *44*, 51–76, doi:10.1146/annurev-nutr-062122-031443.
113. Guarneiri, L.L.; Adams, C.G.; Garcia-Jackson, B.; Koecher, K.; Wilcox, M.L.; Maki, K.C. Effects of Varying Protein Amounts and Types on Diet-Induced Thermogenesis: A Systematic Review and Meta-Analysis. *Adv. Nutr.* **2024**, *15*, 100332, doi:10.1016/j.advnut.2024.100332.
114. Pesta, D.H.; Samuel, V.T. A High-Protein Diet for Reducing Body Fat: Mechanisms and Possible Caveats. *Nutr. Metab. (Lond).* **2014**, *11*, 53, doi:10.1186/1743-7075-11-53.
115. Bissoli, L.; Armellini, F.; Zamboni, M.; Mandragona, R.; Ballarin, A.; Bosello, O. Resting Metabolic Rate and Thermogenic Effect of Food in Vegetarian Diets Compared with Mediterranean Diets. *Ann. Nutr. Metab.* **1999**, *43*, 140–144, doi:10.1159/000012779.
116. Oberlin, P.; Melby, C.L.; Poehlman, E.T. Resting Energy Expenditure in Young Vegetarian and Nonvegetarian Women. *Nutr. Res.* **1990**, *10*, 39–49, doi:10.1016/S0271-5317(05)80764-X.
117. Turner, M.D.; Nedjai, B.; Hurst, T.; Pennington, D.J. Cytokines and Chemokines: At the Crossroads of Cell Signalling and Inflammatory Disease. *Biochim. Biophys. Acta - Mol. Cell Res.* **2014**, *1843*, 2563–2582, doi:10.1016/j.bbamcr.2014.05.014.
118. Dinarello, C.A. Overview of the IL-1 Family in Innate Inflammation and Acquired Immunity. *Immunol. Rev.* **2018**, *281*, 8–27, doi:10.1111/imr.12621.
119. Scheller, J.; Chalaris, A.; Schmidt-Arras, D.; Rose-John, S. The Pro- and Anti-Inflammatory Properties of the Cytokine Interleukin-6. *Biochim. Biophys. Acta - Mol. Cell Res.* **2011**, *1813*, 878–888, doi:10.1016/j.bbamcr.2011.01.034.
120. Molofsky, A.B.; Savage, A.K.; Locksley, R.M. Interleukin-33 in Tissue Homeostasis, Injury, and Inflammation. *Immunity* **2015**, *42*, 1005–1019, doi:10.1016/j.immuni.2015.06.006.
121. Zlotnik, A.; Yoshie, O. Chemokines: A New Classification System and Their Role in Immunity. *Immunity* **2000**, *12*, 121–127, doi:10.1016/s1074-7613(00)80165-x.
122. Moudgil, K.D.; Choubey, D. Cytokines in Autoimmunity: Role in Induction, Regulation, and Treatment. *J. Interf. Cytokine Res.* **2011**, *31*, 695–703, doi:10.1089/jir.2011.0065.
123. Opal, S.M.; DePalo, V.A. Anti-Inflammatory Cytokines. *Chest* **2000**, *117*, 1162–1172, doi:10.1378/chest.117.4.1162.
124. Wautier, J.-L.; Wautier, M.-P. Pro- and Anti-Inflammatory Prostaglandins and Cytokines in Humans: A Mini Review. *Int. J. Mol. Sci.* **2023**, *24*, doi:10.3390/ijms24119647.
125. Wang, F.; Zhao, C.; Jing, Z.; Wang, Q.; Li, M.; Lu, B.; Huo, A.; Liang, W.; Hu, W.; Fu, X. The Dual Roles of Chemokines in Peripheral Nerve Injury and Repair. *Inflamm. Regen.* **2025**, *45*, 11, doi:10.1186/s41232-025-00375-4.
126. Lee, J.J.; Yang, L.; Kotzin, J.J.; Ahimovic, D.; Bale, M.J.; Nigrovic, P.A.; Josefowicz, S.Z.; Mathis, D.; Benoist, C. Early Transcriptional Effects of Inflammatory Cytokines Reveal Highly Redundant Cytokine Networks. *J. Exp. Med.* **2025**, *222*,

doi:10.1084/jem.20241207.
127. Bradley, J.R. TNF-Mediated Inflammatory Disease. *J. Pathol.* **2008**, *214*, 149–160, doi:10.1002/path.2287.
128. Deshmane, S.L.; Kremlev, S.; Amini, S.; Sawaya, B.E. Monocyte Chemoattractant Protein-1 (MCP-1): An Overview. *J. Interf. cytokine Res. Off. J. Int. Soc. Interf. Cytokine Res.* **2009**, *29*, 313–326, doi:10.1089/jir.2008.0027.
129. Gaffen, S.L.; Jain, R.; Garg, A. V; Cua, D.J. The IL-23-IL-17 Immune Axis: From Mechanisms to Therapeutic Testing. *Nat. Rev. Immunol.* **2014**, *14*, 585–600, doi:10.1038/nri3707.
130. Hunter, C.A.; Jones, S.A. IL-6 as a Keystone Cytokine in Health and Disease. *Nat. Immunol.* **2015**, *16*, 448–457, doi:10.1038/ni.3153.
131. Tominaga, K.; Yoshimoto, T.; Torigoe, K.; Kurimoto, M.; Matsui, K.; Hada, T.; Okamura, H.; Nakanishi, K. IL-12 Synergizes with IL-18 or IL-1beta for IFN-Gamma Production from Human T Cells. *Int. Immunol.* **2000**, *12*, 151–160, doi:10.1093/intimm/12.2.151.
132. Dinarello, C.; Novick, D.; Kim, S.; Kaplanski, G. Interleukin-18 and IL-18 Binding Protein. *Front. Immunol.* **2013**, *Volume 4-*.
133. Schroder, K.; Hertzog, P.J.; Ravasi, T.; Hume, D.A. Interferon-γ: An Overview of Signals, Mechanisms and Functions. *J. Leukoc. Biol.* **2004**, *75*, 163–189, doi:10.1189/jlb.0603252.
134. Rincón, M.; Enslen, H.; Raingeaud, J.; Recht, M.; Zapton, T.; Su, M.S.; Penix, L.A.; Davis, R.J.; Flavell, R.A. Interferon-Gamma Expression by Th1 Effector T Cells Mediated by the P38 MAP Kinase Signaling Pathway. *EMBO J.* **1998**, *17*, 2817–2829, doi:10.1093/emboj/17.10.2817.
135. Ter Horst, R.; Jaeger, M.; Smeekens, S.P.; Oosting, M.; Swertz, M.A.; Li, Y.; Kumar, V.; Diavatopoulos, D.A.; Jansen, A.F.M.; Lemmers, H.; et al. Host and Environmental Factors Influencing Individual Human Cytokine Responses. *Cell* **2016**, *167*, 1111-1124.e13, doi:10.1016/j.cell.2016.10.018.
136. Kleiner, G.; Marcuzzi, A.; Zanin, V.; Monasta, L.; Zauli, G. Cytokine Levels in the Serum of Healthy Subjects. *Mediators Inflamm.* **2013**, *2013*, 434010, doi:10.1155/2013/434010.
137. Biancotto, A.; Wank, A.; Perl, S.; Cook, W.; Olnes, M.J.; Dagur, P.K.; Fuchs, J.C.; Langweiler, M.; Wang, E.; McCoy, J.P. Baseline Levels and Temporal Stability of 27 Multiplexed Serum Cytokine Concentrations in Healthy Subjects. *PLoS One* **2013**, *8*, e76091, doi:10.1371/journal.pone.0076091.
138. Wyse, C.; O'Malley, G.; Coogan, A.N.; McConkey, S.; Smith, D.J. Seasonal and Daytime Variation in Multiple Immune Parameters in Humans: Evidence from 329,261 Participants of the UK Biobank Cohort. *iScience* **2021**, *24*, doi:10.1016/j.isci.2021.102255.
139. Thompson, S.R.; Novick, D.; Stock, C.J.; Sanders, J.; Brull, D.; Cooper, J.; Woo, P.; Miller, G.; Rubinstein, M.; Humphries, S.E. Free Interleukin (IL)-18 Levels, and the Impact of IL18 and IL18BP Genetic Variation, in CHD Patients and Healthy Men. *Arterioscler. Thromb. Vasc. Biol.* **2007**, *27*, 2743–2749, doi:10.1161/ATVBAHA.107.149245.
140. Hosnijeh, F.S.; Krop, E.J.M.; Portengen, L.; Rabkin, C.S.; Linseisen, J.; Vineis, P.; Vermeulen, R. Stability and Reproducibility of Simultaneously Detected Plasma and Serum Cytokine Levels in Asymptomatic Subjects. *Biomarkers* **2010**, *15*, 140–148, doi:10.3109/13547500903340570.
141. Rose, G.L.; Farley, M.J.; Flemming, N.B.; Skinner, T.L.; Schaumberg, M.A. Between-Day Reliability of Cytokines and Adipokines for Application in Research and Practice. *Front. Physiol.* **2022**, *Volume 13*.
142. Mallard, A.R.; Hollekim-Strand, S.M.; Ingul, C.B.; Coombes, J.S. High Day-to-Day and Diurnal Variability of Oxidative Stress and Inflammation Biomarkers in People with Type 2 Diabetes Mellitus and Healthy Individuals. *Redox Rep.* **2020**, *25*, 64–69, doi:10.1080/13510002.2020.1795587.
143. Fogarty, C.L.; Nieminen, J.K.; Peräneva, L.; Lassenius, M.I.; Ahola, A.J.; Taskinen, M.-R.; Jauhiainen, M.; Kirveskari, J.; Pussinen, P.; Hörkkö, S.; et al. High-Fat Meals Induce Systemic Cytokine Release without Evidence of Endotoxemia-Mediated Cytokine Production from Circulating Monocytes or Myeloid Dendritic Cells. *Acta Diabetol.* **2015**, *52*, 315–322, doi:10.1007/s00592-014-0641-8.
144. Koelman, L.; Pivovarova-Ramich, O.; Pfeiffer, A.F.H.; Grune, T.; Aleksandrova, K. Cytokines for Evaluation of Chronic

Inflammatory Status in Ageing Research: Reliability and Phenotypic Characterisation. *Immun. Ageing* **2019**, *16*, 11, doi:10.1186/s12979-019-0151-1.
145. Traba, J.; Kwarteng-Siaw, M.; Okoli, T.C.; Li, J.; Huffstutler, R.D.; Bray, A.; Waclawiw, M.A.; Han, K.; Pelletier, M.; Sauve, A.A.; et al. Fasting and Refeeding Differentially Regulate NLRP3 Inflammasome Activation in Human Subjects. *J. Clin. Invest.* **2015**, *125*, 4592–4600, doi:10.1172/JCI83260.
146. Todd, J.; Simpson, P.; Estis, J.; Torres, V.; Wub, A.H.B. Reference Range and Short- and Long-Term Biological Variation of Interleukin (IL)-6, IL-17A and Tissue Necrosis Factor-Alpha Using High Sensitivity Assays. *Cytokine* **2013**, *64*, 660–665, doi:10.1016/j.cyto.2013.09.018.
147. Lopez-Castejon, G.; Brough, D. Understanding the Mechanism of IL-1β Secretion. *Cytokine Growth Factor Rev.* **2011**, *22*, 189–195, doi:10.1016/j.cytogfr.2011.10.001.
148. Ford, S.G.; Caswell, P.; Brough, D.; Seoane, P.I. The Secretion of Interleukin-1β. *Cytokine Growth Factor Rev.* **2025**, *84*, 101–113, doi:10.1016/j.cytogfr.2025.05.005.
149. Sundnes, O.; Ottestad, W.; Schjalm, C.; Lundbäck, P.; la Cour Poulsen, L.; Mollnes, T.E.; Haraldsen, G.; Eken, T. Rapid Systemic Surge of IL-33 after Severe Human Trauma: A Prospective Observational Study. *Mol. Med.* **2021**, *27*, 29, doi:10.1186/s10020-021-00288-1.
150. Heney, D.; Whicher, J.T. Factors Affecting the Measurement of Cytokines in Biological Fluids: Implications for Their Clinical Measurement. *Ann. Clin. Biochem.* **1995**, *32 (Pt 4)*, 358–368, doi:10.1177/000456329503200402.
151. Zhang, Y.; Saccani, S.; Shin, H.; Nikolajczyk, B.S. Dynamic Protein Associations Define Two Phases of IL-1beta Transcriptional Activation. *J. Immunol.* **2008**, *181*, 503–512, doi:10.4049/jimmunol.181.1.503.
152. Liu, C.; Chu, D.; Kalantar-Zadeh, K.; George, J.; Young, H.A.; Liu, G. Cytokines: From Clinical Significance to Quantification. *Adv. Sci. (Weinheim, Baden-Wurttemberg, Ger.* **2021**, *8*, e2004433, doi:10.1002/advs.202004433.
153. Neudorf, H.; Little, J.P. Impact of Fasting & Ketogenic Interventions on the NLRP3 Inflammasome: A Narrative Review. *Biomed. J.* **2024**, *47*, 100677, doi:10.1016/j.bj.2023.100677.
154. Kiers, D.; Koch, R.M.; Hamers, L.; Gerretsen, J.; Thijs, E.J.M.; van Ede, L.; Riksen, N.P.; Kox, M.; Pickkers, P. Characterization of a Model of Systemic Inflammation in Humans in Vivo Elicited by Continuous Infusion of Endotoxin. *Sci. Rep.* **2017**, *7*, 40149, doi:10.1038/srep40149.
155. Granowitz, E. V; Santos, A.A.; Poutsiaka, D.D.; Cannon, J.G.; Wilmore, D.W.; Wolff, S.M.; Dinarello, C.A. Production of Interleukin-1-Receptor Antagonist during Experimental Endotoxaemia. *Lancet (London, England)* **1991**, *338*, 1423–1424, doi:10.1016/0140-6736(91)92725-h.
156. Dorresteijn, M.J.; Pickkers, P.; Netea, M.G. IFN-γ Is Not Induced through Increased Plasma Concentrations of Interleukin-12/Interleukin-18 during Human Endotoxemia. *Eur. Cytokine Netw.* **2005**, *16*, 191–193.
157. Dillingh, M.R.; van Poelgeest, E.P.; Malone, K.E.; Kemper, E.M.; Stroes, E.S.G.; Moerland, M.; Burggraaf, J. Characterization of Inflammation and Immune Cell Modulation Induced by Low-Dose LPS Administration to Healthy Volunteers. *J. Inflamm.* **2014**, *11*, 28, doi:10.1186/s12950-014-0028-1.
158. Copeland, S.; Warren, H.S.; Lowry, S.F.; Calvano, S.E.; Remick, D. Acute Inflammatory Response to Endotoxin in Mice and Humans. *Clin. Diagn. Lab. Immunol.* **2005**, *12*, 60–67, doi:10.1128/CDLI.12.1.60-67.2005.
159. van der Meer, W.; Pickkers, P.; Scott, C.S.; van der Hoeven, J.G.; Gunnewiek, J.K. Hematological Indices, Inflammatory Markers and Neutrophil CD64 Expression: Comparative Trends during Experimental Human Endotoxemia. *J. Endotoxin Res.* **2007**, *13*, 94–100, doi:10.1177/0968051907079101.
160. Keramati, F.; Leijte, G.P.; Bruse, N.; Grondman, I.; Habibi, E.; Ruiz-Moreno, C.; Megchelenbrink, W.; Peters van Ton, A.M.; Heesakkers, H.; Bremmers, M.E.; et al. Systemic Inflammation Impairs Myelopoiesis and Interferon Type I Responses in Humans. *Nat. Immunol.* **2025**, *26*, 737–747, doi:10.1038/s41590-025-02136-4.
161. Grobmyer, S.R.; Lin, E.; Lowry, S.F.; Rivadeneira, D.E.; Potter, S.; Barie, P.S.; Nathan, C.F. Elevation of IL-18 in Human Sepsis. *J. Clin. Immunol.* **2000**, *20*, 212–215, doi:10.1023/a:1006641630904.

162. Papatheodorou, I.; Blažková, G.; Bosáková, V.; Tomášiková, Z.; Spearing, E.; Klieber, R.; Ostašov, P.; Štíchová, J.; Dvončová, M.; Mýtniková, A.; et al. Redefining the Role of IL-18 in Post-Surgical Recovery and Sepsis: A Key Mediator of Inflammation Resolution. *J. Transl. Med.* **2025**, *23*, 728, doi:10.1186/s12967-025-06652-7.
163. Harel, M.; Fauteux-Daniel, S.; Girard-Guyonvarc'h, C.; Gabay, C. Balance between Interleukin-18 and Interleukin-18 Binding Protein in Auto-Inflammatory Diseases. *Cytokine* **2022**, *150*, 155781, doi:10.1016/j.cyto.2021.155781.
164. Dreaden, K.H.; Pearson, S.-P.; Gurel, P.S.; Newman, R.G.; Zhao, Y.; Wang, C.; Heiber, J.F.; Donatelli, S.S.; Chamoun, J.; Whitmore, M.M. Interleukin-18 (IL-18) Engineered for Half-Life Extension and Resistance to IL-18 Binding Protein (IL-18BP) to Enhance Anti-Cancer Therapeutic Potential. *Cytokine* **2025**, *193*, 156979, doi:10.1016/j.cyto.2025.156979.
165. Robertson, M.J.; Kirkwood, J.M.; Logan, T.F.; Koch, K.M.; Kathman, S.; Kirby, L.C.; Bell, W.N.; Thurmond, L.M.; Weisenbach, J.; Dar, M.M. A Dose-Escalation Study of Recombinant Human Interleukin-18 Using Two Different Schedules of Administration in Patients with Cancer. *Clin. cancer Res. an Off. J. Am. Assoc. Cancer Res.* **2008**, *14*, 3462–3469, doi:10.1158/1078-0432.CCR-07-4740.
166. Pourreza, S.; Khademi, Z.; Mirzababaei, A.; Yekaninejad, M.S.; Sadeghniiat-Haghighi, K.; Naghshi, S.; Mirzaei, K. Association of Plant-Based Diet Index with Inflammatory Markers and Sleep Quality in Overweight and Obese Female Adults: A Cross-Sectional Study. *Int. J. Clin. Pract.* **2021**, *75*, e14429, doi:10.1111/ijcp.14429.
167. Koelman, L.; Egea Rodrigues, C.; Aleksandrova, K. Effects of Dietary Patterns on Biomarkers of Inflammation and Immune Responses: A Systematic Review and Meta-Analysis of Randomized Controlled Trials. *Adv. Nutr.* **2022**, *13*, 101–115, doi:10.1093/advances/nmab086.
168. Schell, J.; Betts, N.M.; Foster, M.; Scofield, R.H.; Basu, A. Cranberries Improve Postprandial Glucose Excursions in Type 2 Diabetes. *Food Funct.* **2017**, *8*, 3083–3090, doi:10.1039/c7fo00900c.
169. Nilsson, A.; Johansson-Boll, E.; Sandberg, J.; Björck, I. Gut Microbiota Mediated Benefits of Barley Kernel Products on Metabolism, Gut Hormones, and Inflammatory Markers as Affected by Co-Ingestion of Commercially Available Probiotics: A Randomized Controlled Study in Healthy Subjects. *Clin. Nutr. ESPEN* **2016**, *15*, 49–56, doi:10.1016/j.clnesp.2016.06.006.
170. Devaraj, S.; Wang-Polagruto, J.; Polagruto, J.; Keen, C.L.; Jialal, I. High-Fat, Energy-Dense, Fast-Food-Style Breakfast Results in an Increase in Oxidative Stress in Metabolic Syndrome. *Metabolism.* **2008**, *57*, 867–870, doi:10.1016/j.metabol.2008.02.016.
171. Schönknecht, Y.B.; Crommen, S.; Stoffel-Wagner, B.; Coenen, M.; Fimmers, R.; Stehle, P.; Egert, S. Influence of a Proinflammatory State on Postprandial Outcomes in Elderly Subjects with a Risk Phenotype for Cardiometabolic Diseases. *Eur. J. Nutr.* **2022**, *61*, 3077–3083, doi:10.1007/s00394-022-02870-7.
172. Perez-Herrera, A.; Delgado-Lista, J.; Torres-Sanchez, L.A.; Rangel-Zuñiga, O.A.; Camargo, A.; Moreno-Navarrete, J.M.; Garcia-Olid, B.; Quintana-Navarro, G.M.; Alcala-Diaz, J.F.; Muñoz-Lopez, C.; et al. The Postprandial Inflammatory Response after Ingestion of Heated Oils in Obese Persons Is Reduced by the Presence of Phenol Compounds. *Mol. Nutr. Food Res.* **2012**, *56*, 510–514, doi:10.1002/mnfr.201100533.
173. Demmer, E.; Van Loan, M.D.; Rivera, N.; Rogers, T.S.; Gertz, E.R.; German, J.B.; Zivkovic, A.M.; Smilowitz, J.T. Consumption of a High-Fat Meal Containing Cheese Compared with a Vegan Alternative Lowers Postprandial C-Reactive Protein in Overweight and Obese Individuals with Metabolic Abnormalities: A Randomised Controlled Cross-over Study. *J. Nutr. Sci.* **2016**, *5*, e9, doi:10.1017/jns.2015.40.
174. Esposito, K.; Nappo, F.; Giugliano, F.; Di Palo, C.; Ciotola, M.; Barbieri, M.; Paolisso, G.; Giugliano, D. Meal Modulation of Circulating Interleukin 18 and Adiponectin Concentrations in Healthy Subjects and in Patients with Type 2 Diabetes Mellitus. *Am. J. Clin. Nutr.* **2003**, *78*, 1135–1140, doi:10.1093/ajcn/78.6.1135.
175. Caixàs, A.; Giménez-Palop, O.; Broch, M.; Vilardell, C.; Megía, A.; Simón, I.; Giménez-Pérez, G.; Mauricio, D.; Vendrell, J.; Richart, C.; et al. Adult Subjects with Prader-Willi Syndrome Show More Low-Grade Systemic Inflammation than Matched Obese Subjects. *J. Endocrinol. Invest.* **2008**, *31*, 169–175, doi:10.1007/BF03345585.
176. Goulet, N.; Tetzlaff, E.J.; Morin, R.; Mauger, J.-F.; Amaratunga, R.; Kenny, G.P.; Imbeault, P. No Impact of a High-Fat Meal Coupled with Intermittent Hypoxemia on Acute Kidney Injury Biomarkers in Adults with and without Obstructive Sleep

Apnea. *Physiol. Rep.* **2023**, *11*, e15804, doi:10.14814/phy2.15804.

177. Nilsson, A.; Johansson, E.; Ekström, L.; Björck, I. Effects of a Brown Beans Evening Meal on Metabolic Risk Markers and Appetite Regulating Hormones at a Subsequent Standardized Breakfast: A Randomized Cross-over Study. *PLoS One* **2013**, *8*, e59985, doi:10.1371/journal.pone.0059985.
178. Olszyna, D.P.; De Jonge, E.; Dekkers, P.E.; van Deventer, S.J.; van der Poll, T. Induction of Cell-Associated Chemokines after Endotoxin Administration to Healthy Humans. *Infect. Immun.* **2001**, *69*, 2736–2738, doi:10.1128/IAI.69.4.2736-2738.2001.
179. Heesen, M.; Renckens, R.; de Vos, A.F.; Kunz, D.; van der Poll, T. Human Endotoxemia Induces Down-Regulation of Monocyte CC Chemokine Receptor 2. *Clin. Vaccine Immunol.* **2006**, *13*, 156–159, doi:10.1128/CVI.13.1.156-159.2006.
180. Herieka, M.; Erridge, C. High-Fat Meal Induced Postprandial Inflammation. *Mol. Nutr. Food Res.* **2014**, *58*, 136–146, doi:10.1002/mnfr.201300104.
181. van Wijk, J.P.H.; Cabezas, M.C.; Coll, B.; Joven, J.; Rabelink, T.J.; de Koning, E.J.P. Effects of Rosiglitazone on Postprandial Leukocytes and Cytokines in Type 2 Diabetes. *Atherosclerosis* **2006**, *186*, 152–159, doi:10.1016/j.atherosclerosis.2005.07.001.
182. Liew, F.Y.; Girard, J.-P.; Turnquist, H.R. Interleukin-33 in Health and Disease. *Nat. Rev. Immunol.* **2016**, *16*, 676+.
183. Miller, A.M. Role of IL-33 in Inflammation and Disease. *J. Inflamm. (Lond).* **2011**, *8*, 22, doi:10.1186/1476-9255-8-22.
184. Mildner, M.; Storka, A.; Lichtenauer, M.; Mlitz, V.; Ghannadan, M.; Hoetzenecker, K.; Nickl, S.; Dome, B.; Tschachler, E.; Ankersmit, H.J. Primary Sources and Immunological Prerequisites for SST2 Secretion in Humans. *Cardiovasc. Res.* **2010**, *87*, 769–777, doi:10.1093/cvr/cvq104.
185. Cohen, E.S.; Scott, I.C.; Majithiya, J.B.; Rapley, L.; Kemp, B.P.; England, E.; Rees, D.G.; Overed-Sayer, C.L.; Woods, J.; Bond, N.J.; et al. Oxidation of the Alarmin IL-33 Regulates ST2-Dependent Inflammation. *Nat. Commun.* **2015**, *6*, 8327, doi:10.1038/ncomms9327.
186. Kakkar, R.; Lee, R.T. The IL-33/ST2 Pathway: Therapeutic Target and Novel Biomarker. *Nat. Rev. Drug Discov.* **2008**, *7*, 827–840, doi:10.1038/nrd2660.
187. Saresella, M.; Marventano, I.; Piancone, F.; La Rosa, F.; Galimberti, D.; Fenoglio, C.; Scarpini, E.; Clerici, M. IL-33 and Its Decoy SST2 in Patients with Alzheimer's Disease and Mild Cognitive Impairment. *J. Neuroinflammation* **2020**, *17*, 174, doi:10.1186/s12974-020-01806-4.
188. Cayrol, C. IL-33, an Alarmin of the IL-1 Family Involved in Allergic and Non Allergic Inflammation: Focus on the Mechanisms of Regulation of Its Activity. *Cells* **2021**, *11*, doi:10.3390/cells11010107.
189. Nile, C.J.; Barksby, E.; Jitprasertwong, P.; Preshaw, P.M.; Taylor, J.J. Expression and Regulation of Interleukin-33 in Human Monocytes. *Immunology* **2010**, *130*, 172–180, doi:10.1111/j.1365-2567.2009.03221.x.
190. Gorzelak-Pabiś, P.; Wozniak, E.; Wojdan, K.; Chalubinski, M.; Broncel, M. Single Triglyceride-Rich Meal Destabilizes Barrier Functions and Initiates Inflammatory Processes of Endothelial Cells. *J. Interferon Cytokine Res.* **2020**, *40*, 43–53, doi:10.1089/jir.2018.0173.
191. Quan, J.-H.; Zhou, W.; Cha, G.-H.; Choi, I.-W.; Shin, D.-W.; Lee, Y.-H. Kinetics of IL-23 and IL-12 Secretion in Response to Toxoplasma Gondii Antigens from THP-1 Monocytic Cells. *Korean J. Parasitol.* **2013**, *51*, 85–92, doi:10.3347/kjp.2013.51.1.85.
192. Wynick, C.; Petes, C.; Tigert, A.; Gee, K. Lipopolysaccharide-Mediated Induction of Concurrent IL-1β and IL-23 Expression in THP-1 Cells Exhibits Differential Requirements for Caspase-1 and Cathepsin B Activity. *J. Interferon Cytokine Res.* **2016**, *36*, 477–487, doi:10.1089/jir.2015.0134.
193. Tüccar, T.B.; Akbulut, G. Mediterranean Meal Favorably Effects Postprandial Oxidative Stress Response Compared with a Western Meal in Healthy Women. *Int. J. Vitam. Nutr. Res. Int. Zeitschrift fur Vitamin- und Ernahrungsforschung. J. Int. Vitaminol. Nutr.* **2023**, *93*, 319–328, doi:10.1024/0300-9831/a000734.
194. Pourhassan, M.; Babel, N.; Sieske, L.; Westhoff, T.H.; Wirth, R. Longitudinal Changes of Cytokines and Appetite in Older Hospitalized Patients. *Nutrients* **2021**, *13*, doi:10.3390/nu13082508.
195. Rosenberg-Hasson, Y.; Hansmann, L.; Liedtke, M.; Herschmann, I.; Maecker, H.T. Effects of Serum and Plasma Matrices on Multiplex Immunoassays. *Immunol. Res.* **2014**, *58*, 224–233, doi:10.1007/s12026-014-8491-6.

196. Selby, C. Interference in Immunoassay. *Ann. Clin. Biochem.* **1999**, *36*, 704–721, doi:10.1177/000456329903600603.

197. Kofanova, O.; Henry, E.; Aguilar Quesada, R.; Bulla, A.; Navarro Linares, H.; Lescuyer, P.; Shea, K.; Stone, M.; Tybring, G.; Bellora, C.; et al. IL8 and IL16 Levels Indicate Serum and Plasma Quality. *Clin. Chem. Lab. Med.* **2018**, *56*, 1054–1062, doi:10.1515/cclm-2017-1047.

198. Biancotto, A.; Feng, X.; Langweiler, M.; Young, N.S.; McCoy, J.P. Effect of Anticoagulants on Multiplexed Measurement of Cytokine/Chemokines in Healthy Subjects. *Cytokine* **2012**, *60*, 438–446, doi:10.1016/j.cyto.2012.05.019.

199. Guo, G.-H.; Dong, J.; Yuan, X.-H.; Dong, Z.-N.; Tian, Y.-P. Clinical Evaluation of the Levels of 12 Cytokines in Serum/Plasma under Various Storage Conditions Using Evidence Biochip Arrays. *Mol. Med. Rep.* **2013**, *7*, 775–780, doi:10.3892/mmr.2013.1263.

200. Lee, J.-E.; Kim, J.-W.; Han, B.-G.; Shin, S.-Y. Impact of Whole-Blood Processing Conditions on Plasma and Serum Concentrations of Cytokines. *Biopreserv. Biobank.* **2016**, *14*, 51–55, doi:10.1089/bio.2015.0059.

201. Schnabel, R.B.; Baumert, J.; Barbalic, M.; Dupuis, J.; Ellinor, P.T.; Durda, P.; Dehghan, A.; Bis, J.C.; Illig, T.; Morrison, A.C.; et al. Duffy Antigen Receptor for Chemokines (Darc) Polymorphism Regulates Circulating Concentrations of Monocyte Chemoattractant Protein-1 and Other Inflammatory Mediators. *Blood* **2010**, *115*, 5289–5299, doi:10.1182/blood-2009-05-221382.

202. Breen, E.C.; Reynolds, S.M.; Cox, C.; Jacobson, L.P.; Magpantay, L.; Mulder, C.B.; Dibben, O.; Margolick, J.B.; Bream, J.H.; Sambrano, E.; et al. Multisite Comparison of High-Sensitivity Multiplex Cytokine Assays. *Clin. Vaccine Immunol.* **2011**, *18*, 1229–1242, doi:10.1128/CVI.05032-11.

203. Knight, V.; Sepiashvili, L. Cytokine Testing and Challenges for Diagnostic and Clinical Monitoring Use. *J. Allergy Clin. Immunol.* **2025**, *155*, 410–413, doi:10.1016/j.jaci.2024.11.025.

204. Khan, S.S.; Smith, M.S.; Reda, D.; Suffredini, A.F.; McCoy, J.P.J. Multiplex Bead Array Assays for Detection of Soluble Cytokines: Comparisons of Sensitivity and Quantitative Values among Kits from Multiple Manufacturers. *Cytometry B. Clin. Cytom.* **2004**, *61*, 35–39, doi:10.1002/cyto.b.20021.

205. Knight, V.; Long, T.; Meng, Q.H.; Linden, M.A.; Rhoads, D.D. Variability in the Laboratory Measurement of Cytokines: A Longitudinal Summary of a College of American Pathologists Proficiency Testing Survey. *Arch. Pathol. Lab. Med.* **2020**, *144*, 1230–1233, doi:10.5858/arpa.2019-0519-CP.

206. Kaur, D.; Tallman, D.A.; Khosla, P. The Health Effects of Saturated Fats - the Role of Whole Foods and Dietary Patterns. *Diabetes Metab. Syndr.* **2020**, *14*, 151–153, doi:10.1016/j.dsx.2020.02.002.

207. Vincent, M.J.; Allen, B.; Palacios, O.M.; Haber, L.T.; Maki, K.C. Meta-Regression Analysis of the Effects of Dietary Cholesterol Intake on LDL and HDL Cholesterol. *Am. J. Clin. Nutr.* **2019**, *109*, 7–16, doi:10.1093/ajcn/nqy273.

208. Wu, G. Dietary Protein Intake and Human Health. *Food Funct.* **2016**, *7*, 1251–1265, doi:10.1039/c5fo01530h.

209. Toffaletti, J.G.; Hammett-Stabler, C.; Handel, E.A. Effect of Beef Ingestion by Humans on Plasma Concentrations of Creatinine, Urea, and Cystatin C. *Clin. Biochem.* **2018**, *58*, 26–31, doi:10.1016/j.clinbiochem.2018.05.016.

210. DiNicolantonio, J.J.; McCarty, M.F.; O'Keefe, J.H. Antioxidant Bilirubin Works in Multiple Ways to Reduce Risk for Obesity and Its Health Complications. *Open Hear.* **2018**, *5*, e000914, doi:10.1136/openhrt-2018-000914.

211. Kimura, Y.; Tsukui, D.; Kono, H. Uric Acid in Inflammation and the Pathogenesis of Atherosclerosis. *Int. J. Mol. Sci.* **2021**, *22*, doi:10.3390/ijms222212394.

212. Punzo, A.; Silla, A.; Fogacci, F.; Perillo, M.; Cicero, A.F.G.; Caliceti, C. Bile Acids and Bilirubin Role in Oxidative Stress and Inflammation in Cardiovascular Diseases. *Diseases* 2024, *12*, 103.

213. Zhao, X.; Duan, B.; Wu, J.; Huang, L.; Dai, S.; Ding, J.; Sun, M.; Lin, X.; Jiang, Y.; Sun, T.; et al. Bilirubin Ameliorates Osteoarthritis via Activating Nrf2/HO-1 Pathway and Suppressing NF-KB Signalling. *J. Cell. Mol. Med.* **2024**, *28*, e18173, doi:10.1111/jcmm.18173.

214. Li, Y.; Huang, B.; Ye, T.; Wang, Y.; Xia, D.; Qian, J. Physiological Concentrations of Bilirubin Control Inflammatory Response by Inhibiting NF-KB and Inflammasome Activation. *Int. Immunopharmacol.* **2020**, *84*, 106520, doi:10.1016/j.intimp.2020.106520.

215. Durante, W. Protective Role of Heme Oxygenase-1 against Inflammation in Atherosclerosis. *Front. Biosci. (Landmark Ed.* **2011**,

16, 2372–2388, doi:10.2741/3860.

216. Loboda, A.; Damulewicz, M.; Pyza, E.; Jozkowicz, A.; Dulak, J. Role of Nrf2/HO-1 System in Development, Oxidative Stress Response and Diseases: An Evolutionarily Conserved Mechanism. *Cell. Mol. Life Sci.* **2016**, *73*, 3221–3247, doi:10.1007/s00018-016-2223-0.

217. Pareek, S.; Flegle, A.S.; Boagni, D.; Kim, J.Y.; Yoo, D.; Trujillo-Ocampo, A.; Lee, S.-E.; Zhang, M.; Jon, S.; Im, J.S. Post Transplantation Bilirubin Nanoparticles Ameliorate Murine Graft Versus Host Disease via a Reduction of Systemic and Local Inflammation. *Front. Immunol.* **2022**, *13*, 893659, doi:10.3389/fimmu.2022.893659.

218. Roman, Y.M. The Role of Uric Acid in Human Health: Insights from the Uricase Gene. *J. Pers. Med.* **2023**, *13*, doi:10.3390/jpm13091409.

219. Allegrini, S.; Garcia-Gil, M.; Pesi, R.; Camici, M.; Tozzi, M.G. The Good, the Bad and the New about Uric Acid in Cancer. *Cancers (Basel).* **2022**, *14*, doi:10.3390/cancers14194959.

220. Ghaemi-Oskouie, F.; Shi, Y. The Role of Uric Acid as an Endogenous Danger Signal in Immunity and Inflammation. *Curr. Rheumatol. Rep.* **2011**, *13*, 160–166, doi:10.1007/s11926-011-0162-1.

221. Ridker, P.M. High-Sensitivity C-Reactive Protein and Cardiovascular Risk: Rationale for Screening and Primary Prevention. *Am. J. Cardiol.* **2003**, *92*, 17K-22K, doi:10.1016/s0002-9149(03)00774-4.

222. Nguyen, X.-M.T.; Lane, J.; Smith, B.R.; Nguyen, N.T. Changes in Inflammatory Biomarkers across Weight Classes in a Representative US Population: A Link between Obesity and Inflammation. *J. Gastrointest. Surg.* **2009**, *13*, 1205–1212, doi:10.1007/s11605-009-0904-9.

223. Park, H.S.; Park, J.Y.; Yu, R. Relationship of Obesity and Visceral Adiposity with Serum Concentrations of CRP, TNF-Alpha and IL-6. *Diabetes Res. Clin. Pract.* **2005**, *69*, 29–35, doi:10.1016/j.diabres.2004.11.007.

224. Slaats, J.; Ten Oever, J.; van de Veerdonk, F.L.; Netea, M.G. IL-1β/IL-6/CRP and IL-18/Ferritin: Distinct Inflammatory Programs in Infections. *PLoS Pathog.* **2016**, *12*, e1005973, doi:10.1371/journal.ppat.1005973.

225. Paolo, C.; W., C.D.; T., W.J.; T.H., Y.E. Release of C-Reactive Protein in Response to Inflammatory Cytokines by Human Adipocytes: Linking Obesity to Vascular Inflammation. *JACC* **2005**, *46*, 1112–1113, doi:10.1016/j.jacc.2005.06.017.

226. Colafrancesco, S.; Priori, R.; Alessandri, C.; Perricone, C.; Pendolino, M.; Picarelli, G.; Valesini, G. IL-18 Serum Level in Adult Onset Still's Disease: A Marker of Disease Activity. *Int. J. Inflam.* **2012**, *2012*, 156890, doi:10.1155/2012/156890.

227. Iskandar, A.; Mayashinta, D.K.; Robert, R.; Samsu, N.; Endharti, A.T.; Widjajanto, E. Correlation Between IL-8, C-Reactive Proteins (CRP) and Neutrophil to Lymphocyte Ratio (NLR) as Predictor of Mortality in COVID-19 Patients with Diabetes Mellitus Comorbidity. *Int. J. Gen. Med.* **2023**, *16*, 2349–2354, doi:10.2147/IJGM.S412070.

228. Deo, R.; Khera, A.; McGuire, D.K.; Murphy, S.A.; Meo Neto, J. de P.; Morrow, D.A.; de Lemos, J.A. Association among Plasma Levels of Monocyte Chemoattractant Protein-1, Traditional Cardiovascular Risk Factors, and Subclinical Atherosclerosis. *J. Am. Coll. Cardiol.* **2004**, *44*, 1812–1818, doi:10.1016/j.jacc.2004.07.047.

229. Kim, C.-S.; Park, H.-S.; Kawada, T.; Kim, J.-H.; Lim, D.; Hubbard, N.E.; Kwon, B.-S.; Erickson, K.L.; Yu, R. Circulating Levels of MCP-1 and IL-8 Are Elevated in Human Obese Subjects and Associated with Obesity-Related Parameters. *Int. J. Obes.* **2006**, *30*, 1347–1355, doi:10.1038/sj.ijo.0803259.

230. Valmorbida, A.; Longo, G.Z.; Nascimento, G.M.; de Oliveira, L.L.; de Moraes Trindade, E.B.S. Association between Cytokine Levels and Anthropometric Measurements: A Population-Based Study. *Br. J. Nutr.* **2023**, *129*, 1119–1126, doi:10.1017/S0007114522002148.

231. Bernstein, E.D.; Murasko, D.M. Effect of Age on Cytokine Production in Humans. *Age (Omaha).* **1998**, *21*, 137–151, doi:10.1007/s11357-998-0024-6.

232. Benini, R.; Nunes, P.R.P.; Orsatti, C.L.; Portari, G.V.; Orsatti, F.L. Influence of Sex on Cytokines, Heat Shock Protein and Oxidative Stress Markers in Response to an Acute Total Body Resistance Exercise Protocol. *J. Exerc. Sci. Fit.* **2015**, *13*, 1–7, doi:10.1016/j.jesf.2014.10.002.

233. Kim, H.O.H.-S.; Kim, H.O.H.-S.; Youn, J.-C.; Shin, E.-C.; Park, S. Serum Cytokine Profiles in Healthy Young and Elderly

Population Assessed Using Multiplexed Bead-Based Immunoassays. *J. Transl. Med.* **2011**, *9*, 113, doi:10.1186/1479-5876-9-113.
234. Wilkinson, N.M.; Chen, H.-C.; Lechner, M.G.; Su, M.A. Sex Differences in Immunity. *Annu. Rev. Immunol.* **2022**, *40*, 75–94, doi:https://doi.org/10.1146/annurev-immunol-101320-125133.
235. Bernardi, S.; Toffoli, B.; Tonon, F.; Francica, M.; Campagnolo, E.; Ferretti, T.; Comar, S.; Giudici, F.; Stenner, E.; Fabris, B. Sex Differences in Proatherogenic Cytokine Levels. *Int. J. Mol. Sci.* 2020, *21*, 3861.
236. Beenakker, K.G.M.; Westendorp, R.G.J.; de Craen, A.J.M.; Chen, S.; Raz, Y.; Ballieux, B.E.P.B.; Nelissen, R.G.H.H.; Later, A.F.L.; Huizinga, T.W.; Slagboom, P.E.; et al. Men Have a Stronger Monocyte-Derived Cytokine Production Response upon Stimulation with the Gram-Negative Stimulus Lipopolysaccharide than Women: A Pooled Analysis Including 15 Study Populations. *J. Innate Immun.* **2020**, *12*, 142–153, doi:10.1159/000499840.
237. Rountree, W.; Lynch, H.E.; Denny, T.N.; Sempowski, G.D.; Macintyre, A.N. Sources of Variability in Luminex Bead-Based Cytokine Assays: Evidence from Twelve Years of Multi-Site Proficiency Testing. *J. Immunol. Methods* **2024**, *531*, 113699, doi:10.1016/j.jim.2024.113699.
238. Lasseter, H.C.; Provost, A.C.; Chaby, L.E.; Daskalakis, N.P.; Haas, M.; Jeromin, A. Cross-Platform Comparison of Highly Sensitive Immunoassay Technologies for Cytokine Markers: Platform Performance in Post-Traumatic Stress Disorder and Parkinson's Disease. *Cytokine X* **2020**, *2*, 100027, doi:https://doi.org/10.1016/j.cytox.2020.100027.
239. Arioz, B.I.; Cotuk, A.; Yaka, E.C.; Genc, S. Proximity Extension Assay-Based Proteomics Studies in Neurodegenerative Disorders and Multiple Sclerosis. *Eur. J. Neurosci.* **2024**, *59*, 1348–1358, doi:https://doi.org/10.1111/ejn.16226.
240. Kupcova Skalnikova, H.; Cizkova, J.; Cervenka, J.; Vodicka, P. Advances in Proteomic Techniques for Cytokine Analysis: Focus on Melanoma Research. *Int. J. Mol. Sci.* **2017**, *18*, doi:10.3390/ijms18122697.
241. Leng, S.X.; McElhaney, J.E.; Walston, J.D.; Xie, D.; Fedarko, N.S.; Kuchel, G.A. ELISA and Multiplex Technologies for Cytokine Measurement in Inflammation and Aging Research. *J. Gerontol. A. Biol. Sci. Med. Sci.* **2008**, *63*, 879–884, doi:10.1093/gerona/63.8.879.
242. Dabitao, D.; Margolick, J.B.; Lopez, J.; Bream, J.H. Multiplex Measurement of Proinflammatory Cytokines in Human Serum: Comparison of the Meso Scale Discovery Electrochemiluminescence Assay and the Cytometric Bead Array. *J. Immunol. Methods* **2011**, *372*, 71–77, doi:10.1016/j.jim.2011.06.033.
243. Günther, A.; Becker, M.; Göpfert, J.; Joos, T.; Schneiderhan-Marra, N. Comparison of Bead-Based Fluorescence Versus Planar Electrochemiluminescence Multiplex Immunoassays for Measuring Cytokines in Human Plasma. *Front. Immunol.* **2020**, *11*, 572634, doi:10.3389/fimmu.2020.572634.
244. Hennø, L.T.; Storjord, E.; Christiansen, D.; Bergseth, G.; Ludviksen, J.K.; Fure, H.; Barene, S.; Nielsen, E.W.; Mollnes, T.E.; Brekke, O.-L. Effect of the Anticoagulant, Storage Time and Temperature of Blood Samples on the Concentrations of 27 Multiplex Assayed Cytokines - Consequences for Defining Reference Values in Healthy Humans. *Cytokine* **2017**, *97*, 86–95, doi:10.1016/j.cyto.2017.05.014.
245. Deru, L.S.; Duersch, A.F.; Cleverly, S.C.; Chamberlain, H.D.; Gipson, E.Z.; Graves, P.G.; Jacobsen, C.G.; Tucker, L.A.; Bailey, B.W. The Effects of Combined Fasting and Exercise on Inflammatory Cytokine Concentrations in Healthy Adults: A Randomized Crossover Study. *Int. J. Exerc. Sci.* **2025**, *18*, 1252–1268, doi:10.70252/APHT9483.
246. Duffy, D.; Rouilly, V.; Libri, V.; Hasan, M.; Beitz, B.; David, M.; Urrutia, A.; Bisiaux, A.; Labrie, S.T.; Dubois, A.; et al. Functional Analysis via Standardized Whole-Blood Stimulation Systems Defines the Boundaries of a Healthy Immune Response to Complex Stimuli. *Immunity* **2014**, *40*, 436–450, doi:10.1016/j.immuni.2014.03.002.
247. European Food Safety Authority Guidance on the EU Menu Methodology. *EFSA J.* **2014**, *12*, 3944, doi:10.2903/j.efsa.2014.3944.
248. Dodd, K.W.; Guenther, P.M.; Freedman, L.S.; Subar, A.F.; Kipnis, V.; Midthune, D.; Tooze, J.A.; Krebs-Smith, S.M. Statistical Methods for Estimating Usual Intake of Nutrients and Foods: A Review of the Theory. *J. Am. Diet. Assoc.* **2006**, *106*, 1640–1650, doi:10.1016/j.jada.2006.07.011.
249. Zabetakis, I.; Matthys, C.; Tsoupras, A. Editorial: Coronavirus Disease (COVID-19): Diet, Inflammation and Nutritional Status. *Front. Nutr.* **2021**, *Volume 8*, 760720, doi:10.3389/fnut.2021.760720.

250. Merino, J.; Joshi, A.D.; Nguyen, L.H.; Leeming, E.R.; Mazidi, M.; Drew, D.A.; Gibson, R.; Graham, M.S.; Lo, C.-H.; Capdevila, J.; et al. Diet Quality and Risk and Severity of COVID-19: A Prospective Cohort Study. *Gut* **2021**, *70*, 2096–2104, doi:10.1136/gutjnl-2021-325353.
251. Del Valle, D.M.; Kim-Schulze, S.; Huang, H.-H.; Beckmann, N.D.; Nirenberg, S.; Wang, B.; Lavin, Y.; Swartz, T.H.; Madduri, D.; Stock, A.; et al. An Inflammatory Cytokine Signature Predicts COVID-19 Severity and Survival. *Nat. Med.* **2020**, *26*, 1636–1643, doi:10.1038/s41591-020-1051-9.
252. Lucas, C.; Wong, P.; Klein, J.; Castro, T.B.R.; Silva, J.; Sundaram, M.; Ellingson, M.K.; Mao, T.; Oh, J.E.; Israelow, B.; et al. Longitudinal Analyses Reveal Immunological Misfiring in Severe COVID-19. *Nature* **2020**, *584*, 463–469, doi:10.1038/s41586-020-2588-y.
253. Kim, H.; Rebholz, C.M.; Hegde, S.; LaFiura, C.; Raghavan, M.; Lloyd, J.F.; Cheng, S.; Seidelmann, S.B. Plant-Based Diets, Pescatarian Diets and COVID-19 Severity: A Population-Based Case-Control Study in Six Countries. *BMJ Nutr. Prev. Heal.* **2021**, *4*, null, doi:10.1136/bmjnph-2021-000272.
254. Hou, Y.-C.; Su, W.-L.; Chao, Y.-C. COVID-19 Illness Severity in the Elderly in Relation to Vegetarian and Non-Vegetarian Diets: A Single-Center Experience. *Front. Nutr.* **2022**, *9*, 837458, doi:10.3389/fnut.2022.837458.

References only related to supplementary material

255. Napetschnig, J.; Wu, H. Molecular Basis of NF-KB Signaling. *Annu. Rev. Biophys.* **2013**, *42*, 443–468, doi:10.1146/annurev-biophys-083012-130338.
256. Iacobazzi, D.; Convertini, P.; Todisco, S.; Santarsiero, A.; Iacobazzi, V.; Infantino, V. New Insights into NF-KB Signaling in Innate Immunity: Focus on Immunometabolic Crosstalks. *Biology (Basel).* **2023**, *12*, doi:10.3390/biology12060776.
257. Ahmed, S.M.U.; Luo, L.; Namani, A.; Wang, X.J.; Tang, X. Nrf2 Signaling Pathway: Pivotal Roles in Inflammation. *Biochim. Biophys. Acta - Mol. Basis Dis.* **2017**, *1863*, 585–597, doi:https://doi.org/10.1016/j.bbadis.2016.11.005.
258. Lu, Y.-C.; Yeh, W.-C.; Ohashi, P.S. LPS/TLR4 Signal Transduction Pathway. *Cytokine* **2008**, *42*, 145–151, doi:10.1016/j.cyto.2008.01.006.
259. Dennis, E.A.; Norris, P.C. Eicosanoid Storm in Infection and Inflammation. *Nat. Rev. Immunol.* **2015**, *15*, 511–523, doi:10.1038/nri3859.
260. Sheppe, A.E.F.; Edelmann, M.J. Roles of Eicosanoids in Regulating Inflammation and Neutrophil Migration as an Innate Host Response to Bacterial Infections. *Infect. Immun.* **2021**, *89*, e0009521, doi:10.1128/IAI.00095-21.
261. Rogero, M.M.; Calder, P.C. Obesity, Inflammation, Toll-Like Receptor 4 and Fatty Acids. *Nutrients* 2018, *10*, 432.
262. Zhang, Y.; Chen, H.; Zhang, W.; Cai, Y.; Shan, P.; Wu, D.; Zhang, B.; Liu, H.; Khan, Z.A.; Liang, G. Arachidonic Acid Inhibits Inflammatory Responses by Binding to Myeloid Differentiation Factor-2 (MD2) and Preventing MD2/Toll-like Receptor 4 Signaling Activation. *Biochim. Biophys. acta. Mol. basis Dis.* **2020**, *1866*, 165683, doi:10.1016/j.bbadis.2020.165683.
263. Calder, P.C. Omega-3 Fatty Acids and Inflammatory Processes: From Molecules to Man. *Biochem. Soc. Trans.* **2017**, *45*, 1105–1115, doi:10.1042/BST20160474.
264. Choy, K.W.; Murugan, D.; Leong, X.-F.; Abas, R.; Alias, A.; Mustafa, M.R. Flavonoids as Natural Anti-Inflammatory Agents Targeting Nuclear Factor-Kappa B (NFκB) Signaling in Cardiovascular Diseases: A Mini Review. *Front. Pharmacol.* **2019**, *10*, 1295, doi:10.3389/fphar.2019.01295.
265. Hussain, T.; Murtaza, G.; Yang, H.; Kalhoro, M.S.; Kalhoro, D.H. Exploiting Anti-Inflammation Effects of Flavonoids in Chronic Inflammatory Diseases. *Curr. Pharm. Des.* **2020**, *26*, 2610–2619, doi:10.2174/1381612826666200408101550.
266. Malaguarnera, L. Influence of Resveratrol on the Immune Response. *Nutrients* **2019**, *11*, doi:10.3390/nu11050946.
267. Smith, P.M.; Howitt, M.R.; Panikov, N.; Michaud, M.; Gallini, C.A.; Bohlooly-Y, M.; Glickman, J.N.; Garrett, W.S. The Microbial Metabolites, Short-Chain Fatty Acids, Regulate Colonic Treg Cell Homeostasis. *Science* **2013**, *341*, 569–573, doi:10.1126/science.1241165.

268. Vinolo, M.A.R.; Rodrigues, H.G.; Nachbar, R.T.; Curi, R. Regulation of Inflammation by Short Chain Fatty Acids. *Nutrients* **2011**, *3*, 858–876, doi:10.3390/nu3100858.
269. Koh, A.; De Vadder, F.; Kovatcheva-Datchary, P.; Bäckhed, F. From Dietary Fiber to Host Physiology: Short-Chain Fatty Acids as Key Bacterial Metabolites. *Cell* **2016**, *165*, 1332–1345, doi:10.1016/j.cell.2016.05.041.
270. Corrêa-Oliveira, R.; Fachi, J.L.; Vieira, A.; Sato, F.T.; Vinolo, M.A.R. Regulation of Immune Cell Function by Short-Chain Fatty Acids. *Clin. Transl. Immunol.* **2016**, *5*, e73, doi:10.1038/cti.2016.17.
271. Hays, K.E.; Pfaffinger, J.M.; Ryznar, R. The Interplay between Gut Microbiota, Short-Chain Fatty Acids, and Implications for Host Health and Disease. *Gut Microbes* **2024**, *16*, 2393270, doi:10.1080/19490976.2024.2393270.
272. Parada Venegas, D.; De la Fuente, M.K.; Landskron, G.; González, M.J.; Quera, R.; Dijkstra, G.; Harmsen, H.J.M.; Faber, K.N.; Hermoso, M.A. Short Chain Fatty Acids (SCFAs)-Mediated Gut Epithelial and Immune Regulation and Its Relevance for Inflammatory Bowel Diseases. *Front. Immunol.* **2019**, *10*, 277, doi:10.3389/fimmu.2019.00277.
273. Zhang, D.; Jian, Y.-P.; Zhang, Y.-N.; Li, Y.; Gu, L.-T.; Sun, H.-H.; Liu, M.-D.; Zhou, H.-L.; Wang, Y.-S.; Xu, Z.-X. Short-Chain Fatty Acids in Diseases. *Cell Commun. Signal.* **2023**, *21*, 212, doi:10.1186/s12964-023-01219-9.
274. Charles-Messance, H.; Mitchelson, K.A.J.; De Marco Castro, E.; Sheedy, F.J.; Roche, H.M. Regulating Metabolic Inflammation by Nutritional Modulation. *J. Allergy Clin. Immunol.* **2020**, *146*, 706–720, doi:10.1016/j.jaci.2020.08.013.
275. Yang, Y.; Wang, H.; Kouadir, M.; Song, H.; Shi, F. Recent Advances in the Mechanisms of NLRP3 Inflammasome Activation and Its Inhibitors. *Cell Death Dis.* **2019**, *10*, 128, doi:10.1038/s41419-019-1413-8.
276. Swanson, K. V; Deng, M.; Ting, J.P.-Y. The NLRP3 Inflammasome: Molecular Activation and Regulation to Therapeutics. *Nat. Rev. Immunol.* **2019**, *19*, 477–489, doi:10.1038/s41577-019-0165-0.
277. Blevins, H.M.; Xu, Y.; Biby, S.; Zhang, S. The NLRP3 Inflammasome Pathway: A Review of Mechanisms and Inhibitors for the Treatment of Inflammatory Diseases. *Front. Aging Neurosci.* **2022**, *14*, 879021, doi:10.3389/fnagi.2022.879021.
278. Anand, P.K. Lipids, Inflammasomes, Metabolism, and Disease. *Immunol. Rev.* **2020**, *297*, 108–122, doi:10.1111/imr.12891.
279. Karasawa, T.; Kawashima, A.; Usui-Kawanishi, F.; Watanabe, S.; Kimura, H.; Kamata, R.; Shirasuna, K.; Koyama, Y.; Sato-Tomita, A.; Matsuzaka, T.; et al. Saturated Fatty Acids Undergo Intracellular Crystallization and Activate the NLRP3 Inflammasome in Macrophages. *Arterioscler. Thromb. Vasc. Biol.* **2018**, *38*, 744–756, doi:10.1161/ATVBAHA.117.310581.
280. Yuan, X.; Wang, L.; Bhat, O.M.; Lohner, H.; Li, P.-L. Differential Effects of Short Chain Fatty Acids on Endothelial Nlrp3 Inflammasome Activation and Neointima Formation: Antioxidant Action of Butyrate. *Redox Biol.* **2018**, *16*, 21–31, doi:https://doi.org/10.1016/j.redox.2018.02.007.
281. Yi, C.; Sun, W.; Ding, L.; Yan, M.; Sun, C.; Qiu, C.; Wang, D.; Wu, L. Short-Chain Fatty Acids Weaken Ox-LDL-Induced Cell Inflammatory Injury by Inhibiting the NLRP3/Caspase-1 Pathway and Affecting Cellular Metabolism in THP-1 Cells. *Molecules* **2022**, *27*, doi:10.3390/molecules27248801.
282. Olcum, M.; Tastan, B.; Ercan, I.; Eltutan, I.B.; Genc, S. Inhibitory Effects of Phytochemicals on NLRP3 Inflammasome Activation: A Review. *Phytomedicine* **2020**, *75*, 153238, doi:10.1016/j.phymed.2020.153238.
283. Tőzsér, J.; Benkő, S. Natural Compounds as Regulators of NLRP3 Inflammasome-Mediated IL-1β Production. *Mediators Inflamm.* **2016**, *2016*, 5460302, doi:10.1155/2016/5460302.
284. Bellezza, I.; Giambanco, I.; Minelli, A.; Donato, R. Nrf2-Keap1 Signaling in Oxidative and Reductive Stress. *Biochim. Biophys. acta. Mol. cell Res.* **2018**, *1865*, 721–733, doi:10.1016/j.bbamcr.2018.02.010.
285. Omaye, A.T.; Omaye, S.T. Caveats for the Good and Bad of Dietary Red Meat. *Antioxidants (Basel, Switzerland)* **2019**, *8*, doi:10.3390/antiox8110544.
286. Turner, N.D.; Lloyd, S.K. Association between Red Meat Consumption and Colon Cancer: A Systematic Review of Experimental Results. *Exp. Biol. Med. (Maywood).* **2017**, *242*, 813–839, doi:10.1177/1535370217693117.
287. Dutt, S.; Hamza, I.; Bartnikas, T.B. Molecular Mechanisms of Iron and Heme Metabolism. *Annu. Rev. Nutr.* **2022**, *42*, 311–335, doi:https://doi.org/10.1146/annurev-nutr-062320-112625.
288. Atsaves, V.; Leventaki, V.; Rassidakis, G.Z.; Claret, F.X. AP-1 Transcription Factors as Regulators of Immune Responses in

Cancer. *Cancers (Basel).* **2019**, *11*, doi:10.3390/cancers11071037.

289. Arthur, J.S.C.; Ley, S.C. Mitogen-Activated Protein Kinases in Innate Immunity. *Nat. Rev. Immunol.* **2013**, *13*, 679–692, doi:10.1038/nri3495.

290. Schnoegl, D.; Hiesinger, A.; Huntington, N.D.; Gotthardt, D. AP-1 Transcription Factors in Cytotoxic Lymphocyte Development and Antitumor Immunity. *Curr. Opin. Immunol.* **2023**, *85*, 102397, doi:10.1016/j.coi.2023.102397.

291. Smith-Garvin, J.E.; Koretzky, G.A.; Jordan, M.S. T Cell Activation. *Annu. Rev. Immunol.* **2009**, *27*, 591–619, doi:https://doi.org/10.1146/annurev.immunol.021908.132706.

292. Dal Porto, J.M.; Gauld, S.B.; Merrell, K.T.; Mills, D.; Pugh-Bernard, A.E.; Cambier, J. B Cell Antigen Receptor Signaling 101. *Mol. Immunol.* **2004**, *41*, 599–613, doi:https://doi.org/10.1016/j.molimm.2004.04.008.

293. Fujioka, S.; Niu, J.; Schmidt, C.; Sclabas, G.M.; Peng, B.; Uwagawa, T.; Li, Z.; Evans, D.B.; Abbruzzese, J.L.; Chiao, P.J. NF-KB and AP-1 Connection: Mechanism of NF-KB-Dependent Regulation of AP-1 Activity. *Mol. Cell. Biol.* **2004**, *24*, 7806–7819, doi:10.1128/MCB.24.17.7806-7819.2004.

294. Zhang, S.; Kaplan, M.H. The P38 Mitogen-Activated Protein Kinase Is Required for IL-12-Induced IFN-Gamma Expression. *J. Immunol.* **2000**, *165*, 1374–1380, doi:10.4049/jimmunol.165.3.1374.

295. Ochayon, D.E.; Ali, A.; Alarcon, P.C.; Krishnamurthy, D.; Kottyan, L.C.; Borchers, M.T.; Waggoner, S.N. IL-33 Promotes Type 1 Cytokine Expression via P38 MAPK in Human NK Cells. *J. Leukoc. Biol.* **2020**, *107*, 663–671, doi:10.1002/JLB.3A0120-379RR.

296. Ullrich, K.A.-M.; Schulze, L. Lou; Paap, E.-M.; Müller, T.M.; Neurath, M.F.; Zundler, S. Immunology of IL-12: An Update on Functional Activities and Implications for Disease. *EXCLI J.* **2020**, *19*, 1563–1589, doi:10.17179/excli2020-3104.

297. Srivastava, S.; Pelloso, D.; Feng, H.; Voiles, L.; Lewis, D.; Haskova, Z.; Whitacre, M.; Trulli, S.; Chen, Y.-J.; Toso, J.; et al. Effects of Interleukin-18 on Natural Killer Cells: Costimulation of Activation through Fc Receptors for Immunoglobulin. *Cancer Immunol. Immunother.* **2013**, *62*, 1073–1082, doi:10.1007/s00262-013-1403-0.

298. Kondadasula, S.V.; Roda, J.M.; Parihar, R.; Yu, J.; Lehman, A.; Caligiuri, M.A.; Tridandapani, S.; Burry, R.W.; Carson, W.E. 3rd Colocalization of the IL-12 Receptor and FcgammaRIIIa to Natural Killer Cell Lipid Rafts Leads to Activation of ERK and Enhanced Production of Interferon-Gamma. *Blood* **2008**, *111*, 4173–4183, doi:10.1182/blood-2007-01-068908.

299. Iyer, S.S.; Cheng, G. Role of Interleukin 10 Transcriptional Regulation in Inflammation and Autoimmune Disease. *Crit. Rev. Immunol.* **2012**, *32*, 23–63, doi:10.1615/critrevimmunol.v32.i1.30.

300. Takahashi, A.; de Andrés, M.C.; Hashimoto, K.; Itoi, E.; Oreffo, R.O.C. Epigenetic Regulation of Interleukin-8, an Inflammatory Chemokine, in Osteoarthritis. *Osteoarthr. Cartil.* **2015**, *23*, 1946–1954, doi:10.1016/j.joca.2015.02.168.

301. Jin, Y.; Wang, Y.; Yang, R. Chemokine Ligand 2: Beyond Chemotaxis-a Multifaceted Role in Tumor Progression. *Front. Immunol.* **2025**, *16*, 1685474, doi:10.3389/fimmu.2025.1685474.

302. Zhang, X.; Liu, Z.; Li, W.; Kang, Y.; Xu, Z.; Li, X.; Gao, Y.; Qi, Y. MAPKs/AP-1, Not NF-KB, Is Responsible for MCP-1 Production in TNF-α-Activated Adipocytes. *Adipocyte* **2022**, *11*, 477–486, doi:10.1080/21623945.2022.2107786.

303. Calder, P.C.; Ahluwalia, N.; Brouns, F.; Buetler, T.; Clement, K.; Cunningham, K.; Esposito, K.; Jönsson, L.S.; Kolb, H.; Lansink, M.; et al. Dietary Factors and Low-Grade Inflammation in Relation to Overweight and Obesity. *Br. J. Nutr.* **2011**, *106 Suppl*, S5-78, doi:10.1017/S0007114511005460.

304. Korbecki, J.; Bajdak-Rusinek, K. The Effect of Palmitic Acid on Inflammatory Response in Macrophages: An Overview of Molecular Mechanisms. *Inflamm. Res. Off. J. Eur. Histamine Res. Soc. ... [et al.]* **2019**, *68*, 915–932, doi:10.1007/s00011-019-01273-5.

305. Lancaster, G.I.; Langley, K.G.; Berglund, N.A.; Kammoun, H.L.; Reibe, S.; Estevez, E.; Weir, J.; Mellett, N.A.; Pernes, G.; Conway, J.R.W.; et al. Evidence That TLR4 Is Not a Receptor for Saturated Fatty Acids but Mediates Lipid-Induced Inflammation by Reprogramming Macrophage Metabolism. *Cell Metab.* **2018**, *27*, 1096-1110.e5, doi:10.1016/j.cmet.2018.03.014.

306. Liu, W.; Ouyang, X.; Yang, J.; Liu, J.; Li, Q.; Gu, Y.; Fukata, M.; Lin, T.; He, J.C.; Abreu, M.; et al. AP-1 Activated by Toll-like Receptors Regulates Expression of IL-23 P19. *J. Biol. Chem.* **2009**, *284*, 24006–24016, doi:10.1074/jbc.M109.025528.

307. Bahman, F.J.; Al-Roub, A.; Akther, N.; Al Madhoun, A.; Al-Mulla, F.; Ahmad, R. Insulin Potentiates Lipopolysaccharide-

Induced IL-6 Expression through Epigenetic Remodeling in Adipocytes: In Vitro and in Vivo Mechanistic Study. *Front. Immunol.* **2026**, *Volume 17*, doi:10.3389/fimmu.2026.1840850.

308. Zaloga, G.P. Narrative Review of N-3 Polyunsaturated Fatty Acid Supplementation upon Immune Functions, Resolution Molecules and Lipid Peroxidation. *Nutrients* 2021, *13*, 662.

309. Calder, P.C. N-3 PUFA and Inflammation: From Membrane to Nucleus and from Bench to Bedside. *Proc. Nutr. Soc.* **2020**, 1–13, doi:10.1017/S0029665120007077.

310. Kimball, S.R.; Jefferson, L.S. Signaling Pathways and Molecular Mechanisms through Which Branched-Chain Amino Acids Mediate Translational Control of Protein Synthesis. *J. Nutr.* **2006**, *136*, 227S-31S, doi:10.1093/jn/136.1.227S.

311. Cruzat, V.; Macedo Rogero, M.; Noel Keane, K.; Curi, R.; Newsholme, P. Glutamine: Metabolism and Immune Function, Supplementation and Clinical Translation. *Nutrients* **2018**, *10*, doi:10.3390/nu10111564.

312. Lu, S.C. Glutathione Synthesis. *Biochim. Biophys. Acta* **2013**, *1830*, 3143–3153, doi:10.1016/j.bbagen.2012.09.008.

313. Maynard, A.G.; Petrova, B.; Kanarek, N. Notes from the 2022 Folate, Vitamin B12, and One-Carbon Metabolism Conference. *Metabolites* 2023, *13*, 486.

314. Ristori, S.; Bertoni, G.; Bientinesi, E.; Monti, D. The Role of Nutraceuticals and Functional Foods in Mitigating Cellular Senescence and Its Related Aspects: A Key Strategy for Delaying or Preventing Aging and Neurodegenerative Disorders. *Nutrients* **2025**, *17*, doi:10.3390/nu17111837.

315. Su, Z.-Y.; Shu, L.; Khor, T.O.; Lee, J.H.; Fuentes, F.; Kong, A.-N.T. A Perspective on Dietary Phytochemicals and Cancer Chemoprevention: Oxidative Stress, Nrf2, and Epigenomics. *Top. Curr. Chem.* **2013**, *329*, 133–162, doi:10.1007/128_2012_340.

316. Vargas-Mendoza, N.; Sandoval-Gallegos, E.M.; Madrigal-Santillán, E.O.; Morales-Martínez, M.; Soriano-Ursúa, M.A.; Angeles-Valencia, M.; Morales-González, Á.; Portillo-Reyes, J.; Morales-González, J.A. The Cytoprotective Activity of Nrf2 Is Regulated by Phytochemicals (Sulforaphane, Curcumin, and Silymarin) BT - Plant Antioxidants and Health. In; Ekiert, H.M., Ramawat, K.G., Arora, J., Eds.; Springer International Publishing: Cham, 2020; pp. 1–52 ISBN 978-3-030-45299-5.

317. Fagbohun, O.F.; Gillies, C.R.; Murphy, K.P.J.; Rupasinghe, H.P.V. Role of Antioxidant Vitamins and Other Micronutrients on Regulations of Specific Genes and Signaling Pathways in the Prevention and Treatment of Cancer. *Int. J. Mol. Sci.* 2023, *24*, 6092.

318. Yahfoufi, N.; Alsadi, N.; Jambi, M.; Matar, C. The Immunomodulatory and Anti-Inflammatory Role of Polyphenols. *Nutrients* **2018**, *10*, doi:10.3390/nu10111618.

319. Pollizzi, K.N.; Powell, J.D. Regulation of T Cells by MTOR: The Known Knowns and the Known Unknowns. *Trends Immunol.* **2015**, *36*, 13–20, doi:10.1016/j.it.2014.11.005.

320. Jones, R.G.; Pearce, E.J. MenTORing Immunity: MTOR Signaling in the Development and Function of Tissue-Resident Immune Cells. *Immunity* **2017**, *46*, 730–742, doi:10.1016/j.immuni.2017.04.028.

321. Saleiro, D.; Platanias, L.C. Intersection of MTOR and STAT Signaling in Immunity. *Trends Immunol.* **2015**, *36*, 21–29, doi:10.1016/j.it.2014.10.006.

322. Saxton, R.A.; Sabatini, D.M. MTOR Signaling in Growth, Metabolism, and Disease. *Cell* **2017**, *168*, 960–976, doi:10.1016/j.cell.2017.02.004.

323. Weichhart, T.; Hengstschläger, M.; Linke, M. Regulation of Innate Immune Cell Function by MTOR. *Nat. Rev. Immunol.* **2015**, *15*, 599–614, doi:10.1038/nri3901.

324. Powell, J.D.; Pollizzi, K.N.; Heikamp, E.B.; Horton, M.R. Regulation of Immune Responses by MTOR. *Annu. Rev. Immunol.* **2012**, *30*, 39–68, doi:10.1146/annurev-immunol-020711-075024.

325. Zeng, H.; Chi, H. MTOR Signaling and Transcriptional Regulation in T Lymphocytes. *Transcription* **2014**, *5*, e28263, doi:10.4161/trns.28263.

326. Yang, K.; Chi, H. MTOR and Metabolic Pathways in T Cell Quiescence and Functional Activation. *Semin. Immunol.* **2012**, *24*, 421–428, doi:10.1016/j.smim.2012.12.004.

327. Chi, H. Regulation and Function of MTOR Signalling in T Cell Fate Decisions. *Nat. Rev. Immunol.* **2012**, *12*, 325–338,

doi:10.1038/nri3198.
328. Ananieva, E.A.; Powell, J.D.; Hutson, S.M. Leucine Metabolism in T Cell Activation: MTOR Signaling and Beyond. *Adv. Nutr.* **2016**, *7*, 798S-805S, doi:10.3945/an.115.011221.
329. Shakoor, H.; Feehan, J.; Apostolopoulos, V.; Platat, C.; Al Dhaheri, A.S.; Ali, H.I.; Ismail, L.C.; Bosevski, M.; Stojanovska, L. Immunomodulatory Effects of Dietary Polyphenols. *Nutrients* 2021, *13*, 728.
330. Gasaly, N.; de Vos, P.; Hermoso, M.A. Impact of Bacterial Metabolites on Gut Barrier Function and Host Immunity: A Focus on Bacterial Metabolism and Its Relevance for Intestinal Inflammation. *Front. Immunol.* **2021**, *12*, 658354, doi:10.3389/fimmu.2021.658354.
331. Deane, J.A.; Fruman, D.A. Phosphoinositide 3-Kinase: Diverse Roles in Immune Cell Activation. *Annu. Rev. Immunol.* **2004**, *22*, 563–598, doi:10.1146/annurev.immunol.22.012703.104721.
332. Zhang, Y.; Wang, X.; Yang, H.; Liu, H.; Lu, Y.; Han, L.; Liu, G. Kinase AKT Controls Innate Immune Cell Development and Function. *Immunology* **2013**, *140*, 143–152, doi:10.1111/imm.12123.
333. Beurel, E.; Grieco, S.F.; Jope, R.S. Glycogen Synthase Kinase-3 (GSK3): Regulation, Actions, and Diseases. *Pharmacol. Ther.* **2015**, *148*, 114–131, doi:10.1016/j.pharmthera.2014.11.016.
334. Guerau-de-Arellano, M.; Piedra-Quintero, Z.L.; Tsichlis, P.N. Akt Isoforms in the Immune System. *Front. Immunol.* **2022**, *13*, 990874, doi:10.3389/fimmu.2022.990874.
335. Uti, D.E.; Atangwho, I.J.; Omang, W.A.; Alum, E.U.; Obeten, U.N.; Udeozor, P.A.; Agada, S.A.; Bawa, I.; Ogbu, C.O. Cytokines as Key Players in Obesity Low Grade Inflammation and Related Complications. *Obes. Med.* **2025**, *54*, 100585, doi:https://doi.org/10.1016/j.obmed.2025.100585.
336. Calder, P.C. Dietary Factors and Low-Grade Inflammation in Relation to Overweight and Obesity Revisted. *Br. J. Nutr.* **2022**, *127*, 1455–1457, doi:10.1017/S0007114522000782.
337. Maida, C.D.; Scaglione, S.; Norrito, R.L.; Daidone, M.; Pacinella, G.; Marchese, A.; Vutano, F.; Cassataro, G.; Dell'Ajra, L.; Ferrantelli, S.; et al. Dietary Modulation of Inflammatory and Oxidative Pathways in Type 2 Diabetes: Biomarkers and Cardiorenal Outcomes. *Nutrients* 2026, *18*, 1592.
338. Alwarawrah, Y.; Kiernan, K.; MacIver, N.J. Changes in Nutritional Status Impact Immune Cell Metabolism and Function. *Front. Immunol.* **2018**, *9*, 1055, doi:10.3389/fimmu.2018.01055.
339. Zhao, F.; Ci, X.; Man, X.; Li, J.; Wei, Z.; Zhang, S. Food-Derived Pharmacological Modulators of the Nrf2/ARE Pathway: Their Role in the Treatment of Diseases. *Molecules* **2021**, *26*, doi:10.3390/molecules26041016.
340. Smith, R.E.; Tran, K.; Smith, C.C.; McDonald, M.; Shejwalkar, P.; Hara, K. The Role of the Nrf2/ARE Antioxidant System in Preventing Cardiovascular Diseases. *Dis. (Basel, Switzerland)* **2016**, *4*, doi:10.3390/diseases4040034.
341. Zhang, Q.; Liu, J.; Duan, H.; Li, R.; Peng, W.; Wu, C. Activation of Nrf2/HO-1 Signaling: An Important Molecular Mechanism of Herbal Medicine in the Treatment of Atherosclerosis via the Protection of Vascular Endothelial Cells from Oxidative Stress. *J. Adv. Res.* **2021**, *34*, 43–63, doi:10.1016/j.jare.2021.06.023.
342. Abrescia, P.; Treppiccione, L.; Rossi, M.; Bergamo, P. Modulatory Role of Dietary Polyunsaturated Fatty Acids in Nrf2-Mediated Redox Homeostasis. *Prog. Lipid Res.* **2020**, *80*, 101066, doi:https://doi.org/10.1016/j.plipres.2020.101066.
343. Costa, V.B.; de Matos, I.A.F.; Nogueira, I.R.G.; de Godoi, M.A.; Leite, F.R.M.; Guimarães-Stabili, M.R. Nrf2 Activation in Inflammatory Diseases: A Review of Natural and Synthetic Modulators. *Oxid. Med. Cell. Longev.* 2026, *2026*.
344. Shukla, V.; Lad, V. Short Review: Nrf2 and Omega 3 for Human Health. *Insights Chem. Biochem.* **2022**, *2*, 000534.
345. Stefanson, A.L.; Bakovic, M. Dietary Regulation of Keap1/Nrf2/ARE Pathway: Focus on Plant-Derived Compounds and Trace Minerals. *Nutrients* **2014**, *6*, 3777–3801, doi:10.3390/nu6093777.
346. Gutiérrez-Cuevas, J.; Galicia-Moreno, M.; Monroy-Ramírez, H.C.; Sandoval-Rodriguez, A.; García-Bañuelos, J.; Santos, A.; Armendariz-Borunda, J. The Role of NRF2 in Obesity-Associated Cardiovascular Risk Factors. *Antioxidants* 2022, *11*, 235.
347. Shalova, I.N.; Medoro, A.; Davinelli, S.; Bender, N.; Saso, L. Nrf2 in Obesity: Mechanisms, Models, and Bioactive Modulators. *Obes. Res. Clin. Pract.* **2026**, *20*, 375–395, doi:https://doi.org/10.1016/j.orcp.2026.06.004.

348. Bodur, M.; Yilmaz, B.; Ağagündüz, D.; Ozogul, Y. Immunomodulatory Effects of Omega-3 Fatty Acids: Mechanistic Insights and Health Implications. *Mol. Nutr. Food Res.* **2025**, *69*, e202400752, doi:10.1002/mnfr.202400752.
349. Takić, M.; Ranković, S.; Girek, Z.; Pavlović, S.; Jovanović, P.; Jovanović, V.; Šarac, I. Current Insights into the Effects of Dietary α-Linolenic Acid Focusing on Alterations of Polyunsaturated Fatty Acid Profiles in Metabolic Syndrome. *Int. J. Mol. Sci.* **2024**, *25*, doi:10.3390/ijms25094909.
350. Innes, J.K.; Calder, P.C. Omega-6 Fatty Acids and Inflammation. *Prostaglandins. Leukot. Essent. Fatty Acids* **2018**, *132*, 41–48, doi:10.1016/j.plefa.2018.03.004.
351. Salvi, V.; Vaira, X.; Gianello, V.; Vermi, W.; Bugatti, M.; Sozzani, S.; Bosisio, D. TLR Signalling Pathways Diverge in Their Ability to Induce PGE2. *Mediators Inflamm.* **2016**, *2016*, 5678046, doi:10.1155/2016/5678046.
352. Serio, K.J.; Reddy, K.V.; Bigby, T.D. Lipopolysaccharide Induces 5-Lipoxygenase-Activating Protein Gene Expression in THP-1 Cells via a NF-KappaB and C/EBP-Mediated Mechanism. *Am. J. Physiol. Cell Physiol.* **2005**, *288*, C1125-33, doi:10.1152/ajpcell.00296.2004.
353. Yan, Y.; Jiang, W.; Spinetti, T.; Tardivel, A.; Castillo, R.; Bourquin, C.; Guarda, G.; Tian, Z.; Tschopp, J.; Zhou, R. Omega-3 Fatty Acids Prevent Inflammation and Metabolic Disorder through Inhibition of NLRP3 Inflammasome Activation. *Immunity* **2013**, *38*, 1154–1163, doi:10.1016/j.immuni.2013.05.015.
354. Downer, E.J. Role of Cannabinoids in Inflammation. *Molecules* 2022, *27*.
355. Klein, T.W. Cannabinoid-Based Drugs as Anti-Inflammatory Therapeutics. *Nat. Rev. Immunol.* **2005**, *5*, 400–411, doi:10.1038/nri1602.
356. Turcotte, C.; Blanchet, M.-R.; Laviolette, M.; Flamand, N. The CB(2) Receptor and Its Role as a Regulator of Inflammation. *Cell. Mol. Life Sci.* **2016**, *73*, 4449–4470, doi:10.1007/s00018-016-2300-4.
357. Almogi-Hazan, O.; Or, R. Cannabis, the Endocannabinoid System and Immunity-the Journey from the Bedside to the Bench and Back. *Int. J. Mol. Sci.* **2020**, *21*, doi:10.3390/ijms21124448.
358. Nandi, S.K.; Basu, S.; Bhattacharjya, A.; Dey Ghosh, R.; Bose, C.K.; Mukhopadhyay, S.; Bhattacharya, R. Interplay of Gut Microbiome, Fatty Acids, and the Endocannabinoid System in Regulating Development, Progression, Immunomodulation, and Chemoresistance of Cancer. *Nutrition* **2022**, *103–104*, 111787, doi:10.1016/j.nut.2022.111787.
359. Cluny, N.L.; Reimer, R.A.; Sharkey, K.A. Cannabinoid Signalling Regulates Inflammation and Energy Balance: The Importance of the Brain-Gut Axis. *Brain. Behav. Immun.* **2012**, *26*, 691–698, doi:10.1016/j.bbi.2012.01.004.
360. Watson, J.E.; Kim, J.S.; Das, A. Emerging Class of Omega-3 Fatty Acid Endocannabinoids & Their Derivatives. *Prostaglandins Other Lipid Mediat.* **2019**, *143*, 106337, doi:10.1016/j.prostaglandins.2019.106337.
361. Dos S Silva, P.; Butenko, Y.; Kra, G.; Malitsky, S.; Itkin, M.; Levin, Y.; Moallem, U.; Zachut, M. Omega-3 Fatty Acid Supplementation from Late Pregnancy to Early Lactation Attenuates the Endocannabinoid System and Immune Proteome in Preovulatory Follicles and Endometrium of Holstein Dairy Cows. *J. Dairy Sci.* **2025**, *108*, 4299–4317, doi:10.3168/jds.2024-25409.
362. Ghosh, S.S.S.; Wang, J.; Yannie, P.J.; Ghosh, S.S.S. Intestinal Barrier Dysfunction, LPS Translocation, and Disease Development. *J. Endocr. Soc.* **2020**, *4*, bvz039, doi:10.1210/jendso/bvz039.
363. Młynarska, E.; Wasiak, J.; Gajewska, A.; Steć, G.; Jasińska, J.; Rysz, J.; Franczyk, B. Exploring the Significance of Gut Microbiota in Diabetes Pathogenesis and Management-A Narrative Review. *Nutrients* **2024**, *16*, doi:10.3390/nu16121938.
364. Boutagy, N.E.; McMillan, R.P.; Frisard, M.I.; Hulver, M.W. Metabolic Endotoxemia with Obesity: Is It Real and Is It Relevant? *Biochimie* **2016**, *124*, 11–20, doi:10.1016/j.biochi.2015.06.020.
365. Rocha, D.M.; Bressan, J.; Hermsdorff, H.H. The Role of Dietary Fatty Acid Intake in Inflammatory Gene Expression: A Critical Review. *Sao Paulo Med. J.* **2017**, *135*, 157–168, doi:10.1590/1516-3180.2016.008607072016.
366. David, L.A.; Maurice, C.F.; Carmody, R.N.; Gootenberg, D.B.; Button, J.E.; Wolfe, B.E.; Ling, A. V; Devlin, A.S.; Varma, Y.; Fischbach, M.A.; et al. Diet Rapidly and Reproducibly Alters the Human Gut Microbiome. *Nature* **2014**, *505*, 559–563, doi:10.1038/nature12820.

367. Rohr, M.W.; Narasimhulu, C.A.; Rudeski-Rohr, T.A.; Parthasarathy, S. Negative Effects of a High-Fat Diet on Intestinal Permeability: A Review. *Adv. Nutr.* **2020**, *11*, 77–91, doi:10.1093/advances/nmz061.
368. Windey, K.; De Preter, V.; Verbeke, K. Relevance of Protein Fermentation to Gut Health. *Mol. Nutr. Food Res.* **2012**, *56*, 184–196, doi:10.1002/mnfr.201100542.
369. Rocha, D.M.; Caldas, A.P.; Oliveira, L.L.; Bressan, J.; Hermsdorff, H.H. Saturated Fatty Acids Trigger TLR4-Mediated Inflammatory Response. *Atherosclerosis* **2016**, *244*, 211–215, doi:10.1016/j.atherosclerosis.2015.11.015.
370. Emerson, S.R.; Kurti, S.P.; Harms, C.A.; Haub, M.D.; Melgarejo, T.; Logan, C.; Rosenkranz, S.K. Magnitude and Timing of the Postprandial Inflammatory Response to a High-Fat Meal in Healthy Adults: A Systematic Review. *Adv. Nutr.* **2017**, *8*, 213–225, doi:https://doi.org/10.3945/an.116.014431.
371. Laugerette, F.; Vors, C.; Géloën, A.; Chauvin, M.-A.; Soulage, C.; Lambert-Porcheron, S.; Peretti, N.; Alligier, M.; Burcelin, R.; Laville, M.; et al. Emulsified Lipids Increase Endotoxemia: Possible Role in Early Postprandial Low-Grade Inflammation. *J. Nutr. Biochem.* **2011**, *22*, 53–59, doi:10.1016/j.jnutbio.2009.11.011.
372. Mo, Z.; Huang, S.; Burnett, D.J.; Rutledge, J.C.; Hwang, D.H. Endotoxin May Not Be the Major Cause of Postprandial Inflammation in Adults Who Consume a Single High-Fat or Moderately High-Fat Meal. *J. Nutr.* **2020**, *150*, 1303–1312, doi:https://doi.org/10.1093/jn/nxaa003.
373. Ghoshal, S.; Witta, J.; Zhong, J.; de Villiers, W.; Eckhardt, E. Chylomicrons Promote Intestinal Absorption of Lipopolysaccharides. *J. Lipid Res.* **2009**, *50*, 90–97, doi:10.1194/jlr.M800156-JLR200.
374. Calder, P.C. Marine Omega-3 Fatty Acids and Inflammatory Processes: Effects, Mechanisms and Clinical Relevance. *Biochim. Biophys. Acta* **2015**, *1851*, 469–484, doi:10.1016/j.bbalip.2014.08.010.
375. Quarta, S.; Santarpino, G.; Calabriso, N.; Carluccio, M.A.; Siracusa, L.; Strano, T.; Cardetta, F.; Siculella, L.; Damiano, F.; De Caterina, R.; et al. Omega-3 PUFAs Reduce Inflammation by Targeting NRF2 and NF-KB Activity in an Ex Vivo Model of Cardiac Mature Adipocytes and Adipose Derived Stem Cells from Atherosclerotic Patients. *Food Funct.* **2026**, *17*, 1431–1450, doi:10.1039/d5fo03219a.
376. Golpour, P.; Nourbakhsh, M.M.; Mazaherioun, M.; Janani, L.; Nourbakhsh, M.M.; Yaghmaei, P. Improvement of NRF2 Gene Expression and Antioxidant Status in Patients with Type 2 Diabetes Mellitus after Supplementation with Omega-3 Polyunsaturated Fatty Acids: A Double-Blind Randomised Placebo-Controlled Clinical Trial. *Diabetes Res. Clin. Pract.* **2020**, *162*, 108120, doi:10.1016/j.diabres.2020.108120.
377. Poli, A.; Agostoni, C.; Visioli, F. Dietary Fatty Acids and Inflammation: Focus on the n-6 Series. *Int. J. Mol. Sci.* 2023, *24*, 4567.
378. Oteng, A.-B.; Kersten, S. Mechanisms of Action of Trans Fatty Acids. *Adv. Nutr.* **2020**, *11*, 697–708, doi:10.1093/advances/nmz125.
379. Fessler, M.B. The Intracellular Cholesterol Landscape: Dynamic Integrator of the Immune Response. *Trends Immunol.* **2016**, *37*, 819–830, doi:10.1016/j.it.2016.09.001.
380. Rajamäki, K.; Lappalainen, J.; Oörni, K.; Välimäki, E.; Matikainen, S.; Kovanen, P.T.; Eklund, K.K. Cholesterol Crystals Activate the NLRP3 Inflammasome in Human Macrophages: A Novel Link between Cholesterol Metabolism and Inflammation. *PLoS One* **2010**, *5*, e11765, doi:10.1371/journal.pone.0011765.
381. Duewell, P.; Kono, H.; Rayner, K.J.; Sirois, C.M.; Vladimer, G.; Bauernfeind, F.G.; Abela, G.S.; Franchi, L.; Nuñez, G.; Schnurr, M.; et al. NLRP3 Inflammasomes Are Required for Atherogenesis and Activated by Cholesterol Crystals. *Nature* **2010**, *464*, 1357–1361, doi:10.1038/nature08938.
382. Draganidis, D.; Karagounis, L.G.; Athanailidis, I.; Chatzinikolaou, A.; Jamurtas, A.Z.; Fatouros, I.G. Inflammaging and Skeletal Muscle: Can Protein Intake Make a Difference? *J. Nutr.* **2016**, *146*, 1940–1952, doi:https://doi.org/10.3945/jn.116.230912.
383. Holmer-Jensen, J.; Karhu, T.; Mortensen, L.S.; Pedersen, S.B.; Herzig, K.-H.; Hermansen, K. Differential Effects of Dietary Protein Sources on Postprandial Low-Grade Inflammation after a Single High Fat Meal in Obese Non-Diabetic Subjects. *Nutr. J.* **2011**, *10*, 115, doi:10.1186/1475-2891-10-115.
384. Kinyi, H.W.; Drago, C.K.; Ochola, L.; Kiwanuka, G.N. High-Protein Diets Reduce Plasma pro-Inflammatory Cytokines

Following Lipopolysaccharide Challenge in Swiss Albino Mice. *PLoS One* **2025**, *20*, e0338588, doi:10.1371/journal.pone.0338588.
385. Hruby, A.; Jacques, P.F. Dietary Protein and Changes in Biomarkers of Inflammation and Oxidative Stress in the Framingham Heart Study Offspring Cohort. *Curr. Dev. Nutr.* **2019**, *3*, nzz019, doi:10.1093/cdn/nzz019.
386. Scuto, M.C.; Anfuso, C.D.; Lombardo, C.; Di Fatta, E.; Ferri, R.; Musso, N.; Zerbo, G.; Terrana, M.; Majzúnová, M.; Lupo, G.; et al. Neuronutrition and Nrf2 Brain Resilience Signaling: Epigenomics and Metabolomics for Personalized Medicine in Nervous System Disorders from Bench to Clinic. *Int. J. Mol. Sci.* **2025**, *26*, doi:10.3390/ijms26199391.
387. Gao, J.; Xu, K.; Liu, H.; Liu, G.; Bai, M.; Peng, C.; Li, T.; Yin, Y. Impact of the Gut Microbiota on Intestinal Immunity Mediated by Tryptophan Metabolism. *Front. Cell. Infect. Microbiol.* **2018**, *8*, 13, doi:10.3389/fcimb.2018.00013.
388. Zelante, T.; Iannitti, R.G.; Cunha, C.; De Luca, A.; Giovannini, G.; Pieraccini, G.; Zecchi, R.; D'Angelo, C.; Massi-Benedetti, C.; Fallarino, F.; et al. Tryptophan Catabolites from Microbiota Engage Aryl Hydrocarbon Receptor and Balance Mucosal Reactivity via Interleukin-22. *Immunity* **2013**, *39*, 372–385, doi:10.1016/j.immuni.2013.08.003.
389. Li, S.-Y.; Lu, Z.-H.; Leung, J.C.S.; Kwok, T.C.Y. Association of Dietary Protein Intake, Inflammation with Muscle Mass, Physical Performance and Incident Sarcopenia in Chinese Community-Dwelling Older Adults. *J. Nutr. Health Aging* **2024**, *28*, 100163, doi:10.1016/j.jnha.2024.100163.
390. Zhang, X.; Sergin, I.; Evans, T.D.; Jeong, S.-J.; Rodriguez-Velez, A.; Kapoor, D.; Chen, S.; Song, E.; Holloway, K.B.; Crowley, J.R.; et al. High-Protein Diets Increase Cardiovascular Risk by Activating Macrophage MTOR to Suppress Mitophagy. *Nat. Metab.* **2020**, *2*, 110–125, doi:10.1038/s42255-019-0162-4.
391. Päivärinta, E.; Itkonen, S.T.; Pellinen, T.; Lehtovirta, M.; Erkkola, M.; Pajari, A.-M. Replacing Animal-Based Proteins with Plant-Based Proteins Changes the Composition of a Whole Nordic Diet-A Randomised Clinical Trial in Healthy Finnish Adults. *Nutrients* **2020**, *12*, doi:10.3390/nu12040943.
392. Kitada, M.; Ogura, Y.; Monno, I.; Koya, D. The Impact of Dietary Protein Intake on Longevity and Metabolic Health. *EBioMedicine* **2019**, *43*, 632–640, doi:10.1016/j.ebiom.2019.04.005.
393. Jain, M.; Celis-Morales, C.; Ozanne, S.E.; Burden, S.; Gray, S.R.; Morrison, D.J. Protein Source, Dietary Fibre Intake, and Inflammation in Older Adults: A UK Biobank Study. *Nutrients* **2025**, *17*, doi:10.3390/nu17091454.
394. Wang, S.-N.; Liu, Y.-X.; Sun, G.-Y.; Liu, X.; Sun, Y.; Ma, Y.-X.; Ning, S.-Y.; Shi, Y. Branched-Chain Amino Acids from Plants and the Metabolic Syndrome: Pathways and Pharmacological Applications. *Front. Nutr.* **2026**, *13*, 1805807, doi:10.3389/fnut.2026.1805807.
395. Ramos-Lopez, O.; Martinez-Urbistondo, D.; Vargas-Nuñez, J.A.; Martinez, J.A. The Role of Nutrition on Meta-Inflammation: Insights and Potential Targets in Communicable and Chronic Disease Management. *Curr. Obes. Rep.* **2022**, *11*, 305–335, doi:10.1007/s13679-022-00490-0.
396. Demeyer, D.; Mertens, B.; De Smet, S.; Ulens, M. Mechanisms Linking Colorectal Cancer to the Consumption of (Processed) Red Meat: A Review. *Crit. Rev. Food Sci. Nutr.* **2016**, *56*, 2747–2766, doi:10.1080/10408398.2013.873886.
397. Chelakkot, C.; Ghim, J.; Ryu, S.H. Mechanisms Regulating Intestinal Barrier Integrity and Its Pathological Implications. *Exp. Mol. Med.* **2018**, *50*, 1–9, doi:10.1038/s12276-018-0126-x.
398. Whelan, K.; Bancil, A.S.; Lindsay, J.O.; Chassaing, B. Ultra-Processed Foods and Food Additives in Gut Health and Disease. *Nat. Rev. Gastroenterol. Hepatol.* **2024**, *21*, 406–427, doi:10.1038/s41575-024-00893-5.
399. Kruger, C.; Zhou, Y. Red Meat and Colon Cancer: A Review of Mechanistic Evidence for Heme in the Context of Risk Assessment Methodology. *Food Chem. Toxicol.* **2018**, *118*, 131–153, doi:https://doi.org/10.1016/j.fct.2018.04.048.
400. Bastide, N.M.; Chenni, F.; Audebert, M.; Santarelli, R.L.; Taché, S.; Naud, N.; Baradat, M.; Jouanin, I.; Surya, R.; Hobbs, D.A.; et al. A Central Role for Heme Iron in Colon Carcinogenesis Associated with Red Meat Intake. *Cancer Res.* **2015**, *75*, 870–879, doi:10.1158/0008-5472.CAN-14-2554.
401. Bastide, N.; Morois, S.; Cadeau, C.; Kangas, S.; Serafini, M.; Gusto, G.; Dossus, L.; Pierre, F.H.; Clavel-Chapelon, F.; Boutron-Ruault, M.-C. Heme Iron Intake, Dietary Antioxidant Capacity, and Risk of Colorectal Adenomas in a Large Cohort Study

of French Women. *Cancer Epidemiol. Biomarkers Prev.* **2016**, *25*, 640–647, doi:10.1158/1055-9965.EPI-15-0724.

402. Islam, M.A.; Alam, F.; Solayman, M.; Khalil, M.I.; Kamal, M.A.; Gan, S.H. Dietary Phytochemicals: Natural Swords Combating Inflammation and Oxidation-Mediated Degenerative Diseases. *Oxid. Med. Cell. Longev.* **2016**, *2016*, 5137431, doi:10.1155/2016/5137431.
403. Zhu, F.; Du, B.; Xu, B. Anti-Inflammatory Effects of Phytochemicals from Fruits, Vegetables, and Food Legumes: A Review. *Crit. Rev. Food Sci. Nutr.* **2018**, *58*, 1260–1270, doi:10.1080/10408398.2016.1251390.
404. Zhang, Q.; Wu, D.; Guo, F.; Yang, S.; Bao, L.; Zhang, R.; Wang, P. Dietary Fiber and Metabolic Syndrome in NHANES: Mediation through Inflammation and Modifications by Population Characteristics. *Nutr. Metab. (Lond).* **2026**, *23*, 21, doi:10.1186/s12986-026-01074-8.
405. Zinöcker, M.K.; Lindseth, I.A. The Western Diet-Microbiome-Host Interaction and Its Role in Metabolic Disease. *Nutrients* **2018**, *10*, doi:10.3390/nu10030365.
406. Dinarello, C.A. Interleukin-1 in the Pathogenesis and Treatment of Inflammatory Diseases. *Blood* **2011**, *117*, 3720–3732, doi:10.1182/blood-2010-07-273417.
407. van Eijk, L.T.; Nooteboom, A.; Hendriks, T.; Sprong, T.; Netea, M.G.; Smits, P.; van der Hoeven, J.G.; Pickkers, P. Plasma Obtained during Human Endotoxemia Increases Endothelial Albumin Permeability in Vitro. *Shock* **2006**, *25*, 358–362, doi:10.1097/01.shk.0000209527.35743.b0.
408. Peter, H.; B., L.G.; Hermann, W.; Klaus, P.; Klaus, H. Role of Interleukin-18 (IL-18) during Lethal Shock: Decreased Lipopolysaccharide Sensitivity but Normal Superantigen Reaction in IL-18-Deficient Mice. *Infect. Immun.* **2000**, *68*, 3502–3508, doi:10.1128/iai.68.6.3502-3508.2000.
409. Borge, B.Å.S.; Kalland, K.-H.; Olsen, S.; Bletsa, A.; Berggreen, E.; Wiig, H. Cytokines Are Produced Locally by Myocytes in Rat Skeletal Muscle during Endotoxemia. *Am. J. Physiol. Circ. Physiol.* **2009**, *296*, H735–H744, doi:10.1152/ajpheart.01309.2008.
410. Saluja, R.; Khan, M.; Church, M.K.; Maurer, M. The Role of IL-33 and Mast Cells in Allergy and Inflammation. *Clin. Transl. Allergy* **2015**, *5*, 33, doi:10.1186/s13601-015-0076-5.
411. Cayrol, C.; Girard, J.-P. Interleukin-33 (IL-33): A Nuclear Cytokine from the IL-1 Family. *Immunol. Rev.* **2018**, *281*, 154–168, doi:10.1111/imr.12619.
412. Parameswaran, N.; Patial, S. Tumor Necrosis Factor-α Signaling in Macrophages. *Crit. Rev. Eukaryot. Gene Expr.* **2010**, *20*, 87–103, doi:10.1615/critreveukargeneexpr.v20.i2.10.
413. Teeman, C.S.; Kurti, S.P.; Cull, B.J.; Emerson, S.R.; Haub, M.D.; Rosenkranz, S.K. The Effect of Moderate Intensity Exercise in the Postprandial Period on the Inflammatory Response to a High-Fat Meal: An Experimental Study. *Nutr. J.* **2016**, *15*, 24, doi:10.1186/s12937-016-0134-4.
414. Miglio, C.; Peluso, I.; Raguzzini, A.; Villaño, D. V; Cesqui, E.; Catasta, G.; Toti, E.; Serafini, M. Antioxidant and Inflammatory Response Following High-Fat Meal Consumption in Overweight Subjects. *Eur. J. Nutr.* **2013**, *52*, 1107–1114, doi:10.1007/s00394-012-0420-7.
415. Burdge, G.C.; Calder, P.C. Plasma Cytokine Response during the Postprandial Period: A Potential Causal Process in Vascular Disease? *Br. J. Nutr.* **2005**, *93*, 3–9, doi:10.1079/bjn20041282.
416. Peluso, I.; Raguzzini, A.; Villano, D. V; Cesqui, E.; Toti, E.; Catasta, G.; Serafini, M. High Fat Meal Increase of IL-17 Is Prevented by Ingestion of Fruit Juice Drink in Healthy Overweight Subjects. *Curr. Pharm. Des.* **2012**, *18*, 85–90, doi:10.2174/138161212798919020.
417. Tanaka, T.; Narazaki, M.; Kishimoto, T. IL-6 in Inflammation, Immunity, and Disease. *Cold Spring Harb. Perspect. Biol.* **2014**, *6*, a016295, doi:10.1101/cshperspect.a016295.
418. Rose-John, S. Interleukin-6 Family Cytokines. *Cold Spring Harb. Perspect. Biol.* **2018**, *10*, doi:10.1101/cshperspect.a028415.
419. Lundman, P.; Boquist, S.; Samnegård, A.; Bennermo, M.; Held, C.; Ericsson, C.-G.; Silveira, A.; Hamsten, A.; Tornvall, P. A High-Fat Meal Is Accompanied by Increased Plasma Interleukin-6 Concentrations. *Nutr. Metab. Cardiovasc. Dis.* **2007**, *17*, 195–202, doi:10.1016/j.numecd.2005.11.009.

420. Vignali, D.A.A.; Kuchroo, V.K. IL-12 Family Cytokines: Immunological Playmakers. *Nat. Immunol.* **2012**, *13*, 722–728, doi:10.1038/ni.2366.
421. Gee, K.; Guzzo, C.; Che Mat, N.F.; Ma, W.; Kumar, A. The IL-12 Family of Cytokines in Infection, Inflammation and Autoimmune Disorders. *Inflamm. Allergy Drug Targets* **2009**, *8*, 40–52, doi:10.2174/187152809787582507.
422. Watford, W.T.; Moriguchi, M.; Morinobu, A.; O'Shea, J.J. The Biology of IL-12: Coordinating Innate and Adaptive Immune Responses. *Cytokine Growth Factor Rev.* **2003**, *14*, 361–368, doi:10.1016/s1359-6101(03)00043-1.
423. Trinchieri, G. Interleukin-12 and the Regulation of Innate Resistance and Adaptive Immunity. *Nat. Rev. Immunol.* **2003**, *3*, 133–146, doi:10.1038/nri1001.
424. Vazquez-Madrigal, C.; Lopez, S.; Grao-Cruces, E.; Millan-Linares, M.C.; Rodriguez-Martin, N.M.; Martin, M.E.; Alba, G.; Santa-Maria, C.; Bermudez, B.; Montserrat-de la Paz, S. Dietary Fatty Acids in Postprandial Triglyceride-Rich Lipoproteins Modulate Human Monocyte-Derived Dendritic Cell Maturation and Activation. *Nutrients* 2020, *12*, 3139.
425. Madsen, P.A.; Jerez-Bogotá, K.; Vodolazska, D.; Lauridsen, C. Comparative Study of Cytokine Measurements in Blood Plasma and Serum, and Saliva of Juvenile Pigs During Experimentally Induced Acute Inflammation. *Vet. Sci.* **2026**, *13*, doi:10.3390/vetsci13010068.
426. McGeachy, M.J.; Cua, D.J. The Link between IL-23 and Th17 Cell-Mediated Immune Pathologies. *Semin. Immunol.* **2007**, *19*, 372–376, doi:10.1016/j.smim.2007.10.012.
427. Iwakura, Y.; Ishigame, H. The IL-23/IL-17 Axis in Inflammation. *J. Clin. Invest.* **2006**, *116*, 1218–1222, doi:10.1172/JCI28508.
428. Emerson, S.R.; Sciarrillo, C.M.; Kurti, S.P.; Emerson, E.M.; Rosenkranz, S.K. High-Fat Meal–Induced Changes in Markers of Inflammation and Angiogenesis in Healthy Adults Who Differ by Age and Physical Activity Level. *Curr. Dev. Nutr.* **2019**, *3*, nzy098, doi:https://doi.org/10.1093/cdn/nzy098.
429. Sun, J.; Zhang, S.; Zhang, X.; Zhang, X.; Dong, H.; Qian, Y. IL-17A Is Implicated in Lipopolysaccharide-Induced Neuroinflammation and Cognitive Impairment in Aged Rats via Microglial Activation. *J. Neuroinflammation* **2015**, *12*, 165, doi:10.1186/s12974-015-0394-5.
430. Gaffen, S.L. Structure and Signalling in the IL-17 Receptor Family. *Nat. Rev. Immunol.* **2009**, *9*, 556–567, doi:10.1038/nri2586.
431. Korn, T.; Bettelli, E.; Oukka, M.; Kuchroo, V.K. IL-17 and Th17 Cells. *Annu. Rev. Immunol.* **2009**, *27*, 485–517, doi:10.1146/annurev.immunol.021908.132710.
432. Rocha, D.M.U.P.; Lopes, L.L.; da Silva, A.; Oliveira, L.L.; Bressan, J.; Hermsdorff, H.H.M. Orange Juice Modulates Proinflammatory Cytokines after High-Fat Saturated Meal Consumption. *Food Funct.* **2017**, *8*, 4396–4403, doi:10.1039/c7fo01139c.
433. Brinkhoff, A.; Sieberichs, A.; Engler, H.; Dolff, S.; Benson, S.; Korth, J.; Schedlowski, M.; Kribben, A.; Witzke, O.; Wilde, B. Pro-Inflammatory Th1 and Th17 Cells Are Suppressed During Human Experimental Endotoxemia Whereas Anti-Inflammatory IL-10 Producing T-Cells Are Unaffected. *Front. Immunol.* **2018**, *Volume 9*-.
434. Schoenborn, J.R.; Wilson, C.B. Regulation of Interferon-Gamma during Innate and Adaptive Immune Responses. *Adv. Immunol.* **2007**, *96*, 41–101, doi:10.1016/S0065-2776(07)96002-2.
435. Ivashkiv, L.B.; Donlin, L.T. Regulation of Type I Interferon Responses. *Nat. Rev. Immunol.* **2014**, *14*, 36–49, doi:10.1038/nri3581.
436. Uematsu, S.; Akira, S. Toll-like Receptors and Type I Interferons. *J. Biol. Chem.* **2007**, *282*, 15319–15323, doi:10.1074/jbc.R700009200.
437. McNab, F.; Mayer-Barber, K.; Sher, A.; Wack, A.; O'Garra, A. Type I Interferons in Infectious Disease. *Nat. Rev. Immunol.* **2015**, *15*, 87–103, doi:10.1038/nri3787.
438. Richez, C.; Yasuda, K.; Watkins, A.A.; Akira, S.; Lafyatis, R.; van Seventer, J.M.; Rifkin, I.R. TLR4 Ligands Induce IFN-Alpha Production by Mouse Conventional Dendritic Cells and Human Monocytes after IFN-Beta Priming. *J. Immunol.* **2009**, *182*, 820–828, doi:10.4049/jimmunol.182.2.820.
439. Ouyang, W.; Rutz, S.; Crellin, N.K.; Valdez, P.A.; Hymowitz, S.G. Regulation and Functions of the IL-10 Family of Cytokines in Inflammation and Disease. *Annu. Rev. Immunol.* **2011**, *29*, 71–109, doi:10.1146/annurev-immunol-031210-101312.

440. Saraiva, M.; O'Garra, A. The Regulation of IL-10 Production by Immune Cells. *Nat. Rev. Immunol.* **2010**, *10*, 170–181, doi:10.1038/nri2711.
